# The minor chicken class I gene BF1 is deleted between short imperfect direct repeats in the B14 and typical B15 major histocompatibility complex (MHC) haplotypes

**DOI:** 10.1007/s00251-023-01313-9

**Published:** 2023-07-05

**Authors:** Nicolas I. E. Rocos, Felicity J. Coulter, Thomas C. J. Tan, Jim Kaufman

**Affiliations:** 1 Institute of Immunology and Infection Research, University of Edinburgh, Charlotte Auerbach Road, Edinburgh, EH9 3FL UK; 2 Department of Pathology, University of Cambridge, Tennis Court Road, Cambridge, CB2 1QP UK; 3 Current Address: Oregon Health and Science University, 3181 SW Sam Jackson Park Road, Portland, OR 97239 USA; 4 Current Address: Wellcome Centre for Cell Biology, Max Born Crescent, Edinburgh, EH9 3BF UK; 5 Department of Veterinary Medicine, University of Cambridge, Madingley Road, Cambridge, CB3 0ES UK

**Keywords:** Recombination, PacBio sequencing, Next generation sequencing

## Abstract

The chicken major histocompatibility complex (MHC, also known as the BF-BL region of the B locus) is notably small and simple with few genes, most of which are involved in antigen processing and presentation. There are two classical class I genes, of which only BF2 is well and systemically expressed as the major ligand for cytotoxic T lymphocytes (CTLs). The other class I gene, BF1, is believed to be primarily a natural killer (NK) cell ligand. Among most standard chicken MHC haplotypes examined in detail, BF1 is expressed tenfold less than BF2 at the RNA level due to defects in the promoter or in a splice site. However, in the B14 and typical B15 haplotypes, BF1 RNA was not detected, and here, we show that a deletion between imperfect 32 nucleotide direct repeats has removed the BF1 gene entirely. The phenotypic effects of not having a BF1 gene (particularly on resistance to infectious pathogens) have not been systematically explored, but such deletions between short direct repeats are also found in some BF1 promoters and in the 5′ untranslated region (5′UTR) of some BG genes found in the BG region of the B locus. Despite the opposite transcriptional orientation of homologous genes in the chicken MHC, which might prevent the loss of key genes from a minimal essential MHC, it appears that small direct repeats can still lead to deletion.

**Supplementary Information:**

The online version contains supplementary material available at 10.1007/s00251-023-01313-9.

## Introduction

In mammals, the major histocompatibility complex (MHC) is a large genomic region with much recombination and with hundreds of genes, among which are a few that encode highly polymorphic transplantation antigens, now known as classical class I and class II molecules (Trowsdale and Knight 2013). The classical class I molecules bind peptides which originate primarily from proteins in the cytoplasm and nucleus where viruses (and a few cytoplasmic bacteria) replicate and present them at the cell surface to CD8-bearing cytotoxic T lymphocytes (CTLs) as well as natural killer (NK) cells, which can kill infected cells acting as viral factories (as well as cells with other differences from normal self, including allogeneic cells in transplants) (Blum et al. 2013; Parham and Moffett 2013; Djaoud and Parham 2020).

In humans, there are three classical class I loci, each with thousands of alleles. HLA-A and HLA-B are the primary CTL ligands and are well expressed on most cells (but not on red blood cells), although a few HLA-A alleles and about half of HLA-B alleles can be recognised by NK cells (Trowsdale and Knight 2013; Parham and Moffett 2013; Djaoud and Parham 2020). HLA-C alleles are less well expressed on most cells (although they are very well expressed on placental trophoblasts, but for tissue remodelling rather than as cytotoxic targets) and all alleles act primarily as NK ligands (Trowsdale and Knight 2013; Parham and Moffett 2013; Djaoud and Parham 2020), although they can present peptides to CTLs (Das and Khakoo 2015; Saunders et al. 2015).

By contrast, in chickens, there are two classical class I loci (Kaufman et al. 1999), which have been characterised in detail for only a few chicken MHC haplotypes (Kaufman et al. 1992; Wallny et al. 2006; Shaw et al. 2007; Hosomichi et al. 2008), known in the literature as the standard haplotypes which were originally identified in white Leghorn chickens (Miller et al. 2004). The BF2 gene is well expressed on most cells including red blood cells and is considered the primary CTL ligand (Fulton et al. 1995; Thacker et al. 1995). The BF1 is much less well expressed with fewer alleles (Wallny et al. 2006; Shaw et al. 2007), and limited data suggest that BF1 molecules are primarily NK cell ligands (Ewald and Livant 2004; Livant et al. 2004; Kim et al. 2018). The level of RNA for most BF1 alleles examined is tenfold less than BF2 alleles due to deletions or mutations in the promoter region and in one case due to a splice site mutation (Wallny et al. 2006; Shaw et al. 2007; O’Neill et al. 2009). For two common haplotypes, B14 and the typical B15 found in most chickens examined, no BF1 RNA was detected (Wallny et al. 2006; Shaw et al. 2007). However, a B15 haplotype from a chicken line in Japan is reported to have an intact BF1 gene (Hosomichi et al. 2008).

In the first analyses of the class I genes from the B14 and typical B15 haplotypes, no BF1 gene was amplified from genomic DNA with a range of oligonucleotide primers, but Southern blots of genomic DNA showed a large band as though there had been an insertion (Shaw et al. 2007). An alternative explanation for the large band was polymorphism in the restriction enzyme sites, although no evidence for such changes was found. Thus, the nature of the defect in the BF1 gene in the B14 and typical B15 haplotypes has remained a mystery. In this report, we find that a deletion between imperfect 32 nucleotide direct repeats has removed the BF1 gene entirely.

## Materials and methods

### Short- and long-range amplification from genomic DNA

Erythrocytes were from frozen stocks taken from experimental chicken lines as described (Shaw et al. 2007) but sourced from the Roslin Institute in Edinburgh and kept at the University of Cambridge. Genomic DNA was extracted from erythrocytes using a salting-out procedure previously described (Shaw et al. 2007), and 100 ng/μl dilutions were made. For shorter amplicons from class I exon 2 to neighbouring genes, the amplification used 1 μl of DNA with 1.5 μl each of 10-μM primers (c241 at the end of DMB2 gene (AGTGATGGTGTTGGGGCTCAG), c75 in exon 2 of BF genes (CTCCTGCCCAGCTCAGCCTTC), c350 in the C4 gene (AGGAGATGTGAGGTGACATGGGTGACATG), Shaw et al. 2007), together with Platinum™ SuperFi™ II polymerase and SuperFi II Buffer (Invitrogen by Thermo Fisher Scientific, Cat. No. 12361050, Lot No. 01211903), as well as dNTP mix (10 mM each nucleotide) (Vazyme, Cat. No. P031-01) in a 50-µl reaction volume. Amplification conditions for the shorter sequences were 98 °C for 1 min, followed by 32 cycles of 10 s at 98 °C and 2.5 min at 72 °C, followed by a final extension at 72 °C for 5 min on a Bio-Rad T100 Thermal Cycler. For longer sequences encompassing the whole class I gene, we used sequences of published primers (Hosomichi et al. 2008), modified at the 5′ end to include PacBio tags (/5AmMC6/gcagtcgaacatgtagctgactcaggtcac and /5AmMC6/tggatcacttgtgcaagcatcacatcgtag for forward and reverse primers, respectively). The 5′ amino acid modifier C6 provides a free amino group (www.genelink.com/newsite/products/mod_detail.asp?modid=3) but was present so that in subsequent sequence library preparation (carried out by Edinburgh Genomics), unwanted ligation of non-barcoded amplicons to SMRTbell adaptors was prevented. The amplification mix included 1.5 μl of each 10-μM primer modified as described above (BF1_F1, UE189 at the end of DMB2 gene (CCTATTCCCCCAACAGGTTACGCCC), BF1_R3, UE190 at the end of TAP1 gene (ACAAGGGACCACAAGAGCTGTGCC), BF2_F2, UE191 at the end of TAP2 gene (TTCCATCGGGTGTCCTTCGCC), BF2_R5, UE192 in between BF2 and C4 genes (CACTGATCCCAAAGGAAGCCCTGG)), along with the polymerase, buffer, dNTP mix, and volume as above. The amplification conditions for long-range sequences were 98 °C for 1 min, followed by 34 cycles of 10 s at 98 °C and 5 min at 68 °C, followed by a final extension at 72 °C for 5 min. Amplified products were analysed by electrophoresis through a 1% agarose gel in 40-mM Tris, 20-mM acetate, and 1-mM EDTA buffer pH 8.6 (TAE) including Safe DNA Gel Stain (APExBIO, Cat. No. A8743) at 100 V for 60 min. Gels were imaged by D-DiGit Gel Scanner (LI-COR).

### Sequencing and analysis

The bands from gels like those described above were cut out and DNA extracted using a Gel DNA Recovery Kit (Zymoclean™, Cat. No. D4008). For PacBio sequencing, barcode oligonucleotides (pairs of forward and reverse barcode primers from PacBio Barcoded Universal F/R Primers Plate–96 v2, part number 101–629-100, numbered 86–94) were added on both ends by PCR under the same conditions as the amplification for longer sequences above; then, bands were isolated by electrophoresis and extraction as above and submitted to the local sequencing facility, Edinburgh Genomics, who created a SMRTbell library which was sequenced using a PacBio Sequel IIe SMRT Cell 8MZW in HiFi mode. Sequence data were processed using the pbAA GitHub repository (https://github.com/PacificBiosciences/pbAA) and further visualised and analysed with Geneious Prime 2022.2.2 (http://www.geneious.com/). The amplicons for B14 and B15 BF1 were shorter, so they were submitted to GeneWiz (Leipzig, Germany) for Sanger sequencing and analysed by BioEdit (Hall 1999). Alignments were performed using Clustal Omega (Sievers et al. 2011) hosted on the ENSEMBL website and by BioEdit.

## Results

**Fig. 1:**
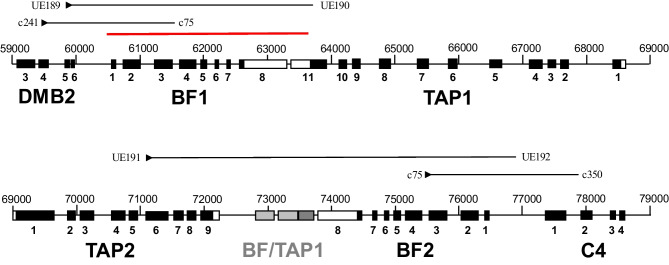
Depiction of part of the BF/BL region of the B locus of the B12 haplotype, from nucleotide 59,000 to 79,000 of accession number AL023516, covering part of the DMB2 gene (called B locus Mβ-chain 1 in the database entry) and the C4 gene, all of the BF1 (called BF2 in the database entry), BF2 (called BF1 in the database entry), TAP1 and TAP2 genes, along with the repeat of a portion of *BF* and *TAP1* genes between TAP2 and BF2 genes. Exons (numbered below) are depicted by boxes: black for protein coding regions, open for UTR, and grey for the unexpressed repeats. The names of the primers used and the locations that they should bind are indicated by solid black arrows; the location of the big deletion is indicated by a red line. Modified from Shaw et al. 2007

**Fig. 2:**
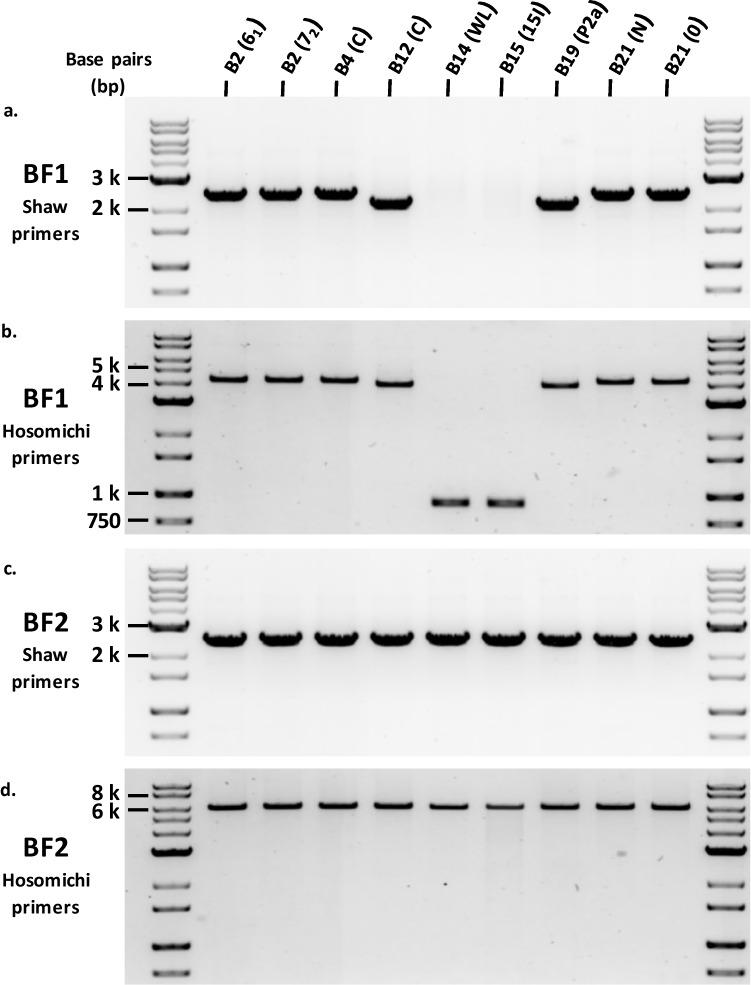
DNA gel electrophoresis of amplicons from genomic DNA shows that all BF2 genes are roughly the same size, while BF1 genes have a variety of sizes: the largest band for the B2, B4, and B21 haplotypes, a slightly smaller band from B12 and B19 haplotypes, and a much smaller band (or no band at all) from B14 and standard B15 haplotypes. Primers used were **a** c75 in BF1 and c241 in DMB2, **b** UE189 in DMB2 and UE190 in TAP1, **c** c75 in BF2 and c350 in C4, and **d** UE191 in TAP2 and UE192 in C4. Equal amounts of PCR reaction were loaded in each lane, except for the amplifications using BF2 Hosomichi primers for lines B14 and B15 (panel d), which required roughly 4 and 10 times as much material. Sources of DNA were lines 6_1_ (B2), 7_2_ (B2), C (B4), C (B12), WL (B14), 15I (B15), P2a (B19), N (B21) and 0 (B21)

Over 15 years ago, we reported the results of PCR using primers from adjacent genes to a conserved site in the chicken classical class I genes (the “Shaw primers”: DMB2 exon 4 to BF1 intron 3; C4 exon 2 to BF2 intron 3, Fig. 1), amplifying from genomic chicken DNA of experimental lines with common standard haplotypes (B2, B4, B12, B14, B15, B19 and B21). All BF2 reactions gave a single band of the same mobility on an agarose gel, while the BF1 reactions gave one band for B2, B4 and B21 DNA, a slightly smaller band for B12 and B19 DNA, and no band at all for B14 and B15 DNA (Shaw et al. 2007). Using the same primers with independent genomic DNA preparations from the same lines, we reproduced those findings (Fig. 2(a, c)).

Using primers between adjacent genes flanking the classical class I genes (the “Hosomichi primers”: DMB2 exon 5 to TAP1 exon 11; TAP2 exon 6 to the intergenic region between BF2 and C4, Fig. 1) (Hosomichi et al. 2008), we found single bands for all haplotypes. As expected, the bands from the Hosomichi primers were larger than those from the Shaw primers, but with the same relationships: the same size bands for BF2 from all MHC haplotypes, the same size bands for BF1 from B2, B4 and B21, and slightly smaller bands for BF1 from B12 and B19 (Fig. 2(b, d)). However, unlike the Shaw primers, the Hosomichi primers amplified bands for BF1 from B14 and B15, but much smaller than the 4–5 kB found for the other haplotypes (Fig. 2(b)), suggesting amplification across a big deletion.

**Fig. 3:**
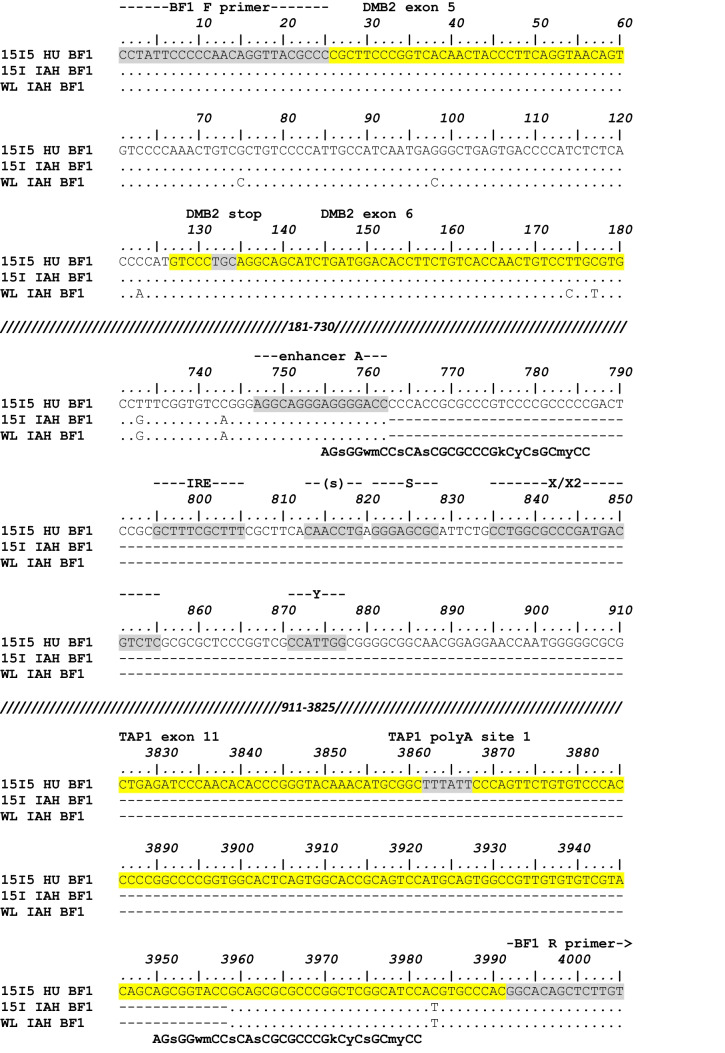
Sequence alignment of the amplified fragments including the BF1 gene from the lines with B14 (WL IAH BF1) and standard B15 (15I IAH BF1) haplotypes (from the Institute for Animal Health) shows a large deletion compared to the reported sequence from a line with an atypical B15 (15I_5_ HU BF1) haplotype (accession number AB426149 from Hiroshima University). Sequences numbered from start of amplicon from the atypical B15 haplotype, with lines of hash marks indicating sequences not depicted, with primer sites (BF1 F primer, UE189; BF1 R primer, UE190 from Hosomichi et al. 2008)) and other sequence features indicated by grey highlights and labelled above the line, except for exons highlighted in yellow. The location of the 32 nucleotide imperfect direct repeats is shown below the alignment using IUPAC single letter code in upper case, except for ambiguities in lower case (s, C or G; w, A or T; m, A or C; k, G or T; y, C or T)

We sequenced all these amplicons and compared them to other sequences in the literature (Figs. 3, S1, and S2). As expected (Shaw et al. 2007), we found excellent alignment of all BF2 gene sequences (Fig. S2). Also as expected, the BF1 sequences from B12 and B19 had a large deletion in the promoter region, running from the intergenic region to just after the enhancer A site, and the BF1 sequences from B2, B4 and B21 had apparent mutations in promoter elements compared to BF2 sequences, as well as some small deletions around the transcriptional start sites compared both to BF2 and to BF1 from B12 and B19 (Fig. S1).

However, the region in between DMB2 and TAP1 in the standard B14 and B15 haplotypes had suffered a deletion of 3196 nucleotides (red bar in Fig. 1), from the middle of the enhancer A site in the BF1 promoter to the 3′UTR of TAP1 (Fig. S1), particularly easy to see in comparison with the BF1 gene from an atypical B15 haplotype reported for a chicken line from Japan (Fig. 3). The start and end points of the big deletion appear identical in the B14 and standard B15 BF1 genes.

**Fig. 4:**
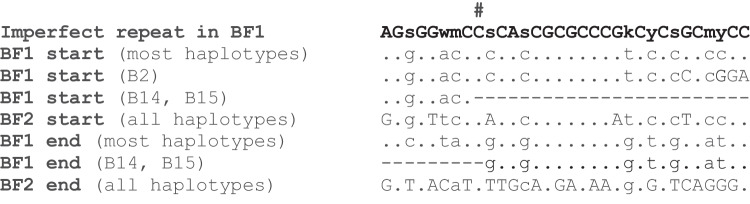
Alignment of the sequences found in the imperfect repeats shows that all BF1 genes but no BF2 genes should be susceptible to deletion between these sites. The motif for the imperfect 32 nucleotide repeat (bold) is shown with the IUPAC single letter code in upper case and ambiguities in lower case (s, C or G; w, A or T; m, A or C; k, G or T; y, C or T), with the sequences of the BF1 and BF2 genes in the position of the repeats which are at the 5′ (start) and 3′ (end) of the BF1 gene from the atypical B15 haplotype below (dots depict identities with the unambiguous nucleotides in the motif, lower case letters fit the ambiguous nucleotides in the motif, uppercase letters do not fit the motif, dashes indicate deleted nucleotides). The hash mark (#) indicates the position of the nucleotide lost during the recombination event

**Fig. 5:**
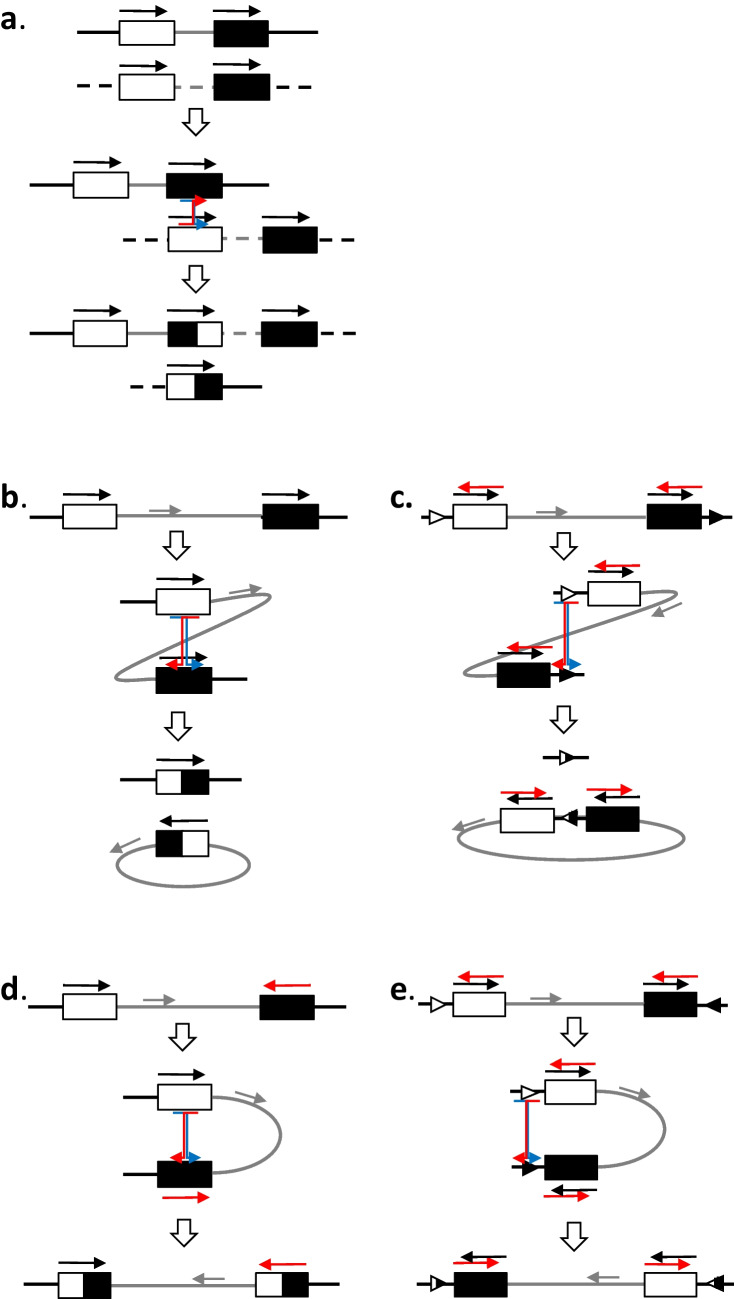
Various outcomes of homologous recombination are found for genes in the B locus. **a** Unequal recombination between similar genes on two chromosomes (sister chromatids) leads to expansion and contraction of multigene families, as seen for BG genes in the BG region. **b**, **c** Recombination on the same chromosome between genes in the same transcriptional orientation **b** or direct repeat elements **c **leads to deletion, giving rise to a hybrid gene **b** as seen for BG genes in the BG region or removal of genes **c** as seen for BF1 genes in certain MHC haplotypes. **d**, **e** Recombination on the same chromosome between genes in the opposite transcriptional orientation **d** or inverted repeat elements **e** leads to inversion, giving rise to hybrid genes without deletion **d** or intact genes without deletion **e,** as may be seen for BLB1 and BLB2 genes in certain MHC haplotypes. Solid lines indicate genomic DNA, with grey lines indicating intergenic DNA in all panels, dashed line indicating a homologous chromosome in **a**, and circles indicating deleted DNA in **b** and **c**. Boxes indicate genes and triangles indicate repeat elements: black, white, or hybrids with both. Arrows indicate transcriptional orientation of genes (black for forward, red for reverse, grey for potential genes in the intergenic regions), and triangles indicate orientation of repeat elements. Coloured box arrows (red and blue) indicate crossovers in recombination

Moreover, two imperfect 32 nucleotide direct repeats in the BF1 gene from the atypical BF1 haplotype overlap the start and end points of the deletion in the B14 and standard B15 haplotype, in such a way that there is only one imperfect 32 nucleotide repeat that overlaps the deletion in B14 and B15, although one nucleotide has been lost (Fig. 4). The reduction from two repeats at the boundaries of a stretch of DNA to just one repeat is exactly what is expected from recombination leading to deletion (Fig. 5c). The same repeat sequences are present in all BF1 genes, except in the B2 haplotype where a short deletion in the first repeat leads to four nucleotide differences at one end (Figs. 4 and S1). In contrast, BF2 lacks the second repeat altogether, so such a deletion would be most unlikely (Figs. 4 and S2). Interestingly, of the two perfect repeats that are associated with deletion of a portion of the BF1 promoter in the B12 and B19 haplotypes (Shaw et al. 2007), one is located directly after one of the imperfect repeats (Fig. S2).

## Discussion

Recombination is an important mechanism in genome evolution, which manifests in a variety of ways. The relative organisation of genes and sequence features can have an effect on the outcome of recombination in a multigene family (Fig. 5).

Examples of different outcomes of recombination have been found in the chicken B locus, which includes the BG genes in the BG region and the chicken MHC in the BF-BL region. For homologous recombination between similar sequences in the same orientation (that is, small direct repeats or homologous genes that are in the same transcriptional orientation) but on different chromosomes, the result can be hybrid genes with resulting expansion or contraction of the gene family (Fig. 5a), as is seen for the multigene family of BG genes (Salomonsen et al. 2014). For such homologous recombination between direct repeats on the same chromosome, the result can be deletion (Fig. 5c), as is seen for the BF1 promotor in the B12 and B19 MHC haplotypes (Shaw et al. 2007) and for the 5′ untranslated regions (5′UTRs) of tissue-expressed BG genes (Salomonsen et al. 2014).

**Fig. 6:**
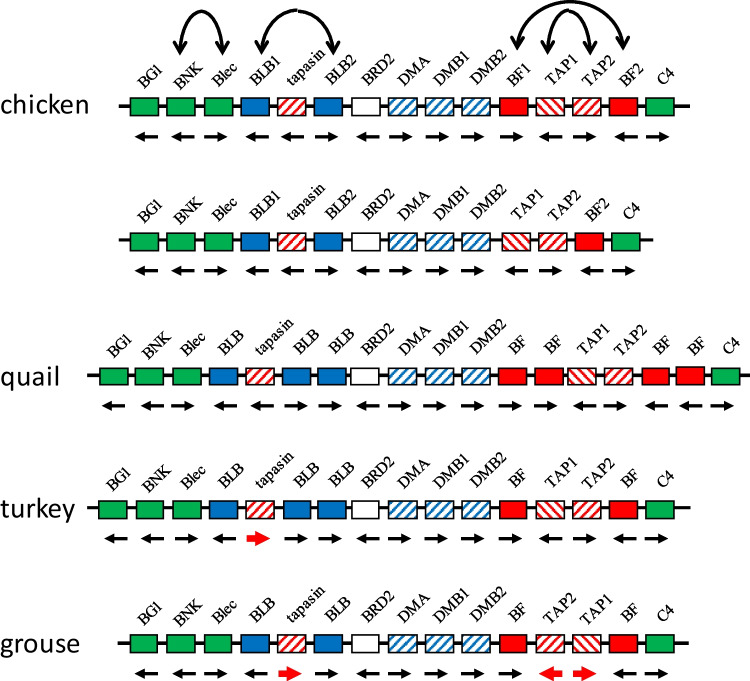
The chicken MHC (BF-BL region) with all the published genes compared to the chicken MHC with the BF1 deletion and with same regions in closely related birds, showing that the transcriptional orientation of the tapasin, TAP1, and TAP2 genes can reverse between species, most easily explained by inversion of flanking genes in opposite transcriptional orientation. Solid lines indicate genomic DNA, boxes indicate genes (red, class I system; blue, class II system; green, other immune genes; white, serine/threonine kinase gene BRD2; slanted lines, genes involved in peptide loading) with names above (except for BF1, BF2, BLB1 and BLB2 genes in chickens whose characteristics are well-described, the class I and class II B genes of other species indicated by BF and BLB, respectively), and transcriptional orientations indicated by arrows below (bold red indicates a switch compared to chickens). Double-headed arrows indicate homologous gene pairs in chickens. Figure modified from Kaufman et al. 1999) with data from this paper and from He et al. 2021)

For such homologous recombination on the same chromosome between genes in opposite transcriptional orientation or between inverted repeats, the result can be inversion (Fig. 5d, e), as may be the case for the BLB1 and BLB2 genes found in the chicken MHC (Afrache et al. 2020). Indeed, the fact that several pairs of genes with similar sequences are in opposite transcriptional orientation (BF1 and BF2, TAP1 and TAP2, BLB1 and BLB2, BNK and Blec, Fig. 6) has been interpreted as a way to prevent loss of essential genes from the compact and simple chicken MHC (Afrache et al. 2020; Kaufman 2023), originally described as a “minimal essential MHC” (Kaufman et al. 1995, 1999). Although such inversions have yet to be conclusively demonstrated for genes in the chicken MHC, the orientation of tapasin and TAP genes relative to flanking genes in closely related avian species (Fig. 6) is consistent with such inversion (He et al. 2021), as previously noted (Kaufman 2023).

However, despite the fact that several key gene pairs in the chicken MHC are in opposite transcriptional orientation which would be expected to protect against deletion, the lack of BF1 cDNA in two standard haplotypes, B14 and B15, was noted long ago (Kaufman et al. 1995; Wallny et al. 2006; Shaw et al. 2007). The attempts to understand the basis of this lack of expression at the level of genomic DNA by PCR using many primers that were successful for all other haplotypes led to no amplification of bands and by Southern blots led to the identification of much larger bands (Shaw et al. 2007). Two interpretations of the Southern blot data were that there had been changes in the restriction enzyme sites between haplotypes or that there had been an insertion. No changes in the sites were found in the sequences which were then available, which led to the tentative conclusion that an insertion was responsible, but no evidence for the inserted sequence was ever found.

In the present study using amplification of much longer DNA fragments, we find that a large deletion between the promoter of the BF1 gene and the end of the TAP1 gene is responsible for the lack of BF1 expression in the B14 and standard B15 haplotypes and that a direct repeat found in the correct positions in an atypical B15 haplotype is responsible for the deletion. Compared to the direct repeats in the BF1 promoter of the B12 and B19 haplotypes and the 5′UTRs of the tissue BG genes (Shaw et al. 2007; Salomonsen et al. 2014), the direct repeat is longer (32 compared to 10 and 27 nucleotides) and is also more imperfectly repeated (10 compared to 0 and around 3 ambiguities).

The deletion of a presumed “essential gene” in two standard haplotypes (found in perhaps billions of domestic chickens) was unexpected, so how commonly would such a deletion occur? All the BF1 genes examined should be susceptible to this deletion (in contrast to the BF2 genes), due to the presence of exactly the same repeat sequences (except for a few nucleotides in B2), and one of these repeats is located adjacent to the repeat associated with the deletion in the promoter of the B12 and B19 haplotypes (Fig. S1), perhaps indicating that the chromatin might be more open for recombination around promoter sites. However, the promoter deletion apparently happened only once (since the BF1 gene in B19 is derived from the B12 haplotype), and only two of the standard haplotypes (B14 and B15) have deleted the BF1 gene entirely. Moreover, the deletion in the B14 and typical B15 haplotypes appears identical (Fig. 3, S1). Indeed, the 14 single nucleotide polymorphisms (SNPs) in the sequences of B14, standard B15, and unusual B15 haplotypes compared to other haplotypes (Fig. S1) are spatially segregated, with SNPs unique to B14 and SNPs shared between the two B15 haplotypes found in the DM gene and the beginning of the intergenic region, followed by a cluster of SNPs that are shared between all three haplotypes, and finally two SNPs and the big deletion that are shared between B14 and the standard B15 haplotype. Together, the final two SNPs and big deletion might be taken as evidence that there was a single deletion in one haplotype that was transferred to the other haplotype by homologous recombination. However, in the absence of a greater number of informative features, it remains possible that there were independent deletions in the two haplotypes.

Another approach to determine the frequency of similar but independent deletions would be to examine a variety of other MHC haplotypes. An ongoing typing exercise has identified nearly 250 MHC haplotypes in chickens, of which 28 lack an obvious BF1 gene and four appear to lack one BLB gene (Tregaskes et al. 2023). A closer look at these other haplotypes is necessary to determine whether these apparent BF1 deletions have the same basis as the ones described here. Moreover, the potential effects of such “natural gene knock-outs” on resistance to infectious pathogens such as Marek’s disease virus have never been assessed.

### Supplementary Information

Below is the link to the electronic supplementary material.

Supplementary file1 (DOCX 352 KB)

## Data Availability

Supplementary figures show that the PacBio data reported in this publication is nearly identical to previously reported sequences available in the GenBank database, but all primary data in this publication is available upon request.
