# Supplementary material for: The minor chicken class I gene BF1 is deleted between short imperfect direct repeats in the B14 and typical B15 major histocompatibility complex (MHC) haplotypes
Source: Immunogenetics. 2023 Jul 5;75(5):455–64. doi: 10.1007/s00251-023-01313-9 (PMC10514180; doi:10.1007/s00251-023-01313-9)
Supplement: Supplementary file 1 — Supplementary file1 (DOCX 352 KB) [file 251_2023_1313_MOESM1_ESM.docx]

Immunogenetics

The minor chicken class I gene BF1 is deleted between short imperfect direct repeats in the B14 and typical B15 major histocompatibility complex (MHC) haplotypes

Nicolas I. E. Rocos, Felicity J. Coulter, Ellen A. Palmer, Thomas C. J. Tan and Jim Kaufman

University of Edinburgh, jim.kaufman@ed.ac.uk

Supplementary text

In order to decide whether to submit the sequences reported in this publication to appropriate databases, we compared the new sequences with existing sequences from the literature (Kaufman et al., 1999; Shaw et al., 2007; Hosomichi et al 2008). Overall, all the sequences for each allele were almost identical, with the differences due most likely to sequencing errors, particular in runs of particular nucleotides (or, in the case of Hosomichi et al., 2008, from long runs of bases called as Ns), although differences in the genomes of birds separated by many generations or differences due to PCR errors cannot be ruled out. Taking the majority call as the correct one, PacBio did just as well as Sanger sequencing.

As expected from previous literature (Wallny et al., 2006; Shaw et al., 2007), the consensus sequences of BF1 and the flanking regions were virtually identical between B12 and B19 (C878T and T1721G, both in B12) and were almost identical between B4 and B21 (with differences between them at C109T, C123T, T195del, C196del, G1639A, T2113C, C2281T, C2235T for B4 along with a nine nucleotide deletion at 3567-3575 compared to all other sequences; C123A, C177T, G200A, T319C, G400A, T2032C, G2140A, C2182T, C2239T, A3092G, C3698T, T3883C and G3917A for B21 with many positions shared with the atypical B15 near the end; thus, most differences in the sequences flanking the BF1 gene). Most of the allelic differences were between B2 versus the rest of the alleles, or between B2, B12 and B19 versus B4, B14, B21 and the two B15 alleles.

Comparing the BF1 and the flanking regions for the B14, typical B15 and atypical B15 sequences, four differences are unique to B14 (G98C, C123A, C177T, C475G), three shared between the two B15 sequences (C75G, C174T, A305G), five shared between all three compared to other haplotypes (G570A, C575T, C587T, G613A, G630A), and two between B14 and the standard B15 (T733G, C743A) with an identical 3196 nucleotide deletion from 763-3958 in the atypical B15 sequence. Although there are not many data points, it would appear that B14 differs from the two B15 haplotypes in the DM gene, but then there is a cluster of variation common to all three haplotypes in the intergenic region followed by some variation shared between B14 and the standard B15 haplotype just before the BF1 promoter, including the big deletion itself.

The BF2 (and adjacent TAP2) gene sequences, unlike the BF1 sequences, are nearly co-linear, with only a few sites of short deletions, all but one located in introns or intragenic regions. As expected from previous literature (Wallny et al., 2006; Shaw et al., 2007), most of the variation is found in BF2 exon 2 and exon 3, with the intron between them nearly invariant. Also, the BF2 gene from B19 is most closely related to B15 (rather than B12 as in BF1), due to a recombination event in TAP2 upstream of the sequenced region depicted.

References

Hosomichi K et al. 2008. J Immunol 181: 3393-9. doi: 10.4049/jimmunol.181.5.3393. PMID: 18714011.

Kaufman J et al. 1999. Nature 401: 923-5. doi: 10.1038/44856. PMID: 10553909.

Shaw I et al. 2007. J Immunol 178: 5744-52. doi: 10.4049/jimmunol.178.9.5744. PMID: 17442958.

Wallny HJ et al. 2006. Proc Natl Acad Sci U S A 103: 1434-9. doi: 10.1073/pnas.0507386103. PMID: 16432226.

Figure legends

Fig. S1. Alignment of PacBio sequences of amplicons reported in this publication (from exon 5 of DMB2 through BF1 to exon 11 of TAP1) compared to other genomic sequences in the literature, with haplotypes/lines including B12/line CB (AL023516) from Kaufman et al 1999, B2/lines 6_1_ and 7_2_ (AM279336, AM279340), B4/line C and subline C-B4 (AM279337, AM279341), B19/P2a (AM279338), B21/lines N and 0 (AM279339, AM279342) from Shaw et al 2007, and B2/line 6_3_ (AB426141), B12/line C (AB426147), B19/line P (AB426151) and B21/line P (AB426152) from Hosomichi et al 2008. IUPAC single letter code is used (A, C, G, T and N), with dashes indicating deletions. Exons are highlighted in yellow, sequence features (primers, transcription factor binding sites, polyadenylation sites, start and stop codons) are highlighted in grey, locations of the imperfect repeat implicated in the deletion of the BF1 gene are highlighted in green, and of the perfect repeat implicated in the deletion within the promoter regions of B12 and 19 highlighted in blue. Presumed sequence errors based on comparison with multiple sequences are also highlighted in grey. Nucleotides and deletions that represent minor alleles are coloured (B2, red; B4 light green; B12 and B19, dark green; B14, light blue; B15, dark blue), B21 (dark red); B12 and B19 are considered as one allele since this region of B19 derives from a recombination with B12. The three nucleotides in the first block and the 11 nucleotides between enhancer A and the S boxes that missing in all sequences are due to sequence misalignments that were only corrected at a late stage of figure preparation.

Fig. S2. Alignment of PacBio sequences of amplicons reported in this publication (from just before the promoter for BF2 to exon 9 of TAP2) compared to other genomic sequences in the literature, with haplotypes/lines including B12/line CB (AL023516) from Kaufman et al 1999, B2/lines 6_1_ and 7_2_ (AM282692, AM282698), B4/line C and subline C-B4 (AM282699, AM282693), B19/P2a (AM232696), B21/lines N and 0 (AM282697, AM282697) from Shaw et al 2007, and B2/line 6_3_ (AB426141), B12/line C (AB426147), B19/line P (AB426151) and B21/line P (AB426152) from Hosomichi et al 2008. IUPAC single letter code is used (A, C, G, T and N), with dashes indicating deletions. Exons are highlighted in yellow, sequence features (primers, transcription factor binding sites, polyadenylation sites, start and stop codons) are highlighted in grey, and the locations corresponding to the imperfect repeat implicated in the deletion of the BF1 gene are highlighted in green. Presumed sequence errors based on comparison with multiple sequences are also highlighted in grey. Nucleotides and deletions that represent minor alleles are coloured (B2, red; B4 light green; B12 and B19, dark green; B14, light blue; B15, dark blue), B21 (dark red).

**-------BF1 F primer------ DMB2 exon 5**

B2_BF1_(AB426141) CCTATTCCCCCAACAGGTTACGCCCCGCTTCCCGGTCACAACTACCCTTCAGGTAAC 57

B2_BF1_(PacBio_6sub1) CCTATTCCCCCAACAGGTTACGCCCCGCTTCCCGGTCACAACTACCCTTCAGGTAAC 57

B2_BF1_(AM279336) 0

B2_BF1_(PacBio_7sub2) CCTATTCCCCCAACAGGTTACGCCCCGCTTCCCGGTCACAACTACCCTTCAGGTAAC 57

B2_BF1_(AM279340) 0

B4_BF1_(AM279341) 0

B4_BF1_(PacBio_C) CCTATTCCCCCAACAGGTTACGCCCCGCTTCCCGGTCACAACTACCCTTCAGGTAAC 57

B4_BF1_(AM279337) 0

B12_BF1_(AB426147) CCTATTCCCCCAACAGGTTACGCCCCGCTTCCCGGTCACAACTACCCTTCAGGTAAC 57

B12_BF1(PacBio_C) CCTATTCCCCCAACAGGTTACGCCCCGCTTCCCGGTCACAACTACCCTTCAGGTAAC 57

B12_BF1_(AL023516) CCTATTCCCCCAACAGGTTACGCCCCGCTTCCCGGTCACAACTACCCTTCAGGTAAC 57

B14_BF1_(PacBio_WL) CCTATTCCCCCAACAGGTTACGCCCCGCTTCCCGGTCACAACTACCCTTCAGGTAAC 57

B15_BF1_(AB426149) CCTATTCCCCCAACAGGTTACGCCCCGCTTCCCGGTCACAACTACCCTTCAGGTAAC 57

B15_BF1_(PacBio_15I) CCTATTCCCCCAACAGGTTACGCCCCGCTTCCCGGTCACAACTACCTTTCAGGTAAC 57

B19_BF1_(AB426151) CCTATTCCCCCAACAGGTTACGCCCCGCTTCCCGGTCACAACTACCCTTCAGGTAAC 57

B19_BF1_(PacBio_P2a) CCTATTCCCCCAACAGGTTACGCCCCGCTTCCCGGTCACAACTACCCTTCAGGTAAC 57

B19_BF1_(AM279338) 0

B21_BF1_(AB426152) CCTATTCCCCCAACAGGTTACGCCCCGCTTCCCGGTCACAACTACCCTTCAGGTAAC 57

B21_BF1_(PacBio_N) CCTATTCCCCCAACAGGTTACGCCCCGCTTCCCGGTCACAACTACCCTTCAGGTAAC 57

B21_BF1_(AM279339) 0

B21_BF1_(PacBio_0) CCTATTCCCCCAACAGGTTACGCCCCGCTTCCCGGTCACAACTACCCTTCAGGTAAC 57

B21_BF1_(AM279342) 0

B2_BF1_(AB426141) AGTGTCCCCAAACTGTCCCTGTCCCCATTGCCATCAATGAGGGCTGAGTGA**T**CCCATCTC 117

B2_BF1_(PacBio_6sub1) AGTGTCCCCAAACTGTCCCTGTCCCCATTGCCATCAATGAGGGCTGAGTGA**T**CCCATCTC 117

B2_BF1_(AM279336) 0

B2_BF1_(PacBio_7sub2) AGTGTCCCCAAACTGTCCCTGTCCCCATTGCCATCAATGAGGGCTGAGTGA**T**CCCATCTC 117

B2_BF1_(AM279340) 0

B4_BF1_(AM279341) 0

B4_BF1_(PacBio_C) AGTGTCCCCAAACTGTCCCTGTCCCCATTGCCATCAATGAGGGCTGAGTGA**T**CCCATCTC 117

B4_BF1_(AM279337) 0

B12_BF1_(AB426147) AGTGTCCCCAAACTGTCCCTGTCCCCATTGCCATCAATGAGGGCTGAGTGACCCCATCTC 117

B12_BF1(PacBio_C) AGTGTCCCCAAACTGTCCCTGTCCCCATTGCCATCAATGAGGGCTGAGTGACCCCATCTC 117

B12_BF1_(AL023516) AGTGTCCCCAAACTGTCCCTGTCCCCATTGCCATCAATGAGGGCTGAGTGACCCCATCTC 117

B14_BF1_(PacBio_WL) AGTGTCCCCAAACTGTCCCTGTCCCCATTGCCATCAATGA**C**GGCTGAGTGACCCCATCTC 117

B15_BF1_(AB426149) AGTGTCCCCAAACTGTC**G**CTGTCCCCATTGCCATCAATGAGGGCTGAGTGACCCCATCTC 117

B15_BF1_(PacBio_15I) AGTGTCCCCAAACTGTC**G**CTGTCCCCATTGCCATCAATGAGGGCTGAGTGACCCCATCTC 117

B19_BF1_(AB426151) AGTGTCCCCAAACTGTCCCTGTCCCCATTGCCATCAATGAGGGCTGAGTGACCCCATCTC 117

B19_BF1_(PacBio_P2a) AGTGTCCCCAAACTGTCCCTGTCCCCATTGCCATCAATGAGGGCTGAGTGACCCCATCTC 117

B19_BF1_(AM279338) 0

B21_BF1_(AB426152) AGTGTCCCCAAACTGTCCCTGTCCCCATTGCCATCAATGAGGGCTGAGTGACCCCATCTC 117

B21_BF1_(PacBio_N) AGTGTCCCCAAACTGTCCCTGTCCCCATTGCCATCAATGAGGGCTGAGTGACCCCATCTC 117

B21_BF1_(AM279339) 0

B21_BF1_(PacBio_0) AGTGTCCCCAAACTGTCCCTGTCCCCATTGCCATCAATGAGGGCTGAGTGACCCCATCTC 117

B21_BF1_(AM279342) 0

**DMB2 stop codon DMB2 exon 6**

B2_BF1_(AB426141) TCACCCCATGTCCCTGCAGGCAGCATCTGATGGACACCTTCTGTCACCAACTGTCCCTGC 177

B2_BF1_(PacBio_6sub1) TCACCCCATGTCCCTGCAGGCAGCATCTGATGGACACCTTCTGTCACCAACTGTCCCTGC 177

B2_BF1_(AM279336) 0

B2_BF1_(PacBio_7sub2) TCACCCCATGTCCCTGCAGGCAGCATCTGATGGACACCTTCTGTCACCAACTGTCCCTGC 177

B2_BF1_(AM279340) 0

B4_BF1_(AM279341) 0

B4_BF1_(PacBio_C) TCACC**T**CATGTCCCTGCAGGCAGCATCTGATGGACACCTTCTGTCACCAACTGTCCCTGC 177

B4_BF1_(AM279337) 0

B12_BF1_(AB426147) TCACCCCATGTCCCTGCAGGCAGCATCTGATGGACACCTTCTGTCACCAACTGTCCCTGC 177

B12_BF1(PacBio_C) TCACCCCATGTCCCTGCAGGCAGCATCTGATGGACACCTTCTGTCACCAACTGTCCCTGC 177

B12_BF1_(AL023516) TCACCCCATGTCCCTGCAGGCAGCATCTGATGGACACCTTCTGTCACCAACTGTCCCTGC 177

B14_BF1_(PacBio_WL) TCACC**A**CATGTCCCTGCAGGCAGCATCTGATGGACACCTTCTGTCACCAACTGTCCCTG**T** 177

B15_BF1_(AB426149) TCACCCCATGTCCCTGCAGGCAGCATCTGATGGACACCTTCTGTCACCAACTGTCC**T**TGC 177

B15_BF1_(PacBio_15I) TCACCCCATGTCCCTGCAGGCAGCATCTGATGGACACCTTCTGTCACCAACTGTCC**T**TGC 177

B19_BF1_(AB426151) TCACCCCATGTCCCTGCAGGCAGCATCTGATGGACACCTTCTGTCACCAACTGTCCCTGC 177

B19_BF1_(PacBio_P2a) TCACCCCATGTCCCTGCAGGCAGCATCTGATGGACACCTTCTGTCACCAACTGTCCCTGC 177

B19_BF1_(AM279338) 0

B21_BF1_(AB426152) TCACC**A**CATGTCCCTGCAGGCAGCATCTGATGGACACCTTCTGTCACCAACTGTCCCTG**T** 177

B21_BF1_(PacBio_N) TCACC**A**CATGTCCCTGCAGGCAGCATCTGATGGACACCTTCTGTCACCAACTGTCCCTG**T** 177

B21_BF1_(AM279339) 0

B21_BF1_(PacBio_0) TCACC**A**CATGTCCCTGCAGGCAGCATCTGATGGACACCTTCTGTCACCAACTGTCCCTG**T** 177

B21_BF1_(AM279342) 0

Fig. S1

**DMB2 PolyA site**

B2_BF1_(AB426141) GTGTCCCCATCCCTGACTCTGCGCCGTGGTGCTGACATTAAAGACACTCTGCAGCCTCTG 237

B2_BF1_(PacBio_6sub1) GTGTCCCCATCCCTGACTCTGCGCCGTGGTGCTGACATTAAAGACACTCTGCAGCCTCTG 237

B2_BF1_(AM279336) 0

B2_BF1_(PacBio_7sub2) GTGTCCCCATCCCTGACTCTGCGCCGTGGTGCTGACATTAAAGACACTCTGCAGCCTCTG 237

B2_BF1_(AM279340) 0

B4_BF1_(AM279341) 0

B4_BF1_(PacBio_C) GTGTCCCCATCCCTGAC**--**TGCGCCGTGGTGCTGACATTAAAGACACTCTGCAGCCTCTG 235

B4_BF1_(AM279337) 0

B12_BF1_(AB426147) GTGTCCCCATCCCTGACTCTGCGCCGTGGTGCTGACATTAAAGACACTCTGCAGCCTCTG 237

B12_BF1(PacBio_C) GTGTCCCCATCCCTGACTCTGCGCCGTGGTGCTGACATTAAAGACACTCTGCAGCCTCTG 237

B12_BF1_(AL023516) GTGTCCCCATCCCTGACTCTGCGCCGTGGTGCTGACATTAAAGACACTCTGCAGCCTCTG 237

B14_BF1_(PacBio_WL) GTGTCCCCATCCCTGACTCTGCGCCGTGGTGCTGACATTAAAGACACTCTGCAGCCTCTG 237

B15_BF1_(AB426149) GTGTCCCCATCCCTGACTCTGCGCCGTGGTGCTGACATTAAAGACACTCTGCAGCCTCTG 237

B15_BF1_(PacBio_15I) GTGTCCCCATCCCTGACTCTGCGCCGTGGTGCTGACATTAAAGACACTCTGCAGCCTCTG 237

B19_BF1_(AB426151) GTGTCCCCATCCCTGACTCTGCGCCGTGGTGCTGACATTAAAGACACTCTGCAGCCTCTG 237

B19_BF1_(PacBio_P2a) GTGTCCCCATCCCTGACTCTGCGCCGTGGTGCTGACATTAAAGACACTCTGCAGCCTCTG 237

B19_BF1_(AM279338) 0

B21_BF1_(AB426152) GTGTCCCCATCCCTGACTCTGC**A**CCGTGGTGCTGACATTAAAGACACTCTGCAGCCTCTG 237

B21_BF1_(PacBio_N) GTGTCCCCATCCCTGACTCTGC**A**CCGTGGTGCTGACATTAAAGACACTCTGCAGCCTCTG 237

B21_BF1_(AM279339) 0

B21_BF1_(PacBio_0) GTGTCCCCATCCCTGACTCTGC**A**CCGTGGTGCTGACATTAAAGACACTCTGCAGCCTCTG 237

B21_BF1_(AM279342) 0

B2_BF1_(AB426141) TTGGTGTCTCTGTGGGCTTTTGGGGTGGGGTGGTGTCACCGGGGAGAGGTTGGGTTGGGG 297

B2_BF1_(PacBio_6sub1) TTGGTGTCTCTGTGGGCTTTTGGGGTGGGGTGGTGTCACCGGGGAGAGGTTGGGTTGGGG 297

B2_BF1_(AM279336) 0

B2_BF1_(PacBio_7sub2) TTGGTGTCTCTGTGGGCTTTTGGGGTGGGGTGGTGTCACCGGGGAGAGGTTGGGTTGGGG 297

B2_BF1_(AM279340) 0

B4_BF1_(AM279341) 0

B4_BF1_(PacBio_C) TTGGTGTCTCTGTGGGCTTTTGGGGTGGGGTGGTGTCACCGGGGAGAGGTTGGGTTGGGG 295

B4_BF1_(AM279337) 0

B12_BF1_(AB426147) TTGGTGTCTCTGTGGGCTTTTGGGGTGGGGTGGTGTCACCGGGGAGAGGTTGGGTTGGGG 297

B12_BF1(PacBio_C) TTGGTGTCTCTGTGGGCTTTTGGGGTGGGGTGGTGTCACCGGGGAGAGGTTGGGTTGGGG 297

B12_BF1_(AL023516) TTGGTGTCTCTGTGGGCTTTTGGGGTGGGGTGGTGTCACCGGGGAGAGGTTGGGTTGGGG 297

B14_BF1_(PacBio_WL) TTGGTGTCTCTGTGGGCTTTTGGGGTGGGGTGGTGTCACCGGGGAGAGGTTGGGTTGGGG 297

B15_BF1_(AB426149) TTGGTGTCTCTGTGGGCTTTTGGGGTGGGGTGGTGTCACCGGGGAGAGGTTGGGTTGGGG 297

B15_BF1_(PacBio_15I) TTGGTGTCTCTGTGGGCTTTTGGGGTGGGGTGGTGTCACCGGGGAGAGGTTGGGTTGGGG 297

B19_BF1_(AB426151) TTGGTGTCTCTGTGGGCTTTTGGGGTGGGGTGGTGTCACCGGGGAGAGGTTGGGTTGGGG 297

B19_BF1_(PacBio_P2a) TTGGTGTCTCTGTGGGCTTTTGGGGTGGGGTGGTGTCACCGGGGAGAGGTTGGGTTGGGG 297

B19_BF1_(AM279338) 0

B21_BF1_(AB426152) TTGGTGTCTCTGTGGGCTTTTGGGGTGGGGTGGTGTCACCAGGGAGAGGTTGGGTTGGGG 297

B21_BF1_(PacBio_N) TTGGTGTCTCTGTGGGCTTTTGGGGTGGGGTGGTGTCACCAGGGAGAGGTTGGGTTGGGG 297

B21_BF1_(AM279339) 0

B21_BF1_(PacBio_0) TTGGTGTCTCTGTGGGCTTTTGGGGTGGGGTGGTGTCACCAGGGAGAGGTTGGGTTGGGG 297

B21_BF1_(AM279342) 0

B2_BF1_(AB426141) TCATTGC**G**TCCATGATGGTGATGGTGATTGACATTGTGCACAGGGAGATGTCCAGGCGCC 357

B2_BF1_(PacBio_6sub1) TCATTGC**G**TCCATGATGGTGATGGTGATTGACATTGTGCACAGGGAGATGTCCAGGCGCC 357

B2_BF1_(AM279336) 0

B2_BF1_(PacBio_7sub2) TCATTGC**G**TCCATGATGGTGATGGTGATTGACATTGTGCACAGGGAGATGTCCAGGCGCC 357

B2_BF1_(AM279340) 0

B4_BF1_(AM279341) 0

B4_BF1_(PacBio_C) TCATTGCATCCATGATGGTGATGGTGATTGACATTGTGCACAGGGAGATGTCCAGGCGCC 355

B4_BF1_(AM279337) 0

B12_BF1_(AB426147) TCATTGCATCCATGATGGTGATGGTGATTGACATTGTGCACAGGGAGATGTCCAGGCGCC 357

B12_BF1(PacBio_C) TCATTGCATCCATGATGGTGATGGTGATTGACATTGTGCACAGGGAGATGTCCAGGCGCC 357

B12_BF1_(AL023516) TCATTGCATCCATGATGGTGATGGTGATTGACATTGTGCACAGGGAGATGTCCAGGCGCC 357

B14_BF1_(PacBio_WL) TCATTGCATCCATGATGGTGATGGTGATTGACATTGTGCACAGGGAGATGTCCAGGCGCC 357

B15_BF1_(AB426149) TCATTGC**G**TCCATGATGGTGATGGTGATTGACATTGTGCACAGGGAGATGTCCAGGCGCC 357

B15_BF1_(PacBio_15I) TCATTGC**G**TCCATGATGGTGATGGTGATTGACATTGTGCACAGGGAGATGTCCAGGCGCC 357

B19_BF1_(AB426151) TCATTGCATCCATGATGGTGATGGTGATTGACATTGTGCACAGGGAGATGTCCAGGCGCC 357

B19_BF1_(PacBio_P2a) TCATTGCATCCATGATGGTGATGGTGATTGACATTGTGCACAGGGAGATGTCCAGGCGCC 357

B19_BF1_(AM279338) 0

B21_BF1_(AB426152) TCATTGCATCCATGATGGTGA**C**GGTGATTGACATTGTGCACAGGGAGATGTCCAGGCGCC 357

B21_BF1_(PacBio_N) TCATTGCATCCATGATGGTGA**C**GGTGATTGACATTGTGCACAGGGAGATGTCCAGGCGCC 357

B21_BF1_(AM279339) 0

B21_BF1_(PacBio_0) TCATTGCATCCATGATGGTGA**C**GGTGATTGACATTGTGCACAGGGAGATGTCCAGGCGCC 357

B21_BF1_(AM279342) 0

Fig. S1

B2_BF1_(AB426141) TGTGGGGTCTGTGTTTTAGGGCCAGTTCTGCTCAGTGCCTCCGTAAGTGATCTGGATAGG 417

B2_BF1_(PacBio_6sub1) TGTGGGGTCTGTGTTTTAGGGCCAGTTCTGCTCAGTGCCTCCGTAAGTGATCTGGATAGG 417

B2_BF1_(AM279336) 0

B2_BF1_(PacBio_7sub2) TGTGGGGTCTGTGTTTTAGGGCCAGTTCTGCTCAGTGCCTCCGTAAGTGATCTGGATAGG 417

B2_BF1_(AM279340) 0

B4_BF1_(AM279341) 0

B4_BF1_(PacBio_C) TGTGGGGTCTGTGTTTTAGGGCCAGTTCTGCTCAGTGCCTCCGTAAGTGATCTGGATAGG 415

B4_BF1_(AM279337) 0

B12_BF1_(AB426147) TGTGGGGTCTGTGTTTTAGGGCCAGTTCTGCTCAGTGCCTCCGTAAGTGATCTGGATAGG 417

B12_BF1(PacBio_C) TGTGGGGTCTGTGTTTTAGGGCCAGTTCTGCTCAGTGCCTCCGTAAGTGATCTGGATAGG 417

B12_BF1_(AL023516) TGTGGGGTCTGTGTTTTAGGGCCAGTTCTGCTCAGTGCCTCCGTAAGTGATCTGGATAGG 417

B14_BF1_(PacBio_WL) TGTGGGATCTGTGTTTTAGGGCCAGTTCTGCTCAGTGCCTCCGTAAGTGATCTGGAGAGG 417

B15_BF1_(AB426149) TGTGGGGTCTGTGTTTTAGGGCCAGTTCTGCTCAGTGCCTCCGTAAGTGATCTGGAGAGG 417

B15_BF1_(PacBio_15I) TGTGGGGTCTGTGTTTTAGGGCCAGTTCTGCTCAGTGCCTCCGTAAGTGATCTGGAGAGG 417

B19_BF1_(AB426151) TGTGGGGTCTGTGTTTTAGGGCCAGTTCTGCTCAGTGCCTCCGTAAGTGATCTGGATAGG 417

B19_BF1_(PacBio_P2a) TGTGGGGTCTGTGTTTTAGGGCCAGTTCTGCTCAGTGCCTCCGTAAGTGATCTGGATAGG 417

B19_BF1_(AM279338) 0

B21_BF1_(AB426152) TGTGGGGTCTGTGTTTTAGGGCCAGTTCTGCTCAGTGCCTCC**A**TAAGTGATCTGGATAGG 417

B21_BF1_(PacBio_N) TGTGGGGTCTGTGTTTTAGGGCCAGTTCTGCTCAGTGCCTCC**A**TAAGTGATCTGGATAGG 417

B21_BF1_(AM279339) 0

B21_BF1_(PacBio_0) TGTGGGGTCTGTGTTTTAGGGCCAGTTCTGCTCAGTGCCTCC**A**TAAGTGATCTGGATAGG 417

B21_BF1_(AM279342) 0

B2_BF1_(AB426141) TCGTCAGTCATCCTAATTAAGGAGGGGACAACAGTGAATGGGGAGGAGCCGATGACTCAG 477

B2_BF1_(PacBio_6sub1) TCGTCAGTCATCCTAATTAAGGAGGGGACAACAGTGAATGGGGAGGAGCCGATGACTCAG 477

B2_BF1_(AM279336) GGAGGGGACAACAGTGAATGGGGAGGAGCCGATGACTCAG 40

B2_BF1_(PacBio_7sub2) TCGTCAGTCATCCTAATTAAGGAGGGGACAACAGTGAATGGGGAGGAGCCGATGACTCAG 477

B2_BF1_(AM279340) GGAGGGGACAACAGTGAATGGGGAGGAGCCGATGACTCAG 40

B4_BF1_(AM279341) GGAGGGGACAACAGTGAATGGGGAGGAGCCGATGACTCAG 40

B4_BF1_(PacBio_C) TCGTCA**A**TCATCCTAATTAAGGAGGGGACAACAGTGAATGGGGAGGAGCCGATGACTCAG 475

B4_BF1_(AM279337) GGAGGGGACAACAGTGAATGGGGAGGAGCCGATGACTCAG 40

B12_BF1_(AB426147) TCGTCAGTCATCCTAATTAAGGAGGGGACAACAGTGAATGGGGAGGAGCCGATGACTCAG 477

B12_BF1(PacBio_C) TCGTCAGTCATCCTAATTAAGGAGGGGACAACAGTGAATGGGGAGGAGCCGATGACTCAG 477

B12_BF1_(AL023516) TCGTCAGTCATCCTAATTAAGGAGGGGACAACAGTGAATGGGGAGGAGCCGATGACTCAG 477

B14_BF1_(PacBio_WL) TCGTCAGTCATCCTAATTAAGGAGGGGACAACAGTGAATGGGGAGGAGCCGATGACT**G**AG 477

B15_BF1_(AB426149) TCGTCAGTCATCCTAATTAAGGAGGGGACAACAGTGAATGGGGAGGAGCCGATGACTCAG 477

B15_BF1_(PacBio_15I) TCGTCAGTCATCCTAATTAAGGAGGGGACAACAGTGAATGGGGAGGAGCCGATGACTCAG 477

B19_BF1_(AB426151) TCGTCAGTCATCCTAATTAAGGAGGGGACAACAGTGAATGGGGAGGAGCCGATGACTCAG 477

B19_BF1_(PacBio_P2a) TCGTCAGTCATCCTAATTAAGGAGGGGACAACAGTGAATGGGGAGGAGCCGATGACTCAG 477

B19_BF1_(AM279338) GGAGGGGACAACAGTGAATGGGGAGGAGCCGATGACTCAG 40

B21_BF1_(AB426152) TCAT**G**AGTCATCCTAATTAAGGAGGGGACAACAGTGAATGGGGAGGAGCCGATGACTCAG 477

B21_BF1_(PacBio_N) TCAT**G**AGTCATCCTAATTAAGGAGGGGACAACAGTGAATGGGGAGGAGCCGATGACTCAG 477

B21_BF1_(AM279339) GGAGGGGACAACAGTGAATGGGGAGGAGCCGATGACTCAG 40

B21_BF1_(PacBio_0) TCAT**G**AGTCATCCTAATTAAGGAGGGGACAACAGTGAATGGGGAGGAGCCGATGACTCAG 477

B21_BF1_(AM279342) GGAGGGGACAACAGTGAATGGGGAGGAGCCGATGACTCAG 40

B2_BF1_(AB426141) GCTGGGAGTGGTGATCCCAGAGGTTTCCTCTGCTGTCAGTGACTCCGTGCCTCTCCGAGT 537

B2_BF1_(PacBio_6sub1) GCTGGGAGTGGTGATCCCAGAGGTTTCCTCTGCTGTCAGTGACTCCGTGCCTCTCCGAGT 537

B2_BF1_(AM279336) GCTGGGAGTGGTGATCCCAGAGGTTTCCTCTGCTGTCAGTGACTCCGTGCCTCTCCGAGT 100

B2_BF1_(PacBio_7sub2) GCTGGGAGTGGTGATCCCAGAGGTTTCCTCTGCTGTCAGTGACTCCGTGCCTCTCCGAGT 537

B2_BF1_(AM279340) GCTGGGAGTGGTGATCCCAGAGGTTTCCTCTGCTGTCAGTGACTCCGTGCCTCTCCGAGT 100

B4_BF1_(AM279341) GCTGGGAGTGGTGATCCCAGAGGTTTCCTCTGCTGTCAGTGACTCCGTGCCTC**A**CCGAGT 100

B4_BF1_(PacBio_C) GCTGGGAGTGGTGATCCCAGAGGTTTCCTCTGCTGTCAGTGACTCCGTGCCTC**A**CCGAGT 535

B4_BF1_(AM279337) GCTGGGAGTGGTGATCCCAGAGGTTTCCTCTGCTGTCAGTGACTCCGTGCCTC**A**CCGAGT 100

B12_BF1_(AB426147) GCTGGGAGTGGTGATCCCAGAGGTTTCCTCTGCTGTCAGTGACTCCGT------------ 527

B12_BF1(PacBio_C) GCTGGGAGTGGTGATCCCAGAGGTTTCCTCTGCTGTCAGTGACTCCGT------------ 527

B12_BF1_(AL023516) GCTGGGAGTGGTGATCCCAGAGGTTTCCTCTGCTGTCAGTGACTCCGT------------ 527

B14_BF1_(PacBio_WL) GCTGGGAGTGGTGATCCCAGAGGTTTCCTCTGCTGTCAGTGACTCCGTGCCTCTCCGAGT 537

B15_BF1_(AB426149) GCTGGGAGTGGTGATCCCAGAGGTTTCCTCTGCTGTCAGTGACTCCGTGCCTCTCCGAGT 537

B15_BF1_(PacBio_15I) GCTGGGAGTGGTGATCCCAGAGGTTTCCTCTGCTGTCAGTGACTCCGTGCCTCTCCGAGT 537

B19_BF1_(AB426151) GCTGGGAGTGGTGATCCCAGAGGTTTCCTCTGCTGTCAGTGACTCCGT------------ 527

B19_BF1_(PacBio_P2a) GCTGGGAGTGGTGATCCCAGAGGTTTCCTCTGCTGTCAGTGACTCCGT------------ 527

B19_BF1_(AM279338) GCTGGGAGTGGTGATCCCAGAGGTTTCCTCTGCTGTCAGTGACTCCGT------------ 90

B21_BF1_(AB426152) GCTGGGAGTGGTGATCCCAGAGGTTTCCTCTGCTGTCAGTGACTCCGTGCCTC**A**CCGAGT 537

B21_BF1_(PacBio_N) GCTGGGAGTGGTGATCCCAGAGGTTTCCTCTGCTGTCAGTGACTCCGTGCCTC**A**CCGAGT 537

B21_BF1_(AM279339) GCTGGGAGTGGTGATCCCAGAGGTTTCCTCTGCTGTCAGTGACTCCGTGCCTC**A**CCGAGT 100

B21_BF1_(PacBio_0) GCTGGGAGTGGTGATCCCAGAGGTTTCCTCTGCTGTCAGTGACTCCGTGCCTC**A**CCGAGT 537

B21_BF1_(AM279342) GCTGGGAGTGGTGATCCCAGAGGTTTCCTCTGCTGTCAGTGACTCCGTGCCTC**A**CCGAGT 100

Fig. S1

B2_BF1_(AB426141) CGGACGGCTCCGTGCAGGAAGGAGCGCTCCGCGCACCCCACG**A**CC**G**CACCCGGG**C**GGGTT 597

B2_BF1_(PacBio_6sub1) CGGACGGCTCCGTGCAGGAAGGAGCGCTCCGCGCACCCCACG**A**CC**G**CACCCGGG**C**GGGTT 597

B2_BF1_(AM279336) CGGACGGCTCCGTGCAGGAAGGAGCGCTCCGCGCACCCCACG**A**CC**G**CACCCGGG**C**GGGTT 160

B2_BF1_(PacBio_7sub2) CGGACGGCTCCGTGCAGGAAGGAGCGCTCCGCGCACCCCACG**A**CC**G**CACCCGGG**C**GGGTT 597

B2_BF1_(AM279340) CGGACGGCTCCGTGCAGGAAGGAGCGCTCCGCGCACCCCACG**A**CC**G**CACCCGGG**C**GGGTT 160

B4_BF1_(AM279341) CGGACGGCTCCGTGCAGGAAGGAGCGCTCCGCGCACCCCACGCCCCCACCCGGGGGGGTT 160

B4_BF1_(PacBio_C) CGGACGGCTCCGTGCAGGAAGGAGCGCTCCGCGCACCCCACGCCCCCACCCGGGGGGGTT 595

B4_BF1_(AM279337) CGGACGGCTCCGTGCAGGAAGGAGCGCTCCGCGCACCCCACGCCCCCACCCGGGGGGGTT 160

B12_BF1_(AB426147) ------------------------------------------------------------ 527

B12_BF1(PacBio_C) ------------------------------------------------------------ 527

B12_BF1_(AL023516) ------------------------------------------------------------ 527

B14_BF1_(PacBio_WL) CGGACGGCTCCGTGCAGGAAGGAGCGCTCCGC**A**CACC**T**CACGCCCCCAC**T**CGGGGGGGTT 597

B15_BF1_(AB426149) CGGACGGCTCCGTGCAGGAAGGAGCGCTCCGC**A**CACC**T**CACGCCCCCAC**T**CGGGGGGGTT 597

B15_BF1_(PacBio_15I) CGGACGGCTCCGTGCAGGAAGGAGCGCTCCGC**A**CACC**T**CACGCCCCCAC**T**CGGGGGGGTT 597

B19_BF1_(AB426151) ------------------------------------------------------------ 527

B19_BF1_(PacBio_P2a) ------------------------------------------------------------ 527

B19_BF1_(AM279338) ------------------------------------------------------------ 90

B21_BF1_(AB426152) C**C**GACGGCTCCGTGCAGGAAGGAGCGCTCCGCGCACCCCACGCCCCCACCCGGGGGGGTT 597

B21_BF1_(PacBio_N) C**C**GACGGCTCCGTGCAGGAAGGAGCGCTCCGCGCACCCCACGCCCCCACCCGGGGGGGTT 597

B21_BF1_(AM279339) C**C**GACGGCTCCGTGCAGGAAGGAGCGCTCCGCGCACCCCACGCCCCCACCCGGGGGGGTT 160

B21_BF1_(PacBio_0) C**C**GACGGCTCCGTGCAGGAAGGAGCGCTCCGCGCACCCCACGCCCCCACCCGGGGGGGTT 597

B21_BF1_(AM279342) C**C**GACGGCTCCGTGCAGGAAGGAGCGCTCCGCGCACCCCACGCCCCCACCCGGGGGGGTT 160

B2_BF1_(AB426141) CAGCGCAGCCCCCCGGCCGTGCTCTGAC**G**-GACGACTGCTTGCCTTCCCATTGCCCCCCG 656

B2_BF1_(PacBio_6sub1) CAGCGCAGCCCCCCGGCCGTGCTCTGAC**G**-GACGACTGCTTGCCTTCCCATTGCCCCCCG 656

B2_BF1_(AM279336) CAGCGCAGCCCCCCGGCCGTGCTCTGAC**GG**GACGACTGCTTGCCTTCCCATTGCCCCCCG 220

B2_BF1_(PacBio_7sub2) CAGCGCAGCCCCCCGGCCGTGCTCTGAC**G**-GACGACTGCTTGCCTTCCCATTGCCCCCCG 656

B2_BF1_(AM279340) CAGCGCAGCCCCCCGGCCGTGCTCTGAC**GG**GACGACTGCTTGCCTTCCCATTGCCCCCCG 220

B4_BF1_(AM279341) CAGCGCAGCCCCCCGGCCGTGCTCTGACA-GACGACTGCTTGCCTTCCCATTGCCCCCCG 219

B4_BF1_(PacBio_C) CAGCGCAGCCCCCCGGCCGTGCTCTGACA-GACGACTGCTTGCCTTCCCATTGCCCCCCG 654

B4_BF1_(AM279337) CAGCGCAGCCCCCCGGCCGTGCTCTGACA-GACGACTGCTTGCCTTCCCATTGCCCCCCG 219

B12_BF1_(AB426147) ------------------------------------------------------------ 527

B12_BF1(PacBio_C) ------------------------------------------------------------ 527

B12_BF1_(AL023516) ------------------------------------------------------------ 527

B14_BF1_(PacBio_WL) CAGCGCAGCCCCCCG**A**CCGTGCTCTGACA-GAC**A**ACTGCTTGCCTTCCCATTGCCCCCCG 656

B15_BF1_(AB426149) CAGCGCAGCCCCCCG**A**CCGTGCTCTGACA-GAC**A**ACTGCTTGCCTTCCCATTGCCCCCCG 656

B15_BF1_(PacBio_15I) CAGCGCAGCCCCCCG**A**CCGTGCTCTGACA-GAC**A**ACTGCTTGCCTTCCCATTGCCCCCCG 656

B19_BF1_(AB426151) ------------------------------------------------------------ 527

B19_BF1_(PacBio_P2a) ------------------------------------------------------------ 527

B19_BF1_(AM279338) ------------------------------------------------------------ 90

B21_BF1_(AB426152) CAGCGCAGCCCCCCGGCCGTGCTCTGACA-GACGACTGCTTGCCTTCCCATTGCCCCCCG 656

B21_BF1_(PacBio_N) CAGCGCAGCCCCCCGGCCGTGCTCTGACA-GACGACTGCTTGCCTTCCCATTGCCCCCCG 656

B21_BF1_(AM279339) CAGCGCAGCCCCCCGGCCGTGCTCTGACA-GACGACTGCTTGCCTTCCCATTGCCCCCCG 219

B21_BF1_(PacBio_0) CAGCGCAGCCCCCCGGCCGTGCTCTGACA-GACGACTGCTTGCCTTCCCATTGCCCCCCG 656

B21_BF1_(AM279342) CAGCGCAGCCCCCCGGCCGTGCTCTGACA-GACGACTGCTTGCCTTCCCATTGCCCCCCG 219

B2_BF1_(AB426141) TCGCCCCGACAGAGGCCCTCGGCGCCCTCCCCTGGGTTAGGAACACGGCCCCGGGGGGGA 716

B2_BF1_(PacBio_6sub1) TCGCCCCGACAGAGGCCCTCGGCGCCCTCCCCTGGGTTAGGAACACGGCCCCGGGGGGGA 716

B2_BF1_(AM279336) TCGCCCCGACAGAGGCCCTCGGCGCCCTCCCCTGGGTTAGGAACACGGCCCCGGGGGGGA 280

B2_BF1_(PacBio_7sub2) TCGCCCCGACAGAGGCCCTCGGCGCCCTCCCCTGGGTTAGGAACACGGCCCCGGGGGGGA 716

B2_BF1_(AM279340) TCGCCCCGACAGAGGCCCTCGGCGCCCTCCCCTGGGTTAGGAACACGGCCCCGGGGGGGA 280

B4_BF1_(AM279341) TCGCCCCGACAGAGGCCCTCGGCGCCCTCCCCTGGGTTAGGAACACGGCCCCGGGGGGGA 279

B4_BF1_(PacBio_C) TCGCCCCGACAGAGGCCCTCGGCGCCCTCCCCTGGGTTAGGAACACGGCCCCGGGGGGGA 714

B4_BF1_(AM279337) TCGCCCCGACAGAGGCCCTCGGCGCCCTCCCCTGGGTTAGGAACACGGCCCCGGGGGGGA 279

B12_BF1_(AB426147) ------------------------------------------------------------ 527

B12_BF1(PacBio_C) ------------------------------------------------------------ 527

B12_BF1_(AL023516) ------------------------------------------------------------ 527

B14_BF1_(PacBio_WL) TCGCCCCGACAGAGGCCCTCGGCGCCCTCCCCTGGGTTAGGAACACGGCCCCGGGGGGGA 716

B15_BF1_(AB426149) TCGCCCCGACAGAGGCCCTCGGCGCCCTCCCCTGGGTTAGGAACACGGCCCCGGGGGGGA 716

B15_BF1_(PacBio_15I) TCGCCCCGACAGAGGCCCTCGGCGCCCTCCCCTGGGTTAGGAACACGGCCCCGGGGGGGA 716

B19_BF1_(AB426151) ------------------------------------------------------------ 527

B19_BF1_(PacBio_P2a) ------------------------------------------------------------ 527

B19_BF1_(AM279338) ------------------------------------------------------------ 90

B21_BF1_(AB426152) TCGCCCCGACAGAGGCCCTCGGCGCCCTCCCCTGGGTTAGGAACACGGCCCCGGGGGGGA 716

B21_BF1_(PacBio_N) TCGCCCCGACAGAGGCCCTCGGCGCCCTCCCCTGGGTTAGGAACACGGCCCCGGGGGGGA 716

B21_BF1_(AM279339) TCGCCCCGACAGAGGCCCTCGGCGCCCTCCCCTGGGTTAGGAACACGGCCCCGGGGGGGA 279

B21_BF1_(PacBio_0) TCGCCCCGACAGAGGCCCTCGGCGCCCTCCCCTGGGTTAGGAACACGGCCCCGGGGGGGA 716

B21_BF1_(AM279342) TCGCCCCGACAGAGGCCCTCGGCGCCCTCCCCTGGGTTAGGAACACGGCCCCGGGGGGGA 279

Fig. S1

**---enhancer A---**

B2_BF1_(AB426141) CACAGCCCGAGTGCCC**G**TTCGGTGTC**A**GGGAGGCAGGGAGGGGACCCCCACCGCGCCCGT 776

B2_BF1_(PacBio_6sub1) CACAGCCCGAGTGCCC**G**TTCGGTGTC**A**GGGAGGCAGGGAGGGGACCCCCACCGCGCCCGT 776

B2_BF1_(AM279336) CACAGCCCGAGTGCCC**G**TTCGGTGTC**A**GGGAGGCAGGGAGGGGACCCCCACCGCGCCCGT 340

B2_BF1_(PacBio_7sub2) CACAGCCCGAGTGCCC**G**TTCGGTGTC**A**GGGAGGCAGGGAGGGGACCCCCACCGCGCCCGT 776

B2_BF1_(AM279340) CACAGCCCGAGTGCCC**G**TTCGGTGTC**A**GGGAGGCAGGGAGGGGACCCCCACCGCGCCCGT 340

B4_BF1_(AM279341) CACAGCCCGAGTGCCCTTTCGGTGTCCGGGAGGCAGGGAGGGGACCCCCACCGCGCCCGT 339

B4_BF1_(PacBio_C) CACAGCCCGAGTGCCCTTTCGGTGTCCGGGAGGCAGGGAGGGGACCCCCACCGCGCCCGT 774

B4_BF1_(AM279337) CACAGCCCGAGTGCCCTTTCGGTGTCCGGGAGGCAGGGAGGGGACCCCCACCGCGCCCGT 339

B12_BF1_(AB426147) ------------------------------------------------------------ 527

B12_BF1(PacBio_C) ------------------------------------------------------------ 527

B12_BF1_(AL023516) ------------------------------------------------------------ 527

B14_BF1_(PacBio_WL) CACAGCCCGAGTGCCC**G**TTCGGTGTC**A**GGGAGGCAGGGAGGGGACC-------------- 762

B15_BF1_(AB426149) CACAGCCCGAGTGCCCTTTCGGTGTCCGGGAGGCAGGGAGGGGACCCCCACCGCGCCCGT 776

B15_BF1_(PacBio_15I) CACAGCCCGAGTGCCC**G**TTCGGTGTC**A**GGGAGGCAGGGAGGGGACC-------------- 762

B19_BF1_(AB426151) ------------------------------------------------------------ 527

B19_BF1_(PacBio_P2a) ------------------------------------------------------------ 527

B19_BF1_(AM279338) ------------------------------------------------------------ 90

B21_BF1_(AB426152) CACAGCCCGAGTGCCCTTTCGGTGTCCGGGAGGCAGGGAGGGGACCCCCACCGCGCCCGT 776

B21_BF1_(PacBio_N) CACAGCCCGAGTGCCCTTTCGGTGTCCGGGAGGCAGGGAGGGGACCCCCACCGCGCCCGT 776

B21_BF1_(AM279339) CACAGCCCGAGTGCCCTTTCGGTGTCCGGGAGGCAGGGAGGGGACCCCCACCGCGCCCGT 339

B21_BF1_(PacBio_0) CACAGCCCGAGTGCCCTTTCGGTGTCCGGGAGGCAGGGAGGGGACCCCCACCGCGCCCGT 776

B21_BF1_(AM279342) CACAGCCCGAGTGCCCTTTCGGTGTCCGGGAGGCAGGGAGGGGACCCCCACCGCGCCCGT 339

**----IRE---- --(s)-- ----S—**

B2_BF1_(AB426141) CCCC**--**CCC**G**GACTCCGCGCTTTCGCTTTCGCTTCACAA**T**CTGAGGGAGC 824

B2_BF1_(PacBio_6sub1) CCCC**--**CCC**G**GACTCCGCGCTTTCGCTTTCGCTTCACAA**T**CTGAGGGAGC 824

B2_BF1_(AM279336) CCCC**--**CCC**G**GACTCCGCGCTTTCGCTTTCGCTTCACAA**T**CTGAGGGAGC 388

B2_BF1_(PacBio_7sub2) CCCC**--**CCC**G**GACTCCGCGCTTTCGCTTTCGCTTCACAA**T**CTGAGGGAGC 824

B2_BF1_(AM279340) CCCC**--**CCC**G**GACTCCGCGCTTTCGCTTTCGCTTCACAA**T**CTGAGGGAGC 388

B4_BF1_(AM279341) CCCCGCCCCCGACTCCGCGCTTTCGCTTTCGCTTCACAACCTGAGGGAGC 389

B4_BF1_(PacBio_C) CCCCGCCCCCGACTCCGCGCTTTCGCTTTCGCTTCACAACCTGAGGGAGC 824

B4_BF1_(AM279337) CCCCGCCCCCGACTCCGCGCTTTCGCTTTCGCTTCACAACCTGAGGGAGC 389

B12_BF1_(AB426147) ------------------GCTTTCGCTTTCGCTTCACAACCTGAGGGAGC 557

B12_BF1(PacBio_C) ------------------GCTTTCGCTTTCGCTTCACAACCTGAGGGAGC 557

B12_BF1_(AL023516) ------------------GCTTTCGCTTTCGCTTCACAACCTGAGGGAGC 557

B14_BF1_(PacBio_WL) -------------------------------------------------- 762

B15_BF1_(AB426149) CCCCGCCCCCGACTCCGCGCTTTCGCTTTCGCTTCACAACCTGAGGGAGC 826

B15_BF1_(PacBio_15I) -------------------------------------------------- 762

B19_BF1_(AB426151) ------------------GCTTTCGCTTTCGCTTCACAACCTGAGGGAGC 557

B19_BF1_(PacBio_P2a) ------------------GCTTTCGCTTTCGCTTCACAACCTGAGGGAGC 557

B19_BF1_(AM279338) ------------------GCTTTCGCTTTCGCTTCACAACCTGAGGGAGC 120

B21_BF1_(AB426152) CCCCGCCCCCGACTCCGCGCTTTCGCTTTCGCTTCACAACCTGAGGGAGC 826

B21_BF1_(PacBio_N) CCCCGCCCCCGACTCCGCGCTTTCGCTTTCGCTTCACAACCTGAGGGAGC 826

B21_BF1_(AM279339) CCCCGCCCCCGACTCCGCGCTTTCGCTTTCGCTTCACAACCTGAGGGAGC 389

B21_BF1_(PacBio_0) CCCCGCCCCCGACTCCGCGCTTTCGCTTTCGCTTCACAACCTGAGGGAGC 826

B21_BF1_(AM279342) CCCCGCCCCCGACTCCGCGCTTTCGCTTTCGCTTCACAACCTGAGGGAGC 389

**-- --------X/X2--------- ---Y---**

B2_BF1_(AB426141) GCATTCTGCCTGGCGCCCGATGACGTCTCGCGCGCTCCCGGCCGCCATTGGCGGGGCGGC 884

B2_BF1_(PacBio_6sub1) GCATTCTGCCTGGCGCCCGATGACGTCTCGCGCGCTCCCGGCCGCCATTGGCGGGGCGGC 884

B2_BF1_(AM279336) GCATTCTGCCTGGCGCCCGATGACGTCTCGCGCGCTCCCGGCCGCCATTGGCGGGGCGGC 448

B2_BF1_(PacBio_7sub2) GCATTCTGCCTGGCGCCCGATGACGTCTCGCGCGCTCCCGGCCGCCATTGGCGGGGCGGC 884

B2_BF1_(AM279340) GCATTCTGCCTGGCGCCCGATGACGTCTCGCGCGCTCCCGGCCGCCATTGGCGGGGCGGC 448

B4_BF1_(AM279341) GCATTCTGCCTGGCGCCCGATGACGTCTCGCGCGCTCCCGG**T**CGCCATTGGCGGGGCGGC 449

B4_BF1_(PacBio_C) GCATTCTGCCTGGCGCCCGATGACGTCTCGCGCGCTCCCGG**T**CGCCATTGGCGGGGCGGC 884

B4_BF1_(AM279337) GCATTCTGCCTGGCGCCCGATGACGTCTCGCGCGCTCCCGG**T**CGCCATTGGCGGGGCGGC 449

B12_BF1_(AB426147) GCATTCTGCCTGGCGCCCGATGACGTC**A**C**ATAAA**C**C**CCCG**A**C**T**GCCATTGGCGG**A**G**A**GGC 617

B12_BF1(PacBio_C) GCATTCTGCCTGGCGC**-**CGATGACGTC**A**C**ATAAA**C**C**CCCG**A**C**T**GCCATTGGCGG**A**G**A**GGC 616

B12_BF1_(AL023516) GCATTCTGCCTGGCGCCCGATGACGTC**A**C**ATAAA**C**C**CCCG**A**C**T**GCCATTGGCGG**A**G**A**GGC 617

B14_BF1_(PacBio_WL) ------------------------------------------------------------ 762

B15_BF1_(AB426149) GCATTCTGCCTGGCGCCCGATGACGTCTCGCGCGCTCCCGGTCGCCATTGGCGGGGCGGC 886

B15_BF1_(PacBio_15I) ------------------------------------------------------------ 762

B19_BF1_(AB426151) GCATTCTGCCTGGCGCCCGATGACGTC**A**C**ATAAA**C**C**CCCG**A**C**T**GCCATTGGCGG**A**G**A**GGC 617

B19_BF1_(PacBio_P2a) GCATTCTGCCTGGCGC**-**CGATGACGTC**A**C**ATAAA**C**C**CCCG**A**C**T**GCCATTGGCGG**A**G**A**GGC 616

B19_BF1_(AM279338) GCATTCTGCCTGGCGCCCGATGACGTC**A**C**ATAAA**C**C**CCCG**A**C**T**GCCATTGGCGG**A**G**A**GGC 180

B21_BF1_(AB426152) GCATTCTGCCTGGCGCCCGATGACGTCTCGCGCGCTCCCGG**T**CGCCATTGGCGGGGCGGC 886

B21_BF1_(PacBio_N) GCATTCTGCCTGGCGCCCGATGACGTCTCGCGCGCTCCCGG**T**CGCCATTGGCGGGGCGGC 886

B21_BF1_(AM279339) GCATTCTGCCTGGCGCCCGATGACGTCTCGCGCGCTCCCGG**T**CGCCATTGGCGGGGCGGC 449

B21_BF1_(PacBio_0) GCATTCTGCCTGGCGCCCGATGACGTCTCGCGCGCTCCCGG**T**CGCCATTGGCGGGGCGGC 886

B21_BF1_(AM279342) GCATTCTGCCTGGCGCCCGATGACGTCTCGCGCGCTCCCGG**T**CGCCATTGGCGGGGCGGC 449

Fig. S1

**mRNA start (BF2)🡪**

B2_BF1_(AB426141) AA**G**GGAGGAACCAATGGGGGCGCGGTGCGGGACGGGG**G**CTGG----**A**CCCAGAACG**A**G-- 937

B2_BF1_(PacBio_6sub1) AA**G**GGAGGAACCAATGGGGGCGCGGTGCGGGACGGGG**G**CTGG----**A**CCCAGAACG**A**G-- 937

B2_BF1_(AM279336) AA**G**GGAGGAACCAATGGGGGCGCGGTGCGGGACGGGG**G**CTGG----**A**CCCAGAACG**A**G-- 501

B2_BF1_(PacBio_7sub2) AA**G**GGAGGAACCAATGGGGGCGCGGTGCGGGACGGGG**G**CTGG----**A**CCCAGAACG**A**G-- 937

B2_BF1_(AM279340) AA**G**GGAGGAACCAATGGGGGCGCGGTGCGGGACGGGG**G**CTGG----**A**CCCAGAACG**A**G-- 501

B4_BF1_(AM279341) AACGGAGGAACCAATGGGGGCGCGGTGCGGGACGGGGACTGG----TCCCAGAACGTG-- 502

B4_BF1_(PacBio_C) AACGGAGGAACCAATGGGGGCGCGGTGCGGGACGGGGACTGG----TCCCAGAACGTG-- 937

B4_BF1_(AM279337) AACGGAGGAACCAATGGGGGCGCGGTGCGGGACGGGGACTGG----TCCCAGAACGTG-- 502

B12_BF1_(AB426147) **G**ACGGAGGA**G**CCAATGGGGGCGCGG**G**GCGGG**G**CGG**A**G**GAGTAGGAAAAG**C**T**GAA**G**G**A**G**CT** 677

B12_BF1(PacBio_C) **G**ACGGAGGA**G**CCAATGGGGGCGCGG**G**GCGGG**G-**GG**A**G**GAGTAGGAAAAG**C**T**GAA**G**G**A**G**CT** 675

B12_BF1_(AL023516) **G**ACGGAGGA**G**CCAATGGGGGCGCGG**G**GCGGG**G**CGG**A**G**GAGTAGGAAAAG**C**T**GAA**G**G**A**G**CT** 677

B14_BF1_(PacBio_WL) ------------------------------------------------------------ 762

B15_BF1_(AB426149) AACGGAGGAACCAATGGGGGCGCGGTGCGGGACGGGGACTGG----TCCCAGAACGTG-- 939

B15_BF1_(PacBio_15I) ------------------------------------------------------------ 762

B19_BF1_(AB426151) **G**ACGGAGGA**G**CCAATGGGGGCGCGG**G**GCGGG**G**CGG**A**G**GAGTAGGAAAAG**C**T**GAA**G**G**A**G**CT** 677

B19_BF1_(PacBio_P2a) **G**ACGGAGGA**G**CCAATGGGGGCGCGG**G**GCGGG**G**-GG**A**G**GAGTAGGAAAAG**C**T**GAA**G**G**A**G**CT** 675

B19_BF1_(AM279338) **G**ACGGAGGA**G**CCAATGGGGGCGCGG**G**GCGGG**G**CGG**A**G**GAGTAGGAAAAG**C**T**GAA**G**G**A**G**CT** 240

B21_BF1_(AB426152) AACGGAGGAACCAATGGGGGCGCGGTGCGGGACGGGGACTGG----TCCCAGAACGTG-- 939

B21_BF1_(PacBio_N) AACGGAGGAACCAATGGGGGCGCGGTGCGGGACGGGGACTGG----TCCCAGAACGTG-- 939

B21_BF1_(AM279339) AACGGAGGAACCAATGGGGGCGCGGTGCGGGACGGGGACTGG----TCCCAGAACGTG-- 502

B21_BF1_(PacBio_0) AACGGAGGAACCAATGGGGGCGCGGTGCGGGACGGGGACTGG----TCCCAGAACGTG-- 939

B21_BF1_(AM279342) AACGGAGGAACCAATGGGGGCGCGGTGCGGGACGGGGACTGG----TCCCAGAACGTG-- 502

**mRNA start (BF2)🡪** **BF1 start**

B2_BF1_(AB426141) ----GAGGAGCGGTG---------------CGGTGCGAGGCGATGCGCCCGTGCGGGGCG 979

B2_BF1_(PacBio_6sub1) ----GAGGAGCGGTG---------------CGGTGCGAGGCGATGCGCCCGTGCGGGGCG 979

B2_BF1_(AM279336) ----GAGGAGCGGTG---------------CGGTGCGAGGCGATGCGCCCGTGCGGGGCG 543

B2_BF1_(PacBio_7sub2) ----GAGGAGCGGTG---------------CGGTGCGAGGCGATGCGCCCGTGCGGGGCG 979

B2_BF1_(AM279340) ----GAGGAGCGGTG---------------CGGTGCGAGGCGATGCGCCCGTGCGGGGCG 543

B4_BF1_(AM279341) ----GAGGAGCGGTG---------------CGGTGCG**G**GGCGATGCGCCCGTGCGGGGCG 544

B4_BF1_(PacBio_C) ----GAGGAGCGGTG---------------CGGTGCG**G**GGCGATGCGCCCGTGCGGGGCG 979

B4_BF1_(AM279337) ----GAGGAGCGGTG---------------CGGTGCG**G**GGCGATGCGCCCGTGCGGGGCG 544

B12_BF1_(AB426147) **GCGCTG**GG**T**GCGG**C**G**GACTTGAGAGTGCAG**CGGTG**T**GAGGCGATGGGGCCGTGCGGGGCG 737

B12_BF1(PacBio_C) **GCC-TG**GG**T**GCGG**C**G**GACTTGAGAGTGCAG**CGGTG**T**GAGGCGATGGGGCCGTGCGGGGCG 734

B12_BF1_(AL023516) **GCGCTG**GG**T**GCGG**C**G**GACTTGAGAGTGCAG**CGGTG**T**GAGGCGATGGGGCCGTGCGGGGCG 737

B14_BF1_(PacBio_WL) ------------------------------------------------------------ 762

B15_BF1_(AB426149) ----GAGGAGCGGTG---------------CGGTGCGGGGCGATGCGCCCGTGCGGGGCG 981

B15_BF1_(PacBio_15I) ------------------------------------------------------------ 762

B19_BF1_(AB426151) **GCGCTG**GG**T**GCGG**C**G**GACTTGAGAGTGCAG**CGGTG**T**GAGGCGATGGGGCCGTGCGGGGCG 737

B19_BF1_(PacBio_P2a) **GCC-TG**GG**T**GCGG**C**G**GACTTGAGAGTGCAG**CGGTG**T**GAGGCGATGGGGCCGTGCGGGGCG 734

B19_BF1_(AM279338) **GCGCTG**GG**T**GCGG**C**G**GACTTGAGAGTGCAG**CGGTG**T**GAGGCGATGGGGCCGTGCGGGGCG 300

B21_BF1_(AB426152) ----GAGGAGCGGTG---------------CGGTGCG**G**GGCGATGCGCCCGTGCGGGGCG 981

B21_BF1_(PacBio_N) ----GAGGAGCGGTG---------------CGGTGCG**G**GGCGATGCGCCCGTGCGGGGCG 981

B21_BF1_(AM279339) ----GAGGAGCGGTG---------------CGGTGCG**G**GGCGATGCGCCCGTGCGGGGCG 544

B21_BF1_(PacBio_0) ----GAGGAGCGGTG---------------CGGTGCG**G**GGCGATGCGCCCGTGCGGGGCG 981

B21_BF1_(AM279342) ----GAGGAGCGGTG---------------CGGTGCG**G**GGCGATGCGCCCGTGCGGGGCG 544

B2_BF1_(AB426141) **C**TGGGCCTG---------------GGGCTGCTGCTCGCCGCCGTGTGCGGGGCGGCGGCC 1024

B2_BF1_(PacBio_6sub1) **C**TGGGCCTG**---------------**GGGCTGCTGCTCGCCGCCGTGTGCGGGGCGGCGGCC 1024

B2_BF1_(AM279336) **C**TGGGCCTG**---------------**GGGCTGCTGCTCGCCGCCGTGTGCGGGGCGGCGGCC 588

B2_BF1_(PacBio_7sub2) **C**TGGGCCTG**---------------**GGGCTGCTGCTCGCCGCCGTGTGCGGGGCGGCGGCC 1024

B2_BF1_(AM279340) **C**TGGGCCTG**---------------**GGGCTGCTGCTCGCCGCCGTGTGCGGGGCGGCGGCC 588

B4_BF1_(AM279341) GTGGGCCTGGGGCTGCTGCGCCTGGGGCTGCTGCTCGCCGCCGTGTGCGGGGCGGCGGCC 604

B4_BF1_(PacBio_C) GTGGGCCTGGGGCTGCTGCGCCTGGGGCTGCTGCTCGCCGCCGTGTGCGGGGCGGCGGCC 1039

B4_BF1_(AM279337) GTGGGCCTGGGGCTGCTGCGCCTGGGGCTGCTGCTCGCCGCCGTGTGCGGGGCGGCGGCC 604

B12_BF1_(AB426147) **C**TGGGCCTG**---------------**GGGCTGCTGCTCGCCGCCGTGTGCGGGGCGGCGGCC 782

B12_BF1(PacBio_C) **C**TGGGCCTG**---------------**GGGCTGCTGCTCGCCGCCGTGTGCGGGGCGGCGGCC 779

B12_BF1_(AL023516) **C**TGGGCCTG**---------------**GGGCTGCTGCTCGCCGCCGTGTGCGGGGCGGCGGCC 782

B14_BF1_(PacBio_WL) ------------------------------------------------------------ 762

B15_BF1_(AB426149) GTGGGCCTGGGGCTGCTGCGCCTGGGGCTGCTGCTCGCCGCCGTGTGCGGGGCGGCGGCC 1041

B15_BF1_(PacBio_15I) ------------------------------------------------------------ 762

B19_BF1_(AB426151) **C**TGGGCCTG**---------------**GGGCTGCTGCTCGCCGCCGTGTGCGGGGCGGCGGCC 782

B19_BF1_(PacBio_P2a) **C**TGGGCCTG**---------------**GGGCTGCTGCTCGCCGCCGTGTGCGGGGCGGCGGCC 779

B19_BF1_(AM279338) **C**TGGGCCTG**---------------**GGGCTGCTGCTCGCCGCCGTGTGCGGGGCGGCGGCC 345

B21_BF1_(AB426152) GTGGGCCTGGGGCTGCTGCGCCTGGGGCTGCTGCTCGCCGCCGTGTGCGGGGCGGCGGCC 1041

B21_BF1_(PacBio_N) GTGGGCCTGGGGCTGCTGCGCCTGGGGCTGCTGCTCGCCGCCGTGTGCGGGGCGGCGGCC 1041

B21_BF1_(AM279339) GTGGGCCTGGGGCTGCTGCGCCTGGGGCTGCTGCTCGCCGCCGTGTGCGGGGCGGCGGCC 604

B21_BF1_(PacBio_0) GTGGGCCTGGGGCTGCTGCGCCTGGGGCTGCTGCTCGCCGCCGTGTGCGGGGCGGCGGCC 1041

B21_BF1_(AM279342) GTGGGCCTGGGGCTGCTGCGCCTGGGGCTGCTGCTCGCCGCCGTGTGCGGGGCGGCGGCC 604

Fig. S1

B2_BF1_(AB426141) GGTGAGTGCGGCCGGACCGGGACCCCTCCCCGCCCGTAACCCCACCCCGGGGCTGTGCCC 1084

B2_BF1_(PacBio_6sub1) GGTGAGTGCGGCCGGACCGGGACCCCTCCCCGCCCGTAACCCCACCCCGGGGCTGTGCCC 1084

B2_BF1_(AM279336) GGTGAGTGCGGCCGGACCGGGACCCCTCCCCGCCCGTAACCCCACCCCGGGGCTGTGCCC 648

B2_BF1_(PacBio_7sub2) GGTGAGTGCGGCCGGACCGGGACCCCTCCCCGCCCGTAACCCCACCCCGGGGCTGTGCCC 1084

B2_BF1_(AM279340) GGTGAGTGCGGCCGGACCGGGACCCCTCCCCGCCCGTAACCCCACCCCGGGGCTGTGCCC 648

B4_BF1_(AM279341) GGTGAGTGCGGCCGGACCGGGACCCCTCCCCGCCCGTAACCCCACCCCGGGGCTGTGCCC 664

B4_BF1_(PacBio_C) GGTGAGTGCGGCCGGACCGGGACCCCTCCCCGCCCGTAACCCCACCCCGGGGCTGTGCCC 1099

B4_BF1_(AM279337) GGTGAGTGCGGCCGGACCGGGACCCCTCCCCGCCCGTAACCCCACCCCGGGGCTGTGCCC 664

B12_BF1_(AB426147) GGTGAGTGCGGCCGGACCGGGACCCCTCCCCGCCCGTAACCCCACCCCGGGGCTGTGCCC 842

B12_BF1(PacBio_C) GGTGAGTGCGGCCGGACCGGGACCCCTCCCCGCCCGTAACCCCACCCCGGGGCTGTGCCC 839

B12_BF1_(AL023516) GGTGAGTGCGGCCGGACCGGGACCCCTCCCCGCCCGTAACCCCACCCCGGGGCTGTGCCC 842

B14_BF1_(PacBio_WL) ------------------------------------------------------------ 762

B15_BF1_(AB426149) GGTGAGTGCGGCCGGACCGGGACCCCTCCCCGCCCGTAACCCCACCCCGGGGCTGTGCCC 1101

B15_BF1_(PacBio_15I) ------------------------------------------------------------ 762

B19_BF1_(AB426151) GGTGAGTGCGGCCGGACCGGGACCCCTCCCCGCCCGTAACCCCACCCCGGGGCTGTGCCC 842

B19_BF1_(PacBio_P2a) GGTGAGTGCGGCCGGACCGGGACCCCTCCCCGCCCGTAACCCCACCCCGGGGCTGTGCCC 839

B19_BF1_(AM279338) GGTGAGTGCGGCCGGACCGGGACCCCTCCCCGCCCGTAACCCCACCCCGGGGCTGTGCCC 405

B21_BF1_(AB426152) GGTGAGTGCGGCCGGACCGGGACCCCTCCCCGCCCGTAACCCCACCCCGGGGCTGTGCCC 1101

B21_BF1_(PacBio_N) GGTGAGTGCGGCCGGACCGGGACCCCTCCCCGCCCGTAACCCCACCCCGGGGCTGTGCCC 1101

B21_BF1_(AM279339) GGTGAGTGCGGCCGGACCGGGACCCCTCCCCGCCCGTAACCCCACCCCGGGGCTGTGCCC 664

B21_BF1_(PacBio_0) GGTGAGTGCGGCCGGACCGGGACCCCTCCCCGCCCGTAACCCCACCCCGGGGCTGTGCCC 1101

B21_BF1_(AM279342) GGTGAGTGCGGCCGGACCGGGACCCCTCCCCGCCCGTAACCCCACCCCGGGGCTGTGCCC 664

**BF1 exon 2**

B2_BF1_(AB426141) GTGGGATCCTCAGACCC**A**CACCCGCGGCTCACGGCCCCGCTGCGCTCCG**T**CCCCGCAGAG 1144

B2_BF1_(PacBio_6sub1) GTGGGATCCTCAGACCC**A**CACCCGCGGCTCACGGCCCCGCTGCGCTCCG**T**CCCCGCAGAG 1144

B2_BF1_(AM279336) GTGGGATCCTCAGACCC**A**CACCCGCGGCTCACGGCCCCGCTGCGCTCCG**T**CCCCGCAGAG 708

B2_BF1_(PacBio_7sub2) GTGGGATCCTCAGACCC**A**CACCCGCGGCTCACGGCCCCGCTGCGCTCCG**T**CCCCGCAGAG 1144

B2_BF1 _(AM279340) GTGGGATCCTCAGACCC**A**CACCCGCGGCTCACGGCCCCGCTGCGCTCCG**T**CCCCGCAGAG 708

B4_BF1_(AM279341) GTGGGATCCTCAGACCCCCACCCGCGGCTCACGGCCCCGCTGCGCTCCGCCCCCGCAGAG 724

B4_BF1_(PacBio_C) GTGGGATCCTCAGACCCCCACCCGCGGCTCACGGCCCCGCTGCGCTCCGCCCCCGCAGAG 1159

B4_BF1_(AM279337) GTGGGATCCTCAGACCCCCACCCGCGGCTCACGGCCCCGCTGCGCTCCGCCCCCGCAGAG 724

B12_BF1_(AB426147) GTGGGATCCTCAGACCCCCACCCGCGGCTCACGGCC**T**CGCTGCGCTCCGCCCCCGCAGAG 902

B12_BF1(PacBio_C) GTGGGATCCTCAGACCCCCACCCGCGGCTCACGGCC**T**CGCTGCGCTCCGCCCCCGCAGAG 899

B12_BF1_(AL023516) GTGGGATCCTCAGACCCCCACCCGCGGCTCACGGCC**T**CGCTGCGCTCCGCCCCCGCAGAG 902

B14_BF1_(PacBio_WL) ------------------------------------------------------------ 762

B15_BF1_(AB426149) GTGGGATCCTCAGACCCCCACCCGCGGCTCACGGCCCCGCTGCGCTCCGCCCCCGCAGAG 1161

B15_BF1_(PacBio_15I) ------------------------------------------------------------ 762

B19_BF1_(AB426151) GTGGGATCCTCAGACCCCCACCCGCGGCTCACGGCCCCGCTGCGCTCCGCCCCCGCAGAG 902

B19_BF1_(PacBio_P2a) GTGGGATCCTCAGACCCCCACCCGCGGCTCACGGCCCCGCTGCGCTCCGCCCCCGCAGAG 899

B19_BF1_(AM279338) GTGGGATCCTCAGACCCCCACCCGCGGCTCACGGCCCCGCTGCGCTCCGCCCCCGCAGAG 465

B21_BF1_(AB426152) GTGGGATCCTCAGACCCCCACCCGCGGCTCACGGCCCCGCTGCGCTCCGCCCCCGCAGAG 1161

B21_BF1_(PacBio_N) GTGGGATCCTCAGACCCCCACCCGCGGCTCACGGCCCCGCTGCGCTCCGCCCCCGCAGAG 1161

B21_BF1_(AM279339) GTGGGATCCTCAGACCCCCACCCGCGGCTCACGGCCCCGCTGCGCTCCGCCCCCGCAGAG 724

B21_BF1_(PacBio_0) GTGGGATCCTCAGACCCCCACCCGCGGCTCACGGCCCCGCTGCGCTCCGCCCCCGCAGAG 1161

B21_BF1_(AM279342) GTGGGATCCTCAGACCCCCACCCGCGGCTCACGGCCCCGCTGCGCTCCGCCCCCGCAGAG 724

B2_BF1_(AB426141) CTCCATACCCTGCGGTACATC**TC**TACGGCGATGACGGATCCCGGCCCCGGGCAGCCGTGG 1204

B2_BF1_(PacBio_6sub1) CTCCATACCCTGCGGTACATC**TC**TACGGCGATGACGGATCCCGGCCCCGGGCAGCCGTGG 1204

B2_BF1_(AM279336) CTCCATACCCTGCGGTACATC**TC**TACGGCGATGACGGATCCCGGCCCCGGGCAGCCGTGG 768

B2_BF1_(PacBio_7sub2) CTCCATACCCTGCGGTACATC**TC**TACGGCGATGACGGATCCCGGCCCCGGGCAGCCGTGG 1204

B2_BF1_(AM279340) CTCCATACCCTGCGGTACATC**TC**TACGGCGATGACGGATCCCGGCCCCGGGCAGCCGTGG 768

B4_BF1_(AM279341) CTCCATACCCTGCGGTACATCCATACGGCGATGACGGATCCCGGCCCCGGGCAGCCGTGG 784

B4_BF1_(PacBio_C) CTCCATACCCTGCGGTACATCCATACGGCGATGACGGATCCCGGCCCCGGGCAGCCGTGG 1219

B4_BF1_(AM279337) CTCCATACCCTGCGGTACATCCATACGGCGATGACGGATCCCGGCCCCGGGCAGCCGTGG 784

B12_BF1_(AB426147) CTCCAT**T**CCCTGCGGTAC**G**TCCATACGGCGATGACGGATCCCGGCCCCGGGC**T**GCCGTGG 962

B12_BF1(PacBio_C) CTCCAT**T**CCCTGCGGTAC**G**TCCATACGGCGATGACGGATCCCGGCCCCGGGC**T**GCCGTGG 959

B12_BF1_(AL023516) CTCCAT**T**CCCTGCGGTAC**G**TCCATACGGCGATGACGGATCCCGGCCCCGGGC**T**GCCGTGG 962

B14_BF1_(PacBio_WL) ------------------------------------------------------------ 762

B15_BF1_(AB426149) CTCCATACCCTGCGGTACATCCATACGGCGATGACGGATCCCGGCCCCGGGCAGCCGTGG 1221

B15_BF1_(PacBio_15I) ------------------------------------------------------------ 762

B19_BF1_(AB426151) CTCCAT**T**CCCTGCGGTAC**G**TCCATACGGCGATGACGGATCCCGGCCCCGGGC**T**GCCGTGG 962

B19_BF1_(PacBio_P2a) CTCCAT**T**CCCTGCGGTAC**G**TCCATACGGCGATGACGGATCCCGGCCCCGGGC**T**GCCGTGG 959

B19_BF1_(AM279338) CTCCAT**T**CCCTGCGGTAC**G**TCCATACGGCGATGACGGATCCCGGCCCCGGGC**T**GCCGTGG 525

B21_BF1_(AB426152) CTCCATACCCTGCGGTACATCCATACGGCGATGACGGATCCCGGCCCCGGGCAGCCGTGG 1221

B21_BF1_(PacBio_N) CTCCATACCCTGCGGTACATCCATACGGCGATGACGGATCCCGGCCCCGGGCAGCCGTGG 1221

B21_BF1_(AM279339) CTCCATACCCTGCGGTACATCCATACGGCGATGACGGATCCCGGCCCCGGGCAGCCGTGG 784

B21_BF1_(PacBio_0) CTCCATACCCTGCGGTACATCCATACGGCGATGACGGATCCCGGCCCCGGGCAGCCGTGG 1221

B21_BF1_(AM279342) CTCCATACCCTGCGGTACATCCATACGGCGATGACGGATCCCGGCCCCGGGCAGCCGTGG 784

Fig. S1

B2_BF1_(AB426141) TACGTGGACGTGGGGTA**C**GTGGACGGGGAACTCTTC**AC**GCACTACAACAGCACCGC**T**CGG 1264

B2_BF1_(PacBio_6sub1) TACGTGGACGTGGGGTA**C**GTGGACGGGGAACTCTTC**AC**GCACTACAACAGCACCGC**T**CGG 1264

B2_BF1_(AM279336) TACGTGGACGTGGGGTA**C**GTGGACGGGGAACTCTTC**AC**GCACTACAACAGCACCGC**T**CGG 828

B2_BF1_(PacBio_7sub2) TACGTGGACGTGGGGTA**C**GTGGACGGGGAACTCTTC**AC**GCACTACAACAGCACCGC**T**CGG 1264

B2_BF1_(AM279340) TACGTGGACGTGGGGTA**C**GTGGACGGGGAACTCTTC**AC**GCACTACAACAGCACCGC**T**CGG 828

B4_BF1_(AM279341) TACGTGGACGTGGGGTATGTGGACGGGGAACTCTTCGTGCACTACAACAGCACCGCGCGG 844

B4_BF1_(PacBio_C) TACGTGGACGTGGGGTATGTGGACGGGGAACTCTTCGTGCACTACAACAGCACCGCGCGG 1279

B4_BF1_(AM279337) TACGTGGACGTGGGGTATGTGGACGGGGAACTCTTCGTGCACTACAACAGCACCGCGCGG 844

B12_BF1_(AB426147) T**T**CGTGGACGTGGGGTA**C**GTGGACGGGGAACTCTTCGTGCACTACAACAGCACCGCGCGG 1022

B12_BF1(PacBio_C) T**T**CGTGGACGTGGGGTA**C**GTGGACGGGGAACTCTTCGTGCACTACAACAGCACCGCGCGG 1019

B12_BF1_(AL023516) T**T**CGTGGACGTGGGGTA**C**GTGGACGGGGAACTCTTCGTGCACTACAACAGCACCGCGCGG 1022

B14_BF1_(PacBio_WL) ------------------------------------------------------------ 762

B15_BF1_(AB426149) TACGTGGACGTGGGGTATGTGGACGGGGAACTCTTCGTGCACTACAACAGCACCGCGCGG 1281

B15_BF1_(PacBio_15I) ------------------------------------------------------------ 762

B19_BF1_(AB426151) T**T**CGTGGACGTGGGGTA**C**GTGGACGGGGAACTCTTCGTGCACTACAACAGCACCGCGCGG 1022

B19_BF1_(PacBio_P2a) T**T**CGTGGACGTGGGGTA**C**GTGGACGGGGAACTCTTCGTGCACTACAACAGCACCGCGCGG 1019

B19_BF1_(AM279338) T**T**CGTGGACGTGGGGTA**C**GTGGACGGGGAACTCTTCGTGCACTACAACAGCACCGCGCGG 585

B21_BF1_(AB426152) TACGTGGACGTGGGGTATGTGGACGGGGAACTCTTCGTGCACTACAACAGCACCGCGCGG 1281

B21_BF1_(PacBio_N) TACGTGGACGTGGGGTATGTGGACGGGGAACTCTTCGTGCACTACAACAGCACCGCGCGG 1281

B21_BF1_(AM279339) TACGTGGACGTGGGGTATGTGGACGGGGAACTCTTCGTGCACTACAACAGCACCGCGCGG 844

B21_BF1_(PacBio_0) TACGTGGACGTGGGGTATGTGGACGGGGAACTCTTCGTGCACTACAACAGCACCGCGCGG 1281

B21_BF1_(AM279342) TACGTGGACGTGGGGTATGTGGACGGGGAACTCTTCGTGCACTACAACAGCACCGCGCGG 844

B2_BF1_(AB426141) AGG**GCT**GTGCCCCGCACCGAGTGGAT**A**GCGGCCAA**CA**CGGACCAGCAGTACTGGGA**CA**GA 1324

B2_BF1_(PacBio_6sub1) AGG**GCT**GTGCCCCGCACCGAGTGGAT**A**GCGGCCAA**CA**CGGACCAGCAGTACTGGGA**CA**GA 1324

B2_BF1_(AM279336) AGG**GCT**GTGCCCCGCACCGAGTGGAT**A**GCGGCCAA**CA**CGGACCAGCAGTACTGGGA**CA**GA 888

B2_BF1_(PacBio_7sub2) AGG**GCT**GTGCCCCGCACCGAGTGGAT**A**GCGGCCAA**CA**CGGACCAGCAGTACTGGGA**CA**GA 1324

B2_BF1_(AM279340) AGG**GCT**GTGCCCCGCACCGAGTGGAT**A**GCGGCCAA**CA**CGGACCAGCAGTACTGGGA**CA**GA 888

B4_BF1_(AM279341) AGGTACGTGCCCCGCACCGAGTGGATGGCGGCCAAGGCGGACCAGCAGTACTGGGATGGA 904

B4_BF1_(PacBio_C) AGGTACGTGCCCCGCACCGAGTGGATGGCGGCCAAGGCGGACCAGCAGTACTGGGATGGA 1339

B4_BF1_(AM279337) AGGTACGTGCCCCGCACCGAGTGGATGGCGGCCAAGGCGGACCAGCAGTACTGGGATGGA 904

B12_BF1_(AB426147) AGGTACGTGCCCCGCACCGAGTGGATGGCGGCCAA**CA**CGGACCAGCAGTACTGGGATGGA 1082

B12_BF1(PacBio_C) AGGTACGTGCCCCGCACCGAGTGGATGGCGGCCAA**CA**CGGACCAGCAGTACTGGGATGGA 1079

B12_BF1_(AL023516) AGGTACGTGCCCCGCACCGAGTGGATGGCGGCCAA**CA**CGGACCAGCAGTACTGGGATGGA 1082

B14_BF1_(PacBio_WL) ------------------------------------------------------------ 762

B15_BF1_(AB426149) AGGTACGTGCCCCGCACCGAGTGGATGGCGGCCAAGGCGGACCAGCAGTACTGGGATGGA 1341

B15_BF1_(PacBio_15I) ------------------------------------------------------------ 762

B19_BF1_(AB426151) AGGTACGTGCCCCGCACCGAGTGGATGGCGGCCAA**CA**CGGACCAGCAGTACTGGGATGGA 1082

B19_BF1_(PacBio_P2a) AGGTACGTGCCCCGCACCGAGTGGATGGCGGCCAA**CA**CGGACCAGCAGTACTGGGATGGA 1079

B19_BF1_(AM279338) AGGTACGTGCCCCGCACCGAGTGGATGGCGGCCAA**CA**CGGACCAGCAGTACTGGGATGGA 645

B21_BF1_(AB426152) AGGTACGTGCCCCGCACCGAGTGGATGGCGGCCAAGGCGGACCAGCAGTACTGGGATGGA 1341

B21_BF1_(PacBio_N) AGGTACGTGCCCCGCACCGAGTGGATGGCGGCCAAGGCGGACCAGCAGTACTGGGATGGA 1341

B21_BF1_(AM279339) AGGTACGTGCCCCGCACCGAGTGGATGGCGGCCAAGGCGGACCAGCAGTACTGGGATGGA 904

B21_BF1_(PacBio_0) AGGTACGTGCCCCGCACCGAGTGGATGGCGGCCAAGGCGGACCAGCAGTACTGGGATGGA 1341

B21_BF1_(AM279342) AGGTACGTGCCCCGCACCGAGTGGATGGCGGCCAAGGCGGACCAGCAGTACTGGGATGGA 904

B2_BF1_(AB426141) **G**AGACGCAGATCG**C**ACAG**G**GCAATGAGC**A**GA**T**TG**ACCGC**G**A**GA**A**CCTGGACA**T**AC**G**GCAG 1384

B2_BF1_(PacBio_6sub1) **G**AGACGCAGATCG**C**ACAG**G**GCAATGAGC**A**GA**T**TG**ACCGC**G**A**GA**A**CCTGGACA**T**AC**G**GCAG 1384

B2_BF1_(AM279336) **G**AGACGCAGATCG**C**ACAG**G**GCAATGAGC**A**GA**T**TG**ACCGC**G**A**GA**A**CCTGGACA**T**AC**G**GCAG 948

B2_BF1_(PacBio_7sub2) **G**AGACGCAGATCG**C**ACAG**G**GCAATGAGC**A**GA**T**TG**ACCGC**G**A**GA**A**CCTGGACA**T**AC**G**GCAG 1384

B2_BF1_(AM279340) **G**AGACGCAGATCG**C**ACAG**G**GCAATGAGC**A**GA**T**TG**ACCGC**G**A**GA**A**CCTGGACA**T**AC**G**GCAG 948

B4_BF1_(AM279341) CAGACGCAGATCGGACAGCGCAATGAGCGGAGTGTGAAAGTGAGCCTGGACACACTGCAG 964

B4_BF1_(PacBio_C) CAGACGCAGATCGGACAGCGCAATGAGCGGAGTGTGAAAGTGAGCCTGGACACACTGCAG 1399

B4_BF1_(AM279337) CAGACGCAGATCGGACAGCGCAATGAGCGGAGTGTGAAAGTGAGCCTGGACACACTGCAG 964

B12_BF1_(AB426147) CAGACGCAGATCGGACAG**G**GCAATGAGCGGAGTGTGGAAGTGAGC**T**TGAACACACTGCAG 1142

B12_BF1(PacBio_C) CAGACGCAGATCGGACAG**G**GCAATGAGCGGAGTGTGGAAGTGAGC**T**TGAACACACTGCAG 1139

B12_BF1_(AL023516) CAGACGCAGATCGGACAG**G**GCAATGAGCGGAGTGTGGAAGTGAGC**T**TGAACACACTGCAG 1142

B14_BF1_(PacBio_WL) ------------------------------------------------------------ 762

B15_BF1_(AB426149) CAGACGCAGATCGGACAGCGCAATGAGCGGAGTGTGAAAGTGAGCCTGGACACACTGCAG 1401

B15_BF1_(PacBio_15I) ------------------------------------------------------------ 762

B19_BF1_(AB426151) CAGACGCAGATCGGACAG**G**GCAATGAGCGGAGTGTGAAAGTGAGC**T**TGAACACACTGCAG 1142

B19_BF1_(PacBio_P2a) CAGACGCAGATCGGACAG**G**GCAATGAGCGGAGTGTGAAAGTGAGC**T**TGAACACACTGCAG 1139

B19_BF1_(AM279338) CAGACGCAGATCGGACAG**G**GCAATGAGCGGAGTGTGAAAGTGAGC**T**TGAACACACTGCAG 705

B21_BF1_(AB426152) CAGACGCAGATCGGACAGCGCAATGAGCGGAGTGTGAAAGTGAGCCTGGACACACTGCAG 1401

B21_BF1_(PacBio_N) CAGACGCAGATCGGACAGCGCAATGAGCGGAGTGTGAAAGTGAGCCTGGACACACTGCAG 1401

B21_BF1_(AM279339) CAGACGCAGATCGGACAGCGCAATGAGCGGAGTGTGAAAGTGAGCCTGGACACACTGCAG 964

B21_BF1_(PacBio_0) CAGACGCAGATCGGACAGCGCAATGAGCGGAGTGTGAAAGTGAGCCTGGACACACTGCAG 1401

B21_BF1_(AM279342) CAGACGCAGATCGGACAGCGCAATGAGCGGAGTGTGAAAGTGAGCCTGGACACACTGCAG 964

Fig. S1

B2_BF1_(AB426141) **C**A**G**CG**CC**ACAACCAGACCGGCGGTGAGCACGGCCGGGGCCGCGGCTCCGTGGGTGTGGGA 1444

B2_BF1_(PacBio_6sub1) **C**A**G**CG**CC**ACAACCAGACCGGCGGTGAGCACGGCCGGGGCCGCGGCTCCGTGGGTGTGGGA 1444

B2_BF1_(AM279336) **C**A**G**CG**CC**ACAACCAGACCGGCGGTGAGCACGGCCGGGGCCGCGGCTCCGTGGGTGTGGGA 1008

B2_BF1_(PacBio_7sub2) **C**A**G**CG**CC**ACAACCAGACCGGCGGTGAGCACGGCCGGGGCCGCGGCTCCGTGGGTGTGGGA 1444

B2_BF1_(AM279340) **C**A**G**CG**CC**ACAACCAGACCGGCGGTGAGCACGGCCGGGGCCGCGGCTCCGTGGGTGTGGGA 1008

B4_BF1_(AM279341) GAACGATACAACCAGACCGGCGGTGAGCACGGCCGGGGCCGCGGCTCCGTGGGTGTGGGA 1024

B4_BF1_(PacBio_C) GAACGATACAACCAGACCGGCGGTGAGCACGGCCGGGGCCGCGGCTCCGTGGGTGTGGGA 1459

B4_BF1_(AM279337) GAACGATACAACCAGACCGGCGGTGAGCACGGCCGGGGCCGCGGCTCCGTGGGTGTGGGA 1024

B12_BF1_(AB426147) GAACGATACAACCAGACCGGCGGTGAGCACGGCCGGGGCCGCGGCTCCGTGGGTGTGGGA 1202

B12_BF1(PacBio_C) GAACGATACAACCAGACCGGCGGTGAGCACGGCCGGGGCCGCGGCTCCGTGGGTGTGGGA 1199

B12_BF1_(AL023516) GAACGATACAACCAGACCGGCGGTGAGCACGGCCGGGGCCGCGGCTCCGTGGGTGTGGGA 1202

B14_BF1_(PacBio_WL) ------------------------------------------------------------ 762

B15_BF1_(AB426149) GAACGATACAACCAGACCGGCGGTGAGCACGGCCGGGGCCGCGGCTCCGTGGGTGTGGGA 1461

B15_BF1_(PacBio_15I) ------------------------------------------------------------ 762

B19_BF1_(AB426151) GAACGATACAACCAGACCGGCGGTGAGCACGGCCGGGGCCGCGGCTCCGTGGGTGTGGGA 1202

B19_BF1_(PacBio_P2a) GAACGATACAACCAGACCGGCGGTGAGCACGGCCGGGGCCGCGGCTCCGTGGGTGTGGGA 1199

B19_BF1_(AM279338) GAACGATACAACCAGACCGGCGGTGAGCACGGCCGGGGCCGCGGCTCCGTGGGTGTGGGA 765

B21_BF1_(AB426152) GAACGATACAACCAGACCGGCGGTGAGCACGGCCGGGGCCGCGGCTCCGTGGGTGTGGGA 1461

B21_BF1_(PacBio_N) GAACGATACAACCAGACCGGCGGTGAGCACGGCCGGGGCCGCGGCTCCGTGGGTGTGGGA 1461

B21_BF1_(AM279339) GAACGATACAACCAGACCGGCGGTGAGCACGGCCGGGGCCGCGGCTCCGTGGGTGTGGGA 1024

B21_BF1_(PacBio_0) GAACGATACAACCAGACCGGCGGTGAGCACGGCCGGGGCCGCGGCTCCGTGGGTGTGGGA 1461

B21_BF1_(AM279342) GAACGATACAACCAGACCGGCGGTGAGCACGGCCGGGGCCGCGGCTCCGTGGGTGTGGGA 1024

B2_BF1_(AB426141) TGG**A**CTCCATGGCGCAGTGCCGCCCACACCCCCCAGGCCTGGCCCTGCCCGGCGGCACCG 1504

B2_BF1_(PacBio_6sub1) TGG**A**CTCCATGGCGCAGTGCCGCCCACACCCCCCAGGCCTGGCCCTGCCCGGCGGCACCG 1504

B2_BF1_(AM279336) TGG**A**CTCCATGGCGCAGTGCCGCCCACACCCCCCAGGCCTGGCCCTGCCCGGCGGCACCG 1068

B2_BF1_(PacBio_7sub2) TGG**A**CTCCATGGCGCAGTGCCGCCCACACCCCCCAGGCCTGGCCCTGCCCGGCGGCACCG 1504

B2_BF1_(AM279340) TGG**A**CTCCATGGCGCAGTGCCGCCCACACCCCCCAGGCCTGGCCCTGCCCGGCGGCACCG 1068

B4_BF1_(AM279341) TGGGCTCCATGGCGCAGTGCCGCCCACACCCCCCAGGCCTGGCCCTGCCCGGCGGCACCG 1084

B4_BF1_(PacBio_C) TGGGCTCCATGGCGCAGTGCCGCCCACACCCCCCAGGCCTGGCCCTGCCCGGCGGCACCG 1519

B4_BF1_(AM279337) TGGGCTCCATGGCGCAGTGCCGCCCACACCCCCCAGGCCTGGCCCTGCCCGGCGGCACCG 1084

B12_BF1_(AB426147) TGGGCTCCATGGCGCAGTGCCGCCCACACCCCCCAGGCCTGGCCCTGCCCGGCGGCACCG 1262

B12_BF1(PacBio_C) TGGGCTCCATGGCGCAGTGCCGCCCACACCCCCCAGGCCTGGCCCTGCCCGGCGGCACCG 1259

B12_BF1_(AL023516) TGGGCTCCATGGCGCAGTGCCGCCCACACCCCCCAGGCCTGGCCCTGCCCGGCGGCACCG 1262

B14_BF1_(PacBio_WL) ------------------------------------------------------------ 762

B15_BF1_(AB426149) TGGGCTCCATGGCGCAGTGCCGCCCACACCCCCCAGGCCTGGCCCTGCCCGGCGGCACCG 1521

B15_BF1_(PacBio_15I) ------------------------------------------------------------ 762

B19_BF1_(AB426151) TGGGCTCCATGGCGCAGTGCCGCCCACACCCCCCAGGCCTGGCCCTGCCCGGCGGCACCG 1262

B19_BF1_(PacBio_P2a) TGGGCTCCATGGCGCAGTGCCGCCCACACCCCCCAGGCCTGGCCCTGCCCGGCGGCACCG 1259

B19_BF1_(AM279338) TGGGCTCCATGGCGCAGTGCCGCCCACACCCCCCAGGCCTGGCCCTGCCCGGCGGCACCG 825

B21_BF1_(AB426152) TGGGCTCCATGGCGCAGTGCCGCCCACACCCCCCAGGCCTGGCCCTGCCCGGCGGCACCG 1521

B21_BF1_(PacBio_N) TGGGCTCCATGGCGCAGTGCCGCCCACACCCCCCAGGCCTGGCCCTGCCCGGCGGCACCG 1521

B21_BF1_(AM279339) TGGGCTCCATGGCGCAGTGCCGCCCACACCCCCCAGGCCTGGCCCTGCCCGGCGGCACCG 1084

B21_BF1_(PacBio_0) TGGGCTCCATGGCGCAGTGCCGCCCACACCCCCCAGGCCTGGCCCTGCCCGGCGGCACCG 1521

B21_BF1_(AM279342) TGGGCTCCATGGCGCAGTGCCGCCCACACCCCCCAGGCCTGGCCCTGCCCGGCGGCACCG 1084

B2_BF1_(AB426141) TCCCGGGGCTGCCCGTCACAGCCCCACCGCGCTCGGGGTGCCGCGTCCCGGGGGGACCCC 1564

B2_BF1_(PacBio_6sub1) TCCCGGGGCTGCCCGTCACAGCCCCACCGCGCTCGGGGTGCCGCGTCCCGGGGGGACCCC 1564

B2_BF1_(AM279336) TCCCGGGGCTGCCCGTCACAGCCCCACCGCGCTCGGGGTGCCGCGTCCCGGGGGGACCCC 1128

B2_BF1_(PacBio_7sub2) TCCCGGGGCTGCCCGTCACAGCCCCACCGCGCTCGGGGTGCCGCGTCCCGGGGGGACCCC 1564

B2_BF1_(AM279340) TCCCGGGGCTGCCCGTCACAGCCCCACCGCGCTCGGGGTGCCGCGTCCCGGGGGGACCCC 1128

B4_BF1_(AM279341) TCCCGGGGCTGCCCGTCACAGCCCCACCGCGCTCGGGGTGCCGCGTCCCGGGGGGACCCC 1144

B4_BF1_(PacBio_C) TCCCGGGGCTGCCCGTCACAGCCCCACCGCGCTCGGGGTGCCGCGTCCCGGGGGGACCCC 1579

B4_BF1_(AM279337) TCCCGGGGCTGCCCGTCACAGCCCCACCGCGCTCGGGGTGCCGCGTCCCGGGGGGACCCC 1144

B12_BF1_(AB426147) TCCCGGGGCTGCCCGTCACAGCCCCACCGCGCTCGGGGTGCCGCGTCCCGGGGGGACCCC 1322

B12_BF1(PacBio_C) TCCCGGGGCTGCCCGTCACAGCCCCACCGCGCTCGGGGTGCCGCGTCCCGGGGGGACCCC 1319

B12_BF1_(AL023516) TCCCGGGGCTGCCCGTCACAGCCCCACCGCGCTCGGGGTGCCGCGTCCCGGGGGGACCCC 1322

B14_BF1_(PacBio_WL) ------------------------------------------------------------ 762

B15_BF1_(AB426149) TCCCGGGGCTGCCCGTCACAGCCCCACCGCGCTCGGGGTGCCGCGTCCCGGGGGGACCCC 1581

B15_BF1_(PacBio_15I) ------------------------------------------------------------ 762

B19_BF1_(AB426151) TCCCGGGGCTGCCCGTCACAGCCCCACCGCGCTCGGGGTGCCGCGTCCCGGGGGGACCCC 1322

B19_BF1_(PacBio_P2a) TCCCGGGGCTGCCCGTCACAGCCCCACCGCGCTCGGGGTGCCGCGTCCCGGGGGGACCCC 1319

B19_BF1_(AM279338) TCCCGGGGCTGCCCGTCACAGCCCCACCGCGCTCGGGGTGCCGCGTCCCGGGGGGACCCC 885

B21_BF1_(AB426152) TCCCGGGGCTGCCCGTCACAGCCCCACCGCGCTCGGGGTGCCGCGTCCCGGGGGGACCCC 1581

B21_BF1_(PacBio_N) TCCCGGGGCTGCCCGTCACAGCCCCACCGCGCTCGGGGTGCCGCGTCCCGGGGGGACCCC 1581

B21_BF1_(AM279339) TCCCGGGGCTGCCCGTCACAGCCCCACCGCGCTCGGGGTGCCGCGTCCCGGGGGGACCCC 1144

B21_BF1_(PacBio_0) TCCCGGGGCTGCCCGTCACAGCCCCACCGCGCTCGGGGTGCCGCGTCCCGGGGGGACCCC 1581

B21_BF1_(AM279342) TCCCGGGGCTGCCCGTCACAGCCCCACCGCGCTCGGGGTGCCGCGTCCCGGGGGGACCCC 1144

Fig. S1

B2_BF1_(AB426141) AACCCATCCCCGCTGCAGTGGGAGCCCCGGAGCCGGAGGGGCCCCTCACCCCCTGCCCGG 1624

B2_BF1_(PacBio_6sub1) AACCCATCCCCGCTGCAGTGGGAGCCCCGGAGCCGGAGGGGCCCCTCACCCCCTGCCCGG 1624

B2_BF1_(AM279336) AACCCATCCCCGCTGCAGTGGGAGCCCCGGAGCCGGAGGGGCCCCTCACCCCCTGCCCGG 1188

B2_BF1_(PacBio_7sub2) AACCCATCCCCGCTGCAGTGGGAGCCCCGGAGCCGGAGGGGCCCCTCACCCCCTGCCCGG 1624

B2_BF1_(AM279340) AACCCATCCCCGCTGCAGTGGGAGCCCCGGAGCCGGAGGGGCCCCTCACCCCCTGCCCGG 1188

B4_BF1_(AM279341) AACCCATCCCCGCTGCAGTGGGAGCCCCGGAGCCGGAGGGGCCCCTCACCCCCTGCCCG**A** 1204

B4_BF1_(PacBio_C) AACCCATCCCCGCTGCAGTGGGAGCCCCGGAGCCGGAGGGGCCCCTCACCCCCTGCCCG**A** 1639

B4_BF1_(AM279337) AACCCATCCCCGCTGCAGTGGGAGCCCCGGAGCCGGAGGGGCCCCTCACCCCCTGCCCG**A** 1204

B12_BF1_(AB426147) AACCCATCCCCGCTGCAGTGGGAGCCCCGGAGCCGGAGGGGCCCCTCACCCCCTGCCCGG 1382

B12_BF1(PacBio_C) AACCCATCCCCGCTGCAGTGGGAGCCCCGGAGCCGGAGGGGCCCCTCACCCCCTGCCCGG 1379

B12_BF1_(AL023516) AACCCATCCCCGCTGCAGTGGGAGCCCCGGAGCCGGAGGGGCCCCTCACCCCCTGCCCGG 1382

B14_BF1_(PacBio_WL) ------------------------------------------------------------ 762

B15_BF1_(AB426149) AACCCATCCCCGCTGCAGTGGGAGCCCCGGAGCCGGAGGGGCCCCTCACCCCCTGCCCGG 1641

B15_BF1_(PacBio_15I) ------------------------------------------------------------ 762

B19_BF1_(AB426151) AACCCATCCCCGCTGCAGTGGGAGCCCCGGAGCCGGAGGGGCCCCTCACCCCCTGCCCGG 1382

B19_BF1_(PacBio_P2a) AACCCATCCCCGCTGCAGTGGGAGCCCCGGAGCCGGAGGGGCCCCTCACCCCCTGCCCGG 1379

B19_BF1_(AM279338) AACCCATCCCCGCTGCAGTGGGAGCCCCGGAGCCGGAGGGGCCCCTCACCCCCTGCCCGG 945

B21_BF1_(AB426152) AACCCATCCCCGCTGCAGTGGGAGCCCCGGAGCCGGAGGGGCCCCTCACCCCCTGCCCGG 1641

B21_BF1_(PacBio_N) AACCCATCCCCGCTGCAGTGGGAGCCCCGGAGCCGGAGGGGCCCCTCACCCCCTGCCCGG 1641

B21_BF1_(AM279339) AACCCATCCCCGCTGCAGTGGGAGCCCCGGAGCCGGAGGGGCCCCTCACCCCCTGCCCGG 1204

B21_BF1_(PacBio_0) AACCCATCCCCGCTGCAGTGGGAGCCCCGGAGCCGGAGGGGCCCCTCACCCCCTGCCCGG 1641

B21_BF1_(AM279342) AACCCATCCCCGCTGCAGTGGGAGCCCCGGAGCCGGAGGGGCCCCTCACCCCCTGCCCGG 1204

**BF1 exon 3**

B2_BF1_(AB426141) CTGTGTTTCAGGGTCTCACACGG**C**GCAGTGGATGTACGGCTGTGACATCCTCGAGGA**C**GG 1684

B2_BF1_(PacBio_6sub1) CTGTGTTTCAGGGTCTCACACGG**C**GCAGTGGATGTACGGCTGTGACATCCTCGAGGA**C**GG 1684

B2_BF1_(AM279336) CTGTGTTTCAGGGTCTCACACGG**C**GCAGTGGATGTACGGCTGTGACATCCTCGAGGA**C**GG 1248

B2_BF1_(PacBio_7sub2) CTGTGTTTCAGGGTCTCACACGG**C**GCAGTGGATGTACGGCTGTGACATCCTCGAGGA**C**GG 1684

B2_BF1_(AM279340) CTGTGTTTCAGGGTCTCACACGG**C**GCAGTGGATGTACGGCTGTGACATCCTCGAGGA**C**GG 1248

B4_BF1_(AM279341) CTGTGTTTCAGGGTCTCACACGGTGCAGTGGATGT**T**CGGCTGTGACATCCTCGAGGATGG 1264

B4_BF1_(PacBio_C) CTGTGTTTCAGGGTCTCACACGGTGCAGTGGATGT**T**CGGCTGTGACATCCTCGAGGATGG 1699

B4_BF1_(AM279337) CTGTGTTTCAGGGTCTCACACGGTGCAGTGGATGT**T**CGGCTGTGACATCCTCGAGGATGG 1264

B12_BF1_(AB426147) CTGTGTTTCAGGGTCTCACACGGTGCAG**CT**GATGTACGGCTGTGACATCCTCGAGGATGG 1442

B12_BF1(PacBio_C) CTGTGTTTCAGGGTCTCACACGGTGCAG**CT**GATGTACGGCTGTGACATCCTCGAGGATGG 1439

B12_BF1_(AL023516) CTGTGTTTCAGGGTCTCACACGGTGCAG**CT**GATGTACGGCTGTGACATCCTCGAGGATGG 1442

B14_BF1_(PacBio_WL) ------------------------------------------------------------ 762

B15_BF1_(AB426149) CTGTGTTTCAGGGTCTCACACGGTGCAGTGGATGTTCGGCTGTGACATCCTCGAGGATGG 1701

B15_BF1_(PacBio_15I) ------------------------------------------------------------ 762

B19_BF1_(AB426151) CTGTGTTTCAGGGTCTCACACGGTGCAG**CT**GATGTACGGCTGTGACATCCTCGAGGATGG 1442

B19_BF1_(PacBio_P2a) CTGTGTTTCAGGGTCTCACACGGTGCAG**CT**GATGTACGGCTGTGACATCCTCGAGGATGG 1439

B19_BF1_(AM279338) CTGTGTTTCAGGGTCTCACACGGTGCAG**CT**GATGTACGGCTGTGACATCCTCGAGGATGG 1005

B21_BF1_(AB426152) CTGTGTTTCAGGGTCTCACACGGTGCAGTGGATGT**T**CGGCTGTGACATCCTCGAGGATGG 1701

B21_BF1_(PacBio_N) CTGTGTTTCAGGGTCTCACACGGTGCAGTGGATGT**T**CGGCTGTGACATCCTCGAGGATGG 1701

B21_BF1_(AM279339) CTGTGTTTCAGGGTCTCACACGGTGCAGTGGATGT**T**CGGCTGTGACATCCTCGAGGATGG 1264

B21_BF1_(PacBio_0) CTGTGTTTCAGGGTCTCACACGGTGCAGTGGATGT**T**CGGCTGTGACATCCTCGAGGATGG 1701

B21_BF1_(AM279342) CTGTGTTTCAGGGTCTCACACGGTGCAGTGGATGT**T**CGGCTGTGACATCCTCGAGGATGG 1264

B2_BF1_(AB426141) CACCATCCGGGGGTATC**A**TCAGATGGCCT**G**CGATGGGA**G**AGACTTCATTGCC**C**TCG**CTG**A 1744

B2_BF1_(PacBio_6sub1) CACCATCCGGGGGTATC**A**TCAGATGGCCT**G**CGATGGGA**G**AGACTTCATTGCC**C**TCG**CTG**A 1744

B2_BF1_(AM279336) CACCATCCGGGGGTATC**A**TCAGATGGCCT**G**CGATGGGA**G**AGACTTCATTGCC**C**TCG**CTG**A 1308

B2_BF1_(PacBio_7sub2) CACCATCCGGGGGTATC**A**TCAGATGGCCT**G**CGATGGGA**G**AGACTTCATTGCC**C**TCG**CTG**A 1744

B2_BF1_(AM279340) CACCATCCGGGGGTATC**A**TCAGATGGCCT**G**CGATGGGA**G**AGACTTCATTGCC**C**TCG**CTG**A 1308

B4_BF1_(AM279341) CACCATCCGGGGGTATCGTCAG**G**TGGCCTACGATGGGAAAGACTTCATTGCCTTCGACAA 1324

B4_BF1_(PacBio_C) CACCATCCGGGGGTATCGTCAG**G**TGGCCTACGATGGGAAAGACTTCATTGCCTTCGACAA 1759

B4_BF1_(AM279337) CACCATCCGGGGGTATCGTCAG**G**TGGCCTACGATGGGAAAGACTTCATTGCCTTCGACAA 1324

B12_BF1_(AB426147) CACCATCCGGGGGTATC**A**TCAGA**CA**GCCTACGATGGGA**G**AGACTTCATTGCCTTCGACAA 1502

B12_BF1(PacBio_C) CACCATCCGGGGGTATC**A**TCAGA**CA**GCCTACGATGGGA**G**AGACTTCATTGCCTTCGACAA 1499

B12_BF1_(AL023516) CACCATCCGGGGGTATC**A**TCAGA**CA**GCCTACGATGGGA**G**AGACTTCATTGCCTTCGACAA 1502

B14_BF1_(PacBio_WL) ------------------------------------------------------------ 762

B15_BF1_(AB426149) CACCATCCGGGGGTATCGTCAGGTGGCCTACGATGGGAAAGACTTCATTGCCTTCGACAA 1761

B15_BF1_(PacBio_15I) ------------------------------------------------------------ 762

B19_BF1_(AB426151) CACCATCCGGGGGTATC**A**TCAGA**CA**GCCTACGATGGGA**G**AGACTTCATTGCCTTCGACAA 1502

B19_BF1_(PacBio_P2a) CACCATCCGGGGGTATC**A**TCAGA**CA**GCCTACGATGGGA**G**AGACTTCATTGCCTTCGACAA 1499

B19_BF1_(AM279338) CACCATCCGGGGGTATC**A**TCAGA**CA**GCCTACGATGGGA**G**AGACTTCATTGCCTTCGACAA 1065

B21_BF1_(AB426152) CACCATCCGGGGGTATCGTCAG**G**TGGCCTACGATGGGAAAGACTTCATTGCCTTCGACAA 1761

B21_BF1_(PacBio_N) CACCATCCGGGGGTATCGTCAG**G**TGGCCTACGATGGGAAAGACTTCATTGCCTTCGACAA 1761

B21_BF1_(AM279339) CACCATCCGGGGGTATCGTCAG**G**TGGCCTACGATGGGAAAGACTTCATTGCCTTCGACAA 1324

B21_BF1_(PacBio_0) CACCATCCGGGGGTATCGTCAG**G**TGGCCTACGATGGGAAAGACTTCATTGCCTTCGACAA 1761

B21_BF1_(AM279342) CACCATCCGGGGGTATCGTCAG**G**TGGCCTACGATGGGAAAGACTTCATTGCCTTCGACAA 1324

Fig. S1

B2_BF1_(AB426141) AGACATGAAGACGTTCACTGC**A**GCAGTTCCAGAGGCAGTTCCCACCAAGAGGAAATGGGA 1804

B2_BF1_(PacBio_6sub1) AGACATGAAGACGTTCACTGC**A**GCAGTTCCAGAGGCAGTTCCCACCAAGAGGAAATGGGA 1804

B2_BF1_(AM279336) AGACATGAAGACGTTCACTGC**A**GCAGTTCCAGAGGCAGTTCCCACCAAGAGGAAATGGGA 1368

B2_BF1_(PacBio_7sub2) AGACATGAAGACGTTCACTGC**A**GCAGTTCCAGAGGCAGTTCCCACCAAGAGGAAATGGGA 1804

B2_BF1_(AM279340) AGACATGAAGACGTTCACTGC**A**GCAGTTCCAGAGGCAGTTCCCACCAAGAGGAAATGGGA 1368

B4_BF1_(AM279341) AGACATGAAGACGTTCACTGCGGCAGTTCCAGAGGCAGTTCCCACCAAGAGGAAATGGGA 1384

B4_BF1_(PacBio_C) AGACATGAAGACGTTCACTGCGGCAGTTCCAGAGGCAGTTCCCACCAAGAGGAAATGGGA 1819

B4_BF1_(AM279337) AGACATGAAGACGTTCACTGCGGCAGTTCCAGAGGCAGTTCCCACCAAGAGGAAATGGGA 1384

B12_BF1_(AB426147) AG**G**CA**C**GA**T**GACGTTCACTGCGGCAGTTCCAGAGGCAGTTCCCACCAAGAGGAAATGGGA 1562

B12_BF1(PacBio_C) AG**G**CA**C**GA**T**GACGTTCACTGCGGCAGTTCCAGAGGCAGTTCCCACCAAGAGGAAATGGGA 1559

B12_BF1_(AL023516) AG**G**CA**C**GA**T**GACGTTCACTGCGGCAGTTCCAGAGGCAGTTCCCACCAAGAGGAAATGGGA 1562

B14_BF1_(PacBio_WL) ------------------------------------------------------------ 762

B15_BF1_(AB426149) AGACATGAAGACGTTCACTGCGGCAGTTCCAGAGGCAGTTCCCACCAAGAGGAAATGGGA 1821

B15_BF1_(PacBio_15I) ------------------------------------------------------------ 762

B19_BF1_(AB426151) AG**G**CA**C**GA**T**GACGTTCACTGCGGCAGTTCCAGAGGCAGTTCCCACCAAGAGGAAATGGGA 1562

B19_BF1_(PacBio_P2a) AG**G**CA**C**GA**T**GACGTTCACTGCGGCAGTTCCAGAGGCAGTTCCCACCAAGAGGAAATGGGA 1559

B19_BF1_(AM279338) AG**G**CA**C**GA**T**GACGTTCACTGCGGCAGTTCCAGAGGCAGTTCCCACCAAGAGGAAATGGGA 1125

B21_BF1_(AB426152) AGACATGAAGACGTTCACTGCGGCAGTTCCAGAGGCAGTTCCCACCAAGAGGAAATGGGA 1821

B21_BF1_(PacBio_N) AGACATGAAGACGTTCACTGCGGCAGTTCCAGAGGCAGTTCCCACCAAGAGGAAATGGGA 1821

B21_BF1_(AM279339) AGACATGAAGACGTTCACTGCGGCAGTTCCAGAGGCAGTTCCCACCAAGAGGAAATGGGA 1384

B21_BF1_(PacBio_0) AGACATGAAGACGTTCACTGCGGCAGTTCCAGAGGCAGTTCCCACCAAGAGGAAATGGGA 1821

B21_BF1_(AM279342) AGACATGAAGACGTTCACTGCGGCAGTTCCAGAGGCAGTTCCCACCAAGAGGAAATGGGA 1384

B2_BF1_(AB426141) GGAAGGAGGT**TA**TGCTGAG**A**GG**AA**GAAG**CAG**TACCTGGAGGAAACCTGCGTGGAG**G**GGCT 1864

B2_BF1_(PacBio_6sub1) GGAAGGAGGT**TA**TGCTGAG**A**GG**AA**GAAG**CAG**TACCTGGAGGAAACCTGCGTGGAG**G**GGCT 1864

B2_BF1_(AM279336) GGAAGGAGGT**TA**TGCTGAG**A**GG**AA**GAAG**CAG**TACCTGGAGGAAACCTGCGTGGAG**G**GGCT 1428

B2_BF1_(PacBio_7sub2) GGAAGGAGGT**TA**TGCTGAG**A**GG**AA**GAAG**CAG**TACCTGGAGGAAACCTGCGTGGAG**G**GGCT 1864

B2_BF1_(AM279340) GGAAGGAGGT**TA**TGCTGAG**A**GG**AA**GAAG**CAG**TACCTGGAGGAAACCTGCGTGGAG**G**GGCT 1428

B4_BF1_(AM279341) GGAAGGAGGTGTTGCTGAGGGGTGGAAGAGTTACCTGGAGGAAACCTGCGTGGAGTGGCT 1444

B4_BF1_(PacBio_C) GGAAGGAGGTGTTGCTGAGGGGTGGAAGAGTTACCTGGAGGAAACCTGCGTGGAGTGGCT 1879

B4_BF1_(AM279337) GGAAGGAGGTGTTGCTGAGGGGTGGAAGAGTTACCTGGAGGAAACCTGCGTGGAGTGGCT 1444

B12_BF1_(AB426147) GGAAGGAGGTGTTGCTGAG**A**GGTGGAAGAGTTACCTGGAGGAAACCTGCGTGGAG**G**GGCT 1622

B12_BF1(PacBio_C) GGAAGGAGGTGTTGCTGAG**A**GGTGGAAGAGTTACCTGGAGGAAACCTGCGTGGAG**G**GGCT 1619

B12_BF1_(AL023516) GGAAGGAGGTGTTGCTGAG**A**GGTGGAAGAGTTACCTGGAGGAAACCTGCGTGGAG**G**GGCT 1622

B14_BF1_(PacBio_WL) ------------------------------------------------------------ 762

B15_BF1_(AB426149) GGAAGGAGGTGTTGCTGAGGGGTGGAAGAGTTACCTGGAGGAAACCTGCGTGGAGTGGCT 1881

B15_BF1_(PacBio_15I) ------------------------------------------------------------ 762

B19_BF1_(AB426151) GGAAGGAGGTGTTGCTGAG**A**GGTGGAAGAGTTACCTGGAGGAAACCTGCGTGGAG**G**GGCT 1622

B19_BF1_(PacBio_P2a) GGAAGGAGGTGTTGCTGAG**A**GGTGGAAGAGTTACCTGGAGGAAACCTGCGTGGAG**G**GGCT 1619

B19_BF1_(AM279338) GGAAGGAGGTGTTGCTGAG**A**GGTGGAAGAGTTACCTGGAGGAAACCTGCGTGGAG**G**GGCT 1185

B21_BF1_(AB426152) GGAAGGAGGTGTTGCTGAGGGGTGGAAGAGTTACCTGGAGGAAACCTGCGTGGAGTGGCT 1881

B21_BF1_(PacBio_N) GGAAGGAGGTGTTGCTGAGGGGTGGAAGAGTTACCTGGAGGAAACCTGCGTGGAGTGGCT 1881

B21_BF1_(AM279339) GGAAGGAGGTGTTGCTGAGGGGTGGAAGAGTTACCTGGAGGAAACCTGCGTGGAGTGGCT 1444

B21_BF1_(PacBio_0) GGAAGGAGGTGTTGCTGAGGGGTGGAAGAGTTACCTGGAGGAAACCTGCGTGGAGTGGCT 1881

B21_BF1_(AM279342) GGAAGGAGGTGTTGCTGAGGGGTGGAAGAGTTACCTGGAGGAAACCTGCGTGGAGTGGCT 1444

B2_BF1_(AB426141) GCGGAGATACGTGGAATACGGGAAGGCTGAGCTGGGCAGGAGAGGTGAG**CA**GGGTGG--- 1921

B2_BF1_(PacBio_6sub1) GCGGAGATACGTGGAATACGGGAAGGCTGAGCTGGGCAGGAGAGGTGAG**CA**GGGTGG--- 1921

B2_BF1_(AM279336) GCGGAGATACGTGGAATACGGGAAGGCTGAGCTGGGCAGGAGAGGTGAG**CA**GGGTGG--- 1485

B2_BF1_(PacBio_7sub2) GCGGAGATACGTGGAATACGGGAAGGCTGAGCTGGGCAGGAGAGGTGAG**CA**GGGTGG--- 1921

B2_BF1_(AM279340) GCGGAGATACGTGGAATACGGGAAGGCTGAGCTGGGCAGGAGAGGTGAG**CA**GGGTGG--- 1485

B4_BF1_(AM279341) GCGGAGATACGTGGAATACGGGAAGGCTGAGCTGGGCAGGAGAGGTGAGTGGGGTGG--- 1501

B4_BF1_(PacBio_C) GCGGAGATACGTGGAATACGGGAAGGCTGAGCTGGGCAGGAGAGGTGAGTGGGGTGG--- 1936

B4_BF1_(AM279337) GCGGAGATACGTGGAATACGGGAAGGCTGAGCTGGGCAGGAGAGGTGAGTGGGGTGG--- 1501

B12_BF1_(AB426147) GCGGAGATA**T**GTGGAATACGGGAAGGCTGAGCTGGGCAGGAGAGGTGAG**C**GGGGT**C**G**GGG** 1682

B12_BF1(PacBio_C) GCGGAGATA**T**GTGGAATACGGGAAGGCTGAGCTGGGCAGGAGAGGTGAG**C**GGGGT**C**G**GGG** 1679

B12_BF1_(AL023516) GCGGAGATA**T**GTGGAATACGGGAAGGCTGAGCTGGGCAGGAGAGGTGAG**C**GGGGT**C**G**GGG** 1682

B14_BF1_(PacBio_WL) ------------------------------------------------------------ 762

B15_BF1_(AB426149) GCGGAGATACGTGGAATACGGGAAGGCTGAGCTGGGCAGGAGAGGTGAGTGGGGTGG--- 1938

B15_BF1_(PacBio_15I) ------------------------------------------------------------ 762

B19_BF1_(AB426151) GCGGAGATA**T**GTGGAATACGGGAAGGCTGAGCTGGGCAGGAGAGGTGAG**C**GGGGT**C**G**GGG** 1682

B19_BF1_(PacBio_P2a) GCGGAGATA**T**GTGGAATACGGGAAGGCTGAGCTGGGCAGGAGAGGTGAG**C**GGGGT**C**G**GGG** 1679

B19_BF1_(AM279338) GCGGAGATATGTGGAATACGGGAAGGCTGAGCTGGGCAGGAGAGGTGAG**C**GGGGT**C**G**GGG** 1245

B21_BF1_(AB426152) GCGGAGATACGTGGAATACGGGAAGGCTGAGCTGGGCAGGAGAGGTGAGTGGGGTGG--- 1938

B21_BF1_(PacBio_N) GCGGAGATACGTGGAATACGGGAAGGCTGAGCTGGGCAGGAGAGGTGAGTGGGGTGG--- 1938

B21_BF1_(AM279339) GCGGAGATACGTGGAATACGGGAAGGCTGAGCTGGGCAGGAGAGGTGAGTGGGGTGG--- 1501

B21_BF1_(PacBio_0) GCGGAGATACGTGGAATACGGGAAGGCTGAGCTGGGCAGGAGAGGTGAGTGGGGTGG--- 1938

B21_BF1_(AM279342) GCGGAGATACGTGGAATACGGGAAGGCTGAGCTGGGCAGGAGAGGTGAGTGGGGTGG--- 1501

Fig. S1

B2_BF1_(AB426141) -----------GGGGGGGGCCGC**A**GTGTGGGGCTGGACGTGGG**C**CGGGGGCTCAG**T**GTGG 1970

B2_BF1_(PacBio_6sub1) -----------GGGGGGGGCCGC**A**GTGTGGGGCTGGACGTGGG**C**CGGGGGCTCAG**T**GTGG 1970

B2_BF1_(AM279336) -----------GGGGGGGGCCGC**A**GTGTGGGGCTGGACGTGGG**C**CGGGGGCTCAG**T**GTGG 1534

B2_BF1_(PacBio_7sub2) -----------GGGGGGGGCCGC**A**GTGTGGGGCTGGACGTGGG**C**CGGGGGCTCAG**T**GTGG 1970

B2_BF1_(AM279340) -----------GGGGGGGGCCGC**A**GTGTGGGGCTGGACGTGGG**C**CGGGGGCTCAG**T**GTGG 1534

B4_BF1_(AM279341) ------------GGGGGGGCCGCGGTGTGGGGCTGGACGTGGGGCGGGGGCTCAGCGTGG 1549

B4_BF1_(PacBio_C) -----------GGGGGGGGCCGCGGTGTGGGGCTGGACGTGGGGCGGGGGCTCAGCGTGG 1985

B4_BF1_(AM279337) ------------GGGGGGGCCGCGGTGTGGGGCTGGACGTGGGGCGGGGGCTCAGCGTGG 1549

B12_BF1_(AB426147) **TGGGGGGGGGG**GGGGGGGG**A**C**C**C**A**GTGTGGGGCTGGAC**T**TGGGGCGGGGGCTCA**T**CGTGG 1742

B12_BF1(PacBio_C) **GGGGGGGGGGG**GGGGGCGG**A**CGC**A**GTGTGGGGCTGGACGTGGGGCGGGGGCTCA**T**CGTGG 1739

B12_BF1_(AL023516) **TGGGGGGGGGG**GGGGGCGG**A**C**C**C**A**GTGTGGGGCTGGAC**T**TGGGGCGGGGGCTCA**T**CGTGG 1742

B14_BF1_(PacBio_WL) ------------------------------------------------------------ 762

B15_BF1_(AB426149) -----------GGGGGGGGCCGCGGTGTGGGGCTGGACGTGGGGCGGGGGCTCAGCGTGG 1987

B15_BF1_(PacBio_15I) ------------------------------------------------------------ 762

B19_BF1_(AB426151) **TGGGGGGGG--**GGGGGCGG**A**CGC**A**GTGTGGGGCTGGACGTGGGGCGGGGGCTCA**T**CGTGG 1740

B19_BF1_(PacBio_P2a) **TGGGGGGGGGG**GGGGGCGG**A**CGC**A**GTGTGGGGCTGGACGTGGGGCGGGGGCTCA**T**CGTGG 1739

B19_BF1_(AM279338) **TGGGGGGGGGG**GGGGGCGG**A**CGC**A**GTGTGGGGCTGGACGTGGGGCGGGGGCTCA**T**CGTGG 1305

B21_BF1_(AB426152) -----------GGGGGGGGCCGCGGTGTGGGGCTGGACGTGGGGCGGGGGCTCAGCGTGG 1987

B21_BF1_(PacBio_N) -----------GGGGGGGGCCGCGGTGTGGGGCTGGACGTGGGGCGGGGGCTCAGCGTGG 1987

B21_BF1_(AM279339) -----------GGGGGGGGCCGCGGTGTGGGGCTGGACGTGGGGCGGGGGCTCAGCGTGG 1550

B21_BF1_(PacBio_0) -----------GGGGGGGGCCGCGGTGTGGGGCTGGACGTGGGGCGGGGGCTCAGCGTGG 1987

B21_BF1_(AM279342) -----------GGGGGGGGCCGCGGTGTGGGGCTGGACGTGGGGCGGGGGCTCAGCGTGG 1550

**BF1 exon 4**

B2_BF1_(AB426141) GGAGCTCAGCCCGGCCCTCATTGCCACC**C**GCCTGCAGAGCGGCCTGAGGTGCGAGTGTGG 2030

B2_BF1_(PacBio_6sub1) GGAGCTCAGCCCGGCCCTCATTGCCACC**C**GCCTGCAGAGCGGCCTGAGGTGCGAGTGTGG 2030

B2_BF1_(AM279336) GGAGCTCAGCCCGGCCCTCATTGCCACC**C**GCCTGCAGAGCGGCCTGAGGTGCGAGTGTGG 1594

B2_BF1_(PacBio_7sub2) GGAGCTCAGCCCGGCCCTCATTGCCACC**C**GCCTGCAGAGCGGCCTGAGGTGCGAGTGTGG 2030

B2_BF1_(AM279340) GGAGCTCAGCCCGGCCCTCATTGCCACC**C**GCCTGCAGAGCGGCCTGAGGTGCGAGTGTGG 1594

B4_BF1_(AM279341) GGAGCTCAGCCCGGCCCTCATTGCCACCTGCCTGCAGAGCGGCCTGAGGTGCGAGTGTGG 1609

B4_BF1_(PacBio_C) GGAGCTCAGCCCGGCCCTCATTGCCACCTGCCTGCAGAGCGGCCTGAGGTGCGAGTGTGG 2045

B4_BF1_(AM279337) GGAGCTCAGCCCGGCCCTCATTGCCACCTGCCTGCAGAGCGGCCTGAGGTGCGAGTGTGG 1609

B12_BF1_(AB426147) GGAGCTCAGCCCGGCCCTCA**C**TGCC**G**CC**CA**CC**CA**CAGAGCGGCCTGAGGTGCGAGTGTGG 1802

B12_BF1(PacBio_C) GGAGCTCAGCCCGGCCCTCA**C**TGCC**G**CC**CA**CC**CA**CAGAGCGGCCTGAGGTGCGAGTGTGG 1799

B12_BF1_(AL023516) GGAGCTCAGCCCGGCCCTCA**C**TGCC**G**CC**CA**CC**CA**CAGAGCGGCCTGAGGTGCGAGTGTGG 1802

B14_BF1_(PacBio_WL) ------------------------------------------------------------ 762

B15_BF1_(AB426149) GGAGCTCAGCCCGGCCCTCATTGCCACCTGCCTGCAGAGCGGCCCGAGGTGCGAGTGTGG 2047

B15_BF1_(PacBio_15I) ------------------------------------------------------------ 762

B19_BF1_(AB426151) GGAGCTCAGCCCGGCCCTCA**C**TGCC**G**CC**CA**CC**CA**CAGAGCGGCCTGAGGTGCGAGTGTGG 1800

B19_BF1_(PacBio_P2a) GGAGCTCAGCCCGGCCCTCA**C**TGCC**G**CC**CA**CC**CA**CAGAGCGGCCTGAGGTGCGAGTGTGG 1799

B19_BF1_(AM279338) GGAGCTCAGCCCGGCCCTCA**C**TGCC**G**CC**CA**CC**CA**CAGAGCGGCCTGAGGTGCGAGTGTGG 1365

B21_BF1_(AB426152) GGAGCTCAGCCCGGCCCTCATTGCCACCTGCCTGCAGAGCGGCC**C**GAGGTGCGAGTGTGG 2047

B21_BF1_(PacBio_N) GGAGCTCAGCCCGGCCCTCATTGCCACCTGCCTGCAGAGCGGCC**C**GAGGTGCGAGTGTGG 2047

B21_BF1_(AM279339) GGAGCTCAGCCCGGCCCTCATTGCCACCTGCCTGCAGAGCGGCC**C**GAGGTGCGAGTGTGG 1610

B21_BF1_(PacBio_0) GGAGCTCAGCCCGGCCCTCATTGCCACCTGCCTGCAGAGCGGCC**C**GAGGTGCGAGTGTGG 2047

B21_BF1_(AM279342) GGAGCTCAGCCCGGCCCTCATTGCCACCTGCCTGCAGAGCGGCC**C**GAGGTGCGAGTGTGG 1610

B2_BF1_(AB426141) GGGAAGGAGGCCGACGGGATCCTGACCTTGTCCTGCCGCGCTCACGGCTTCTACCCGCGG 2090

B2_BF1_(PacBio_6sub1) GGGAAGGAGGCCGACGGGATCCTGACCTTGTCCTGCCGCGCTCACGGCTTCTACCCGCGG 2090

B2_BF1_(AM279336) GGGAAGGAGGCCGACGGGATCCTGACCTTGTCCTGCCGCGCTCACGGCTTCTACCCGCGG 1654

B2_BF1_(PacBio_7sub2) GGGAAGGAGGCCGACGGGATCCTGACCTTGTCCTGCCGCGCTCACGGCTTCTACCCGCGG 2090

B2_BF1_(AM279340) GGGAAGGAGGCCGACGGGATCCTGACCTTGTCCTGCCGCGCTCACGGCTTCTACCCGCGG 1654

B4_BF1_(AM279341) GGGAAGGAGGCCGACGGGATCCTGACCTTGTCCTGCCGCGCTCACGGCTTCTACCCGCGG 1669

B4_BF1_(PacBio_C) GGGAAGGAGGCCGACGGGATCCTGACCTTGTCCTGCCGCGCTCACGGCTTCTACCCGCGG 2105

B4_BF1_(AM279337) GGGAAGGAGGCCGACGGGATCCTGACCTTGTCCTGCCGCGCTCACGGCTTCTACCCGCGG 1669

B12_BF1_(AB426147) GGGAAGGAGGC**T**GACGGGATCCTGACCTTGTCCTGCCGCGCTCACGGCTTCTACCCGCGG 1862

B12_BF1(PacBio_C) GGGAAGGAGGC**T**GACGGGATCCTGACCTTGTCCTGCCGCGCTCACGGCTTCTACCCGCGG 1859

B12_BF1_(AL023516) GGGAAGGAGGC**T**GACGGGATCCTGACCTTGTCCTGCCGCGCTCACGGCTTCTACCCGCGG 1862

B14_BF1_(PacBio_WL) ------------------------------------------------------------ 762

B15_BF1_(AB426149) GGGAAGGAGGCCGACGGGATCCTGACCTTGTCCTGCCGCGCTCACGGCTTCTACCCGCGG 2107

B15_BF1_(PacBio_15I) ------------------------------------------------------------ 762

B19_BF1_(AB426151) GGGAAGGAGGC**T**GACGGGATCCTGACCTTGTCCTGCCGCGCTCACGGCTTCTACCCGCGG 1860

B19_BF1_(PacBio_P2a) GGGAAGGAGGC**T**GACGGGATCCTGACCTTGTCCTGCCGCGCTCACGGCTTCTACCCGCGG 1859

B19_BF1_(AM279338) GGGAAGGAGGC**T**GACGGGATCCTGACCTTGTCCTGCCGCGCTCACGGCTTCTACCCGCGG 1425

B21_BF1_(AB426152) GGGAAGGAGGCCGACGGGATCCTGACCTTGTCCTGCCGCGCTCACGGCTTCTACCCGCGG 2107

B21_BF1_(PacBio_N) GGGAAGGAGGCCGACGGGATCCTGACCTTGTCCTGCCGCGCTCACGGCTTCTACCCGCGG 2107

B21_BF1_(AM279339) GGGAAGGAGGCCGACGGGATCCTGACCTTGTCCTGCCGCGCTCACGGCTTCTACCCGCGG 1670

B21_BF1_(PacBio_0) GGGAAGGAGGCCGACGGGATCCTGACCTTGTCCTGCCGCGCTCACGGCTTCTACCCGCGG 2107

B21_BF1_(AM279342) GGGAAGGAGGCCGACGGGATCCTGACCTTGTCCTGCCGCGCTCACGGCTTCTACCCGCGG 1670

Fig. S1

B2_BF1_(AB426141) CCCATCGTTGTCAGCTGGCTGAAGGACGGCGCGGTGCGGGGCCAGGACGCCCAGTCGGGG 2150

B2_BF1_(PacBio_6sub1) CCCATCGTTGTCAGCTGGCTGAAGGACGGCGCGGTGCGGGGCCAGGACGCCCAGTCGGGG 2150

B2_BF1_(AM279336) CCCATCGTTGTCAGCTGGCTGAAGGACGGCGCGGTGCGGGGCCAGGACGCCCAGTCGGGG 1714

B2_BF1_(PacBio_7sub2) CCCATCGTTGTCAGCTGGCTGAAGGACGGCGCGGTGCGGGGCCAGGACGCCCAGTCGGGG 2150

B2_BF1_(AM279340) CCCATCGTTGTCAGCTGGCTGAAGGACGGCGCGGTGCGGGGCCAGGACGCCCAGTCGGGG 1714

B4_BF1_(AM279341) CCCATCG**C**TGTCAGCTGGCTGAAGGACGGCGCGGTGCGGGGCCAGGACGCCCAGTCGGGG 1729

B4_BF1_(PacBio_C) CCCATCG**C**TGTCAGCTGGCTGAAGGACGGCGCGGTGCGGGGCCAGGACGCCCAGTCGGGG 2165

B4_BF1_(AM279337) CCCATCG**C**TGTCAGCTGGCTGAAGGACGGCGCGGTGCGGGGCCAGGACGCCCAGTCGGGG 1729

B12_BF1_(AB426147) CCCATCG**CC**GTCAGCTGGCTGAAGGACGGCGCGGTGCGGGGCCAGGACGCCCAGTCGGGG 1922

B12_BF1(PacBio_C) CCCATCG**CC**GTCAGCTGGCTGAAGGACGGCGCGGTGCGGGGCCAGGACGCCCAGTCGGGG 1919

B12_BF1_(AL023516) CCCATCG**CC**GTCAGCTGGCTGAAGGACGGCGCGGTGCGGGGCCAGGACGCCCAGTCGGGG 1922

B14_BF1_(PacBio_WL) ------------------------------------------------------------ 762

B15_BF1_(AB426149) CCCATCGTTGTCAGCTGGCTGAAGGACGGCGCAGTGCGGGGCCAGGACGCCCAGTCGGGG 2167

B15_BF1_(PacBio_15I) ------------------------------------------------------------ 762

B19_BF1_(AB426151) CCCATCG**CC**GTCAGCTGGCTGAAGGACGGCGCGGTGCGGGGCCAGGACGCCCAGTCGGGG 1920

B19_BF1_(PacBio_P2a) CCCATCG**CC**GTCAGCTGGCTGAAGGACGGCGCGGTGCGGGGCCAGGACGCCCAGTCGGGG 1919

B19_BF1_(AM279338) CCCATCG**CC**GTCAGCTGGCTGAAGGACGGCGCGGTGCGGGGCCAGGACGCCCAGTCGGGG 1485

B21_BF1_(AB426152) CCCATCGTTGTCAGCTGGCTGAAGGACGGCGC**A**GTGCGGGGCCAGGACGCCCAGTCGGGG 2167

B21_BF1_(PacBio_N) CCCATCGTTGTCAGCTGGCTGAAGGACGGCGC**A**GTGCGGGGCCAGGACGCCCAGTCGGGG 2167

B21_BF1_(AM279339) CCCATCGTTGTCAGCTGGCTGAAGGACGGCGC**A**GTGCGGGGCCAGGACGCCCAGTCGGGG 1730

B21_BF1_(PacBio_0) CCCATCGTTGTCAGCTGGCTGAAGGACGGCGC**A**GTGCGGGGCCAGGACGCCCAGTCGGGG 2167

B21_BF1_(AM279342) CCCATCGTTGTCAGCTGGCTGAAGGACGGCGC**A**GTGCGGGGCCAGGACGCCCAGTCGGGG 1730

B2_BF1_(AB426141) GGCATC**G**TGCCCAACGGCGA**C**GGCACCTACCACACCTGGGTCACCATCGATGCGCAGCCG 2210

B2_BF1_(PacBio_6sub1) GGCATC**G**TGCCCAACGGCGA**C**GGCACCTACCACACCTGGGTCACCATCGATGCGCAGCCG 2210

B2_BF1_(AM279336) GGCATC**G**TGCCCAACGGCGA**C**GGCACCTACCACACCTGGGTCACCATCGATGCGCAGCCG 1774

B2_BF1_(PacBio_7sub2) GGCATC**G**TGCCCAACGGCGA**C**GGCACCTACCACACCTGGGTCACCATCGATGCGCAGCCG 2210

B2_BF1_(AM279340) GGCATC**G**TGCCCAACGGCGA**C**GGCACCTACCACACCTGGGTCACCATCGATGCGCAGCCG 1774

B4_BF1_(AM279341) GGCATCATGCCCAACGGCGATGGCACCTACCACACCTGGGTCACCATCGATG**T**GCAGCCG 1789

B4_BF1_(PacBio_C) GGCATCATGCCCAACGGCGATGGCACCTACCACACCTGGGTCACCATCGATG**T**GCAGCCG 2225

B4_BF1_(AM279337) GGCATCATGCCCAACGGCGATGGCACCTACCACACCTGGGTCACCATCGATG**T**GCAGCCG 1789

B12_BF1_(AB426147) GGCATC**G**TGCCCAACGGCGA**C**GGCACCTACCACACCTGGGTCACCATCGATGCGCAGCCG 1982

B12_BF1(PacBio_C) GGCATC**G**TGCCCAACGGCGA**C**GGCACCTACCACACCTGGGTCACCATCGATGCGCAGCCG 1979

B12_BF1_(AL023516) GGCATC**G**TGCCCAACGGCGA**C**GGCACCTACCACACCTGGGTCACCATCGATGCGCAGCCG 1982

B14_BF1_(PacBio_WL) ------------------------------------------------------------ 762

B15_BF1_(AB426149) GGCATCATGCCCAA**T**GGCGATGGCACCTACCACACCTGGGTCACCATCGATGCGCAGCCG 2227

B15_BF1_(PacBio_15I) ------------------------------------------------------------ 762

B19_BF1_(AB426151) GGCATC**G**TGCCCAACGGCGA**C**GGCACCTACCACACCTGGGTCACCATCGATGCGCAGCCG 1980

B19_BF1_(PacBio_P2a) GGCATC**G**TGCCCAACGGCGA**C**GGCACCTACCACACCTGGGTCACCATCGATGCGCAGCCG 1979

B19_BF1_(AM279338) GGCATC**G**TGCCCAACGGCGA**C**GGCACCTACCACACCTGGGTCACCATCGATGCGCAGCCG 1545

B21_BF1_(AB426152) GGCATCATGCCCAA**T**GGCGATGGCACCTACCACACCTGGGTCACCATCGATGCGCAGCCG 2227

B21_BF1_(PacBio_N) GGCATCATGCCCAA**T**GGCGATGGCACCTACCACACCTGGGTCACCATCGATGCGCAGCCG 2227

B21_BF1_(AM279339) GGCATCATGCCCAA**T**GGCGATGGCACCTACCACACCTGGGTCACCATCGATGCGCAGCCG 1790

B21_BF1_(PacBio_0) GGCATCATGCCCAA**T**GGCGATGGCACCTACCACACCTGGGTCACCATCGATGCGCAGCCG 2227

B21_BF1_(AM279342) GGCATCATGCCCAA**T**GGCGATGGCACCTACCACACCTGGGTCACCATCGATGCGCAGCCG 1790

B2_BF1_(AB426141) GGGGACGGGGACAAGTACCAGTGCCGCGTGGAGCACGCCAGCCTGCCCCAGCCCGGCCTC 2270

B2_BF1_(PacBio_6sub1) GGGGACGGGGACAAGTACCAGTGCCGCGTGGAGCACGCCAGCCTGCCCCAGCCCGGCCTC 2270

B2_BF1_(AM279336) GGGGACGGGGACAAGTACCAGTGCCGCGTGGAGCACGCCAGCCTGCCCCAGCCCGGCCTC 1834

B2_BF1_(PacBio_7sub2) GGGGACGGGGACAAGTACCAGTGCCGCGTGGAGCACGCCAGCCTGCCCCAGCCCGGCCTC 2270

B2_BF1_(AM279340) GGGGACGGGGACAAGTACCAGTGCCGCGTGGAGCACGCCAGCCTGCCCCAGCCCGGCCTC 1834

B4_BF1_(AM279341) GGGGACGGGGACAAGTACCAGTGCCGCGTGGAGCACGCCAGCCTGCCCCAGCCCGGCCTC 1849

B4_BF1_(PacBio_C) GGGGACGGGGACAAGTACCAGTGCCGCGTGGAGCACGCCAGCCTGCCCCAGCCCGGCCTC 2285

B4_BF1_(AM279337) GGGGACGGGGACAAGTACCAGTGCCGCGTGGAGCACGCCAGCCTGCCCCAGCCCGGCCTC 1849

B12_BF1_(AB426147) GGGGACGGGGACAAGTACCAGTGCCGCGTGGAGCACGCCAGCCTGCCCCAGCCCGGCCTC 2042

B12_BF1(PacBio_C) GGGGACGGGGACAAGTACCAGTGCCGCGTGGAGCACGCCAGCCTGCCCCAGCCCGGCCTC 2039

B12_BF1_(AL023516) GGGGACGGGGACAAGTACCAGTGCCGCGTGGAGCACGCCAGCCTGCCCCAGCCCGGCCTC 2042

B14_BF1_(PacBio_WL) ------------------------------------------------------------ 762

B15_BF1_(AB426149) GGGGACGGGGA**T**AAGTACCAGTGCCGCGTGGAGCACGCCAGCCTGCCCCAGCCCGGCCTC 2287

B15_BF1_(PacBio_15I) ------------------------------------------------------------ 762

B19_BF1_(AB426151) GGGGACGGGGACAAGTACCAGTGCCGCGTGGAGCACGCCAGCCTGCCCCAGCCCGGCCTC 2040

B19_BF1_(PacBio_P2a) GGGGACGGGGACAAGTACCAGTGCCGCGTGGAGCACGCCAGCCTGCCCCAGCCCGGCCTC 2039

B19_BF1_(AM279338) GGGGACGGGGACAAGTACCAGTGCCGCGTGGAGCACGCCAGCCTGCCCCAGCCCGGCCTC 1605

B21_BF1_(AB426152) GGGGACGGGGA**T**AAGTACCAGTGCCGCGTGGAGCACGCCAGCCTGCCCCAGCCCGGCCTC 2287

B21_BF1_(PacBio_N) GGGGACGGGGA**T**AAGTACCAGTGCCGCGTGGAGCACGCCAGCCTGCCCCAGCCCGGCCTC 2287

B21_BF1_(AM279339) GGGGACGGGGA**T**AAGTACCAGTGCCGCGTGGAGCACGCCAGCCTGCCCCAGCCCGGCCTC 1850

B21_BF1_(PacBio_0) GGGGACGGGGA**T**AAGTACCAGTGCCGCGTGGAGCACGCCAGCCTGCCCCAGCCCGGCCTC 2287

B21_BF1_(AM279342) GGGGACGGGGA**T**AAGTACCAGTGCCGCGTGGAGCACGCCAGCCTGCCCCAGCCCGGCCTC 1850

Fig. S1

B2_BF1_(AB426141) TACTCGTGGGGTGAGTGAGGGGATGTGGGGCTGGGGGGCTGCGGGCTGCCCCTTCCCCTG 2330

B2_BF1_(PacBio_6sub1) TACTCGTGGGGTGAGTGAGGGGATGTGGGGCTGGGGGGCTGCGGGCTGCCCCTTCCCCTG 2330

B2_BF1_(AM279336) TACTCGTGGGGTGAGTGAGGGGATGTGGGGCTGGGGGGCTGCGGGCTGCCCCTTCCCCTG 1894

B2_BF1_(PacBio_7sub2) TACTCGTGGGGTGAGTGAGGGGATGTGGGGCTGGGGGGCTGCGGGCTGCCCCTTCCCCTG 2330

B2_BF1_(AM279340) TACTCGTGGGGTGAGTGAGGGGATGTGGGGCTGGGGGGCTGCGGGCTGCCCCTTCCCCTG 1894

B4_BF1_(AM279341) TACTCGTGGGGTGAGTGAGGGGATGTGGGGCTGGGGGGCTGCGGGCTGCCCCTTCCCCTG 1909

B4_BF1_(PacBio_C) TACTCGTGGGGTGAGTGAGGGGATGTGGGGCTGGGGGGCTGCGGGCTGCCCCTTCCCCTG 2345

B4_BF1_(AM279337) TACTCGTGGGGTGAGTGAGGGGATGTGGGGCTGGGGGGCTGCGGGCTGCCCCTTCCCCTG 1909

B12_BF1_(AB426147) TACTCGTGGGGTGAGTGAGGGGATGTGGGGCTGGGGGGCTGCGGGCTGCCCCTTCCCCTG 2102

B12_BF1(PacBio_C) TACTCGTGGGGTGAGTGAGGGGATGTGGGGCTGGGGGGCTGCGGGCTGCCCCTTCCCCTG 2099

B12_BF1_(AL023516) TACTCGTGGGGTGAGTGAGGGGATGTGGGGCTGGGGGGCTGCGGGCTGCCCCTTCCCCTG 2102

B14_BF1_(PacBio_WL) ------------------------------------------------------------ 762

B15_BF1_(AB426149) TACTCGTGGGGTGAGTGAGGGGATGTGGGGCTGGGGGGCTGCGGGCTGCCCCTTCCCCTG 2347

B15_BF1_(PacBio_15I) ------------------------------------------------------------ 762

B19_BF1_(AB426151) TACTCGTGGGGTGAGTGAGGGGATGTGGGGCTGGGGGGCTGCGGGCTGCCCCTTCCCCTG 2100

B19_BF1_(PacBio_P2a) TACTCGTGGGGTGAGTGAGGGGATGTGGGGCTGGGGGGCTGCGGGCTGCCCCTTCCCCTG 2099

B19_BF1_(AM279338) TACTCGTGGGGTGAGTGAGGGGATGTGGGGCTGGGGGGCTGCGGGCTGCCCCTTCCCCTG 1665

B21_BF1_(AB426152) TACTCGTGGGGTGAGTGAGGGGATGTGGGGCTGGGGGGCTGCGGGCTGCCCCTTCCCCTG 2347

B21_BF1_(PacBio_N) TACTCGTGGGGTGAGTGAGGGGATGTGGGGCTGGGGGGCTGCGGGCTGCCCCTTCCCCTG 2347

B21_BF1_(AM279339) TACTCGTGGGGTGAGTGAGGGGATGTGGGGCTGGGGGGCTGCGGGCTGCCCCTTCCCCTG 1910

B21_BF1_(PacBio_0) TACTCGTGGGGTGAGTGAGGGGATGTGGGGCTGGGGGGCTGCGGGCTGCCCCTTCCCCTG 2347

B21_BF1_(AM279342) TACTCGTGGGGTGAGTGAGGGGATGTGGGGCTGGGGGGCTGCGGGCTGCCCCTTCCCCTG 1910

**BF1 exon 5**

B2_BF1_(AB426141) CTGATGGCCCCGCTCTCCCCCAGAGCCGCCACAGCCCAACCTGGTGCCCATCGTGGCGGG 2390

B2_BF1_(PacBio_6sub1) CTGATGGCCCCGCTCTCCCCCAGAGCCGCCACAGCCCAACCTGGTGCCCATCGTGGCGGG 2390

B2_BF1_(AM279336) CTGATGGCCCCGCTCTCCCCCAGAGCCGCCACAGCCCAACCTGGTGCCCATCGTGGCGGG 1954

B2_BF1_(PacBio_7sub2) CTGATGGCCCCGCTCTCCCCCAGAGCCGCCACAGCCCAACCTGGTGCCCATCGTGGCGGG 2390

B2_BF1_(AM279340) CTGATGGCCCCGCTCTCCCCCAGAGCCGCCACAGCCCAACCTGGTGCCCATCGTGGCGGG 1954

B4_BF1_(AM279341) CTGATGGCCCCGCTCTCCCCCAGAGCCGCCACAGCCCAACCTGGTGCCCATCGTGGCGGG 1969

B4_BF1_(PacBio_C) CTGATGGCCCCGCTCTCCCCCAGAGCCGCCACAGCCCAACCTGGTGCCCATCGTGGCGGG 2405

B4_BF1_(AM279337) CTGATGGCCCCGCTCTCCCCCAGAGCCGCCACAGCCCAACCTGGTGCCCATCGTGGCGGG 1969

B12_BF1_(AB426147) CTGATGGCCCCGCTCTCCCCCAGAGCCGCCACAGCCCAACCTGGTGCCCATCGTGGCGGG 2162

B12_BF1(PacBio_C) CTGATGGCCCCGCTCTCCCCCAGAGCCGCCACAGCCCAACCTGGTGCCCATCGTGGCGGG 2159

B12_BF1_(AL023516) CTGATGGCCCCGCTCTCCCCCAGAGCCGCCACAGCCCAACCTGGTGCCCATCGTGGCGGG 2162

B14_BF1_(PacBio_WL) ------------------------------------------------------------ 762

B15_BF1_(AB426149) CTGATGGCCCCGCTCTCCCCCAGAGCCGCCACAGCCCAACCTGGTGCCCATCGTGGCGGG 2407

B15_BF1_(PacBio_15I) ------------------------------------------------------------ 762

B19_BF1_(AB426151) CTGATGGCCCCGCTCTCCCCCAGAGCCGCCACAGCCCAACCTGGTGCCCATCGTGGCGGG 2160

B19_BF1_(PacBio_P2a) CTGATGGCCCCGCTCTCCCCCAGAGCCGCCACAGCCCAACCTGGTGCCCATCGTGGCGGG 2159

B19_BF1_(AM279338) CTGATGGCCCCGCTCTCCCCCAGAGCCGCCACAGCCCAACCTGGTGCCCATCGTGGCGGG 1725

B21_BF1_(AB426152) CTGATGGCCCCGCTCTCCCCCAGAGCCGCCACAGCCCAACCTGGTGCCCATCGTGGCGGG 2407

B21_BF1_(PacBio_N) CTGATGGCCCCGCTCTCCCCCAGAGCCGCCACAGCCCAACCTGGTGCCCATCGTGGCGGG 2407

B21_BF1_(AM279339) CTGATGGCCCCGCTCTCCCCCAGAGCCGCCACAGCCCAACCTGGTGCCCATCGTGGCGGG 1970

B21_BF1_(PacBio_0) CTGATGGCCCCGCTCTCCCCCAGAGCCGCCACAGCCCAACCTGGTGCCCATCGTGGCGGG 2407

B21_BF1_(AM279342) CTGATGGCCCCGCTCTCCCCCAGAGCCGCCACAGCCCAACCTGGTGCCCATCGTGGCGGG 1970

B2_BF1_(AB426141) GGTGGCCGTCGCCATTGTGGCCAT**T**GCCATCGTGGTTGGTGTTGGATTCATC**C**TCTACAG 2450

B2_BF1_(PacBio_6sub1) GGTGGCCGTCGCCATTGTGGCCAT**T**GCCATCGTGGTTGGTGTTGGATTCATC**C**TCTACAG 2450

B2_BF1_(AM279336) GGTGGCCGTCGCCATTGTGGCCAT**T**GCCATCGTGGTTGGTGTTGGATTCATC**C**TCTACAG 2014

B2_BF1_(PacBio_7sub2) GGTGGCCGTCGCCATTGTGGCCAT**T**GCCATCGTGGTTGGTGTTGGATTCATC**C**TCTACAG 2450

B2_BF1_(AM279340) GGTGGCCGTCGCCATTGTGGCCAT**T**GCCATCGTGGTTGGTGTTGGATTCATC**C**TCTACAG 2014

B4_BF1_(AM279341) GGTGGCCGTCGCCATTGTGGCCATCGCCATCGTGGTTGGTGTTGGATTCATCATCTACAG 2029

B4_BF1_(PacBio_C) GGTGGCCGTCGCCATTGTGGCCATCGCCATCGTGGTTGGTGTTGGATTCATCATCTACAG 2465

B4_BF1_(AM279337) GGTGGCCGTCGCCATTGTGGCCATCGCCATCGTGGTTGGTGTTGGATTCATCATCTACAG 2029

B12_BF1_(AB426147) GGTGGCCGTCGCCATTGTGGCCATCGCCATCGTGGTTGGTGTTGGATTCATCATCTACAG 2222

B12_BF1(PacBio_C) GGTGGCCGTCGCCATTGTGGCCATCGCCATCGTGGTTGGTGTTGGATTCATCATCTACAG 2219

B12_BF1_(AL023516) GGTGGCCGTCGCCATTGTGGCCATCGCCATCGTGGTTGGTGTTGGATTCATCATCTACAG 2222

B14_BF1_(PacBio_WL) ------------------------------------------------------------ 762

B15_BF1_(AB426149) GGTGGCCGTCGCCATTGTGGCCATCGCCATCGTGGTTGGTGTTGGATTCATCATCTACAG 2467

B15_BF1_(PacBio_15I) ------------------------------------------------------------ 762

B19_BF1_(AB426151) GGTGGCCGTCGCCATTGTGGCCATCGCCATCGTGGTTGGTGTTGGATTCATCATCTACAG 2220

B19_BF1_(PacBio_P2a) GGTGGCCGTCGCCATTGTGGCCATCGCCATCGTGGTTGGTGTTGGATTCATCATCTACAG 2219

B19_BF1_(AM279338) GGTGGCCGTCGCCATTGTGGCCATCGCCATCGTGGTTGGTGTTGGATTCATCATCTACAG 1785

B21_BF1_(AB426152) GGTGGCCGTCGCCATTGTGGCCATCGCCATCGTGGTTGGTGTTGGATTCATCATCTACAG 2467

B21_BF1_(PacBio_N) GGTGGCCGTCGCCATTGTGGCCATCGCCATCGTGGTTGGTGTTGGATTCATCATCTACAG 2467

B21_BF1_(AM279339) GGTGGCCGTCGCCATTGTGGCCATCGCCATCGTGGTTGGTGTTGGATTCATCATCTACAG 2030

B21_BF1_(PacBio_0) GGTGGCCGTCGCCATTGTGGCCATCGCCATCGTGGTTGGTGTTGGATTCATCATCTACAG 2467

B21_BF1_(AM279342) GGTGGCCGTCGCCATTGTGGCCATCGCCATCGTGGTTGGTGTTGGATTCATCATCTACAG 2030

Fig. S1

B2_BF1_(AB426141) A**T**GCCA**T**GCAGGTAAAAGCAGAGGGGTGCAGGCGGGC**C**G**---------**TGGGGGGATCTG 2501

B2_BF1_(PacBio_6sub1) A**T**GCCA**T**GCAGGTAAAAGCAGAGGGGTGCAGGCGGGC**C**G**---------**TGGGGGGATCTG 2501

B2_BF1_(AM279336) A**T**GCCA**T**GCAGGTAAAAGCAGAGGGGTGCAGGCGGGC**C**G**---------**TGGGGGGATCTG 2065

B2_BF1_(PacBio_7sub2) A**T**GCCA**T**GCAGGTAAAAGCAGAGGGGTGCAGGCGGGC**C**G**---------**TGGGGGGATCTG 2501

B2_BF1_(AM279340) A**T**GCCA**T**GCAGGTAAAAGCAGAGGGGTGCAGGCGGGC**C**G**---------**TGGGGGGATCTG 2065

B4_BF1_(AM279341) ACGCCACGCAGGTAAAAGCAGAGGGGTGCAGGCGGGCAGTGGTGGCAGTGGGGGGATCTG 2089

B4_BF1_(PacBio_C) ACGCCACGCAGGTAAAAGCAGAGGGGTGCAGGCGGGCAGTGGTGGCAGTGGGGGGATCTG 2525

B4_BF1_(AM279337) ACGCCACGCAGGTAAAAGCAGAGGGGTGCAGGCGGGCAGTGGTGGCAGTGGGGGGATCTG 2089

B12_BF1_(AB426147) ACGCCACGCAGGTAAAAGCAGAGGGGTGCAGGCGGGCAGTGG**G**GGC**T**GT**A**GGGGGATCTG 2282

B12_BF1(PacBio_C) ACGCCACGCAGGTAAAAGCAGAGGGGTGCAGGCGGGCAGTGG**G**GGC**T**GT**A**GGGGGATCTG 2279

B12_BF1_(AL023516) ACGCCACGCAGGTAAAAGCAGAGGGGTGCAGGCGGGCAGTGG**G**GGC**T**GT**A**GGGGGATCTG 2282

B14_BF1_(PacBio_WL) ------------------------------------------------------------ 762

B15_BF1_(AB426149) ACGCCACGCAGGTAAAAGCAGAGGGGTGCAGGCGGGCAGTGGTGGCAGTGGGGGGATCTG 2527

B15_BF1_(PacBio_15I) ------------------------------------------------------------ 762

B19_BF1_(AB426151) ACGCCACGCAGGTAAAAGCAGAGGGGTGCAGGCGGGCAGTGG**G**GGC**T**GT**A**GGGGGATCTG 2280

B19_BF1_(PacBio_P2a) ACGCCACGCAGGTAAAAGCAGAGGGGTGCAGGCGGGCAGTGG**G**GGC**T**GT**A**GGGGGATCTG 2279

B19_BF1_(AM279338) ACGCCACGCAGGTAAAAGCAGAGGGGTGCAGGCGGGCAGTGG**G**GGC**T**GT**A**GGGGGATCTG 1845

B21_BF1_(AB426152) ACGCCACGCAGGTAAAAGCAGAGGGGTGCAGGCGGGCAGTGGTGGCAGTGGGGGGATCTG 2527

B21_BF1_(PacBio_N) ACGCCACGCAGGTAAAAGCAGAGGGGTGCAGGCGGGCAGTGGTGGCAGTGGGGGGATCTG 2527

B21_BF1_(AM279339) ACGCCACGCAGGTAAAAGCAGAGGGGTGCAGGCGGGCAGTGGTGGCAGTGGGGGGATCTG 2090

B21_BF1_(PacBio_0) ACGCCACGCAGGTAAAAGCAGAGGGGTGCAGGCGGGCAGTGGTGGCAGTGGGGGGATCTG 2527

B21_BF1_(AM279342) ACGCCACGCAGGTAAAAGCAGAGGGGTGCAGGCGGGCAGTGGTGGCAGTGGGGGGATCTG 2090

B2_BF1_(AB426141) GGTCCCCCTTGGGAGCCC**C**CA**A**CCTGGCTGTGATGTGAACCTGTG**C**TGAT**G**CATCTCTCT 2561

B2_BF1_(PacBio_6sub1) GGTCCCCCTTGGGAGCCC**C**CA**A**CCTGGCTGTGATGTGAACCTGTG**C**TGAT**G**CATCTCTCT 2561

B2_BF1_(AM279336) GGTCCCCCTTGGGAGCCC**C**CA**A**CCTGGCTGTGATGTGAACCTGTG**C**TGAT**G**CATCTCTCT 2125

B2_BF1_(PacBio_7sub2) GGTCCCCCTTGGGAGCCC**C**CA**A**CCTGGCTGTGATGTGAACCTGTG**C**TGAT**G**CATCTCTCT 2561

B2_BF1_(AM279340) GGTCCCCCTTGGGAGCCC**C**CAACCTGGCTGTGATGTGAACCTGTG**C**TGAT**G**CATCTCTCT 2125

B4_BF1_(AM279341) GGTCCCCCTTGGGAGCCCTCAGCCTGGCTGTGATGTGAACCTGTGTTGATTCATCTCTCT 2149

B4_BF1_(PacBio_C) GGTCCCCCTTGGGAGCCCTCAGCCTGGCTGTGATGTGAACCTGTGTTGATTCATCTCTCT 2585

B4_BF1_(AM279337) GGTCCCCCTTGGGAGCCCTCAGCCTGGCTGTGATGTGAACCTGTGTTGATTCATCTCTCT 2149

B12_BF1_(AB426147) GGTCCCCCTTGGGAGCCC**C**CA**A**CCTGGCTGTGATGTGAACCTGTG**A**TGA**AG**CATCTCTCT 2342

B12_BF1(PacBio_C) GGTCCCCCTTGGGAGCCC**C**CA**A**CCTGGCTGTGATGTGAACCTGTG**A**TGA**AG**CATCTCTCT 2339

B12_BF1_(AL023516) GGTCCCCCTTGGGAGCCC**C**CA**A**CCTGGCTGTGATGTGAACCTGTG**A**TGA**AG**CATCTCTCT 2342

B14_BF1_(PacBio_WL) ------------------------------------------------------------ 762

B15_BF1_(AB426149) GGTCCCCCTTGGGAGCCCTCAGCCTGGCTGTGATGTGAACCTGTGTTGATTCATCTCTCT 2587

B15_BF1_(PacBio_15I) ------------------------------------------------------------ 762

B19_BF1_(AB426151) GGTCCCCCTTGGGAGCCC**C**CA**A**CCTGGCTGTGATGTGAACCTGTG**A**TGA**AG**CATCTCTCT 2340

B19_BF1_(PacBio_P2a) GGTCCCCCTTGGGAGCCC**C**CA**A**CCTGGCTGTGATGTGAACCTGTG**A**TGA**AG**CATCTCTCT 2339

B19_BF1_(AM279338) GGTCCCCCTTGGGAGCCC**C**CA**A**CCTGGCTGTGATGTGAACCTGTG**A**TGA**AG**CATCTCTCT 1905

B21_BF1_(AB426152) GGTCCCCCTTGGGAGCCCTCAGCCTGGCTGTGATGTGAACCTGTGTTGATTCATCTCTCT 2587

B21_BF1_(PacBio_N) GGTCCCCCTTGGGAGCCCTCAGCCTGGCTGTGATGTGAACCTGTGTTGATTCATCTCTCT 2587

B21_BF1_(AM279339) GGTCCCCCTTGGGAGCCCTCAGCCTGGCTGTGATGTGAACCTGTGTTGATTCATCTCTCT 2150

B21_BF1_(PacBio_0) GGTCCCCCTTGGGAGCCCTCAGCCTGGCTGTGATGTGAACCTGTGTTGATTCATCTCTCT 2587

B21_BF1_(AM279342) GGTCCCCCTTGGGAGCCCTCAGCCTGGCTGTGATGTGAACCTGTGTTGATTCATCTCTCT 2150

**BF1 exon 6**

B2_BF1_(AB426141) GTCTGCAGGGAAGAAGGGGAAGGGCTACAACATCGCGCCCGGTGAGTGATGAGGGCAGCG 2621

B2_BF1_(PacBio_6sub1) GTCTGCAGGGAAGAAGGGGAAGGGCTACAACATCGCGCCCGGTGAGTGATGAGGGCAGCG 2621

B2_BF1_(AM279336) GTCTGCAGGGAAGAAGGGGAAGGGCTACAACATCGCGCCCGGTGAGTGATGAGGGCAGCG 2185

B2_BF1_(PacBio_7sub2) GTCTGCAGGGAAGAAGGGGAAGGGCTACAACATCGCGCCCGGTGAGTGATGAGGGCAGCG 2621

B2_BF1_(AM279340) GTCTGCAGGGAAGAAGGGGAAGGGCTACAACATCGCGCCCGGTGAGTGATGAGGGCAGCG 2185

B4_BF1_(AM279341) GTCTGCAGGGAAGAAGGGGAAGGGCTACAACATCGCGCCCGGTGAGTGATGAGGGCAGCG 2209

B4_BF1_(PacBio_C) GTCTGCAGGGAAGAAGGGGAAGGGCTACAACATCGCGCCCGGTGAGTGATGAGGGCAGCG 2645

B4_BF1_(AM279337) GTCTGCAGGGAAGAAGGGGAAGGGCTACAACATCGCGCCCGGTGAGTGATGAGGGCAGCG 2209

B12_BF1_(AB426147) GTCTGCAGGGAAGAAGGGGAAGGGCTACAACATCGCGCCCGGTGAGTGATGAGGGCAGCG 2402

B12_BF1(PacBio_C) GTCTGCAGGGAAGAAGGGGAAGGGCTACAACATCGCGCCCGGTGAGTGATGAGGGCAGCG 2399

B12_BF1_(AL023516) GTCTGCAGGGAAGAAGGGGAAGGGCTACAACATCGCGCCCGGTGAGTGATGAGGGCAGCG 2402

B14_BF1_(PacBio_WL) ------------------------------------------------------------ 762

B15_BF1_(AB426149) GTCTGCAGGGAAGAAGGGGAAGGGCTACAACATCGCGCCCGGTGAGTGATGAGGGCAGCG 2647

B15_BF1_(PacBio_15I) ------------------------------------------------------------ 762

B19_BF1_(AB426151) GTCTGCAGGGAAGAAGGGGAAGGGCTACAACATCGCGCCCGGTGAGTGATGAGGGCAGCG 2400

B19_BF1_(PacBio_P2a) GTCTGCAGGGAAGAAGGGGAAGGGCTACAACATCGCGCCCGGTGAGTGATGAGGGCAGCG 2399

B19_BF1_(AM279338) GTCTGCAGGGAAGAAGGGGAAGGGCTACAACATCGCGCCCGGTGAGTGATGAGGGCAGCG 1965

B21_BF1_(AB426152) GTCTGCAGGGAAGAAGGGGAAGGGCTACAACATCGCGCCCGGTGAGTGATGAGGGCAGCG 2647

B21_BF1_(PacBio_N) GTCTGCAGGGAAGAAGGGGAAGGGCTACAACATCGCGCCCGGTGAGTGATGAGGGCAGCG 2647

B21_BF1_(AM279339) GTCTGCAGGGAAGAAGGGGAAGGGCTACAACATCGCGCCCGGTGAGTGATGAGGGCAGCG 2210

B21_BF1_(PacBio_0) GTCTGCAGGGAAGAAGGGGAAGGGCTACAACATCGCGCCCGGTGAGTGATGAGGGCAGCG 2647

B21_BF1_(AM279342) GTCTGCAGGGAAGAAGGGGAAGGGCTACAACATCGCGCCCGGTGAGTGATGAGGGCAGCG 2210

Fig. S1

B2_BF1_(AB426141) CTGTCCCCCACCTCTGCCCAGTGCCAGGGCGGTCCTGGGGTCT**C**CACTTTCTCCCAGGGT 2681

B2_BF1_(PacBio_6sub1) CTGTCCCCCACCTCTGCCCAGTGCCAGGGCGGTCCTGGGGTCT**C**CACTTTCTCCCAGGGT 2681

B2_BF1_(AM279336) CTGTCCCCCACCTCTGCCCAGTGCCAGGGCGGTCCTGGGGTCT**C**CACTTTCTCCCAGGGT 2245

B2_BF1_(PacBio_7sub2) CTGTCCCCCACCTCTGCCCAGTGCCAGGGCGGTCCTGGGGTCT**C**CACTTTCTCCCAGGGT 2681

B2_BF1_(AM279340) CTGTCCCCCACCTCTGCCCAGTGCCAGGGCGGTCCTGGGGTCT**C**CACTTTCTCCCAGGGT 2245

B4_BF1_(AM279341) CTGTCCCCCACCTCTGCCCAGTGCCAGGGCGGTCCTGGGGTCTGCACTTTCTCCCAGGGT 2269

B4_BF1_(PacBio_C) CTGTCCCCCACCTCTGCCCAGTGCCAGGGCGGTCCTGGGGTCTGCACTTTCTCCCAGGGT 2705

B4_BF1_(AM279337) CTGTCCCCCACCTCTGCCCAGTGCCAGGGCGGTCCTGGGGTCTGCACTTTCTCCCAGGGT 2269

B12_BF1_(AB426147) CTGTCCCCCACCTCTGCCCAGTGCCAGGG**T**GGTCCTGGGGTC**CCTG**CTTTCTCCCAAGGT 2462

B12_BF1(PacBio_C) CTGTCCCCCACCTCTGCCCAGTGCCAGGG**T**GGTCCTGGGGTC**CCTG**CTTTCTCCCAAGGT 2459

B12_BF1_(AL023516) CTGTCCCCCACCTCTGCCCAGTGCCAGGG**T**GGTCCTGGGGTC**CCTG**CTTTCTCCCAAGGT 2462

B14_BF1_(PacBio_WL) ------------------------------------------------------------ 762

B15_BF1_(AB426149) CTGTCCCCCACCTCTGCCCAGTGCCAGGGCGGTCCTGGGGTCTGCACTTTCTCCCAGGGT 2707

B15_BF1_(PacBio_15I) ------------------------------------------------------------ 762

B19_BF1_(AB426151) CTGTCCCCCACCTCTGCCCAGTGCCAGGG**T**GGTCCTGGGGTC**CCTG**CTTTCTCCCAAGGT 2460

B19_BF1_(PacBio_P2a) CTGTCCCCCACCTCTGCCCAGTGCCAGGG**T**GGTCCTGGGGTC**CCTG**CTTTCTCCCAAGGT 2459

B19_BF1_(AM279338) CTGTCCCCCACCTCTGCCCAGTGCCAGGG**T**GGTCCTGGGGTC**CCTG**CTTTCTCCCAAGGT 2025

B21_BF1_(AB426152) CTGTCCCCCACCTCTGCCCAGTGCCAGGGCGGTCCTGGGGTCTGCACTTTCTCCCAGGGT 2707

B21_BF1_(PacBio_N) CTGTCCCCCACCTCTGCCCAGTGCCAGGGCGGTCCTGGGGTCTGCACTTTCTCCCAGGGT 2707

B21_BF1_(AM279339) CTGTCCCCCACCTCTGCCCAGTGCCAGGGCGGTCCTGGGGTCTGCACTTTCTCCCAGGGT 2270

B21_BF1_(PacBio_0) CTGTCCCCCACCTCTGCCCAGTGCCAGGGCGGTCCTGGGGTCTGCACTTTCTCCCAGGGT 2707

B21_BF1_(AM279342) CTGTCCCCCACCTCTGCCCAGTGCCAGGGCGGTCCTGGGGTCTGCACTTTCTCCCAGGGT 2270

B2_BF1_(AB426141) ACCCATTCCTGGTGCTTGGGGCTGCTCCACGCCCCATAGGGAGCACAGGGCTGG**A**TCTCA 2741

B2_BF1_(PacBio_6sub1) ACCCATTCCTGGTGCTTGGGGCTGCTCCACGCCCCATAGGGAGCACAGGGCTGG**A**TCTCA 2741

B2_BF1_(AM279336) ACCCATTCCTGGTGCTTGGGGCTGCTCCACGCCCCATAGGGAGCACAGGGCTGG**A**TCTCA 2305

B2_BF1_(PacBio_7sub2) ACCCATTCCTGGTGCTTGGGGCTGCTCCACGCCCCATAGGGAGCACAGGGCTGG**A**TCTCA 2741

B2_BF1_(AM279340) ACCCATTCCTGGTGCTTGGGGCTGCTCCACGCCCCATAGGGAGCACAGGGCTGG**A**TCTCA 2305

B4_BF1_(AM279341) ACCCATTCCTGGTGCTTGGGGCTGCTCCA**T**GCCCCATAGGGAGCACAGGGCTGGGTCTCA 2329

B4_BF1_(PacBio_C) ACCCATTCCTGGTGCTTGGGGCTGCTCCA**T**GCCCCATAGGGAGCACAGGGCTGGGTCTCA 2765

B4_BF1_(AM279337) ACCCATTCCTGGTGCTTGGGGCTGCTCCA**T**GCCCCATAGGGAGCACAGGGCTGGGTCTCA 2329

B12_BF1_(AB426147) ACCCATTCCTGGTGCTTGGGGCTGCTCCA**T**GCCCCATAGGGAGCACAGGGCTGG**A**TCTCA 2522

B12_BF1(PacBio_C) ACCCATTCCTGGTGCTTGGGGCTGCTCCA**T**GCCCCATAGGGAGCACAGGGCTGG**A**TCTCA 2519

B12_BF1_(AL023516) ACCCATTCCTGGTGCTTGGGGCTGCTCCA**T**GCCCCATAGGGAGCACAGGGCTGG**A**TCTCA 2522

B14_BF1_(PacBio_WL) ------------------------------------------------------------ 762

B15_BF1_(AB426149) ACCCATTCCTGGTGCTTGGGGCTGCTCCACGCCCCATAGGGAGCACAGGGCTGGGTCTCA 2767

B15_BF1_(PacBio_15I) ------------------------------------------------------------ 762

B19_BF1_(AB426151) ACCCATTCCTGGTGCTTGGGGCTGCTCCA**T**GCCCCATAGGGAGCACAGGGCTGG**A**TCTCA 2520

B19_BF1_(PacBio_P2a) ACCCATTCCTGGTGCTTGGGGCTGCTCCA**T**GCCCCATAGGGAGCACAGGGCTGG**A**TCTCA 2519

B19_BF1_(AM279338) ACCCATTCCTGGTGCTTGGGGCTGCTCCA**T**GCCCCATAGGGAGCACAGGGCTGG**A**TCTCA 2085

B21_BF1_(AB426152) ACCCATTCCTGGTGCTTGGGGCTGCTCCACGCCCCATAGGGAGCACAGGGCTGGGTCTCA 2767

B21_BF1_(PacBio_N) ACCCATTCCTGGTGCTTGGGGCTGCTCCACGCCCCATAGGGAGCACAGGGCTGGGTCTCA 2767

B21_BF1_(AM279339) ACCCATTCCTGGTGCTTGGGGCTGCTCCACGCCCCATAGGGAGCACAGGGCTGGGTCTCA 2330

B21_BF1_(PacBio_0) ACCCATTCCTGGTGCTTGGGGCTGCTCCACGCCCCATAGGGAGCACAGGGCTGGGTCTCA 2767

B21_BF1_(AM279342) ACCCATTCCTGGTGCTTGGGGCTGCTCCACGCCCCATAGGGAGCACAGGGCTGGGTCTCA 2330

**BF1 exon 7**

B2_BF1_(AB426141) CAGCTGTTCCTCCCTTATAGACAGGGAAG**A**TGGATCCAGCAGCTCGAGCACAGGTGCGGT 2801

B2_BF1_(PacBio_6sub1) CAGCTGTTCCTCCCTTATAGACAGGGAAG**A**TGGATCCAGCAGCTCGAGCACAGGTGCGGT 2801

B2_BF1_(AM279336) CAGCTGTTCCTCCCTTATAGACAGGGAAG**A**TGGATCCAGCAGCTCGAGCACAGGTGCGGT 2365

B2_BF1_(PacBio_7sub2) CAGCTGTTCCTCCCTTATAGACAGGGAAG**A**TGGATCCAGCAGCTCGAGCACAGGTGCGGT 2801

B2_BF1_(AM279340) CAGCTGTTCCTCCCTTATAGACAGGGAAG**A**TGGATCCAGCAGCTCGAGCACAGGTGCGGT 2365

B4_BF1_(AM279341) CAGCTGTTCCTCCCTTATAGACAGGGAAGGTGGATCCAGCAGCTCGAGCACAGGTGCGGT 2389

B4_BF1_(PacBio_C) CAGCTGTTCCTCCCTTATAGACAGGGAAGGTGGATCCAGCAGCTCGAGCACAGGTGCGGT 2825

B4_BF1_(AM279337) CAGCTGTTCCTCCCTTATAGACAGGGAAGGTGGATCCAGCAGCTCGAGCACAGGTGCGGT 2389

B12_BF1_(AB426147) CAGCTGTTCCTCCCTTATAGACAGGGAAGGTGGATCCAGCAGCTCGAGCACAGGTGCGGT 2582

B12_BF1(PacBio_C) CAGCTGTTCCTCCCTTATAGACAGGGAAGGTGGATCCAGCAGCTCGAGCACAGGTGCGGT 2579

B12_BF1_(AL023516) CAGCTGTTCCTCCCTTATAGACAGGGAAGGTGGATCCAGCAGCTCGAGCACAGGTGCGGT 2582

B14_BF1_(PacBio_WL) ------------------------------------------------------------ 762

B15_BF1_(AB426149) CAGCTGTTCCTCCCTTATAGACAGGGAAGGTGGATCCAGCAGCTCGAGCACAGGTGCGGT 2827

B15_BF1_(PacBio_15I) ------------------------------------------------------------ 762

B19_BF1_(AB426151) CAGCTGTTCCTCCCTTATAGACAGGGAAGGTGGATCCAGCAGCTCGAGCACAGGTGCGGT 2580

B19_BF1_(PacBio_P2a) CAGCTGTTCCTCCCTTATAGACAGGGAAGGTGGATCCAGCAGCTCGAGCACAGGTGCGGT 2579

B19_BF1_(AM279338) CAGCTGTTCCTCCCTTATAGACAGGGAAGGTGGATCCAGCAGCTCGAGCACAGGTGCGGT 2145

B21_BF1_(AB426152) CAGCTGTTCCTCCCTTATAGACAGGGAAGGTGGATCCAGCAGCTCGAGCACAGGTGCGGT 2827

B21_BF1_(PacBio_N) CAGCTGTTCCTCCCTTATAGACAGGGAAGGTGGATCCAGCAGCTCGAGCACAGGTGCGGT 2827

B21_BF1_(AM279339) CAGCTGTTCCTCCCTTATAGACAGGGAAGGTGGATCCAGCAGCTCGAGCACAGGTGCGGT 2390

B21_BF1_(PacBio_0) CAGCTGTTCCTCCCTTATAGACAGGGAAGGTGGATCCAGCAGCTCGAGCACAGGTGCGGT 2827

B21_BF1_(AM279342) CAGCTGTTCCTCCCTTATAGACAGGGAAGGTGGATCCAGCAGCTCGAGCACAGGTGCGGT 2390

Fig. S1

B2_BF1_(AB426141) GTGGGGCTGTGGGTTGGGAGGGGTCCGTGTGCTCTCTGTGGTACTGCCCAGGGCTGGGCT 2861

B2_BF1_(PacBio_6sub1) GTGGGGCTGTGGGTTGGGAGGGGTCCGTGTGCTCTCTGTGGTACTGCCCAGGGCTGGGCT 2861

B2_BF1_(AM279336) GTGGGGCTGTGGGTTG-GAGGGGTCCGTGTGCTCTCTGTGGTACTGCCCAGGGCTGGGCT 2424

B2_BF1_(PacBio_7sub2) GTGGGGCTGTGGGTTGGGAGGGGTCCGTGTGCTCTCTGTGGTACTGCCCAGGGCTGGGCT 2861

B2_BF1_(AM279340) GTGGGGCTGTGGGTTG-GAGGGGTCCGTGTGCTCTCTGTGGTACTGCCCAGGGCTGGGCT 2424

B4_BF1_(AM279341) GTGGGGCTGTGGGTTGGGAGGGGTCCGTGTGCTCTCTGTGGTACTGCCCAGGGCTGGGCT 2449

B4_BF1_(PacBio_C) GTGGGGCTGTGGGTTGGGAGGGGTCCGTGTGCTCTCTGTGGTACTGCCCAGGGCTGGGCT 2885

B4_BF1_(AM279337) GTGGGGCTGTGGGTTGGGAGGGGTCCGTGTGCTCTCTGTGGTACTGCCCAGGGCTGGGCT 2449

B12_BF1_(AB426147) GTGGGGCTGTGGGTTGGGAGGGGTCCGTGTGCTCTCTGTGGTACTGCCCAGGGCTGGGCT 2642

B12_BF1(PacBio_C) GTGGGGCTGTGGGTTGGGAGGGGTCCGTGTGCTCTCTGTGGTACTGCCCAGGGCTGGGCT 2639

B12_BF1_(AL023516) GTGGGGCTGTGGGTTGGGAGGGGTCCGTGTGCTCTCTGTGGTACTGCCCAGGGCTGGGCT 2642

B14_BF1_(PacBio_WL) ------------------------------------------------------------ 762

B15_BF1_(AB426149) GTGGGGCTGTGGGTTGGGAGGGGTCCGTGTGCTCTCTGTGGTACTGCCCAGGGCTGGGCT 2887

B15_BF1_(PacBio_15I) ------------------------------------------------------------ 762

B19_BF1_(AB426151) GTGGGGCTGTGGGTTGGGAGGGGTCCGTGTGCTCTCTGTGGTACTGCCCAGGGCTGGGCT 2640

B19_BF1_(PacBio_P2a) GTGGGGCTGTGGGTTGGGAGGGGTCCGTGTGCTCTCTGTGGTACTGCCCAGGGCTGGGCT 2639

B19_BF1_(AM279338) GTGGGGCTGTGGGTTGGGAGGGGTCCGTGTGCTCTCTGTGGTACTGCCCAGGGCTGGGCT 2205

B21_BF1_(AB426152) GTGGGGCTGTGGGTTGGGAGGGGTCCGTGTGCTCTCTGTGGTACTGCCCAGGGCTGGGCT 2887

B21_BF1_(PacBio_N) GTGGGGCTGTGGGTTGGGAGGGGTCCGTGTGCTCTCTGTGGTACTGCCCAGGGCTGGGCT 2887

B21_BF1_(AM279339) GTGGGGCTGTGGGTTGGGAGGGGTCCGTGTGCTCTCTGTGGTACTGCCCAGGGCTGGGCT 2450

B21_BF1_(PacBio_0) GTGGGGCTGTGGGTTGGGAGGGGTCCGTGTGCTCTCTGTGGTACTGCCCAGGGCTGGGCT 2887

B21_BF1_(AM279342) GTGGGGCTGTGGGTTGGGAGGGGTCCGTGTGCTCTCTGTGGTACTGCCCAGGGCTGGGCT 2450

B2_BF1_(AB426141) ATGCTGGGGCTCTGCGGGGAGACCCC**T**GGAGCAGAGGGTTGGGATGTGAAC**C**TG**-**GCCCC 2920

B2_BF1_(PacBio_6sub1) ATGCTGGGGCTCTGCGGGGAGACCCC**T**GGAGCAGAGGGTTGGGATGTGAAC**C**TG**-**GCCCC 2920

B2_BF1_(AM279336) ATGCTGGGGCTCTGCGGGGAGACCCC**T**GGAGCAGAGGGTTGGGATGTGAAC**C**TG**-**GCCCC 2483

B2_BF1_(PacBio_7sub2) ATGCTGGGGCTCTGCGGGGAGACCCC**T**GGAGCAGAGGGTTGGGATGTGAAC**C**TG**-**GCCCC 2920

B2_BF1_(AM279340) ATGCTGGGGCTCTGCGGGGAGACCCC**T**GGAGCAGAGGGTTGGGATGTGAAC**C**TG**-**GCCCC 2483

B4_BF1_(AM279341) ATGCTGGGGCTCTGCGGGGAGACCCCCGGAGCAGAGGGTTGGGATGTGAACATGGGCCCC 2509

B4_BF1_(PacBio_C) ATGCTGGGGCTCTGCGGGGAGACCCCCGGAGCAGAGGGTTGGGATGTGAACATGGGCCCC 2945

B4_BF1_(AM279337) ATGCTGGGGCTCTGCGGGGAGACCCCCGGAGCAGAGGGTTGGGATGTGAACATGGGCCCC 2509

B12_BF1_(AB426147) ATGCTGGGGCTCTGCGGGGAGACCCCCGGAGCAGAGGGTTGGGATGTGAAC**C**TG**-**GCCCC 2701

B12_BF1(PacBio_C) ATGCTGGGGCTCTGCGGGGAGACCCCCGGAGCAGAGGGTTGGGATGTGAAC**C**TG**-**GCCCC 2698

B12_BF1_(AL023516) ATGCTGGGGCTCTGCGGGGAGACCCCCGGAGCAGAGGGTTGGGATGTGAAC**C**TG**-**GCCCC 2701

B14_BF1_(PacBio_WL) ------------------------------------------------------------ 762

B15_BF1_(AB426149) ATGCTGGGGCTCTGCGGGGAGACCCCCGGAGCAGAGGGTTGGGATGTGAACATGGGCCCC 2947

B15_BF1_(PacBio_15I) ------------------------------------------------------------ 762

B19_BF1_(AB426151) ATGCTGGGGCTCTGCGGGGAGACCCCCGGAGCAGAGGGTTGGGATGTGAAC**C**TG**-**GCCCC 2699

B19_BF1_(PacBio_P2a) ATGCTGGGGCTCTGCGGGGAGACCCCCGGAGCAGAGGGTTGGGATGTGAAC**C**TG**-**GCCCC 2698

B19_BF1_(AM279338) ATGCTGGGGCTCTGCGGGGAGACCCCCGGAGCAGAGGGTTGGGATGTGAAC**C**TG**-**GCCCC 2264

B21_BF1_(AB426152) ATGCTGGGGCTCTGCGGGGAGACCCCCGGAGCAGAGGGTTGGGATGTGAACATGGGCCCC 2947

B21_BF1_(PacBio_N) ATGCTGGGGCTCTGCGGGGAGACCCCCGGAGCAGAGGGTTGGGATGTGAACATGGGCCCC 2947

B21_BF1_(AM279339) ATGCTGGGGCTCTGCGGGGAGACCCCCGGAGCAGAGGGTTGGGATGTGAACATGGGCCCC 2510

B21_BF1_(PacBio_0) ATGCTGGGGCTCTGCGGGGAGACCCCCGGAGCAGAGGGTTGGGATGTGAACATGGGCCCC 2947

B21_BF1_(AM279342) ATGCTGGGGCTCTGCGGGGAGACCCCCGGAGCAGAGGGTTGGGATGTGAACATGGGCCCC 2510

**BF1 exon 8 BF1 stop**

B2_BF1_(AB426141) GTGGGACA**T**CATC**C**CTTCTCATCCCCACAGGGAGCAACCCCTCCATCTGAGTGCTGTGCT 2980

B2_BF1_(PacBio_6sub1) GTGGGACA**T**CATC**C**CTTCTCATCCCCACAGGGAGCAACCCCTCCATCTGAGTGCTGTGCT 2980

B2_BF1_(AM279336) GTGGGACA**T**CATC**C**CTTCTCATCCCCACAGGGAGCAACCCCTCCATCTGAGTGCTGTGCT 2543

B2_BF1_(PacBio_7sub2) GTGGGACA**T**CATC**C**CTTCTCATCCCCACAGGGAGCAACCCCTCCATCTGAGTGCTGTGCT 2980

B2_BF1_(AM279340) GTGGGACA**T**CATC**C**CTTCTCATCCCCACAGGGAGCAACCCCTCCATCTGAGTGCTGTGCT 2543

B4_BF1_(AM279341) GTGGGACACCATCTCTTCTCATCCCCACAGGGAGCAACCCCTCCATCTGAGTGCTGTGCT 2569

B4_BF1_(PacBio_C) GTGGGACACCATCTCTTCTCATCCCCACAGGGAGCAACCCCTCCATCTGAGTGCTGTGCT 3005

B4_BF1_(AM279337) GTGGGACACCATCTCTTCTCATCCCCACAGGGAGCAACCCCTCCATCTGAGTGCTGTGCT 2569

B12_BF1_(AB426147) GTGGGACA**T**CATC**C**CTTCTCATCCCCACAGGGAGCAACCCC**G**CCATCTGAGTGCTGTGCT 2761

B12_BF1(PacBio_C) GTGGGACA**T**CATC**C**CTTCTCATCCCCACAGGGAGCAACCCC**G**CCATCTGAGTGCTGTGCT 2758

B12_BF1_(AL023516) GTGGGACA**T**CATC**C**CTTCTCATCCCCACAGGGAGCAACCCC**G**CCATCTGAGTGCTGTGCT 2761

B14_BF1_(PacBio_WL) ------------------------------------------------------------ 762

B15_BF1_(AB426149) GTGGGACACCATCTCTTCTCATCCCCACAGGGAGCAACCCCTCCATCTGAGTGCTGTGCT 3007

B15_BF1_(PacBio_15I) ------------------------------------------------------------ 762

B19_BF1_(AB426151) GTGGGACA**T**CATC**C**CTTCTCATCCCCACAGGGAGCAACCCC**G**CCATCTGAGTGCTGTGCT 2759

B19_BF1_(PacBio_P2a) GTGGGACA**T**CATC**C**CTTCTCATCCCCACAGGGAGCAACCCC**G**CCATCTGAGTGCTGTGCT 2758

B19_BF1_(AM279338) GTGGGACA**T**CATC**C**CTTCTCATCCCCACAGGGAGCAACCCC**G**CCATCTGAGTGCTGTGCT 2324

B21_BF1_(AB426152) GTGGGACACCATCTCTTCTCATCCCCACAGGGAGCAACCCCTCCATCTGAGTGCTGTGCT 3007

B21_BF1_(PacBio_N) GTGGGACACCATCTCTTCTCATCCCCACAGGGAGCAACCCCTCCATCTGAGTGCTGTGCT 3007

B21_BF1_(AM279339) GTGGGACACCATCTCTTCTCATCCCCACAGGGAGCAACCCCTCCATCTGAGTGCTGTGCT 2570

B21_BF1_(PacBio_0) GTGGGACACCATCTCTTCTCATCCCCACAGGGAGCAACCCCTCCATCTGAGTGCTGTGCT 3007

B21_BF1_(AM279342) GTGGGACACCATCTCTTCTCATCCCCACAGGGAGCAACCCCTCCATCTGAGTGCTGTGCT 2570

Fig. S1

B2_BF1_(AB426141) TCAGC**C**TGCA**A**G**G**AGCCAACAGTCCACACCAGCATTTGGGGTC**G**GTGATGGGCACAGCCC 3040

B2_BF1_(PacBio_6sub1) TCAGC**C**TGCA**A**G**G**AGCCAACAGTCCACACCAGCATTTGGGGTC**G**GTGATGGGCACAGCCC 3040

B2_BF1_(AM279336) TCAGC**C**TGCA**A**G**G**AGCCAACAGTCCACACCAGCATTTGGGGTC**G**GTGATGGGCACAGCCC 2603

B2_BF1_(PacBio_7sub2) TCAGC**C**TGCA**A**G**G**AGCCAACAGTCCACACCAGCATTTGGGGTC**G**GTGATGGGCACAGCCC 3040

B2_BF1_(AM279340) TCAGC**C**TGCA**A**G**G**AGCCAACAGTCCACACCAGCATTTGGGGTC**G**GTGATGGGCACAGCCC 2603

B4_BF1_(AM279341) TCAGCATGCACGAAGCCAACAGTCCACACCAGCATTTGGGGTCAGTGATGGGCACAGCCC 2629

B4_BF1_(PacBio_C) TCAGCATGCACGAAGCCAACAGTCCACACCAGCATTTGGGGTCAGTGATGGGCACAGCCC 3065

B4_BF1_(AM279337) TCAGCATGCACGAAGCCAACAGTCCACACCAGCATTTGGGGTCAGTGATGGGCACAGCCC 2629

B12_BF1_(AB426147) TCAGC**C**TGCA**A**G**G**AGCCAACAGTCCACACCAGCATTTGGGGTC**G**GTGATGG**A**CACAGCCC 2821

B12_BF1(PacBio_C) TCAGC**C**TGCA**A**G**G**AGCCAACAGTCCACACCAGCATTTGGGGTC**G**GTGATGG**A**CACAGCCC 2818

B12_BF1_(AL023516) TCAGC**C**TGCA**A**G**G**AGCCAACAGTCCACACCAGCATTTGGGGTC**G**GTGATGG**A**CACAGCCC 2821

B14_BF1_(PacBio_WL) ------------------------------------------------------------ 762

B15_BF1_(AB426149) TCAGCATGCACGAAGCCAACAGTCCACACCAGCATTTGGGGTCAGTGATGGGCACAGCCC 3067

B15_BF1_(PacBio_15I) ------------------------------------------------------------ 762

B19_BF1_(AB426151) TCAGC**C**TGCA**A**G**G**AGCCAACAGTCCACACCAGCATTTGGGGTC**G**GTGATGG**A**CACAGCCC 2819

B19_BF1_(PacBio_P2a) TCAGC**C**TGCA**A**G**G**AGCCAACAGTCCACACCAGCATTTGGGGTC**G**GTGATGG**A**CACAGCCC 2818

B19_BF1_(AM279338) TCAGC**C**TGCA**A**G**G**AGCCAACAGTCCACACCAGCATTTGGGGTC**G**GTGATGG**A**CACAGCCC 2384

B21_BF1_(AB426152) TCAGCATGCACGAAGCCAACAGTCCACACCAGCATTTGGGGTCAGTGATGGGCACAGCCC 3067

B21_BF1_(PacBio_N) TCAGCATGCACGAAGCCAACAGTCCACACCAGCATTTGGGGTCAGTGATGGGCACAGCCC 3067

B21_BF1_(AM279339) TCAGCATGCACGAAGCCAACAGTCCACACCAGCATTTGGGGTCAGTGATGGGCACAGCCC 2630

B21_BF1_(PacBio_0) TCAGCATGCACGAAGCCAACAGTCCACACCAGCATTTGGGGTCAGTGATGGGCACAGCCC 3067

B21_BF1_(AM279342) TCAGCATGCACGAAGCCAACAGTCCACACCAGCATTTGGGGTCAGTGATGGGCACAGCCC 2630

B2_BF1_(AB426141) CATCCTCTTGACCTCTCACATCTCATTCTGCTTCCTATGCTGACTGTTATGCTTTGCCTG 3100

B2_BF1_(PacBio_6sub1) CATCCTCTTGACCTCTCACATCTCATTCTGCTTCCTATGCTGACTGTTATGCTTTGCCTG 3100

B2_BF1_(AM279336) CATCCTCTTGACCTCTCACATCTCATTCTGCTTCCTATGCTGACTGTTATGCTTTGCCTG 2663

B2_BF1_(PacBio_7sub2) CATCCTCTTGACCTCTCACATCTCATTCTGCTTCCTATGCTGACTGTTATGCTTTGCCTG 3100

B2_BF1_(AM279340) CATCCTCTTGACCTCTCACATCTCATTCTGCTTCCTATGCTGACTGTTATGCTTTGCCTG 2663

B4_BF1_(AM279341) CATCCTCTTGACCTCTCACATCTCATTCTGCTTCCTATGCTGACTGTTATGCTTTGCCTG 2689

B4_BF1_(PacBio_C) CATCCTCTTGACCTCTCACATCTCATTCTGCTTCCTATGCTGACTGTTATGCTTTGCCTG 3125

B4_BF1_(AM279337) CATCCTCTTGACCTCTCACATCTCATTCTGCTTCCTATGCTGACTGTTATGCTTTGCCTG 2689

B12_BF1_(AB426147) CATCCTC**C**TGACCTCTCACATCTCATTCTGCTTCCTATGCTGACTGTTATGCTTTGCCTG 2881

B12_BF1(PacBio_C) CATCCTC**C**TGACCTCTCACATCTCATTCTGCTTCCTATGCTGACTGTTATGCTTTGCCTG 2878

B12_BF1_(AL023516) CATCCTC**C**TGACCTCTCACATCTCATTCTGCTTCCTATGCTGACTGTTATGCTTTGCCTG 2881

B14_BF1_(PacBio_WL) ------------------------------------------------------------ 762

B15_BF1_(AB426149) CATCCTCTTGACCTCTCACATCTC**G**TTCTGCTTCCTATGCTGACTGTTATGCTTTGCCTG 3127

B15_BF1_(PacBio_15I) ------------------------------------------------------------ 762

B19_BF1_(AB426151) CATCCTC**C**TGACCTCTCACATCTCATTCTGCTTCCTATGCTGACTGTTATGCTTTGCCTG 2879

B19_BF1_(PacBio_P2a) CATCCTC**C**TGACCTCTCACATCTCATTCTGCTTCCTATGCTGACTGTTATGCTTTGCCTG 2878

B19_BF1_(AM279338) CATCCTC**C**TGACCTCTCACATCTCATTCTGCTTCCTATGCTGACTGTTATGCTTTGCCTG 2444

B21_BF1_(AB426152) CATCCTCTTGACCTCTCACATCTC**G**TTCTGCTTCCTATGCTGACTGTTATGCTTTGCCTG 3127

B21_BF1_(PacBio_N) CATCCTCTTGACCTCTCACATCTC**G**TTCTGCTTCCTATGCTGACTGTTATGCTTTGCCTG 3127

B21_BF1_(AM279339) CATCCTCTTGACCTCTCACATCTC**G**TTCTGCTTCCTATGCTGACTGTTATGCTTTGCCTG 2690

B21_BF1_(PacBio_0) CATCCTCTTGACCTCTCACATCTC**G**TTCTGCTTCCTATGCTGACTGTTATGCTTTGCCTG 3127

B21_BF1_(AM279342) CATCCTCTTGACCTCTCACATCTC**G**TTCTGCTTCCTATGCTGACTGTTATGCTTTGCCTG 2690

**BF1 PolyA site 1**

B2_BF1_(AB426141) CACTGCT**T**CCTGTGAAATA**C**AATGATGGG**A**CATTCTGT**G**CTCAGCTTGCCT**G**CATTCTGC 3160

B2_BF1_(PacBio_6sub1) CACTGCT**T**CCTGTGAAATA**C**AATGATGGG**A**CATTCTGT**G**CTCAGCTTGCCT**G**CATTCTGC 3160

B2_BF1_(AM279336) CACTGCT**T**CCTGTGAAATA**C**AATGATGGG**A**CATTCTGT**G**CTCAGCTTGCCT**G**CATTCTGC 2723

B2_BF1_(PacBio_7sub2) CACTGCT**T**CCTGTGAAATA**C**AATGATGGG**A**CATTCTGT**G**CTCAGCTTGCCT**G**CATTCTGC 3160

B2_BF1_(AM279340) CACTGCT**T**CCTGTGAAATA**C**AATGATGGGACATTCTGT**G**CTCAGCTTGCCT**G**CATTCTGC 2723

B4_BF1_(AM279341) CACTGCTCCCTGTGAAATAAAATGATGGGCCATTCTGT-CTCAGCTTGCCTTCATTCTGC 2748

B4_BF1_(PacBio_C) CACTGCTCCCTGTGAAATAAAATGATGGGCCATTCTGT-CTCAGCTTGCCTTCATTCTGC 3184

B4_BF1_(AM279337) CACTGCTCCCTGTGAAATAAAATGATGGGCCATTCTGT-CTCAGCTTGCCTTCATTCTGC 2748

B12_BF1_(AB426147) CACTGCT**T**CCTGTGAAATAAAATGATGGGCCATTCTGT**G**CTCAGCTTGCCT**G**CATTCTGC 2941

B12_BF1(PacBio_C) CACTGCT**T**CCTGTGAAATAAAATGATGGGCCATTCTGT**G**CTCAGCTTGCCT**G**CATTCTGC 2938

B12_BF1_(AL023516) CACTGCT**T**CCTGTGAAATAAAATGATGGGCCATTCTGT**G**CTCAGCTTGCCT**G**CATTCTGC 2941

B14_BF1_(PacBio_WL) ------------------------------------------------------------ 762

B15_BF1_(AB426149) CACTGCTCCCTGTGAAATAAAATGATGGGCCATTCTGT-CTCAGCTTGCCTTCATTCTGC 3186

B15_BF1_(PacBio_15I) ------------------------------------------------------------ 762

B19_BF1_(AB426151) CACTGCT**T**CCTGTGAAATAAAATGATGGGCCATTCTGT**G**CTCAGCTTGCCT**G**CATTCTGC 2939

B19_BF1_(PacBio_P2a) CACTGCT**T**CCTGTGAAATAAAATGATGGGCCATTCTGT**G**CTCAGCTTGCCT**G**CATTCTGC 2938

B19_BF1_(AM279338) CACTGCT**T**CCTGTGAAATAAAATGATGGGCCATTCTGT**G**CTCAGCTTGCCT**G**CATTCTGC 2504

B21_BF1_(AB426152) CACTGCTCCCTGTGAAATAAAATGATGGGCCATTCTGT-CTCAGCTTGCCTTCATTCTGC 3186

B21_BF1_(PacBio_N) CACTGCTCCCTGTGAAATAAAATGATGGGCCATTCTGT-CTCAGCTTGCCTTCATTCTGC 3186

B21_BF1_(AM279339) CACTGCTCCCTGTGAAATAAAATGATGGGCCATTCTGT-CTCAGCTTGCCTTCATTCTGC 2749

B21_BF1_(PacBio_0) CACTGCTCCCTGTGAAATAAAATGATGGGCCATTCTGT-CTCAGCTTGCCTTCATTCTGC 3186

B21_BF1_(AM279342) CACTGCTCCCTGTGAAATAAAATGATGGGCCATTCTGT-CTCAGCTTGCCTTCATTCTGC 2749

Fig. S1

B2_BF1_(AB426141) ACTGTGCTGTGGTTGGGGATGGGGTGGGTGAGGGGACCGTGTCCCAGTTTGGCTGCTCAG 3220

B2_BF1_(PacBio_6sub1) ACTGTGCTGTGGTTGGGGATGGGGTGGGTGAGGGGACCGTGTCCCAGTTTGGCTGCTCAG 3220

B2_BF1_(AM279336) ACTGTGCTGTGGTTGGGGATGGGGTGGGTGAGGGGACCGTGTCCCAGTTTGGCTGCTCAG 2783

B2_BF1_(PacBio_7sub2) ACTGTGCTGTGGTTGGGGATGGGGTGGGTGAGGGGACCGTGTCCCAGTTTGGCTGCTCAG 3220

B2_BF1_(AM279340) ACTGTGCTGTGGTTGGGGATGGGGTGGGTGAGGGGACCGTGTCCCAGTTTGGCTGCTCAG 2783

B4_BF1_(AM279341) ACTGTGCTGTGGTTGGGGATGGGGTGGGTGAGGGGACCGTGTCCCAGTTTGGCTGCTCAG 2808

B4_BF1_(PacBio_C) ACTGTGCTGTGGTTGGGGATGGGGTGGGTGAGGGGACCGTGTCCCAGTTTGGCTGCTCAG 3244

B4_BF1_(AM279337) ACTGTGCTGTGGTTGGGGATGGGGTGGGTGAGGGGACCGTGTCCCAGTTTGGCTGCTCAG 2808

B12_BF1_(AB426147) ACTGTGCTGTGGTTGGGGATGGGGTGGGTGAG**A**GGACCGTGTCCCAGTTTGGCTGCTCAG 3001

B12_BF1(PacBio_C) ACTGTGCTGTGGTTGGGGATGGGGTGGGTGAG**A**GGACCGTGTCCCAGTTTGGCTGCTCAG 2998

B12_BF1_(AL023516) ACTGTGCTGTGGTTGGGGATGGGGTGGGTGAG**A**GGACCGTGTCCCAGTTTGGCTGCTCAG 3001

B14_BF1_(PacBio_WL) ------------------------------------------------------------ 762

B15_BF1_(AB426149) ACTGTGCTGTGGTTGGGGATGGGGTGGGTGAGGGGACCGTGTCCCAGTTTGGCTGCTCAG 3246

B15_BF1_(PacBio_15I) ------------------------------------------------------------ 762

B19_BF1_(AB426151) ACTGTGCTGTGGTTGGGGATGGGGTGGGTGAG**A**GGACCGTGTCCCAGTTTGGCTGCTCAG 2999

B19_BF1_(PacBio_P2a) ACTGTGCTGTGGTTGGGGATGGGGTGGGTGAG**A**GGACCGTGTCCCAGTTTGGCTGCTCAG 2998

B19_BF1_(AM279338) ACTGTGCTGTGGTTGGGGATGGGGTGGGTGAG**A**GGACCGTGTCCCAGTTTGGCTGCTCAG 2564

B21_BF1_(AB426152) ACTGTGCTGTGGTTGGGGATGGGGTGGGTGAGGGGACCGTGTCCCAGTTTGGCTGCTCAG 3246

B21_BF1_(PacBio_N) ACTGTGCTGTGGTTGGGGATGGGGTGGGTGAGGGGACCGTGTCCCAGTTTGGCTGCTCAG 3246

B21_BF1_(AM279339) ACTGTGCTGTGGTTGGGGATGGGGTGGGTGAGGGGACCGTGTCCCAGTTTGGCTGCTCAG 2809

B21_BF1_(PacBio_0) ACTGTGCTGTGGTTGGGGATGGGGTGGGTGAGGGGACCGTGTCCCAGTTTGGCTGCTCAG 3246

B21_BF1_(AM279342) ACTGTGCTGTGGTTGGGGATGGGGTGGGTGAGGGGACCGTGTCCCAGTTTGGCTGCTCAG 2809

B2_BF1_(AB426141) GGTGCAG**A**TGTGGCCCTGTGCTGAGTACCCACAGCCCTC**C**CCCCCTATCTGCCTGCTGCT 3280

B2_BF1_(PacBio_6sub1) GGTGCAG**A**TGTGGCCCTGTGCTGAGTACCCACAGCCCTC**C**CCCCCTATCTGCCTGCTGCT 3280

B2_BF1_(AM279336) GGTGCAG**A**TGTGGCCCTGTGCTGAGTACCCACAGCCCTC**C**CCCCCTATCTGCCTGCTGCT 2843

B2_BF1_(PacBio_7sub2) GGTGCAG**A**TGTGGCCCTGTGCTGAGTACCCACAGCCCTC**C**CCCCCTATCTGCCTGCTGCT 3280

B2_BF1_(AM279340) GGTGCAG**A**TGTGGCCCTGTGCTGAGTACCCACAGCCCTC**C**CCCCCTATCTGCCTGCTGCT 2843

B4_BF1_(AM279341) GGTGCAGGTGTGGCCCTGTGCTGAGTACCCACAGCCCTCTCCCCCTATCTGCCTGCTGCT 2868

B4_BF1_(PacBio_C) GGTGCAGGTGTGGCCCTGTGCTGAGTACCCACAGCCCTCTCCCCCTATCTGCCTGCTGCT 3304

B4_BF1_(AM279337) GGTGCAGGTGTGGCCCTGTGCTGAGTACCCACAGCCCTCTCCCCCTATCTGCCTGCTGCT 2868

B12_BF1_(AB426147) GGTGCAG**A**TGTGGCCCTGTGCTGAGTACCCACAGCCCTC**C**CCCCCTATCTGCCTGCTGCT 3061

B12_BF1(PacBio_C) GGTGCAG**A**TGTGGCCCTGTGCTGAGTACCCACAGCCCTC**C**CCCCCTATCTGCCTGCTGCT 3058

B12_BF1_(AL023516) GGTGCAG**A**TGTGGCCCTGTGCTGAGTACCCACAGCCCTC**C**CCCCCTATCTGCCTGCTGCT 3061

B14_BF1_(PacBio_WL) ------------------------------------------------------------ 762

B15_BF1_(AB426149) GGTGCAGGTGTGGCCCTGTGCTGAGTACCCACAGCCCTCTCCCCCTATCTGCCTGCTGCT 3306

B15_BF1_(PacBio_15I) ------------------------------------------------------------ 762

B19_BF1_(AB426151) GGTGCAG**A**TGTGGCCCTGTGCTGAGTACCCACAGCCCTC**C**CCCCCTATCTGCCTGCTGCT 3059

B19_BF1_(PacBio_P2a) GGTGCAG**A**TGTGGCCCTGTGCTGAGTACCCACAGCCCTC**C**CCCCCTATCTGCCTGCTGCT 3058

B19_BF1_(AM279338) GGTGCAG**A**TGTGGCCCTGTGCTGAGTACCCACAGCCCTC**C**CCCCCTATCTGCCTGCTGCT 2624

B21_BF1_(AB426152) GGTGCAGGTGTGGCCCTGTGCTGAGTACCCACAGCCCTCTCCCCCTATCTGCCTGCTGCT 3306

B21_BF1_(PacBio_N) GGTGCAGGTGTGGCCCTGTGCTGAGTACCCACAGCCCTCTCCCCCTATCTGCCTGCTGCT 3306

B21_BF1_(AM279339) GGTGCAGGTGTGGCCCTGTGCTGAGTACCCACAGCCCTCTCCCCCTATCTGCCTGCTGCT 2869

B21_BF1_(PacBio_0) GGTGCAGGTGTGGCCCTGTGCTGAGTACCCACAGCCCTCTCCCCCTATCTGCCTGCTGCT 3306

B21_BF1_(AM279342) GGTGCAGGTGTGGCCCTGTGCTGAGTACCCACAGCCCTCTCCCCCTATCTGCCTGCTGCT 2869

B2_BF1_(AB426141) CACTCCCCCCT**G**TGTACCCCC**A**T**C**CCTTCTCACCTCTCCTCTGTGACCCCATGCTGGTGG 3340

B2_BF1_(PacBio_6sub1) CACTCCCCCCT**G**TGTACCCCC**A**T**C**CCTTCTCACCTCTCCTCTGTGACCCCATGCTGGTGG 3340

B2_BF1_(AM279336) CACTCCCCCCT**G**TGTACCCCC**A**T**C**CCTTCTCACCTCTCCTCTGTGACCCCATGCTGGTGG 2903

B2_BF1_(PacBio_7sub2) CACTCCCCCCT**G**TGTACCCCC**A**T**C**CCTTCTCACCTCTCCTCTGTGACCCCATGCTGGTGG 3340

B2_BF1_(AM279340) CACTCCCCCCT**G**TGTACCCCC**A**T**C**CCTTCTCACCTCTCCTCTGTGACCCCATGCTGGTGG 2903

B4_BF1_(AM279341) CACTCCCCCCT**G**TGTACCCCCGTTCCTTCTCACCTCTCCTCTGTGACCCCATGCTGGTGG 2928

B4_BF1_(PacBio_C) CACTCCCCCCT**G**TGTACCCCCGTTCCTTCTCACCTCTCCTCTGTGACCCCATGCTGGTGG 3364

B4_BF1_(AM279337) CACTCCCCCCT**G**TGTACCCCCGTTCCTTCTCACCTCTCCTCTGTGACCCCATGCTGGTGG 2928

B12_BF1_(AB426147) CACTCCCCC**T**TCTGTACCCCC**A**T**C**CCTTCTCACCTCTCCTCTGTGACCCCATGCTGGTGG 3121

B12_BF1(PacBio_C) CACTCCCCC**T**TCTGTACCCCC**A**T**C**CCTTCTCACCTCTCCTCTGTGACCCCATGCTGGTGG 3118

B12_BF1_(AL023516) CACTCCCCC**T**TCTGTACCCCC**A**T**C**CCTTCTCACCTCTCCTCTGTGACCCCATGCTGGTGG 3121

B14_BF1_(PacBio_WL) ------------------------------------------------------------ 762

B15_BF1_(AB426149) CACTCCCCCCTCTGTACCCCCGTTCCTTCTCACCTCTCCTCTGTGACCCCATGCTGGTGG 3366

B15_BF1_(PacBio_15I) ------------------------------------------------------------ 762

B19_BF1_(AB426151) CACTCCCCC**T**TCTGTACCCCC**A**T**C**CCTTCTCACCTCTCCTCTGTGACCCCATGCTGGTGG 3119

B19_BF1_(PacBio_P2a) CACTCCCCC**T**TCTGTACCCCC**A**T**C**CCTTCTCACCTCTCCTCTGTGACCCCATGCTGGTGG 3118

B19_BF1_(AM279338) CACTCCCCC**T**TCTGTACCCCC**A**T**C**CCTTCTCACCTCTCCTCTGTGACCCCATGCTGGTGG 2684

B21_BF1_(AB426152) CACTCCCCCCTCTGTACCCCCGTTCCTTCTCACCTCTCCTCTGTGACCCCATGCTGGTGG 3366

B21_BF1_(PacBio_N) CACTCCCCCCTCTGTACCCCCGTTCCTTCTCACCTCTCCTCTGTGACCCCATGCTGGTGG 3366

B21_BF1_(AM279339) CACTCCCCCCTCTGTACCCCCGTTCCTTCTCACCCCTCCTCTGTGACCCCATGCTGGTGG 2929

B21_BF1_(PacBio_0) CACTCCCCCCTCTGTACCCCCGTTCCTTCTCACCTCTCCTCTGTGACCCCATGCTGGTGG 3366

B21_BF1_(AM279342) CACTCCCCCCTCTGTACCCCCGTTCCTTCTCACCCCTCCTCTGTGACCCCATGCTGGTGG 2929

Fig. S1

B2_BF1_(AB426141) TTGCTTGCTCCCTGTCCTGGCAGAACTCTGATTTTCCCAATGGCATCCCTG**TT**TGTTGGG 3400

B2_BF1_(PacBio_6sub1) TTGCTTGCTCCCTGTCCTGGCAGAACTCTGATTTTCCCAATGGCATCCCTG**TT**TGTTGGG 3400

B2_BF1_(AM279336) TTGCTTGCTCCCTGTCCTGGCAGAACTCTGATTTTCCCAATGGCATCCCTG**TT**TGTTGGG 2963

B2_BF1_(PacBio_7sub2) TTGCTTGCTCCCTGTCCTGGCAGAACTCTGATTTTCCCAATGGCATCCCTG**TT**TGTTGGG 3400

B2_BF1_(AM279340) TTGCTTGCTCCCTGTCCTGGCAGAACTCTGATTTTCCCAATGGCATCCCTG**TT**TGTTGGG 2963

B4_BF1_(AM279341) TTGCTTGCTCCCTGTCCTGGCAGAACTCTGATTTTCCCAATGGCATCCCTGGGTGTTGGG 2988

B4_BF1_(PacBio_C) TTGCTTGCTCCCTGTCCTGGCAGAACTCTGATTTTCCCAATGGCATCCCTGGGTGTTGGG 3424

B4_BF1_(AM279337) TTGCTTGCTCCCTGTCCTGGCAGAACTCTGATTTTCCCAATGGCATCCCTGGGTGTTGGG 2988

B12_BF1_(AB426147) TTGCTTGCTCCCTGTCCTGGCAGAACTCT**C**ATTTTCCCAATGGCATCCCTGGGTGTTGGG 3181

B12_BF1(PacBio_C) TTGCTTGCTCCCTGTCCTGGCAGAACTCT**C**ATTTTCCCAATGGCATCCCTGGGTGTTGGG 3178

B12_BF1_(AL023516) TTGCTTGCTCCCTGTCCTGGCAGAACTCT**C**ATTTTCCCAATGGCATCCCTGGGTGTTGGG 3181

B14_BF1_(PacBio_WL) ------------------------------------------------------------ 762

B15_BF1_(AB426149) TTGCTTGCTCCCTGTCCTGGCAGAACTCTGATTTTCCCAATGGCATCCCTGGGTGTTGGG 3426

B15_BF1_(PacBio_15I) ------------------------------------------------------------ 762

B19_BF1_(AB426151) TTGCTTGCTCCCTGTCCTGGCAGAACTCT**C**ATTTTCCCAATGGCATCCCTGGGTGTTGGG 3179

B19_BF1_(PacBio_P2a) TTGCTTGCTCCCTGTCCTGGCAGAACTCT**C**ATTTTCCCAATGGCATCCCTGGGTGTTGGG 3178

B19_BF1_(AM279338) TTGCTTGCTCCCTGTCCTGGCAGAACTCT**C**ATTTTCCCAATGGCATCCCTGGGTGTTGGG 2744

B21_BF1_(AB426152) TTGCTTGCTCCCTGTCCTGGCAGAACTCTGATTTTCCCAATGGCATCCCTGGGTGTTGGG 3426

B21_BF1_(PacBio_N) TTGCTTGCTCCCTGTCCTGGCAGAACTCTGATTTTCCCAATGGCATCCCTGGGTGTTGGG 3426

B21_BF1_(AM279339) TTGCTTGCTCCCTGTCCTGGCAGAACTCTGATTTTCCCAATGGCATCCCTGGGTGTTGGG 2989

B21_BF1_(PacBio_0) TTGCTTGCTCCCTGTCCTGGCAGAACTCTGATTTTCCCAATGGCATCCCTGGGTGTTGGG 3426

B21_BF1_(AM279342) TTGCTTGCTCCCTGTCCTGGCAGAACTCTGATTTTCCCAATGGCATCCCTGGGTGTTGGG 2989

B2_BF1_(AB426141) ATGTGGTCTCCTTGGTCCTCCCCCCAGCAGTCACTGCACATATCCACCCCACTTCCCCCC 3460

B2_BF1_(PacBio_6sub1) ATGTGGTCTCCTTGGTCCTCCCCCCAGCAGTCACTGCACATATCCACCCCACTTCCCCCC 3460

B2_BF1_(AM279336) ATGTGGTCTCCTTGGTCCTCCCCCCAGCAGTCACTGCACATATCCACCCCACTTCCCCCC 3023

B2_BF1_(PacBio_7sub2) ATGTGGTCTCCTTGGTCCTCCCCCCAGCAGTCACTGCACATATCCACCCCACTTCCCCCC 3460

B2_BF1_(AM279340) ATGTGGTCTCCTTGGTCCTCCCCCCAGCAGTCACTGCACATATCCACCCCACTTCCCCCC 3023

B4_BF1_(AM279341) ATGTGGTCTCCTTGGTCCTCCCCCCAGCAGTCACTGCACATATCCACCCCACTTCCCCCC 3048

B4_BF1_(PacBio_C) ATGTGGTCTCCTTGGTCCTCCCCCCAGCAGTCACTGCACATATCCACCCCACTTCCCCCC 3484

B4_BF1_(AM279337) ATGTGGTCTCCTTGGTCCTCCCCCCAGCAGTCACTGCACATATCCACCCCACTTCCCCCC 3048

B12_BF1_(AB426147) ATGTGGTCTCCTTGGTCCTCCCCCCAGCAGTCACTGCACATATCCACCCCACTTCCCCCC 3241

B12_BF1(PacBio_C) ATGTGGTCTCCTTGGTCCTCCCCCCAGCAGTCACTGCACATATCCACCCCACTTCCCCCC 3238

B12_BF1_(AL023516) ATGTGGTCTCCTTGGTCCTCCCCCCAGCAGTCACTGCACATATCCACCCCACTTCCCCCC 3241

B14_BF1_(PacBio_WL) ------------------------------------------------------------ 762

B15_BF1_(AB426149) ATGTGGTCTCCTTGGTCCTCCCCCCAGCAGTCACTGCACATATCCACCCCACTTCCCCCC 3486

B15_BF1_(PacBio_15I) ------------------------------------------------------------ 762

B19_BF1_(AB426151) ATGTGGTCTCCTTGGTCCTCCCCCCAGCAGTCACTGCACATATCCACCCCACTTCCCCCC 3239

B19_BF1_(PacBio_P2a) ATGTGGTCTCCTTGGTCCTCCCCCCAGCAGTCACTGCACATATCCACCCCACTTCCCCCC 3238

B19_BF1_(AM279338) ATGTGGTCTCCTTGGTCCTCCCCCCAGCAGTCACTGCACATATCCACCCCACTTCCCCCC 2804

B21_BF1_(AB426152) ATGTGGTCTCCTTGGTCCTCCCCCCAGCAGTCACTGCACATATCCACCCCACTTCCCCCC 3486

B21_BF1_(PacBio_N) ATGTGGTCTCCTTGGTCCTCCCCCCAGCAGTCACTGCACATATCCACCCCACTTCCCCCC 3486

B21_BF1_(AM279339) ATGTGGTCTCCTTGGTCCTCCCCCCAGCAGTCACTGCACATATCCACCCCACTTCCCCCC 3049

B21_BF1_(PacBio_0) ATGTGGTCTCCTTGGTCCTCCCCCCAGCAGTCACTGCACATATCCACCCCACTTCCCCCC 3486

B21_BF1_(AM279342) ATGTGGTCTCCTTGGTCCTCCCCCCAGCAGTCACTGCACATATCCACCCCACTTCCCCCC 3049

B2_BF1_(AB426141) CAGGTTGCTGTCCCACAGCACTCCT**A**TTTCCCTCTCCC**C**CCCCC**A**CC----------CGC 3510

B2_BF1_(PacBio_6sub1) CAGGTTGCTGTCCCACAGCACTCCT**A**TTTCCCTCTCCC**C**CC-CC**A**CC----------CGC 3509

B2_BF1_(AM279336) CAGGTTGCTGTCCCACAGCACTCCT**A**TTTCCCTCTCCC**C**CCCCC**A**CC----------CGC 3073

B2_BF1_(PacBio_7sub2) CAGGTTGCTGTCCCACAGCACTCCT**A**TTTCCCTCTCCC**C**CCCCC**A**CC----------CGC 3510

B2_BF1_(AM279340) CAGGTTGCTGTCCCACAGCACTCCT**A**TTTCCCTCTCCC**C**CCCCC**A**CC----------CGC 3073

B4_BF1_(AM279341) CAGGTTGCTGTCCCACAGCACTCCTGTTTCCCTCTCCCTCCCCCCCCC---------CGC 3099

B4_BF1_(PacBio_C) CAGGTTGCTGTCCCACAGCACTCCTGTTTCCCTCTCCCTCCCCCCCCC---------CGC 3535

B4_BF1_(AM279337) CAGGTTGCTGTCCCACAGCACTCCTGTTTCCCTCTCCCTCCCCCCCCC---------CGC 3099

B12_BF1_(AB426147) CAGGTTG**T**TGTCCCACAGCACTCCT**A**TTTCCCTCTCCC**C**CCCCCCCCC**CCCCCCCCC**CGC 3301

B12_BF1(PacBio_C) CAGGTTG**T**TGTCCCACAGCACTCCT**A**TTTCCCCTCCCC**C**CCCCCCCCC**--CCCCCCC**CGC 3296

B12_BF1_(AL023516) CAGGTTG**T**TGTCCCACAGCACTCCT**A**TTTCCCTCTCCC**C**CCCCCCCCC**CCCCCCCCC**CGC 3301

B14_BF1_(PacBio_WL) ------------------------------------------------------------ 762

B15_BF1_(AB426149) CAGGTTGCTGTCCCACAGCACTCCTGTTTCCCTCTCCCTCCCCCCCCC---------CGC 3537

B15_BF1_(PacBio_15I) ------------------------------------------------------------ 762

B19_BF1_(AB426151) CAGGTTG**T**TGTCCCACAGCACTCCT**A**TTTCCCTCTCCC**C**CCCCCCCCC**CC**NNNNNNNNNN 3299

B19_BF1_(PacBio_P2a) CAGGTTG**T**TGTCCCACAGCACTCCT**A**TTTCCCTCTCCC**C**CCCCCCCCC**---CCCCCC**CGC 3295

B19_BF1_(AM279338) CAGGTTG**T**TGTCCCACAGCACTCCT**A**TTTCCCTCTCCC**C**CCCCCCCCC**-CCCCGCCC**CGC 2863

B21_BF1_(AB426152) CAGGTTGCTGTCCCACAGCACTCCTGTTTCCCTCTCCCTCCCCCCCCC---------CGC 3537

B21_BF1_(PacBio_N) CAGGTTGCTGTCCCACAGCACTCCTGTTTCCCTCTCCCTCCCCCCCCC---------CGC 3537

B21_BF1_(AM279339) CAGGTTGCTGTCCCACAGCACTCCTGTTTCCCTCTCCCTCCCCCTCCC---------CGC 3100

B21_BF1_(PacBio_0) CAGGTTGCTGTCCCACAGCACTCCTGTTTCCCTCTCCCTCCCCCCCCC---------CGC 3537

B21_BF1_(AM279342) CAGGTTGCTGTCCCACAGCACTCCTGTTTCCCTCTCCCTCCCCCTCCC---------CGC 3100

Fig. S1

B2_BF1_(AB426141) CCATCCAGCTGCCTCTGCAATCCTCACCCTTGCCCAC**C**CACAACCTTGCCCACTCCACCT 3570

B2_BF1_(PacBio_6sub1) CCATCCAGCTGCCTCTGCAATCCTCACCCTTGCCCAC**C**CACAACCTTGCCCACTCCACCT 3569

B2_BF1_(AM279336) CCATCCAGCTGCCTCTGCAATCCTCACCCTTGCCCAC**C**CACAACCTTGCCCACTCCACCT 3133

B2_BF1_(PacBio_7sub2) CCATCCAGCTGCCTCTGCAATCCTCACCCTTGCCCAC**C**CACAACCTTGCCCACTCCACCT 3570

B2_BF1_(AM279340) CCATCCAGCTGCCTCTGCAATCCTCACCCTTGCCCAC**C**CACAACCTTGCCCACTCCACCT 3133

B4_BF1_(AM279341) CCATCCAGCTGCCTCTGCAATCCTCACC**----------**CACAACCTTGCCCACTCCACCT 3149

B4_BF1_(PacBio_C) CCATCCAGCTGCCTCTGCAATCCTCACC**----------**CACAACCTTGCCCACTCCACCT 3585

B4_BF1_(AM279337) CCATCCAGCTGCCTCTGCAATCCTCACC**----------**CACAACCTTGCCCACTCCACCT 3149

B12_BF1_(AB426147) CCATCCAGCTGCCTCTGCAATCCTCACCCTTGCCCACACACAAC**T**TTGC**G**CACTCCACCT 3361

B12_BF1(PacBio_C) CCATCCAGCTGCCTCTGCAATCCTCACCCTTGCCCACACACAAC**T**TTGC**G**CACTCCACCT 3356

B12_BF1_(AL023516) CCATCCAGCTGCCTCTGCAATCCTCACCCTTGCCCACACACAAC**T**TTGC**G**CACTCCACCT 3361

B14_BF1_(PacBio_WL) ------------------------------------------------------------ 762

B15_BF1_(AB426149) CCATCCAGCTGCCTCTGCAATCCTCACCCTTGCCCACACACAACCTTGCCCACTCCACCT 3597

B15_BF1_(PacBio_15I) ------------------------------------------------------------ 762

B19_BF1_(AB426151) NNNNNNNNNNNNNNNNNNNNNNNNNNNNNNNNNNNNNNNNNNNNNNNNNNNNNN------ 3353

B19_BF1_(PacBio_P2a) CCATCCAGCTGCCTCTGCAATCCTCACCCTTGCCCACACACAAC**T**TTGC**G**CACTCCACCT 3355

B19_BF1_(AM279338) CCATCCAGCTGCCTCTGCAA-CCTCACCCTTGCCCACACACAAC**T**TTGC**G**CACTCCACCT 2922

B21_BF1_(AB426152) CCATCCAGCTGCCTCTGCAATCCTCACCCTTGCCCACACACAACCTTGCCCACTCCACCT 3597

B21_BF1_(PacBio_N) CCATCCAGCTGCCTCTGCAATCCTCACCCTTGCCCACACACAACCTTGCCCACTCCACCT 3597

B21_BF1_(AM279339) CCATCCAGCTGCCTCTGCAATCCTCACCCTTGCCCACACACAACCTTGCCCACTCCACCT 3160

B21_BF1_(PacBio_0) CCATCCAGCTGCCTCTGCAAT-CTCACCCTTGCCCACACACAACCTTGCCCACTCCACCT 3596

B21_BF1_(AM279342) CCATCCAGCTGCCTCTGCAAT-CTCACCCTTGCCCACACACAACCTTGCCCACTCCACCT 3159

B2_BF1_(AB426141) CCCTCATCCCGCCCTTCCCCCAGCTCTCCTGTCCCTGCTGGGCCCCCTC---CCCCCACA 3627

B2_BF1_(PacBio_6sub1) CCCTCATCCCGCCCTTCCCCCAGCTCTCCTGTCCCTGCTGGGCCCCCTC---CCCCCACA 3626

B2_BF1_(AM279336) CCCTCATCCCGCCCTTCCCCCAGCTCTCCTGTCCCTGCTGGGCCCCCTC---CCCCCACA 3190

B2_BF1_(PacBio_7sub2) CCCTCATCCCGCCCTTCCCCCAGCTCTCCTGTCCCTGCTGGGCCCCCTC---CCCCCACA 3627

B2_BF1_(AM279340) CCCTCATCCCGCCCTTCCCCCAGCTCTCCTGTCCCTGCTGGGCCCCCTC---CCCCCACA 3190

B4_BF1_(AM279341) CCCTCATCCCGCCCTTCCCCCAGCTCTCCTGTCCCTGCTGGGCCCCCTC---CCCCCACA 3206

B4_BF1_(PacBio_C) CCCTCATCCCGCCCTTCCCCCAGCTCTCCTGTCCCTGCTGGGCCCCCTC---CCCCCACA 3642

B4_BF1_(AM279337) CCCTCATCCCGCCCTTCCCCCAGCTCTCCTGTCCCTGCTGGGCCCCCTC---CCCCCACA 3206

B12_BF1_(AB426147) CCCTCATCCCGCCCTTCCCCCAGCTCTCCTGTCCCTGCTGGCCCCCCTC**CCC**CCCCC**C**CA 3421

B12_BF1(PacBio_C) CCCTCATCCCGCCCTTCCCCCAGCTCTCCTGTCCCTGCTGGCCCCCCTC**CCC**CCCCC**C**CA 3416

B12_BF1_(AL023516) CCCTCATCCCGCCCTTCCCCCAGCTCTCCTGTCCCTGCTGGCCCCCCTC**CCC**CCCCC**C**CA 3421

B14_BF1_(PacBio_WL) ------------------------------------------------------------ 762

B15_BF1_(AB426149) CCCTCATCCCGCCCTTCCCCCAGCTCTCCTGTCCCTGCTGGGCCCCCTC---CCCCCACA 3654

B15_BF1_(PacBio_15I) ------------------------------------------------------------ 762

B19_BF1_(AB426151) -------------NNNNNNNNNNNNNNNNNNNNNNNNNNNNNNNNNCCC**CTC**TCCCCACA 3400

B19_BF1_(PacBio_P2a) CCCTCATCCCGCCCTTCCCCCAGCTCTCCTGTCCCTGCTGGCCCCCCTC**CCC**CCCCC**C**CA 3415

B19_BF1_(AM279338) CCCTCATCCCGCCCTTCCCCCAGCTCTCCTGTCCCTGCTGGCCCCCCTC**CCC**CCCCC**C**CA 2982

B21_BF1_(AB426152) CCCTCATCCCGCCCTTCCCCCAGCTCTCCTGTCCCTGCTGGGCCCCCTC---CCCCCACA 3654

B21_BF1_(PacBio_N) CCCTCATCCCGCCCTTCCCCCAGCTCTCCTGTCCCTGCTGGGCCCCCTC---CCCCCACA 3654

B21_BF1_(AM279339) CCCTCATCCCGCCCTTCCCCCAGCTCTCCTGTCCCTGCTGGGCCCCCTC---CCCCCACA 3217

B21_BF1_(PacBio_0) CCCTCATCCCGCCCTTCCCCCAGCTCTCCTGTCCCTGCTGGGCCCCCTC---CCCCCACA 3653

B21_BF1_(AM279342) CCCTCATCCCGCCCTTCCCCCAGCTCTCCTGTCCCTGCTGGGCCCCCTC---CCCCCACA 3216

**BF1 PolyA site 2 TAP1 PolyA site 2**

B2_BF1_(AB426141) TTGTACCCTACACCCAAATAAATATGTTTGTTCTGCTGCCCTCCAGCCGTCT**T**CTGGTTT 3687

B2_BF1_(PacBio_6sub1) TTGTACCCTACACCCAAATAAATATGTTTGTTCTGCTGCCCTCCAGCCGTCT**T**CTGGTTT 3686

B2_BF1_(AM279336) TTGTACCCTACACCCAAATAAATATGTTTGTTCTGCTGCCCTCCAGCCGTCT**T**CTGG 3247

B2_BF1_(PacBio_7sub2) TTGTACCCTACACCCAAATAAATATGTTTGTTCTGCTGCCCTCCAGCCGTCT**T**CTGGTTT 3687

B2_BF1_(AM279340) TTGTACCCTACACCCAAATAAATATGTTTGTTCTGCTGCCCTCCAGCCGTCT**T**CTGG 3247

B4_BF1_(AM279341) TTGTACCCTACACCCAAATAAATATGTTTGTTCTGCTGCCCTCCAGCCGTCTCCTGG 3263

B4_BF1_(PacBio_C) TTGTACCCTACACCCAAATAAATATGTTTGTTCTGCTGCCCTCCAGCCGTCTCCTGGTTT 3702

B4_BF1_(AM279337) TTGTACCCTACACCCAAATAAATATGTTTGTTCTGCTGCCCTCCAGCCGTCTCCTGG 3263

B12_BF1_(AB426147) TTGTACCCTACACCCAAATAAATATGTTTGTTCTGCTGCCCTCCAGCCGTCTCCTGGTTT 3481

B12_BF1(PacBio_C) TTGTACCCTACACCCAAATAAATATGTTTGTTCTGCTGCCCTCCAGCCGTCTCCTGGTTT 3476

B12_BF1_(AL023516) TTGTACCCTACACCCAAATAAATATGTTTGTTCTGCTGCCCTCCAGCCGTCTCCTGGTTT 3481

B14_BF1_(PacBio_WL) ------------------------------------------------------------ 762

B15_BF1_(AB426149) TTGTACCCTACACCCAAATAAATATGTTTGTTCTGCTGCCCTCTAGCCGTCTCCTGGTTT 3714

B15_BF1_(PacBio_15I) ------------------------------------------------------------ 762

B19_BF1_(AB426151) TTGTACCCTACACCCAAATAAATATGTTTGTTCTGCTGCCCTCCAGCCGTCTCCTGGTTT 3460

B19_BF1_(PacBio_P2a) TTGTACCCTACACCCAAATAAATATGTTTGTTCTGCTGCCCTCCAGCCGTCTCCTGGTTT 3475

B19_BF1_(AM279338) TTGTACCCTACACCCAAATAAATATGTTTGTTCTGCTGCCCTCCAGCCGTCTCCTGG 3039

B21_BF1_(AB426152) TTGTACCCTACACCCAAATAAATATGTTTGTTCTGCTGCCCTC**T**AGCCGTCTCCTGGTTT 3714

B21_BF1_(PacBio_N) TTGTACCCTACACCCAAATAAATATGTTTGTTCTGCTGCCCTC**T**AGCCGTCTCCTGGTTT 3714

B21_BF1_(AM279339) TTGTACCCTACACCCAAATAAATATGTTTGTTCTGCTGCCCTC**T**AGCCGTCTCCTGG 3274

B21_BF1_(PacBio_0) TTGTACCCTACACCCAAATAAATATGTTTGTTCTGCTGCCCTC**T**AGCCGTCTCCTGGTTT 3713

B21_BF1_(AM279342) TTGTACCCTACACCCAAATAAATATGTTTGTTCTGCTGCCCTC**T**AGCCGTCTCCTGG 3273

Fig. S1

**TAP1 exon 11**

B2_BF1_(AB426141) ATT**C**C**C**CCCCCGATTTGTTGTTGTTGGGGGCTCCGCTCTTCACCCTGGGGGGAAGGGGCT 3747

B2_BF1_(PacBio_6sub1) ATT**C**C**C**CCCCCGATTTGTTGTTGTTGGGGGCTCCGCTCTTCACCCTGGGGGGAAGGGGCT 3746

B2_BF1_(AM279336) 3247

B2_BF1_(PacBio_7sub2) ATT**C**C**C**CCCCCGATTTGTTGTTGTTGGGGGCTCCGCTCTTCACCCTGGGGGGAAGGGGCT 3747

B2_BF1_(AM279340) 3247

B4_BF1_(AM279341) 3263

B4_BF1_(PacBio_C) ATTTC-CCCCCGATTTGTTGTTGTTGGGGGCTCCGCTCTTCACCCTGGGGGGAAGGGGCT 3761

B4_BF1_(AM279337) 3263

B12_BF1_(AB426147) ATTTC-CCCCCGATTTGTTGTTGTTGGGGGCTCCGCTCTTCACCCTGGGGGGAAGGGGCT 3540

B12_BF1(PacBio_C) ATTTC-CCCCCGATTTGTTGTTGTTGGGGGCTCCGCTCTTCACCCTGGGGGGAAGGGGCT 3535

B12_BF1_(AL023516) ATTTC-CCCCCGATTTGTTGTTGTTGGGGGCTCCGCTCTTCACCCTGGGGGGAAGGGGCT 3540

B14_BF1_(PacBio_WL) ------------------------------------------------------------ 762

B15_BF1_(AB426149) ATTTC-CCCCCGATTTGTTGTTGTTGGGGGCTCCGCTCTTCACCCTGGGGGGAAGGGGCT 3773

B15_BF1_(PacBio_15I) ------------------------------------------------------------ 762

B19_BF1_(AB426151) ATTTC-CCCCCGATTTGTTGTTGTTGGGGGCTCCGCTCTTCACCCTGGGGGGAAGGGGCT 3519

B19_BF1_(PacBio_P2a) ATTTC-CCCCCGATTTGTTGTTGTTGGGGGCTCCGCTCTTCACCCTGGGGGGAAGGGGCT 3534

B19_BF1_(AM279338) 3039

B21_BF1_(AB426152) ATTTC-CCCCCGATTTGTTGTTGTTGGGGGCTCCGCTCTTCACCCTGGGGGGAAGGGGCT 3773

B21_BF1_(PacBio_N) ATTTC-CCCCCGATTTGTTGTTGTTGGGGGCTCCGCTCTTCACCCTGGGGGGAAGGGGCT 3773

B21_BF1_(AM279339) 3274

B21_BF1_(PacBio_0) ATTTC-CCCCCGATTTGTTGTTGTTGGGGGCTCCGCTCTTCACCCTGGGGGGAAGGGGCT 3772

B21_BF1_(AM279342) 3273

B2_BF1_(AB426141) CTGGGGGTCCCTCATTCTCCCTGCACTTCTTACAGCACCGGGACTCCCCGCGCTGAGATC 3807

B2_BF1_(PacBio_6sub1) CTGGGGGTCCCTCATTCTCCCTGCACTTCTTACAGCACCGGGACTCCCCGCGCTGAGATC 3806

B2_BF1_(AM279336) 3247

B2_BF1_(PacBio_7sub2) CTGGGGGTCCCTCATTCTCCCTGCACTTCTTACAGCACCGGGACTCCCCGCGCTGAGATC 3807

B2_BF1_(AM279340) 3247

B4_BF1_(AM279341) 3263

B4_BF1_(PacBio_C) CTGGGGGTCCCTCATTCTCCCTGCACTTCTTACAGCACCGGGACTCCCCGTGCTGAGATC 3821

B4_BF1_(AM279337) 3263

B12_BF1_(AB426147) CTGGGGGTCCCTCATTCTCCCTGCACTTCTTACAGCACCGGGACTC**-**CCGCGCTGAGATC 3599

B12_BF1(PacBio_C) CTGGGGGTCCCTCATTCTCCCTGCACTTCTTACAGCACCGGGACTC**-**CCGCGCTGAGATC 3594

B12_BF1_(AL023516) CTGGGGGTCCCTCATTCTCCCTGCACTTCTTACAGCACCGGGACTC**-**CCGCGCTGAGATC 3599

B14_BF1_(PacBio_WL) ------------------------------------------------------------ 762

B15_BF1_(AB426149) CTGGGGGTCCCTCATTCTCCCTGCACTTCTTACAGCACCGGGACTCCCCGCGCTGAGATC 3833

B15_BF1_(PacBio_15I) ------------------------------------------------------------ 762

B19_BF1_(AB426151) CTGGGGGTCCCTCATTCTCCCTGCACTTCTTACAGCACCGGGACTC**-**CCGCGCTGAGATC 3578

B19_BF1_(PacBio_P2a) CTGGGGGTCCCTCATTCTCCCTGCACTTCTTACAGCACCGGGACTC**-**CCGCGCTGAGATC 3593

B19_BF1_(AM279338) 3039

B21_BF1_(AB426152) CTGGGGGTCCCTCATTCTCCCTGCACTTCTTACAGCACCGGGACTCCCCGCGCTGAGATC 3833

B21_BF1_(PacBio_N) CTGGGGGTCCCTCATTCTCCCTGCACTTCTTACAGCACCGGGACTCCCCGCGCTGAGATC 3833

B21_BF1_(AM279339) 3274

B21_BF1_(PacBio_0) CTGGGGGTCCCTCATTCTCCCTGCACTTCTTACAGCACCGGGACTCCCCGCGCTGAGATC 3832

B21_BF1_(AM279342) 3273

**TAP1 PolyA site 1**

B2_BF1_(AB426141) CCAACACACCCGGGTACAAACATGCGGCTTTATTCCCAGTTCTGTGTCCCACCCCCGGCC 3867

B2_BF1_(PacBio_6sub1) CCAACACACCCGGGTACAAACATGCGGCTTTATTCCCAGTTCTGTGTCCCACCCCCGGCC 3866

B2_BF1_(AM279336) 3247

B2_BF1_(PacBio_7sub2) CCAACACACCCGGGTACAAACATGCGGCTTTATTCCCAGTTCTGTGTCCCACCCCCGGCC 3867

B2_BF1_(AM279340) 3247

B4_BF1_(AM279341) 3263

B4_BF1_(PacBio_C) CCAACACACCCGGGTACAAACATGCGGCTTTATTCCCAGTTCTGTGTCCCACCCCCGGCC 3881

B4_BF1_(AM279337) 3263

B12_BF1_(AB426147) CCA**T**CACACCCGGGTACAAACATGCGGCTTTATTCCCAGTTCTGTGTCCCACCCCCGGCC 3659

B12_BF1(PacBio_C) CCA**T**CACACCCGGGTACAAACATGCGGCTTTATTCCCAGTTCTGTGTCCCACCCCCGGCC 3654

B12_BF1_(AL023516) CCA**T**CACACCCGGGTACAAACATGCGGCTTTATTCCCAGTTCTGTGTCCCACCCCCGGCC 3659

B14_BF1_(PacBio_WL) ------------------------------------------------------------ 762

B15_BF1_(AB426149) CCAACACACCCGGGTACAAACATGCGGCTTTATTCCCAGTTCTGTGTCCCACCCCCGGCC 3893

B15_BF1_(PacBio_15I) ------------------------------------------------------------ 762

B19_BF1_(AB426151) CCA**T**CACACCCGGGTACAAACATGCGGCTTTATTCCCAGTTCTGTGTCCCACCCCCGGCC 3638

B19_BF1_(PacBio_P2a) CCA**T**CACACCCGGGTACAAACATGCGGCTTTATTCCCAGTTCTGTGTCCCACCCCCGGCC 3653

B19_BF1_(AM279338) 3039

B21_BF1_(AB426152) CCAACACACCCGGGTACAAACATGCGGCTTTATTCCCAGTTCTGTGTCCCACCCCCGGCC 3893

B21_BF1_(PacBio_N) CCAACACACCCGGGTACAAACATGCGGCTTTATTCCCAGTTCTGTGTCCCACCCCCGGCC 3893

B21_BF1_(AM279339) 3274

B21_BF1_(PacBio_0) CCAACACACCCGGGTACAAACATGCGGCTTTATTCCCAGTTCTGTGTCCCACCCCCGGCC 3892

B21_BF1_(AM279342) 3273

Fig. S1

B2_BF1_(AB426141) CTGGTGGCACTCAGTGGCACCGCAGTCCATGCAGTGGCCGTTGTGTGTCGTACAGCAGCG 3927

B2_BF1_(PacBio_6sub1) CTGGTGGCACTCAGTGGCACCGCAGTCCATGCAGTGGCCGTTGTGTGTCGTACAGCAGCG 3926

B2_BF1_(AM279336) 3247

B2_BF1_(PacBio_7sub2) CTGGTGGCACTCAGTGGCACCGCAGTCCATGCAGTGGCCGTTGTGTGTCGTACAGCAGCG 3927

B2_BF1_(AM279340) 3247

B4_BF1_(AM279341) 3263

B4_BF1_(PacBio_C) CTGGTGGCACTCAGTGGCACTGCAGTCCATGC**T**GTGGCCGTTGTGTGTCGTACAGCAGCG 3941

B4_BF1_(AM279337) 3263

B12_BF1_(AB426147) CTGGTGGCACTCAGTGGCACCGCAGTCCATGCAGTGGCCGTTGTGTGTCGTACAGCAGCG 3719

B12_BF1(PacBio_C) CTGGTGGCACTCAGTGGCACCGCAGTCCATGCAGTGGCCGTTGTGTGTCGTACAGCAGCG 3714

B12_BF1_(AL023516) CTGGTGGCACTCAGTGGCACCGCAGTCCATGCAGTGGCCGTTGTGTGTCGTACAGCAGCG 3719

B14_BF1_(PacBio_WL) ------------------------------------------------------------ 762

B15_BF1_(AB426149) C**C**GGTGGCACTCAGTGGCACCGCAGTCCATGCAGTGGCCGTTGTGTGTCGTACAGCAGCG 3953

B15_BF1_(PacBio_15I) ------------------------------------------------------------ 762

B19_BF1_(AB426151) CTGGTGGCACTCAGTGGCACCGCAGTCCATGCAGTGGCCGTTGTGTGTCGTACAGCAGCG 3698

B19_BF1_(PacBio_P2a) CTGGTGGCACTCAGTGGCACCGCAGTCCATGCAGTGGCCGTTGTGTGTCGTACAGCAGCG 3713

B19_BF1_(AM279338) 3039

B21_BF1_(AB426152) C**C**GGTGGCACTCAGTGGCACCGCAGTCCATGC**T**GT**A**GCCGTTGTGTGTCGTACAGCAGCG 3953

B21_BF1_(PacBio_N) C**C**GGTGGCACTCAGTGGCACCGCAGTCCATGC**T**GT**A**GCCGTTGTGTGTCGTACAGCAGCG 3953

B21_BF1_(AM279339) 3274

B21_BF1_(PacBio_0) C**C**GGTGGCACTCAGTGGCACCGCAGTCCATGC**T**GT**A**GCCGTTGTGTGTCGTACAGCAGCG 3952

B21_BF1_(AM279342) 3273

**-------BF1 R primer---**

B2_BF1_(AB426141) GTACCGCAGCGCGCCCGGCTCGGCATCCATGTGCCCACGGCACAGCTCTTGTGGTCCCTT 3987

B2_BF1_(PacBio_6sub1) GTACCGCAGCGCGCCCGGCTCGGCATCCATGTGCCCACGGCACAGCTCTTGTGGTCCCTT 3986

B2_BF1_(AM279336) 3247

B2_BF1_(PacBio_7sub2) GTACCGCAGCGCGCCCGGCTCGGCATCCATGTGCCCACGGCACAGCTCTTGTGGTCCCTT 3987

B2_BF1_(AM279340) 3247

B4_BF1_(AM279341) 3263

B4_BF1_(PacBio_C) GTACCGCAGCGCGCCCGGCTCGGCATCCATGTGCCCACGGCACAGCTCTTGTGGTCCCTT 4001

B4_BF1_(AM279337) 3263

B12_BF1_(AB426147) GTACCGCAGCGCGCCCGGCTCGGCATCCATGTGCCCACGGCACAGCTCTTGTGGTCCCTT 3779

B12_BF1(PacBio_C) GTACCGCAGCGCGCCCGGCTCGGCATCCATGTGCCCACGGCACAGCTCTTGTGGTCCCTT 3774

B12_BF1_(AL023516) GTACCGCAGCGCGCCCGGCTCGGCATCCATGTGCCCACGGCACAGCTCTTGTGGTCCCTT 3779

B14_BF1_(PacBio_WL) -----GCAGCGCGCCCGGCTCGGCATCCATGTGCCCACGGCACAGCTCTTGTGGTCCCTT 817

B15_BF1_(AB426149) GTACCGCAGCGCGCCCGGCTCGGCATCCA**C**GTGCCCACGGCACAGCTCTTGTGGTCCCTT 4013

B15_BF1_(PacBio_15I) -----GCAGCGCGCCCGGCTCGGCATCCATGTGCCCACGGCACAGCTCTTGTGGTCCCTT 817

B19_BF1_(AB426151) GTACCGCAGCGCGCCCGGCTCGGCATCCATGTGCCCACGGCACAGCTCTTGTGGTCCCTT 3758

B19_BF1_(PacBio_P2a) GTACCGCAGCGCGCCCGGCTCGGCATCCATGTGCCCACGGCACAGCTCTTGTGGTCCCTT 3773

B19_BF1_(AM279338) 3039

B21_BF1_(AB426152) GTACCGCAGCGCGCCCGGCTCGGCATCCATGTGCCCACGGCACAGCTCTTGTGGTCCCTT 4013

B21_BF1_(PacBio_N) GTACCGCAGCGCGCCCGGCTCGGCATCCATGTGCCCACGGCACAGCTCTTGTGGTCCCTT 4013

B21_BF1_(AM279339) 3274

B21_BF1_(PacBio_0) GTACCGCAGCGCGCCCGGCTCGGCATCCATGTGCCCACGGCACAGCTCTTGTGGTCCCTT 4012

B21_BF1_(AM279342) 3273

**---**

B2_BF1_(AB426141) GTC 3990

B2_BF1_(PacBio_6sub1) GTC 3989

B2_BF1_(AM279336) 3247

B2_BF1_(PacBio_7sub2) GTC 3990

B2_BF1_(AM279340) 3247

B4_BF1_(AM279341) 3263

B4_BF1_(PacBio_C) GTC 4004

B4_BF1_(AM279337) 3263

B12_BF1_(AB426147) GTC 3782

B12_BF1(PacBio_C) GTC 3777

B12_BF1_(AL023516) GTC 3782

B14_BF1_(PacBio_WL) GTC 820

B15_BF1_(AB426149) GTC 4016

B15_BF1_(PacBio_15I) GTC 820

B19_BF1_(AB426151) GTC 3761

B19_BF1_(PacBio_P2a) GTC 3776

B19_BF1_(AM279338) 3039

B21_BF1_(AB426152) GTC 4016

B21_BF1_(PacBio_N) GTC 4016

B21_BF1_(AM279339) 3274

B21_BF1_(PacBio_0) GTC 4015

B21_BF1_(AM279342) 3273

Fig. S1

------**BF2 R primer------**

B2_BF2_(AB426141) ------------------------------------------------------------ 0

B2_BF2_(PacBio_6sub1) CACTGATCCCAAAGGAAGCCCTGGGGGACACCACTGTCACCACCC--CC**T**CCCCCCCGGA 58

B2_BF2_(AM282692) ------------------------------------------------------------ 0

B2_BF2_(PacBio_7sub2) CACTGATCCCAAAGGAAGCCCTGGGGGACACCACTGTCACCACCC--CC**T**CCCCCCCGGA 58

B2_BF2_(AM282698) ------------------------------------------------------------ 0

B4_BF2_(AM282699) ------------------------------------------------------------ 0

B4_BF2_(PacBio_C) CACTGATCCCAAAGGAAGCCCTGGGGGACACCACTGTCACCACCC--**--**CCCCCCCCGGA 56

B4_BF2_(AM282693) ------------------------------------------------------------ 0

B12_BF2_(AB426147) ------------------------------------------------------------ 0

B12_BF2_(PacBio_C) CACTGATCCCAAAGGAAGCCCTGGGGGACACCACTGTCACCACCC--CC**T**CCCCCCCGGA 58

B12_BF2_(AL023516) CACTGATCCCAAAGGAAGCCCTGGGGGACACCACTGTCACCACCC--CC**T**CCCCCCCGGA 58

B14_BF2_(PacBio_WL) CACTGATCCCAAAGGAAGCCCTGGGGGACACCACTGTCACCACC**ACT**CCCCCCCCCCGGA 60

B14_BF2_(AM282694) ------------------------------------------------------------ 0

B15_BF2_(AB426149) ------------------------------------------------------------ 0

B15_BF2_(PacBio_15I) CACTGATCCCAAAGGAAGCCCTGGGGGACACCACTGTCACCACCC--CC**T**CCCCCCCGGA 58

B15_BF2_(AM282695) ------------------------------------------------------------ 0

B19_BF2_(AB426151) ------------------------------------------------------------ 0

B19_BF2_(PacBio_P2a) CACTGATCCCAAAGGAAGCCCTGGAGGACACCACTGTCACCGCCC--**-T**CCCCCCCCGGA 57

B19_BF2_(AM282696) ------------------------------------------------------------ 0

B21_BF2_(AB426152) ------------------------------------------------------------ 0

B21_BF2_(PacBio_N) CACTGATCCCAAAGGAAGCCCTGGGGGACACCACTGTCACCACCC-**C**CCCCC**A**CCCCGGA 59

B21_BF2_(AM282697) ------------------------------------------------------------ 0

B21_BF2_(PacBio_0) ------------------------------------------------------------ 0

B21_BF2_(AM282700) ------------------------------------------------------------ 0

B2_BF2_(AB426141) CCTTGACTGGAAT 13

B2_BF2_(PacBio_6sub1) CACGGAGCCTCTAACCCTATGGATGTGACC-CCCCCCCCAAACAGTTCCTTGACTGGAAT 117

B2_BF2_(AM282692) ------------------------------------------------------------ 0

B2_BF2_(PacBio_7sub2) CACGGAGCCTCTAACCCTATGGATGTGACC-CCCCCCCCAAACAGTTCCTTGACTGGAAT 117

B2_BF2_(AM282698) ------------------------------------------------------------ 0

B4_BF2_(AM282699) ------------------------------------------------------------ 0

B4_BF2_(PacBio_C) CACGGAGCCTCTAACCCTATGGATGTGACC-**-**CCCCCCCAAACAGTTCCTTGACTGGAAT 114

B4_BF2_(AM282693) ------------------------------------------------------------ 0

B12_BF2_(AB426147) CCTTGACTGGAAT 13

B12_BF2_(PacBio_C) CACGGAGCCTCTAACCCTATGGATGTGACC-CCCCCCCCAAACAGTTCCTTGACTGGAAT 117

B12_BF2_(AL023516) CACGGAGCCTCTAACCCTATGGATGTGACC-CCCCCCCCAAACAGTTCCTTGACTGGAAT 117

B14_BF2_(PacBio_WL) CACGGAGCCTCTAACCCTATGGATGTGACC-CCCCCCACAAACAGTTCCTTGACTGGAAT 119

B14_BF2_(AM282694) ------------------------------------------------------------ 0

B15_BF2_(AB426149) AT 2

B15_BF2_(PacBio_15I) CACGGAGCCTCTAACCCTATGGATGTGACC-CCCCCCCCAAACAGTTCCTTGACTGGAAT 117

B15_BF2_(AM282695) ------------------------------------------------------------ 0

B19_BF2_(AB426151) CCTTGACTGGAAT 13

B19_BF2_(PacBio_P2a) CACGGAGCCTCTAACCCTATGGATGTGACC**C**CCCCCCCCAAACAGTTCCTTGACTGGAAT 117

B19_BF2_(AM282696) ------------------------------------------------------------ 0

B21_BF2_(AB426152) CCTTGACTGGAAT 13

B21_BF2_(PacBio_N) CACGGAGCCTCTAACCCTATGGATGTGACC-**-**CCCCCCCAAACAGTTCCTTGACTGGAAT 117

B21_BF2_(AM282697) ------------------------------------------------------------ 0

B21_BF2_(PacBio_0) CCTTGACTGGAAT 13

B21_BF2_(AM282700) ------------------------------------------------------------ 0

B2_BF2_(AB426141) GGAGAATGACTCCTCCTCTTCTCACGGCCCCCGTCCTCCCGGCAGAGACCCTCGGCCCCC 73

B2_BF2_(PacBio_6sub1) GGAGAATGACTCCTCCTCTTCTCACGGCCCCCGTCCTCCCGGCAGAGACCCTCGGCCCCC 177

B2_BF2_(AM282692) CTCCTCCTCTTCTCACGGCCCCCGTCCTCCCGGCAGAGACCCTCGGCCCCC 51

B2_BF2_(PacBio_7sub2) GGAGAATGACTCCTCCTCTTCTCACGGCCCCCGTCCTCCCGGCAGAGACCCTCGGCCCCC 177

B2_BF2_(AM282698) CTCCTCCTCTTCTCACGGCCCCCGTCCTCCCGGCAGAGACCCTCGGCCCCC 51

B4_BF2_(AM282699) CTCCTCCTCTTCTCACGGCCCCCGTCCTCCCGGCAGAGACCCTCGGCCCCC 51

B4_BF2_(PacBio_C) GGAGAATGACTCCTCCTCTTCTCACGGCCCCCGTCCTCCCGGCAGAGACCCTCGGCCCCC 174

B4_BF2_(AM282693) CTCCTCCTCTTCTCACGGCCCCCGTCCTCCCGGCAGAGACCCTCGGCCCCC 51

B12_BF2_(AB426147) GGAGAATGACTCCTCCTCTTCTCACGGCCCCCGTCCTCCCGGCAGAGACCCTCGGCCCCC 73

B12_BF2_(PacBio_C) GGAGAATGACTCCTCCTCTTCTCACGGCCCCCGTCCTCCCGGCAGAGACCCTCGGCCCCC 177

B12_BF2_(AL023516) GGAGAATGACTCCTCCTCTTCTCACGGCCCCCGTCCTCCCGGCAGAGACCCTCGGCCCCC 177

B14_BF2_(PacBio_WL) GGAGAATGACTCCTCCTCTTCTCACGGCCCCCGTCCTCCCGGCAGAGACCCTCGGCCCCC 179

B14_BF2_(AM282694) CTCCTCCTCTTCTCACGGCCCCCGTCCTCCCGGCAGAGACCCTCGGCCCCC 51

B15_BF2_(AB426149) GGAGAATGACTCCTCCTCTTCTCACGGCCCCCGTCCTCCCGGCAGAGACCCTCGGCCCCC 62

B15_BF2_(PacBio_15I) GGAGAATGACTCCTCCTCTTCTCACGGCCCCCGTCCTCCCGGCAGAGACCCTCGGCCCCC 177

B15_BF2_(AM282695) CTCCTCCTCTTCTCACGGCCCCCGTCCTCCCGGCAGAGACCCTCGGCCCCC 51

B19_BF2_(AB426151) GGAGAATGACTCCTCCTCTTCTCACGGCCCCCGTCCTCCCGGCAGAGACCCTCGGCCCCC 73

B19_BF2_(PacBio_P2a) GGAGAATGACTCCTCCTCTTCTCACGGCCCCCGTCCTCCCGGCAGAGACCCTCGGCCCCC 177

B19_BF2_(AM282696) CTCCTCCTCTTCTCACGGCCCCCGTCCTCCCGGCAGAGACCCTCGGCCCCC 51

B21_BF2_(AB426152) GGAGAATGACTCCTCCTCTTCTCACGGCCCCCGTCCTCCCGGCAGAGACCCTCGGCCCCC 73

B21_BF2_(PacBio_N) GGAGAATGACTCCTCCTCTTCTCACGGCCCCCGTCCTCCCGGCAGAGACCCTCGGCCCCC 177

B21_BF2_(AM282697) CTCCTCCTCTTCTCACGGCCCCCGTCCTCCCGGCAGAGACCCTCGGCCCCC 51

B21_BF2_(PacBio_0) GGAGAATGACTCCTCCTCTTCTCACGGCCCCCGTCCTCCCGGCAGAGACCCTCGGCCCCC 73

B21_BF2_(AM282700) CTCCTCCTCTTCTCACGGCCCCCGTCCTCCCGGCAGAGACCCTCGGCCCCC 51

Fig. S2

**-**

B2_BF2_(AB426141) TCCCCCGGGGTAAGGACGCGGCCCCGTGCG**A**ACACAGCCCGGGTGCCCCCCCGTGCTCGA 133

B2_BF2_(PacBio_6sub1) TCCCCCGGGGTAAGGACGCGGCCCCGTGCG**A**ACACAGCCCGGGTGCCCCCCCGTGCTCGA 237

B2_BF2_(AM282692) TCCCCCGGGGTAAGGACGCGGCCCCGTGCG**A**ACACAGCCCGGGTGCCCCCCCGTGCTCGA 111

B2_BF2_(PacBio_7sub2) TCCCCCGGGGTAAGGACGCGGCCCCGTGCG**A**ACACAGCCCGGGTGCCCCCCCGTGCTCGA 237

B2_BF2_(AM282698) TCCCCCGGGGTAAGGACGCGGCCCCGTGCG**A**ACACAGCCCGGGTGCCCCCCCGTGCTCGA 111

B4_BF2_(AM282699) TCC**A**CCGGGGTAA**A**GACGCGGCCCCGTGCGGACACAGCCCGGGTGCCCCCCCGTGCTCGA 111

B4_BF2_(PacBio_C) TCC**A**CCGGGGTAA**A**GACGCGGCCCCGTGCGGACACAGCCCGGGTGCCCCCCCGTGCTCGA 234

B4_BF2_(AM282693) TCC**A**CCGGGGTAA**A**GACGCGGCCCCGTGCGGACACAGCCCGGGTGCCCCCCCGTGCTCGA 111

B12_BF2_(AB426147) TCCCCCGGGGTAAGGACGCGGCCCCGTGCG**A**ACACAGCCCGGGTGCCCCCCCGTGCTCGA 133

B12_BF2_(PacBio_C) TCCCCCGGGGTAAGGACGCGGCCCCGTGCG**A**ACACAGCCCGGGTGCCCCCCCGTGCTCGA 237

B12_BF2_(AL023516) TCCCCCGGGGTAAGGACGCGGCCCCGTGCG**A**ACACAGCCCGGGTGCCCCCCCGTGCTCGA 237

B14_BF2_(PacBio_WL) TCCCCCGGGGTAA**A**GACGCGGCCCCGTGCGGACACAGCCCGGGTGCCCCCCCGTGCTCGA 239

B14_BF2_(AM282694) TCCCCCGGGGTAA**A**GACGCGGCCCCGTGCGGACACAGCCCGGGTGCCCCCCCGTGCTCGA 111

B15_BF2_(AB426149) TCC**A**CCGGGGTAA**A**GAC**A**CGGCCCCGTGCGGACACAGCCCGGGTGCCCCCCCGTGCTCGA 122

B15_BF2_(PacBio_15I) TCC**A**CCGGGGTAA**A**GAC**A**CGGCCCCGTGCGGACACAGCCCGGGTGCCCCCCCGTGCTCGA 237

B15_BF2_(AM282695) TCC**A**CCGGGGTAA**A**GAC**A**CGGCCCCGTGCGGACACAGCCCGGGTGCCCCCCCGTGCTCGA 111

B19_BF2_(AB426151) TCCCCCGGGGTAAGGACGCGGCCCCGTGCGGACACAGCCCGGGTGCCCCCCCGTGCTCGA 133

B19_BF2_(PacBio_P2a) TCCCCCGGGGTAAGGACGCGGCCCCGTGCGGACACAGCCCGGGTGCCCCCCCGTGCTCGA 237

B19_BF2_(AM282696) TCCCCCGGGGTAAGGACGCGGCCCCGTGCGGACACAGCCCGGGTGCCCCCCCGTGCTCGA 111

B21_BF2_(AB426152) TCC**A**CCGGGGTAA**A**GAC**A**CGGCCCCGTGCGGACACAGCCCGGGTGCCCCCCCGTGCTCGA 133

B21_BF2_(PacBio_N) TCC**A**CCGGGGTAA**A**GAC**A**CGGCCCCGTGCGGACACAGCCCGGGTGCCCCCCCGTGCTCGA 237

B21_BF2_(AM282697) TCC**A**CCGGGGTAA**A**GAC**A**CGGCCCCGTGCGGACACAGCCCGGGTGCCCCCCCGTGCTCGA 111

B21_BF2_(PacBio_0) TCC**A**CCGGGGTAA**A**GAC**A**CGGCCCCGTGCGGACACAGCCCGGGTGCCCCCCCGTGCTCGA 133

B21_BF2_(AM282700) TCC**A**CCGGGGTAA**A**GAC**A**CGGCCCCGTGCGGACACAGCCCGGGTGCCCCCCCGTGCTCGA 111

**--enhancer A---** **----IRE----**

B2_BF2_(AB426141) AGGGCCGGGGGTTCCCACACCGCGCCCATCCCCTCCCCCGCTCCGCGCTTTCGCTTTCGC 193

B2_BF2_(PacBio_6sub1) AGGGCCGGGGGTTCCCACACCGCGCCCATCCCCTCCCCCGCTCCGCGCTTTCGCTTTCGC 297

B2_BF2_(AM282692) AGGGCCGGGGGTTCCCACACCGCGCCCATCCCCTCCCCCGCTCCGCGCTTTCGCTTTCGC 171

B2_BF2_(PacBio_7sub2) AGGGCCGGGGGTTCCCACACCGCGCCCATCCCCTCCCCCGCTCCGCGCTTTCGCTTTCGC 297

B2_BF2_(AM282698) AGGGCCGGGGGTTCCCACACCGCGCCCATCCCCTCCCCCGCTCCGCGCTTTCGCTTTCGC 171

B4_BF2_(AM282699) AGGGCCGGGGGTTCCCACACCGCGCCCATCCCCTCCCCCGCTCCGCGCTTTCGCTTTCGC 171

B4_BF2_(PacBio_C) AGGGCCGGGGGTTCCCACACCGCGCCCATCCCCTCCCCCGCTCCGCGCTTTCGCTTTCGC 294

B4_BF2_(AM282693) AGGGCCGGGGGTTCCCACACCGCGCCCATCCCCTCCCCCGCTCCGCGCTTTCGCTTTCGC 171

B12_BF2_(AB426147) AGGGCCGGGGGTTCCCACACCGCGCCCATCCCCTCCCCCGCTCCGCGCTTTCGCTTTCGC 193

B12_BF2_(PacBio_C) AGGGCCGGGGGTTCCCACACCGCGCCCATCCCCTCCCCCGCTCCGCGCTTTCGCTTTCGC 297

B12_BF2_(AL023516) AGGGCCGGGGGTTCCCACACCGCGCCCATCCCCTCCCCCGCTCCGCGCTTTCGCTTTCGC 297

B14_BF2_(PacBio_WL) AGGGCCGGGGGTTCCCACACCGCGCCCATCCCCTCCCCCGCTCCGCGCTTTCGCTTTCGC 299

B14_BF2_(AM282694) AGGGCCGGGGGTTCCCACACCGCGCCCATCCCCTCCCCCGCTCCGCGCTTTCGCTTTCGC 171

B15_BF2_(AB426149) AGGGCCGGGGGTTCCCACACCGCGCCCATCCCCTCCCCCGCTCCGCGCTTTCGCTTTCGC 182

B15_BF2_(PacBio_15I) AGGGCCGGGGGTTCCCACACCGCGCCCATCCCCTCCCCCGCTCCGCGCTTTCGCTTTCGC 297

B15_BF2_(AM282695) AGGGCCGGGGGTTCCCACACCGCGCCCATCCCCTCCCCCGCTCCGCGCTTTCGCTTTCGC 171

B19_BF2_(AB426151) AGGGCCGGGGGTTCCCACACCGCGCCCATCCCCTCCCCCGCTCCGCGCTTTCGCTTTCGC 193

B19_BF2_(PacBio_P2a) AGGGCCGGGGGTTCCCACACCGCGCCCATCCCCTCCCCCGCTCCGCGCTTTCGCTTTCGC 297

B19_BF2_(AM282696) AGGGCCGGGGGTTCCCACACCGCGCCCATCCCCTCCCCCGCTCCGCGCTTTCGCTTTCGC 171

B21_BF2_(AB426152) AGGGCCGGGGGTTCCCACACCGCGCCCATCCCCTCCCCCGCTCCGCGCTTTCGCTTTCGC 193

B21_BF2_(PacBio_N) AGGGCCGGGGGTTCCCACACCGCGCCCATCCCCTCCCCCGCTCCGCGCTTTCGCTTTCGC 297

B21_BF2_(AM282697) AGGGCCGGGGGTTCCCACACCGCGCCCATCCCCTCCCCCGCTCCGCGCTTTCGCTTTCGC 171

B21_BF2_(PacBio_0) AGGGCCGGGGGTTCCCACACCGCGCCCATCCCCTCCCCCGCTCCGCGCTTTCGCTTTCGC 193

B21_BF2_(AM282700) AGGGCCGGGGGTTCCCACACCGCGCCCATCCCCTCCCCCGCTCCGCGCTTTCGCTTTCGC 171

**--(s)--** **----s----** **--------X/X2---------**

B2_BF2_(AB426141) TTCACAACCTGAGGGAGCGCATTCTGCCTGGCGCCCGATGACGTCACATAAAACTCCAAC 253

B2_BF2_(PacBio_6sub1) TTCACAACCTGAGGGAGCGCATTCTGCCTGGCGCCCGATGACGTCACATAAAACTCCAAC 357

B2_BF2_(AM282692) TTCACAACCTGAGGGAGCGCATTCTGCCTGGCGCCCGATGACGTCACATAAAACTCCAAC 231

B2_BF2_(PacBio_7sub2) TTCACAACCTGAGGGAGCGCATTCTGCCTGGCGCCCGATGACGTCACATAAAACTCCAAC 357

B2_BF2_(AM282698) TTCACAACCTGAGGGAGCGCATTCTGCCTGGCGCCCGATGACGTCACATAAAACTCCAAC 231

B4_BF2_(AM282699) TTCACAACCTGAGGGAGCGCATTCTGCCTGGCGCCCGATGACGTCACATAAA**C**CTCC**G**AC 231

B4_BF2_(PacBio_C) TTCACAACCTGAGGGAGCGCATTCTGCCTGGCGCCCGATGACGTCACATAAA**C**CTCC**G**AC 354

B4_BF2_(AM282693) TTCACAACCTGAGGGAGCGCATTCTGCCTGGCGCCCGATGACGTCACATAAA**C**CTCC**G**AC 231

B12_BF2_(AB426147) TTCACAACCTGAGGGAGCGCATTCTGCCTGGCGCCCGATGACGTCACATAAAACTCCAAC 253

B12_BF2_(PacBio_C) TTCACAACCTGAGGGAGCGCATTCTGCCTGGCGCCCGATGACGTCACATAAAACTCCAAC 357

B12_BF2_(AL023516) TTCACAACCTGAGGGAGCGCATTCTGCCTGGCGCCCGATGACGTCACATAAAACTCCAAC 357

B14_BF2_(PacBio_WL) TTCACAACCTGAGGGAGCGCATTCTGCCTGGCGCCCGATGACGTCACATAAAACTCCAAC 359

B14_BF2_(AM282694) TTCACAACCTGAGGGAGCGCATTCTGCCTGGCGCCCGATGACGTCACATAAAACTCCAAC 231

B15_BF2_(AB426149) TTCACAACCTGAGGGAGC**C**CATTCTGCCTGGCGCCCGATGACGTCACATAAA**C**CTCC**G**AC 242

B15_BF2_(PacBio_15I) TTCACAACCTGAGGGAGC**C**CATTCTGCCTGGCGCCCGATGACGTCACATAAA**C**CTCC**G**AC 357

B15_BF2_(AM282695) TTCACAACCTGAGGGAGC**C**CATTCTGCCTGGCGCCCGATGACGTCACATAAA**C**CTCC**G**AC 231

B19_BF2_(AB426151) TTCACAACCTGAGGGAGCGCATTCTGCCTGGCGCCCGATGACGTCACATAAA**C**CTC**AG**AC 253

B19_BF2_(PacBio_P2a) TTCACAACCTGAGGGAGCGCATTCTGCCTGGCGCCCGATGACGTCACATAAA**C**CTC**AG**AC 357

B19_BF2_(AM282696) TTCACAACCTGAGGGAGCGCATTCTGCCTGGCGCCCGATGACGTCACATAAA**C**CTC**AG**AC 231

B21_BF2_(AB426152) TTCACAACCTGAGGGAGCGCATTCTGCCTGGCGCCCGATGACGTCACATAA**G**ACTCCAAC 253

B21_BF2_(PacBio_N) TTCACAACCTGAGGGAGCGCATTCTGCCTGGCGCCCGATGACGTCACATAA**G**ACTCCAAC 357

B21_BF2_(AM282697) TTCACAACCTGAGGGAGCGCATTCTGCCTGGCGCCCGATGACGTCACATAA**G**ACTCCAAC 231

B21_BF2_(PacBio_0) TTCACAACCTGAGGGAGCGCATTCTGCCTGGCGCCCGATGACGTCACATAA**G**ACTCCAAC 253

Fig. S2

B21_BF2_(AM282700) TTCACAACCTGAGGGAGCGCATTCTGCCTGGCGCCCGATGACGTCACATAA**G**ACTCCAAC 231

**---Y---**

B2_BF2_(AB426141) TACCATTGGCGGAGAGGCGACGGAGGAGCCAATGGGGGCGCGGGGCGGGGCGGAGGAGTA 313

B2_BF2_(PacBio_6sub1) TACCATTGGCGGAGAGGCGACGGAGGAGCCAATGGGGGCGCGGGGCGGGGCGGAGGAGTA 417

B2_BF2_(AM282692) TACCATTGGCGGAGAGGCGACGGAGGAGCCAATGGGGGCGCGGGGCGGGGCGGAGGAGTA 291

B2_BF2_(PacBio_7sub2) TACCATTGGCGGAGAGGCGACGGAGGAGCCAATGGGGGCGCGGGGCGGGGCGGAGGAGTA 417

B2_BF2_(AM282698) TACCATTGGCGGAGAGGCGACGGAGGAGCCAATGGGGGCGCGGGGCGGGGCGGAGGAGTA 291

B4_BF2_(AM282699) TACCATTGGCGGAGAGGCGACG**A**AGGAGCCAATGGGGGCGCGGGGCGGGGCGGAGGAGTA 291

B4_BF2_(PacBio_C) TACCATTGGCGGAGAGGCGACG**A**AGGAGCCAATGGGGGCGCGGGGCGGGGCGGAGGAGTA 414

B4_BF2_(AM282693) TACCATTGGCGGAGAGGCGACG**A**AGGAGCCAATGGGGGCGCGGGGCGGGGCGGAGGAGTA 291

B12_BF2_(AB426147) TACCATTGGCGGAGAGGCGACGGAGGAGCCAATGGGGGCGCGGGGCGGGGCGGAGGAGTA 313

B12_BF2_(PacBio_C) TACCATTGGCGGAGAGGCGACGGAGGAGCCAATGGGGGCGCGGGGCGGGGCGGAGGAGTA 417

B12_BF2_(AL023516) TACCATTGGCGGAGAGGCGACGGAGGAGCCAATGGGGGCGCGGGGCGGGGCGGAGGAGTA 417

B14_BF2_(PacBio_WL) TACCATTGGCGGAGAGGCGACGGAGGAGCCAATGGGGGCGCGGGGCGGGGCGGAGGAGTA 419

B14_BF2_(AM282694) TACCATTGGCGGAGAGGCGACGGAGGAGCCAATGGGGGCGCGGGGCGGGGCGGAGGAGTA 291

B15_BF2_(AB426149) TACCATTGGCGGAGAGGCGACG**A**AGGAGCCAATGGGGGCGCGGGGCGGGGCGGAGGAGTA 302

B15_BF2_(PacBio_15I) TACCATTGGCGGAGAGGCGACG**A**AGGAGCCAATGGGGGCGCGGGGCGGGGCGGAGGAGTA 417

B15_BF2_(AM282695) TACCATTGGCGGAGAGGCGACG**A**AGGAGCCAATGGGGGCGCGGGGCGGGGCGGAGGAGTA 291

B19_BF2_(AB426151) TACCATTGGCGGAGAGGCGACG**A**AGGAGCCAATGGGGGCGCGGGGCGGGGCGGAGGAGTA 313

B19_BF2_(PacBio_P2a) TACCATTGGCGGAGAGGCGACG**A**AGGAGCCAATGGGGGCGCGGGGCGGGGCGGAGGAGTA 417

B19_BF2_(AM282696) TACCATTGGCGGAGAGGCGACG**A**AGGAGCCAATGGGGGCGCGGGGCGGGGCGGAGGAGTA 291

B21_BF2_(AB426152) TACCATTGGCGGAGAGGCGACGGAGGAGCCAATGGGGGCGCGGGGCGGGGCGGAGGAGTA 313

B21_BF2_(PacBio_N) TACCATTGGCGGAGAGGCGACGGAGGAGCCAATGGGGGCGCGGGGCGGGGCGGAGGAGTA 417

B21_BF2_(AM282697) TACCATTGGCGGAGAGGCGACGGAGGAGCCAATGGGGGCGCGGGGCGGGGCGGAGGAGTA 291

B21_BF2_(PacBio_0) TACCATTGGCGGAGAGGCGACGGAGGAGCCAATGGGGGCGCGGGGCGGGGCGGAGGAGTA 313

B21_BF2_(AM282700) TACCATTGGCGGAGAGGCGACGGAGGAGCCAATGGGGGCGCGGGGCGGGGCGGAGGAGTA 291

B2_BF2_(AB426141) GGAAAAGCTGAAGGAGCTGCGCTGGGTGCGGCGGACTTGAGAGTGCAGCGGTGCGAGGCG 373

B2_BF2_(PacBio_6sub1) GGAAAAGCTGAAGGAGCTGCGCTGGGTGCGGCGGACTTGAGAGTGCAGCGGTGCGAGGCG 477

B2_BF2_(AM282692) GGAAAAGCTGAAGGAGCTGCGCTGGGTGCGGCGGACTTGAGAGTGCAGCGGTGCGAGGCG 351

B2_BF2_(PacBio_7sub2) GGAAAAGCTGAAGGAGCTGCGCTGGGTGCGGCGGACTTGAGAGTGCAGCGGTGCGAGGCG 477

B2_BF2_(AM282698) GGAAAAGCTGAAGGAGCTGCGCTGGGTGCGGCGGACTTGAGAGTGCAGCGGTGCGAGGCG 351

B4_BF2_(AM282699) GGAAAAGCTGAAGGAGCTGCGC**G**GGGTGCGGCGGACTTGAGAGTGCAGCGGTG**T**GAGGCG 351

B4_BF2_(PacBio_C) GGAAAAGCTGAAGGAGCTGCGC**G**GGGTGCGGCGGACTTGAGAGTGCAGCGGTG**T**GAGGCG 474

B4_BF2_(AM282693) GGAAAAGCTGAAGGAGCTGCGC**G**GGGTGCGGCGGACTTGAGAGTGCAGCGGTG**T**GAGGCG 351

B12_BF2_(AB426147) GGAAAAGCTGAAGGAGCTGCGCTGGGTGCGGCGGACTTGAGAGTGCAGCGGTGCGAGGCG 373

B12_BF2_(PacBio_C) GGAAAAGCTGAAGGAGCTGCGCTGGGTGCGGCGGACTTGAGAGTGCAGCGGTGCGAGGCG 477

B12_BF2_(AL023516) GGAAAAGCTGAAGGAGCTGCGCTGGGTGCGGCGGACTTGAGAGTGCAGCGGTGCGAGGCG 477

B14_BF2_(PacBio_WL) GGAAAAGCTGAAGGAGC**C**GCGCTGGGTGCGGCGGACTTGAGAGTGCAGCGGTGCGAGGCG 479

B14_BF2_(AM282694) GGAAAAGCTGAAGGAGC**C**GCGCTGGGTGCGGCGGACTTGAGAGTGCAGCGGTGCGAGGCG 351

B15_BF2_(AB426149) GGAAAAGCTGAAGGAGCTGCGCTGGGTGCGGCGGACTTGAGAGTGCAGCGGTG**T**GAGGCG 362

B15_BF2_(PacBio_15I) GGAAAAGCTGAAGGAGCTGCGCTGGGTGCGGCGGACTTGAGAGTGCAGCGGTG**T**GAGGCG 477

B15_BF2_(AM282695) GGAAAAGCTGAAGGAGCTGCGCTGGGTGCGGCGGACTTGAGAGTGCAGCGGTG**T**GAGGCG 351

B19_BF2_(AB426151) GGAAAAGCTGAAGGAGCTGCGCTGGGTGCGGCGGACTTGAGAGTGCAGCGGTGCGAGGCG 373

B19_BF2_(PacBio_P2a) GGAAAAGCTGAAGGAGCTGCGCTGGGTGCGGCGGACTTGAGAGTGCAGCGGTGCGAGGCG 477

B19_BF2_(AM282696) GGAAAAGCTGAAGGAGCTGCGCTGGGTGCGGCGGACTTGAGAGTGCAGCGGTGCGAGGCG 351

B21_BF2_(AB426152) GGAAAAGCTGAAGGAGCTGCGCTGGGTGCGGCGGACTTGAGAGTGCAGCGGTGCGAGGCG 373

B21_BF2_(PacBio_N) GGAAAAGCTGAAGGAGCTGCGCTGGGTGCGGCGGACTTGAGAGTGCAGCGGTGCGAGGCG 477

B21_BF2_(AM282697) GGAAAAGCTGAAGGAGCTGCGCTGGGTGCGGCGGACTTGAGAGTGCAGCGGTGCGAGGCG 351

B21_BF2_(PacBio_0) GGAAAAGCTGAAGGAGCTGCGCTGGGTGCGGCGGACTTGAGAGTGCAGCGGTGCGAGGCG 373

B21_BF2_(AM282700) GGAAAAGCTGAAGGAGCTGCGCTGGGTGCGGCGGACTTGAGAGTGCAGCGGTGCGAGGCG 351

**BF2 Start**

B2_BF2_(AB426141) ATGGGGCCGTGCGGGGCGCTGGGCCTG------GGGCTGCTGCTCGCCGCCGTGTGCGGG 427

B2_BF2_(PacBio_6sub1) ATGGGGCCGTGCGGGGCGCTGGGCCTG------GGGCTGCTGCTCGCCGCCGTGTGCGGG 531

B2_BF2_(AM282692) ATGGGGCCGTGCGGGGCGCTGGGCCTG------GGGCTGCTGCTCGCCGCCGTGTGCGGG 405

B2_BF2_(PacBio_7sub2) ATGGGGCCGTGCGGGGCGCTGGGCCTG------GGGCTGCTGCTCGCCGCCGTGTGCGGG 531

B2_BF2_(AM282698) ATGGGGCCGTGCGGGGCGCTGGGCCTG------GGGCTGCTGCTCGCCGCCGTGTGCGGG 405

B4_BF2_(AM282699) ATGGGGCCGTGCGGGGCGCTGGGCCTG------GGGCTGCTGCTCGCCGCCGTGTGCGGG 405

B4_BF2_(PacBio_C) ATGGGGCCGTGCGGGGCGCTGGGCCTG------GGGCTGCTGCTCGCCGCCGTGTGCGGG 528

B4_BF2_(AM282693) ATGGGGCCGTGCGGGGCGCTGGGCCTG------GGGCTGCTGCTCGCCGCCGTGTGCGGG 405

B12_BF2_(AB426147) ATGGGGCCGTGCGGGGCGCTGGGCCTG------GGGCTGCTGCTCGCCGCCGTGTGCGGG 427

B12_BF2_(PacBio_C) ATGGGGCCGTGCGGGGCGCTGGGCCTG------GGGCTGCTGCTCGCCGCCGTGTGCGGG 531

B12_BF2_(AL023516) ATGGGGCCGTGCGGGGCGCTGGGCCTG------GGGCTGCTGCTCGCCGCCGTGTGCGGG 531

B14_BF2_(PacBio_WL) ATGGGGCCGTGCGGGGCGCTGGGCCTG**GGGCTG**GGGCTGCTGCTCGCCGCCGTGTGCGGG 539

B14_BF2_(AM282694) ATGGGGCCGTGCGGGGCGCTGGGCCTG**GGGCTG**GGGCTGCTGCTCGCCGCCGTGTGCGGG 411

B15_BF2_(AB426149) ATGGGGCCGTGCGGGGCGCTGGGCCTG------GGGCTGCTGCTCG**G**CGCCGTGTGCGGG 416

B15_BF2_(PacBio_15I) ATGGGGCCGTGCGGGGCGCTGGGCCTG------GGGCTGCTGCTCG**G**CGCCGTGTGCGGG 531

B15_BF2_(AM282695) ATGGGGCCGTGCGGGGCGCTGGGCCTG------GGGCTGCTGCTCG**G**CGCCGTGTGCGGG 405

B19_BF2_(AB426151) ATGGGGCCGTGCGGGGCGCTGGGCCTG------GGGCTGCTGCTCGCCGCCGTGTGCGGG 427

B19_BF2_(PacBio_P2a) ATGGGGCCGTGCGGGGCGCTGGGCCTG------GGGCTGCTGCTCGCCGCCGTGTGCGGG 531

B19_BF2_(AM282696) ATGGGGCCGTGCGGGGCGCTGGGCCTG------GGGCTGCTGCTCGCCGCCGTGTGCGGG 405

B21_BF2_(AB426152) ATGGG**CT**CGTGCGGGGCGCTGGGCCTG------GGGCTGCTGCTCGCCGCCGTGTGCGGG 427

B21_BF2_(PacBio_N) ATGGG**CT**CGTGCGGGGCGCTGGGCCTG------GGGCTGCTGCTCGCCGCCGTGTGCGGG 531

B21_BF2_(AM282697) ATGGG**CT**CGTGCGGGGCGCTGGGCCTG------GGGCTGCTGCTCGCCGCCGTGTGCGGG 405

Fig. S2

B21_BF2_(PacBio_0) ATGGG**CT**CGTGCGGGGCGCTGGGCCTG------GGGCTGCTGCTCGCCGCCGTGTGCGGG 427

B21_BF2_(AM282700) ATGGG**CT**CGTGCGGGGCGCTGGGCCTG------GGGCTGCTGCTCGCCGCCGTGTGCGGG 405

B2_BF2_(AB426141) GCGGCGGCCGGTGAGTGCGGCCGGACCGGGACCCCTCCCCGCCCGTAACCCCACCCCGGG 487

B2_BF2_(PacBio_6sub1) GCGGCGGCCGGTGAGTGCGGCCGGACCGGGACCCCTCCCCGCCCGTAACCCCACCCCGGG 591

B2_BF2_(AM282692) GCGGCGGCCGGTGAGTGCGGCCGGACCGGGACCCCTCCCCGCCCGTAACCCCACCCCGGG 465

B2_BF2_(PacBio_7sub2) GCGGCGGCCGGTGAGTGCGGCCGGACCGGGACCCCTCCCCGCCCGTAACCCCACCCCGGG 591

B2_BF2_(AM282698) GCGGCGGCCGGTGAGTGCGGCCGGACCGGGACCCCTCCCCGCCCGTAACCCCACCCCGGG 465

B4_BF2_(AM282699) GCGGCGGCCGGTGAGTGCGGCCGGACCGGGACCCCTCCCCGCCCGTAACCCCACCCCGGG 465

B4_BF2_(PacBio_C) GCGGCGGCCGGTGAGTGCGGCCGGACCGGGACCCCTCCCCGCCCGTAACCCCACCCCGGG 588

B4_BF2_(AM282693) GCGGCGGCCGGTGAGTGCGGCCGGACCGGGACCCCTCCCCGCCCGTAACCCCACCCCGGG 465

B12_BF2_(AB426147) GCGGCGGCCGGTGAGTGCGGCCGGACCGGGACCCCTCCCCGCCCGTAACCCCACCCCGGG 487

B12_BF2_(PacBio_C) GCGGCGGCCGGTGAGTGCGGCCGGACCGGGACCCCTCCCCGCCCGTAACCCCACCCCGGG 591

B12_BF2_(AL023516) GCGGCGGCCGGTGAGTGCGGCCGGACCGGGACCCCTCCCCGCCCGTAACCCCACCCCGGG 591

B14_BF2_(PacBio_WL) GCGGCGGCCGGTGAGTGCGGCCGGACCGGGACCCCTCCCCGCCCGTAACCCCACCCCGGG 599

B14_BF2_(AM282694) GCGGCGGCCGGTGAGTGCGGCCGGACCGGGACCCCTCCCCGCCCGTAACCCCACCCCGGG 471

B15_BF2_(AB426149) GCGGCGGCCGGTGAGTGCGGCCGGACCGGGACCCCTCCCCGCCCGTAACCCCACCCCGGG 476

B15_BF2_(PacBio_15I) GCGGCGGCCGGTGAGTGCGGCCGGACCGGGACCCCTCCCCGCCCGTAACCCCACCCCGGG 591

B15_BF2_(AM282695) GCGGCGGCCGGTGAGTGCGGCCGGACCGGGACCCCTCCCCGCCCGTAACCCCACCCCGGG 465

B19_BF2_(AB426151) **A**CGGCGGCCGGTGAGTGCGGCCGGACCGGGACCCCTCCCCGCCCGTAACCCCACCCCGGG 487

B19_BF2_(PacBio_P2a) **A**CGGCGGCCGGTGAGTGCGGCCGGACCGGGACCCCTCCCCGCCCGTAACCCCACCCCGGG 591

B19_BF2_(AM282696) **A**CGGCGGCCGGTGAGTGCGGCCGGACCGGGACCCCTCCCCGCCCGTAACCCCACCCCGGG 465

B21_BF2_(AB426152) GCGGCGGCCGGTGAGTGCGGCCGGACCGGGACCCCTCCCCGCCC**A**TAACCCCACCCCGGG 487

B21_BF2_(PacBio_N) GCGGCGGCCGGTGAGTGCGGCCGGACCGGGACCCCTCCCCGCCC**A**TAACCCCACCCCGGG 591

B21_BF2_(AM282697) GCGGCGGCCGGTGAGTGCGGCCGGACCGGGACCCCTCCCCGCCC**A**TAACCCCACCCCGGG 465

B21_BF2_(PacBio_0) GCGGCGGCCGGTGAGTGCGGCCGGACCGGGACCCCTCCCCGCCC**A**TAACCCCACCCCGGG 487

B21_BF2_(AM282700) GCGGCGGCCGGTGAGTGCGGCCGGACCGGGACCCCTCCCCGCCC**A**TAACCCCACCCCGGG 465

B2_BF2_(AB426141) GCTGTG**T**CCGTGGGATCCTCAGACCCCCACCCGCGGCTCACGGCCCCGCTG**T**GCTCCGTC 547

B2_BF2_(PacBio_6sub1) GCTGTG**T**CCGTGGGATCCTCAGACCCCCACCCGCGGCTCACGGCCCCGCTG**T**GCTCCGTC 651

B2_BF2_(AM282692) GCTGTG**T**CCGTGGGATCCTCAGACCCCCACCCGCGGCTCACGGCCCCGCTG**T**GCTCCGTC 525

B2_BF2_(PacBio_7sub2) GCTGTG**T**CCGTGGGATCCTCAGACCCCCACCCGCGGCTCACGGCCCCGCTG**T**GCTCCGTC 651

B2_BF2_(AM282698) GCTGTG**T**CCGTGGGATCCTCAGACCCCCACCCGCGGCTCACGGCCCCGCTG**T**GCTCCGTC 525

B4_BF2_(AM282699) GCTGTGCCCGTGGGATCCTCAGACCCCCACCCGCGGCTCACGGCCCCGCTGCGCTCCGTC 525

B4_BF2_(PacBio_C) GCTGTGCCCGTGGGATCCTCAGACCCCCACCCGCGGCTCACGGCCCCGCTGCGCTCCGTC 648

B4_BF2_(AM282693) GCTGTGCCCGTGGGATCCTCAGACCCCCACCCGCGGCTCACGGCCCCGCTGCGCTCCGTC 525

B12_BF2_(AB426147) GCTGTG**T**CCGTGGGATCCTCAGACCC**A**CACCCGCGGCTCACGGCCCCGCTG**T**GCTCCGTC 547

B12_BF2_(PacBio_C) GCTGTGTCCGTGGGATCCTCAGACCC**A**CACCCGCGGCTCACGGCCCCGCTG**T**GCTCCGTC 651

B12_BF2_(AL023516) GCTGTGTCCGTGGGATCCTCAGACCCACACCCGCGGCTCACGGCCCCGCTG**T**GCTCCGTC 651

B14_BF2_(PacBio_WL) GCTGTGCCCGTGGGATCCTCAGACCCCCACCCGCGGCTCACGGCCCCGCTGCGCTCCGTC 659

B14_BF2_(AM282694) GCTGTGCCCGTGGGATCCTCAGACCCCCACCCGCGGCTCACGGCCCCGCTGCGCTCCGTC 531

B15_BF2_(AB426149) GCTGTGCCCGTGGGATCCTCAGACCCCCACCCGCGGCTCACGGCCCCGCTGCGCTCCGTC 536

B15_BF2_(PacBio_15I) GCTGTGCCCGTGGGATCCTCAGACCCCCACCCGCGGCTCACGGCCCCGCTGCGCTCCGTC 651

B15_BF2_(AM282695) GCTGTGCCCGTGGGATCCTCAGACCCCCACCCGCGGCTCACGGCCCCGCTGCGCTCCGTC 525

B19_BF2_(AB426151) GCTGTGCCCGTGGGATCCTCAGACCCCCACCCGCGGCTCACGGCCCCGCTGCGCTCCGTC 547

B19_BF2_(PacBio_P2a) GCTGTGCCCGTGGGATCCTCAGACCCCCACCCGCGGCTCACGGCCCCGCTGCGCTCCGTC 651

B19_BF2_(AM282696) GCTGTGCCCGTGGGATCCTCAGACCCCCACCCGCGGCTCACGGCCCCGCTGCGCTCCGTC 525

B21_BF2_(AB426152) GCTGTG**T**CCGTGGGATCCTCAGACCCCCACCCGCGGCTCACGGCCCCGCTGCGCTCCGTC 547

B21_BF2_(PacBio_N) GCTGTG**T**CCGTGGGATCCTCAGACCCCCACCCGCGGCTCACGGCCCCGCTGCGCTCCGTC 651

B21_BF2_(AM282697) GCTGTG**T**CCGTGGGATCCTCAGACCCCCACCCGCGGCTCACGGCCCCGCTGCGCTCCGTC 525

B21_BF2_(PacBio_0) GCTGTG**T**CCGTGGGATCCTCAGACCCCCACCCGCGGCTCACGGCCCCGCTGCGCTCCGTC 547

B21_BF2_(AM282700) GCTGTG**T**CCGTGGGATCCTCAGACCCCCACCCGCGGCTCACGGCCCCGCTGCGCTCCGTC 525

**BF2 exon 2**

B2_BF2_(AB426141) CCCGCAGAGCTCCATACCCTGCGGTACATCCGTACGGCGATGACGGATCCCGGCCCCGGG 607

B2_BF2_(PacBio_6sub1) CCCGCAGAGCTCCATACCCTGCGGTACATCCGTACGGCGATGACGGATCCCGGCCCCGGG 711

B2_BF2_(AM282692) CCCGCAGAGCTCCATACCCTGCGGTACATCCGTACGGCGATGACGGATCCCGGCCCCGGG 585

B2_BF2_(PacBio_7sub2) CCCGCAGAGCTCCATACCCTGCGGTACATCCGTACGGCGATGACGGATCCCGGCCCCGGG 711

B2_BF2_(AM282698) CCCGCAGAGCTCCATACCCTGCGGTACATCCGTACGGCGATGACGGATCCCGGCCCCGGG 585

B4_BF2_(AM282699) CCCGCAGAGCTCCATACCCTGCGGTACATCCGTACGGCGATGACGGATCCCGGCCCCGGG 585

B4_BF2_(PacBio_C) CCCGCAGAGCTCCATACCCTGCGGTACATCCGTACGGCGATGACGGATCCCGGCCCCGGG 708

B4_BF2_(AM282693) CCCGCAGAGCTCCATACCCTGCGGTACATCCGTACGGCGATGACGGATCCCGGCCCCGGG 585

B12_BF2_(AB426147) CCCGCAGAGCTCCATACCCTGCGGTACATCC**AA**ACGGCGATGACGGATCCCGGCCCCGGG 607

B12_BF2_(PacBio_C) CCCGCAGAGCTCCATACCCTGCGGTACATCC**AA**ACGGCGATGACGGATCCCGGCCCCGGG 711

B12_BF2_(AL023516) CCCGCAGAGCTCCATACCCTGCGGTACATCC**AA**ACGGCGATGACGGATCCCGGCCCCGGG 711

B14_BF2_(PacBio_WL) CCCGCAGAGCTCCATACCCTGCGGTACATCC**AA**ACGGCGATGACGGATCCCGGCCCCGGG 719

B14_BF2_(AM282694) CCCGCAGAGCTCCATACCCTGCGGTACATCC**AA**ACGGCGATGACGGATCCCGGCCCCGGG 591

B15_BF2_(AB426149) CCCGCAGAGCTCCATACCCTGCGGTACATC**TC**TACGGCGATGACGGATCCCGGCCCCGGG 596

B15_BF2_(PacBio_15I) CCCGCAGAGCTCCATACCCTGCGGTACATC**TC**TACGGCGATGACGGATCCCGGCCCCGGG 711

B15_BF2_(AM282695) CCCGCAGAGCTCCATACCCTGCGGTACATC**TC**TACGGCGATGACGGATCCCGGCCCCGGG 585

B19_BF2_(AB426151) CCCGCAGAGCTCCATACCCTGCGGTACATC**TC**TACGGCGATGACGGATCCCGGCCCCGGG 607

B19_BF2_(PacBio_P2a) CCCGCAGAGCTCCATACCCTGCGGTACATC**TC**TACGGCGATGACGGATCCCGGCCCCGGG 711

B19_BF2_(AM282696) CCCGCAGAGCTCCATACCCTGCGGTACATC**TC**TACGGCGATGACGGATCCCGGCCCCGGG 585

B21_BF2_(AB426152) CCCGCAGAGCTCCATACCCTGCGGTACATCCGTACGGCGATGACGGATCCCGGCCCCGGG 607

B21_BF2_(PacBio_N) CCCGCAGAGCTCCATACCCTGCGGTACATCCGTACGGCGATGACGGATCCCGGCCCCGGG 711

Fig. S2

B21_BF2_(AM282697) CCCGCAGAGCTCCATACCCTGCGGTACATCCGTACGGCGATGACGGATCCCGGCCCCGGG 585

B21_BF2_(PacBio_0) CCCGCAGAGCTCCATACCCTGCGGTACATCCGTACGGCGATGACGGATCCCGGCCCCGGG 607

B21_BF2_(AM282700) CCCGCAGAGCTCCATACCCTGCGGTACATCCGTACGGCGATGACGGATCCCGGCCCCGGG 585

B2_BF2_(AB426141) C**T**GCCGTGGT**A**CGTGGACGTGGGGTACGTGGACGGGGAACTCTTCGTGCACTACAACAGC 667

B2_BF2_(PacBio_6sub1) C**T**GCCGTGGT**A**CGTGGACGTGGGGTACGTGGACGGGGAACTCTTCGTGCACTACAACAGC 771

B2_BF2_(AM282692) C**T**GCCGTGGT**A**CGTGGACGTGGGGTACGTGGACGGGGAACTCTTCGTGCACTACAACAGC 645

B2_BF2_(PacBio_7sub2) C**T**GCCGTGGT**A**CGTGGACGTGGGGTACGTGGACGGGGAACTCTTCGTGCACTACAACAGC 771

B2_BF2_(AM282698) C**T**GCCGTGGT**A**CGTGGACGTGGGGTACGTGGACGGGGAACTCTTCGTGCACTACAACAGC 645

B4_BF2_(AM282699) CAGCCGTGGTTCGTG**ACT**GTGGGGTA**T**GTGGACGGGGAACTCTTCGTGCACTACAACAGC 645

B4_BF2_(PacBio_C) CAGCCGTGGTTCGTG**ACT**GTGGGGTA**T**GTGGACGGGGAACTCTTCGTGCACTACAACAGC 768

B4_BF2_(AM282693) CAGCCGTGGTTCGTG**ACT**GTGGGGTA**T**GTGGACGGGGAACTCTTCGTGCACTACAACAGC 645

B12_BF2_(AB426147) CAGCCGTGGTTCGTG**ACT**GTGGGGTACGTGGACGGGGAACTCTTCGTGCACTACAACAGC 667

B12_BF2_(PacBio_C) CAGCCGTGGTTCGTG**ACT**GTGGGGTACGTGGACGGGGAACTCTTCGTGCACTACAACAGC 771

B12_BF2_(AL023516) CAGCCGTGGTTCGTG**ACT**GTGGGGTACGTGGACGGGGAACTCTTCGTGCACTACAACAGC 771

B14_BF2_(PacBio_WL) CAGCCGTGGTTCGTG**ACT**GTGGGGTACGTGGACGGGGAACTCTTCGTGCACTACAACAGC 779

B14_BF2_(AM282694) CAGCCGTGGTTCGTG**ACT**GTGGGGTACGTGGACGGGGAACTCTTCGTGCACTACAACAGC 651

B15_BF2_(AB426149) CAGCCGTGGT**A**CGTGGACGTGGGGTACGTGGACGGGGAACTCTTC**A**CGCACTACAACAGC 656

B15_BF2_(PacBio_15I) CAGCCGTGGT**A**CGTGGACGTGGGGTACGTGGACGGGGAACTCTTC**A**CGCACTACAACAGC 771

B15_BF2_(AM282695) CAGCCGTGGT**A**CGTGGACGTGGGGTACGTGGACGGGGAACTCTTC**A**CGCACTACAACAGC 645

B19_BF2_(AB426151) CAGCCGTGGTTCGTGGACGTGGGGTACGTGGACGGGGAACTCTTC**AC**GCACTACAACAGC 667

B19_BF2_(PacBio_P2a) CAGCCGTGGTTCGTGGACGTGGGGTACGTGGACGGGGAACTCTTC**AC**GCACTACAACAGC 771

B19_BF2_(AM282696) CAGCCGTGGTTCGTGGACGTGGGGTACGTGGACGGGGAACTCTTC**AC**GCACTACAACAGC 645

B21_BF2_(AB426152) C**T**GCCGTGGTTCGTGGACGTGGGGTACGTGGACGGGGAACTCTTC**A**TGCACTACAACAGC 667

B21_BF2_(PacBio_N) C**T**GCCGTGGTTCGTGGACGTGGGGTACGTGGACGGGGAACTCTTC**A**TGCACTACAACAGC 771

B21_BF2_(AM282697) C**T**GCCGTGGTTCGTGGACGTGGGGTACGTGGACGGGGAACTCTTC**A**TGCACTACAACAGC 645

B21_BF2_(PacBio_0) C**T**GCCGTGGTTCGTGGACGTGGGGTACGTGGACGGGGAACTCTTC**A**TGCACTACAACAGC 667

B21_BF2_(AM282700) C**T**GCCGTGGTTCGTGGACGTGGGGTACGTGGACGGGGAACTCTTC**A**TGCACTACAACAGC 645

B2_BF2_(AB426141) ACCGCGCGGAGG**TAC**GTGCCCCGCACCGAGTGGATAGCGGCCAA**GG**CGGACCAGCAGTAC 727

B2_BF2_(PacBio_6sub1) ACCGCGCGGAGG**TAC**GTGCCCCGCACCGAGTGGATAGCGGCCAA**GG**CGGACCAGCAGTAC 831

B2_BF2_(AM282692) ACCGCGCGGAGG**TAC**GTGCCCCGCACCGAGTGGATAGCGGCCAA**GG**CGGACCAGCAGTAC 705

B2_BF2_(PacBio_7sub2) ACCGCGCGGAGG**TAC**GTGCCCCGCACCGAGTGGATAGCGGCCAA**GG**CGGACCAGCAGTAC 831

B2_BF2_(AM282698) ACCGCGCGGAGG**TAC**GTGCCCCGCACCGAGTGGATAGCGGCCAA**GG**CGGACCAGCAGTAC 705

B4_BF2_(AM282699) ACCGCGCGGAGG**TAC**GTGCCCCGCACCGAGTGGATAGCGGCCAACACGGACCAGCAGTAC 705

B4_BF2_(PacBio_C) ACCGCGCGGAGG**TAC**GTGCCCCGCACCGAGTGGATAGCGGCCAACACGGACCAGCAGTAC 828

B4_BF2_(AM282693) ACCGCGCGGAGG**TAC**GTGCCCCGCACCGAGTGGATAGCGGCCAACACGGACCAGCAGTAC 705

B12_BF2_(AB426147) ACCGCGCGGAGG**TAC**GTGCCCCGCACCGAGTGGATAGCGGCCAA**GG**CGGACCAGCAGTAC 727

B12_BF2_(PacBio_C) ACCGCGCGGAGG**TAC**GTGCCCCGCACCGAGTGGATAGCGGCCAA**GG**CGGACCAGCAGTAC 831

B12_BF2_(AL023516) ACCGCGCGGAGG**TAC**GTGCCCCGCACCGAGTGGATAGCGGCCAA**GG**CGGACCAGCAGTAC 831

B14_BF2_(PacBio_WL) ACCGCGCGGAGGG**T**TGTGCCCCGCACCGAGTGGAT**G**GCGGCCAACACGGACCAGCAGTAC 839

B14_BF2_(AM282694) ACCGCGCGGAGGG**T**TGTGCCCCGCACCGAGTGGAT**G**GCGGCCAACACGGACCAGCAGTAC 711

B15_BF2_(AB426149) ACCGCGCGGAGGGCTGTGCCCCGCACCGAGTGGATAGCGGCCAACACGGACCAGCAGTAC 716

B15_BF2_(PacBio_15I) ACCGCGCGGAGGGCTGTGCCCCGCACCGAGTGGATAGCGGCCAACACGGACCAGCAGTAC 831

B15_BF2_(AM282695) ACCGCGCGGAGGGCTGTGCCCCGCACCGAGTGGATAGCGGCCAACACGGACCAGCAGTAC 705

B19_BF2_(AB426151) ACCGCGCGGAGGGCTGTGCCCCGCACCGAGTGGATAGCGGCCAACACGGACCAGCAGTAC 727

B19_BF2_(PacBio_P2a) ACCGCGCGGAGGGCTGTGCCCCGCACCGAGTGGATAGCGGCCAACACGGACCAGCAGTAC 831

B19_BF2_(AM282696) ACCGCGCGGAGGGCTGTGCCCCGCACCGAGTGGATAGCGGCCAACACGGACCAGCAGTAC 705

B21_BF2_(AB426152) ACCGC**T**CGGAGGGCTGTGCCCCGCACCGAGTGGATAGCGGCCAACACGGACCAGCAGTAC 727

B21_BF2_(PacBio_N) ACCGC**T**CGGAGGGCTGTGCCCCGCACCGAGTGGATAGCGGCCAACACGGACCAGCAGTAC 831

B21_BF2_(AM282697) ACCGC**T**CGGAGGGCTGTGCCCCGCACCGAGTGGATAGCGGCCAACACGGACCAGCAGTAC 705

B21_BF2_(PacBio_0) ACCGC**T**CGGAGGGCTGTGCCCCGCACCGAGTGGATAGCGGCCAACACGGACCAGCAGTAC 727

B21_BF2_(AM282700) ACCGC**T**CGGAGGGCTGTGCCCCGCACCGAGTGGATAGCGGCCAACACGGACCAGCAGTAC 705

B2_BF2_(AB426141) TGGGATGGACAGACGCAGATCGGACAGGGCAATGAGCAGATTGACCGCGAGAACCTGGGC 787

B2_BF2_(PacBio_6sub1) TGGGATGGACAGACGCAGATCGGACAGGGCAATGAGCAGATTGACCGCGAGAACCTGGGC 891

B2_BF2_(AM282692) TGGGATGGACAGACGCAGATCGGACAGGGCAATGAGCAGATTGACCGCGAGAACCTGGGC 765

B2_BF2_(PacBio_7sub2) TGGGATGGACAGACGCAGATCGGACAGGGCAATGAGCAGATTGACCGCGAGAACCTGGGC 891

B2_BF2_(AM282698) TGGGATGGACAGACGCAGATCGGACAGGGCAATGAGCAGATTGACCGCGAGAACCTGGGC 765

B4_BF2_(AM282699) TGGGATGGACAGACGCAGATCGGACAG**CT**CAATGAGCAGATT**A**ACCGCGAGAACCTGGGC 765

B4_BF2_(PacBio_C) TGGGATGGACAGACGCAGATCGGACAG**CT**CAATGAGCAGATT**A**ACCGCGAGAACCTGGGC 888

B4_BF2_(AM282693) TGGGATGGACAGACGCAGATCGGACAG**CT**CAATGAGCAGATT**A**ACCGCGAGAACCTGGGC 765

B12_BF2_(AB426147) TGGGATGGACAGACGCAGATCGGACAGGGCAATGAGCAGATTGACCGCGAGAACCTGGGC 787

B12_BF2_(PacBio_C) TGGGATGGACAGACGCAGATCGGACAGGGCAATGAGCAGATTGACCGCGAGAACCTGGGC 891

B12_BF2_(AL023516) TGGGATGGACAGACGCAGATCGGACAGGGCAATGAGCAGATTGACCGCGAGAACCTGGGC 891

B14_BF2_(PacBio_WL) TGG**A**ATGGACAGACGCAGATCG**T**ACAGGGCAATGAGCAGATTGACCGCGA**TG**ACCTGGGC 899

B14_BF2_(AM282694) TGG**A**ATGGACAGACGCAGATCG**T**ACAGGGCAATGAGCAGATTGACCGCGA**TG**ACCTGGGC 771

B15_BF2_(AB426149) TGGGA**CA**G**TG**AGACGCAGA**C**C**TC**ACAG**C**GCA**C**TGAGCAGATTGACCGCGA**TGG**CCTGGGC 776

B15_BF2_(PacBio_15I) TGGGA**CA**G**TG**AGACGCAGA**C**C**TC**ACAG**C**GCA**C**TGAGCAGATTGACCGCGA**TGG**CCTGGGC 891

B15_BF2_(AM282695) TGGGA**CA**G**TG**AGACGCAGA**C**C**TC**ACAG**C**GCA**C**TGAGCAGATTGACCGCGA**TGG**CCTGGGC 765

B19_BF2_(AB426151) TGGGA**CA**G**TG**AGACGCAGA**C**C**TC**ACAG**C**GCA**G**TGAGCAGATTGACCGCGA**TGG**CCTGGGC 787

B19_BF2_(PacBio_P2a) TGGGA**CA**G**TG**AGACGCAGA**C**C**TC**ACAG**C**GCA**G**TGAGCAGATTGACCGCGA**TGG**CCTGGGC 891

B19_BF2_(AM282696) TGGGA**CA**G**TG**AGACGCAGA**C**C**TC**ACAG**C**GCA**G**TGAGCAGATTGACCGCGA**TGG**CCTGGGC 765

B21_BF2_(AB426152) TGGGA**CA**GA**G**AGACGCAGATCG**T**ACAGGGCA**G**TGAGCAGATT**A**ACCGCGAGAACCTGG**A**C 787

Fig. S2

B21_BF2_(PacBio_N) TGGGA**CA**GA**G**AGACGCAGATCG**T**ACAGGGCA**G**TGAGCAGATT**A**ACCGCGAGAACCTGG**A**C 891

B21_BF2_(AM282697) TGGGA**CA**GA**G**AGACGCAGATCG**T**ACAGGGCA**G**TGAGCAGATT**A**ACCGCGAGAACCTGG**A**C 765

B21_BF2_(PacBio_0) TGGGA**CA**GA**G**AGACGCAGATCG**T**ACAGGGCA**G**TGAGCAGATT**A**ACCGCGAGAACCTGG**A**C 787

B21_BF2_(AM282700) TGGGA**CA**GA**G**AGACGCAGATCG**T**ACAGGGCA**G**TGAGCAGATT**A**ACCGCGAGAACCTGG**A**C 765

B2_BF2_(AB426141) ATACTGCAGCGGCGCTACAACCAGACCGGCGGTGAGCACGGCCGGGGCCGCGGCTCCGTG 847

B2_BF2_(PacBio_6sub1) ATACTGCAGCGGCGCTACAACCAGACCGGCGGTGAGCACGGCCGGGGCCGCGGCTCCGTG 951

B2_BF2_(AM282692) ATACTGCAGCGGCGCTACAACCAGACCGGCGGTGAGCACGGCCGGGGCCGCGGCTCCGTG 825

B2_BF2_(PacBio_7sub2) ATACTGCAGCGGCGCTACAACCAGACCGGCGGTGAGCACGGCCGGGGCCGCGGCTCCGTG 951

B2_BF2_(AM282698) ATACTGCAGCGGCGCTACAACCAGACCGGCGGTGAGCACGGCCGGGGCCGCGGCTCCGTG 825

B4_BF2_(AM282699) ATAC**G**GCAGCGGCGCTACAACCAGAC**T**GGCGGTGAGCACGGCCGGGGCCGCGGCTCCGTG 825

B4_BF2_(PacBio_C) ATACGGCAGCGGCGCTACAACCAGAC**T**GGCGGTGAGCACGGCCGGGGCCGCGGCTCCGTG 948

B4_BF2_(AM282693) ATAC**G**GCAGCGGCGCTACAACCAGAC**T**GGCGGTGAGCACGGCCGGGGCCGCGGCTCCGTG 825

B12_BF2_(AB426147) ATACTGCAGCGGCGCTACAACCAGACCGGCGGTGAGCACGGCCGGGGCCGCGGCTCCGTG 847

B12_BF2_(PacBio_C) ATACTGCAGCGGCGCTACAACCAGACCGGCGGTGAGCACGGCCGGGGCCGCGGCTCCGTG 951

B12_BF2_(AL023516) ATACTGCAGCGGCGCTACAACCAGACCGGCGGTGAGCACGGCCGGGGCCGCGGCTCCGTG 951

B14_BF2_(PacBio_WL) A**C**ACTGCAGCGGCGCTACAACCAGACCGGCGGTGAGCACGGCCGGGGCCGCGGCTCCGTG 959

B14_BF2_(AM282694) A**C**ACTGCAGCGGCGCTACAACCAGACCGGCGGTGAGCACGGCCGGGGCCGCGGCTCCGTG 831

B15_BF2_(AB426149) A**C**ACTGCAGCGGCGCTACAACCAGACCGGCGGTGAGCACGGCCGGGGCCGCGGCTCCGTG 836

B15_BF2_(PacBio_15I) A**C**ACTGCAGCGGCGCTACAACCAGACCGGCGGTGAGCACGGCCGGGGCCGCGGCTCCGTG 951

B15_BF2_(AM282695) A**C**ACTGCAGCGGCGCTACAACCAGACCGGCGGTGAGCACGGCCGGGGCCGCGGCTCCGTG 825

B19_BF2_(AB426151) ATACTGCAGCGGCGCTACAACCAGACCGGCGGTGAGCACGGCCGGGGCCGCGGCTCCGTG 847

B19_BF2_(PacBio_P2a) ATACTGCAGCGGCGCTACAACCAGACCGGCGGTGAGCACGGCCGGGGCCGCGGCTCCGTG 951

B19_BF2_(AM282696) ATACTGCAGCGGCGCTACAACCAGACCGGCGGTGAGCACGGCCGGGGCCGCGGCTCCGTG 825

B21_BF2_(AB426152) ATACTGC**G**GCGGCGCTACAACCAGACCGGCGGTGAGCACGGCCGGGGCCGCGGCTCCGTG 847

B21_BF2_(PacBio_N) ATACTGC**G**GCGGCGCTACAACCAGACCGGCGGTGAGCACGGCCGGGGCCGCGGCTCCGTG 951

B21_BF2_(AM282697) ATACTGC**G**GCGGCGCTACAACCAGACCGGCGGTGAGCACGGCCGGGGCCGCGGCTCCGTG 825

B21_BF2_(PacBio_0) ATACTGC**G**GCGGCGCTACAACCAGACCGGCGGTGAGCACGGCCGGGGCCGCGGCTCCGTG 847

B21_BF2_(AM282700) ATACTGC**G**GCGGCGCTACAACCAGACCGGCGGTGAGCACGGCCGGGGCCGCGGCTCCGTG 825

B2_BF2_(AB426141) GGTGTGGGATGGGCTCCATGGCGCAGTGCCGCCCACACCCCCCAGGCCTGGCCCTGCCCG 907

B2_BF2_(PacBio_6sub1) GGTGTGGGATGGGCTCCATGGCGCAGTGCCGCCCACACCCCCCAGGCCTGGCCCTGCCCG 1011

B2_BF2_(AM282692) GGTGTGGGATGGGCTCCATGGCGCAGTGCCGCCCACACCCCCCAGGCCTGGCCCTGCCCG 885

B2_BF2_(PacBio_7sub2) GGTGTGGGATGGGCTCCATGGCGCAGTGCCGCCCACACCCCCCAGGCCTGGCCCTGCCCG 1011

B2_BF2_(AM282698) GGTGTGGGATGGGCTCCATGGCGCAGTGCCGCCCACACCCCCCAGGCCTGGCCCTGCCCG 885

B4_BF2_(AM282699) GGTGTGGGATGGGCTCCATGGCGCAGTGCCGCCCACAC**T**CCCCAGGCCTGGCCCTGCCCG 885

B4_BF2_(PacBio_C) GGTGTGGGATGGGCTCCATGGCGCAGTGCCGCCCACAC**T**CCCCAGGCCTGGCCCTGCCCG 1008

B4_BF2_(AM282693) GGTGTGGGATGGGCTCCATGGCGCAGTGCCGCCCACAC**T**CCCCAGGCCTGGCCCTGCCCG 885

B12_BF2_(AB426147) GGTGTGGGATGGGCTCCATGGCGCAGTGCCGCCCACACCCCCCAGGCCTGGCCCTGCCCG 907

B12_BF2_(PacBio_C) GGTGTGGGATGGGCTCCATGGCGCAGTGCCGCCCACACCCCCCAGGCCTGGCCCTGCCCG 1011

B12_BF2_(AL023516) GGTGTGGGATGGGCTCCATGGCGCAGTGCCGCCCACACCCCCCAGGCCTGGCCCTGCCCG 1011

B14_BF2_(PacBio_WL) GGTGTGGGATGGGCTCCATGGCGCAGTGCCGCCCACACCCCCCAGGCCTGGCCCTGCCCG 1019

B14_BF2_(AM282694) GGTGTGGGATGGGCTCCATGGCGCAGTGCCGCCCACACCCCCCAGGCCTGGCCCTGCCCG 891

B15_BF2_(AB426149) GGTGTGGGATGGGCTCCATGGCGCAGTGCCGCCCACACCCCCCAGGCCTGGCCCTGCCCG 896

B15_BF2_(PacBio_15I) GGTGTGGGATGGGCTCCATGGCGCAGTGCCGCCCACACCCCCCAGGCCTGGCCCTGCCCG 1011

B15_BF2_(AM282695) GGTGTGGGATGGGCTCCATGGCGCAGTGCCGCCCACACCCCCCAGGCCTGGCCCTGCCCG 885

B19_BF2_(AB426151) GGTGTGGGATGGGCTCCATGGCGCAGTGCCGCCCACACCCCCCAGGCCTGGCCCTGCCCG 907

B19_BF2_(PacBio_P2a) GGTGTGGGATGGGCTCCATGGCGCAGTGCCGCCCACACCCCCCAGGCCTGGCCCTGCCCG 1011

B19_BF2_(AM282696) GGTGTGGGATGGGCTCCATGGCGCAGTGCCGCCCACACCCCCCAGGCCTGGCCCTGCCCG 885

B21_BF2_(AB426152) GGTGTGGGATGGGCTCCATGGCGCAGTGCCGCCCACACCCCCCAGGCCTGGCCCTGCCCG 907

B21_BF2_(PacBio_N) GGTGTGGGATGGGCTCCATGGCGCAGTGCCGCCCACACCCCCCAGGCCTGGCCCTGCCCG 1011

B21_BF2_(AM282697) GGTGTGGGATGGGCTCCATGGCGCAGTGCCGCCCACACCCCCCAGGCCTGGCCCTGCCCG 885

B21_BF2_(PacBio_0) GGTGTGGGATGGGCTCCATGGCGCAGTGCCGCCCACACCCCCCAGGCCTGGCCCTGCCCG 907

B21_BF2_(AM282700) GGTGTGGGATGGGCTCCATGGCGCAGTGCCGCCCACACCCCCCAGGCCTGGCCCTGCCCG 885

B2_BF2_(AB426141) GCGGCACCGTCCCGGGGCTGCCCGTCACAGCCCCACCGCGCTCGGGGTGCCGCGTCCCGG 967

B2_BF2_(PacBio_6sub1) GCGGCACCGTCCCGGGGCTGCCCGTCACAGCCCCACCGCGCTCGGGGTGCCGCGTCCCGG 1071

B2_BF2_(AM282692) GCGGCACCGTCCCGGGGCTGCCCGTCACAGCCCCACCGCGCTCGGGGTGCCGCGTCCCGG 945

B2_BF2_(PacBio_7sub2) GCGGCACCGTCCCGGGGCTGCCCGTCACAGCCCCACCGCGCTCGGGGTGCCGCGTCCCGG 1071

B2_BF2_(AM282698) GCGGCACCGTCCCGGGGCTGCCCGTCACAGCCCCACCGCGCTCGGGGTGCCGCGTCCCGG 945

B4_BF2_(AM282699) GCGGCACCGTCCCGGGGCTGCCCGTCACAGCCCCACCGCGCTCGGGGTGCCGCGTCCCGG 945

B4_BF2_(PacBio_C) GCGGCACCGTCCCGGGGCTGCCCGTCACAGCCCCACCGCGCTCGGGGTGCCGCGTCCCGG 1068

B4_BF2_(AM282693) GCGGCACCGTCCCGGGGCTGCCCGTCACAGCCCCACCGCGCTCGGGGTGCCGCGTCCCGG 945

B12_BF2_(AB426147) GCGGCACCGTCCCGGGGCTGCCCGTCACAGCCCCACCGCGCTCGGGGTGCCGCGTCCCGG 967

B12_BF2_(PacBio_C) GCGGCACCGTCCCGGGGCTGCCCGTCACAGCCCCACCGCGCTCGGGGTGCCGCGTCCCGG 1071

B12_BF2_(AL023516) GCGGCACCGTCCCGGGGCTGCCCGTCACAGCCCCACCGCGCTCGGGGTGCCGCGTCCCGG 1071

B14_BF2_(PacBio_WL) GCGGCACCGTCCCGGGGCTGCCCGTCACAGCCCCACCGCGCTCGGGGTGCCGCGTCCCGG 1079

B14_BF2_(AM282694) GCGGCACCGTCCCGGGGCTGCCCGTCACAGCCCCACCGCGCTCGGGGTGCCGCGTCCCGG 951

B15_BF2_(AB426149) GCGGCACCGTCCCGGGGCTGCCCGTCACAGCCCCACCGCGCTCGGGGTGCCGCGTCCCGG 956

B15_BF2_(PacBio_15I) GCGGCACCGTCCCGGGGCTGCCCGTCACAGCCCCACCGCGCTCGGGGTGCCGCGTCCCGG 1071

B15_BF2_(AM282695) GCGGCACCGTCCCGGGGCTGCCCGTCACAGCCCCACCGCGCTCGGGGTGCCGCGTCCCGG 945

B19_BF2_(AB426151) GCGGCACCGTCCCGGGGCTGCCCGTCACAGCCCCACCGCGCTCGGGGTGCCGCGTCCCGG 967

B19_BF2_(PacBio_P2a) GCGGCACCGTCCCGGGGCTGCCCGTCACAGCCCCACCGCGCTCGGGGTGCCGCGTCCCGG 1071

B19_BF2_(AM282696) GCGGCACCGTCCCGGGGCTGCCCGTCACAGCCCCACCGCGCTCGGGGTGCCGCGTCCCGG 945

B21_BF2_(AB426152) GCGGCACCGTCCCGGGGCTGCCCGTCACAGCCCCACCGCGCTCGGGGTGCCGCGTCCCGG 967

B21_BF2_(PacBio_N) GCGGCACCGTCCCGGGGCTGCCCGTCACAGCCCCACCGCGCTCGGGGTGCCGCGTCCCGG 1071

B21_BF2_(AM282697) GCGGCACCGTCCCGGGGCTGCCCGTCACAGCCCCACCGCGCTCGGGGTGCCGCGTCCCGG 945

B21_BF2_(PacBio_0) GCGGCACCGTCCCGGGGCTGCCCGTCACAGCCCCACCGCGCTCGGGGTGCCGCGTCCCGG 967

B21_BF2_(AM282700) GCGGCACCGTCCCGGGGCTGCCCGTCACAGCCCCACCGCGCTCGGGGTGCCGCGTCCCGG 945

Fig. S2

B2_BF2_(AB426141) GGGGACCCCAACCCATCCCCGCTGCAGTGGGAGCCCCGGAGCCGGAGGGGCCCCTCACCC 1027

B2_BF2_(PacBio_6sub1) GGGGACCCCAACCCATCCCCGCTGCAGTGGGAGCCCCGGAGCCGGAGGGGCCCCTCACCC 1131

B2_BF2_(AM282692) GGGGACCCCAACCCATCCCCGCTGCAGTGGGAGCCCCGGAGCCGGAGGGGCCCCTCACCC 1005

B2_BF2_(PacBio_7sub2) GGGGACCCCAACCCATCCCCGCTGCAGTGGGAGCCCCGGAGCCGGAGGGGCCCCTCACCC 1131

B2_BF2_(AM282698) GGGGACCCCAACCCATCCCCGCTGCAGTGGGAGCCCCGGAGCCGGAGGGGCCCCTCACCC 1005

B4_BF2_(AM282699) GGGGACCCCAACCCATCCCCGCTGCAGTGGGAGCCCCGGAGCCGGAGGGGCCCCTCACCC 1005

B4_BF2_(PacBio_C) GGGGACCCCAACCCATCCCCGCTGCAGTGGGAGCCCCGGAGCCGGAGGGGCCCCTCACCC 1128

B4_BF2_(AM282693) GGGGACCCCAACCCATCCCCGCTGCAGTGGGAGCCCCGGAGCCGGAGGGGCCCCTCACCC 1005

B12_BF2_(AB426147) GGGGACCCCAACCCATCCCCGCTGCAGTGGGAGCCCCGGAGCCGGAGGGGCCCCTCACCC 1027

B12_BF2_(PacBio_C) GGGGACCCCAACCCATCCCCGCTGCAGTGGGAGCCCCGGAGCCGGAGGGGCCCCTCACCC 1131

B12_BF2_(AL023516) GGGGACCCCAACCCATCCCCGCTGCAGTGGGAGCCCCGGAGCCGGAGGGGCCCCTCACCC 1131

B14_BF2_(PacBio_WL) GGGGACCCCAACCCATCCCCGCTGCAGTGGGAGCCCCGGAGCCGGAGGGGCCCCTCACCC 1139

B14_BF2_(AM282694) GGGGACCCCAACCCATCCCCGCTGCAGTGGGAGCCCCGGAGCCGGAGGGGCCCCTCACCC 1011

B15_BF2_(AB426149) GGGGACCCCAACCCATCCCCGCTGCAGTGGGAGCCCCGGAGCCGGAGGGGCCCCTCACCC 1016

B15_BF2_(PacBio_15I) GGGGACCCCAACCCATCCCCGCTGCAGTGGGAGCCCCGGAGCCGGAGGGGCCCCTCACCC 1131

B15_BF2_(AM282695) GGGGACCCCAACCCATCCCCGCTGCAGTGGGAGCCCCGGAGCCGGAGGGGCCCCTCACCC 1005

B19_BF2_(AB426151) GGGGACCCCAACCCATCCCCGCTGCAGTGGGAGCCCCGGAGCCGGAGGGGCCCCTCACCC 1027

B19_BF2_(PacBio_P2a) GGGGACCCCAACCCATCCCCGCTGCAGTGGGAGCCCCGGAGCCGGAGGGGCCCCTCACCC 1131

B19_BF2_(AM282696) GGGGACCCCAACCCATCCCCGCTGCAGTGGGAGCCCCGGAGCCGGAGGGGCCCCTCACCC 1005

B21_BF2_(AB426152) GGGGACCCCAACCCATCCCCGCTGCAGTGGGAGCCCCGGAGCCGGAGGGGCCCCTCACCC 1027

B21_BF2_(PacBio_N) GGGGACCCCAACCCATCCCCGCTGCAGTGGGAGCCCCGGAGCCGGAGGGGCCCCTCACCC 1131

B21_BF2_(AM282697) GGGGACCCCAACCCATCCCCGCTGCAGTGGGAGCCCCGGAGCCGGAGGGGCCCCTCACCC 1005

B21_BF2_(PacBio_0) GGGGACCCCAACCCATCCCCGCTGCAGTGGGAGCCCCGGAGCCGGAGGGGCCCCTCACCC 1027

B21_BF2_(AM282700) GGGGACCCCAACCCATCCCCGCTGCAGTGGGAGCCCCGGAGCCGGAGGGGCCCCTCACCC 1005

**BF2 exon 3**

B2_BF2_(AB426141) CCTGCCCGGCTGTGTTTCAGGGTCTCACACGGTGCAGTGGATGTACGGCTGTGACATCCT 1087

B2_BF2_(PacBio_6sub1) CCTGCCCGGCTGTGTTTCAGGGTCTCACACGGTGCAGTGGATGTACGGCTGTGACATCCT 1191

B2_BF2_(AM282692) CCTGCCCGGCTGTGTTTCAGGGTCTCACACGGTGCAGTGGATGTACGGCTGTGACATCCT 1065

B2_BF2_(PacBio_7sub2) CCTGCCCGGCTGTGTTTCAGGGTCTCACACGGTGCAGTGGATGTACGGCTGTGACATCCT 1191

B2_BF2_(AM282698) CCTGCCCGGCTGTGTTTCAGGGTCTCACACGGTGCAGTGGATGTACGGCTGTGACATCCT 1065

B4_BF2_(AM282699) CCTGCCCGGCTGTGTTTCAGGGTCTCACACGGTGCAGTGGATGTTCGGCTGTGACATCCT 1065

B4_BF2_(PacBio_C) CCTGCCCGGCTGTGTTTCAGGGTCTCACACGGTGCAGTGGATGTTCGGCTGTGACATCCT 1188

B4_BF2_(AM282693) CCTGCCCGGCTGTGTTTCAGGGTCTCACACGGTGCAGTGGATGTTCGGCTGTGACATCCT 1065

B12_BF2_(AB426147) CCTGCCCGGCTGTGTTTCAGGGTCTCACACGGTGCAGTGGATGTACGGCTGTGACATCCT 1087

B12_BF2_(PacBio_C) CCTGCCCGGCTGTGTTTCAGGGTCTCACACGGTGCAGTGGATGTACGGCTGTGACATCCT 1191

B12_BF2_(AL023516) CCTGCCCGGCTGTGTTTCAGGGTCTCACACGGTGCAGTGGATGTACGGCTGTGACATCCT 1191

B14_BF2_(PacBio_WL) CCTGCCCGGCTGTGTTTCAGGGTCTCACACGGTGCAG**CT**GATGTACGGCTGTGACATCCT 1199

B14_BF2_(AM282694) CCTGCCCGGCTGTGTTTCAGGGTCTCACACGGTGCAG**CT**GATGTACGGCTGTGACATCCT 1071

B15_BF2_(AB426149) CCTGCCCGGCTGTGTTTCAGGGTCTCACACGGTGCAG**CT**GATGTACGGCTGTGACATCCT 1076

B15_BF2_(PacBio_15I) CCTGCCCGGCTGTGTTTCAGGGTCTCACACGGTGCAG**CT**GATGTACGGCTGTGACATCCT 1191

B15_BF2_(AM282695) CCTGCCCGGCTGTGTTTCAGGGTCTCACACGGTGCAG**CT**GATGTACGGCTGTGACATCCT 1065

B19_BF2_(AB426151) CCTGCCCGGCTGTGTTTCAGGGTCTCACAC**A**GTGCAGTGGATGTACGGCTGTGACATCCT 1087

B19_BF2_(PacBio_P2a) CCTGCCCGGCTGTGTTTCAGGGTCTCACAC**A**GTGCAGTGGATGTACGGCTGTGACATCCT 1191

B19_BF2_(AM282696) CCTGCCCGGCTGTGTTTCAGGGTCTCACAC**A**GTGCAGTGGATGTACGGCTGTGACATCCT 1065

B21_BF2_(AB426152) CCTGCCCGGCTGTGTTTCAGGGTCTCACAC**A**GTGCAGTGGATGT**C**CGGCTGTGACATCCT 1087

B21_BF2_(PacBio_N) CCTGCCCGGCTGTGTTTCAGGGTCTCACAC**A**GTGCAGTGGATGT**C**CGGCTGTGACATCCT 1191

B21_BF2_(AM282697) CCTGCCCGGCTGTGTTTCAGGGTCTCACAC**A**GTGCAGTGGATGT**C**CGGCTGTGACATCCT 1065

B21_BF2_(PacBio_0) CCTGCCCGGCTGTGTTTCAGGGTCTCACAC**A**GTGCAGTGGATGT**C**CGGCTGTGACATCCT 1087

B21_BF2_(AM282700) CCTGCCCGGCTGTGTTTCAGGGTCTCACAC**A**GTGCAGTGGATGT**C**CGGCTGTGACATCCT 1065

B2_BF2_(AB426141) CGAGG**G**CGGC**C**CCATCCGGGGGTAT**TA**TCAG**ATG**GCCTACGATGGGAGAGACTTCA**C**TGC 1147

B2_BF2_(PacBio_6sub1) CGAGG**G**CGGC**C**CCATCCGGGGGTAT**TA**TCAG**ATG**GCCTACGATGGGAGAGACTTCA**C**TGC 1251

B2_BF2_(AM282692) CGAGG**G**CGGC**C**CCATCCGGGGGTAT**TA**TCAG**ATG**GCCTACGATGGGAGAGACTTCA**C**TGC 1125

B2_BF2_(PacBio_7sub2) CGAGG**G**CGGC**C**CCATCCGGGGGTAT**TA**TCAG**ATG**GCCTACGATGGGAGAGACTTCA**C**TGC 1251

B2_BF2_(AM282698) CGAGG**G**CGGC**C**CCATCCGGGGGTAT**TA**TCAG**ATG**GCCTACGATGGGAGAGACTTCA**C**TGC 1125

B4_BF2_(AM282699) CGAGGA**T**GGCACCATCCGGGGGTATCGTCAG**TCA**GCCTACGATGGGAGAGACTTCATTGC 1125

B4_BF2_(PacBio_C) CGAGGA**T**GGCACCATCCGGGGGTATCGTCAG**TCA**GCCTACGATGGGAGAGACTTCATTGC 1248

B4_BF2_(AM282693) CGAGGA**T**GGCACCATCCGGGGGTATCGTCAG**TCA**GCCTACGATGGGAGAGACTTCATTGC 1125

B12_BF2_(AB426147) CGAGG**G**CGGC**C**CCATCCGGGGGTAT**TA**TCAG**ATG**GCCTACGATGGGAGAGACTTCA**C**TGC 1147

B12_BF2_(PacBio_C) CGAGG**G**CGGC**C**CCATCCGGGGGTAT**TA**TCAG**ATG**GCCTACGATGGGAGAGACTTCA**C**TGC 1251

B12_BF2_(AL023516) CGAGG**G**CGGC**C**CCATCCGGGGGTAT**TA**TCAG**ATG**GCCTACGATGGGAGAGACTTCA**C**TGC 1251

B14_BF2_(PacBio_WL) CGAGGACGGCACCATCCGGGGGTAT**A**GTCAGGATGCCTACGATGGGAGAGACTTCATTGC 1259

B14_BF2_(AM282694) CGAGGACGGCACCATCCGGGGGTAT**A**GTCAGGATGCCTACGATGGGAGAGACTTCATTGC 1131

B15_BF2_(AB426149) CGAGGACGGCACCATCCGGGGGTAT**A**GTCAGGATGCCTACGATGGGAGAGACTTCATTGC 1136

B15_BF2_(PacBio_15I) CGAGGACGGCACCATCCGGGGGTAT**A**GTCAGGATGCCTACGATGGGAGAGACTTCATTGC 1251

B15_BF2_(AM282695) CGAGGACGGCACCATCCGGGGGTAT**A**GTCAGGATGCCTACGATGGGAGAGACTTCATTGC 1125

B19_BF2_(AB426151) CGAGGACGGCACCATCCGGGGGTATCGTCAG**T**ATGCCTACGATGGGAGAGACTTCATTGC 1147

B19_BF2_(PacBio_P2a) CGAGGACGGCACCATCCGGGGGTATCGTCAG**T**ATGCCTACGATGGGAGAGACTTCATTGC 1251

B19_BF2_(AM282696) CGAGGACGGCACCATCCGGGGGTATCGTCAG**T**ATGCCTACGATGGGAGAGACTTCATTGC 1125

B21_BF2_(AB426152) CGAGGA**T**GGCACCATCCGGGGGTATC**A**TCAGG**CA**GCCTACGATGGGAGAGACTTC**G**TTGC 1147

B21_BF2_(PacBio_N) CGAGGA**T**GGCACCATCCGGGGGTATC**A**TCAGG**CA**GCCTACGATGGGAGAGACTTC**G**TTGC 1251

B21_BF2_(AM282697) CGAGGA**T**GGCACCATCCGGGGGTATC**A**TCAGG**CA**GCCTACGATGGGAGAGACTTC**G**TTGC 1125

B21_BF2_(PacBio_0) CGAGGA**T**GGCACCATCCGGGGGTATC**A**TCAGG**CA**GCCTACGATGGGAGAGACTTC**G**TTGC 1147

B21_BF2_(AM282700) CGAGGA**T**GGCACCATCCGGGGGTATC**A**TCAGG**CA**GCCTACGATGGGAGAGACTTC**G**TTGC 1125

Fig. S2

B2_BF2_(AB426141) CTTCGACAAAGGCACGATGACGTTCACTGCGGCAGTTCCAGAGGCAGTTCCCACCAAGAG 1207

B2_BF2_(PacBio_6sub1) CTTCGACAAAGGCACGATGACGTTCACTGCGGCAGTTCCAGAGGCAGTTCCCACCAAGAG 1311

B2_BF2_(AM282692) CTTCGACAAAGGCACGATGACGTTCACTGCGGCAGTTCCAGAGGCAGTTCCCACCAAGAG 1185

B2_BF2_(PacBio_7sub2) CTTCGACAAAGGCACGATGACGTTCACTGCGGCAGTTCCAGAGGCAGTTCCCACCAAGAG 1311

B2_BF2_(AM282698) CTTCGACAAAGGCACGATGACGTTCACTGCGGCAGTTCCAGAGGCAGTTCCCACCAAGAG 1185

B4_BF2_(AM282699) C**C**TCGACAAAG**A**CA**T**GA**A**GACGTTCACTGCGGCAGTTCCAGAGGCAGTTCCCACCAAGAG 1185

B4_BF2_(PacBio_C) C**C**TCGACAAAG**A**CA**T**GA**A**GACGTTCACTGCGGCAGTTCCAGAGGCAGTTCCCACCAAGAG 1308

B4_BF2_(AM282693) C**C**TCGACAAAG**A**CA**T**GA**A**GACGTTCACTGCGGCAGTTCCAGAGGCAGTTCCCACCAAGAG 1185

B12_BF2_(AB426147) CTTCGACAAAGGCACGATGACGTTCACTGCGGCAGTTCCAGAGGCAGTTCCCACCAAGAG 1207

B12_BF2_(PacBio_C) CTTCGACAAAGGCACGATGACGTTCACTGCGGCAGTTCCAGAGGCAGTTCCCACCAAGAG 1311

B12_BF2_(AL023516) CTTCGACAAAGGCACGATGACGTTCACTGCGGCAGTTCCAGAGGCAGTTCCCACCAAGAG 1311

B14_BF2_(PacBio_WL) CTTCGACAAAGGCACGATGACGTTCACTGCGGCAGTTCCAGAGGCAGTTCCCACCAAGAG 1319

B14_BF2_(AM282694) CTTCGACAAAGGCACGATGACGTTCACTGCGGCAGTTCCAGAGGCAGTTCCCACCAAGAG 1191

B15_BF2_(AB426149) CTTCGACAAAG**A**CACGATGACGTTCACTGC**A**GCAGTTCCAGAGGCAGTTCCCACCAAGAG 1196

B15_BF2_(PacBio_15I) CTTCGACAAAG**A**CACGATGACGTTCACTGC**A**GCAGTTCCAGAGGCAGTTCCCACCAAGAG 1311

B15_BF2_(AM282695) CTTCGACAAAG**A**CACGATGACGTTCACTGC**A**GCAGTTCCAGAGGCAGTTCCCACCAAGAG 1185

B19_BF2_(AB426151) CTTCGACAAAGGCACGATGACGTTCACTGCGGCAGTTCCAGAGGCAGTTCCCACCAAGAG 1207

B19_BF2_(PacBio_P2a) CTTCGACAAAGGCACGATGACGTTCACTGCGGCAGTTCCAGAGGCAGTTCCCACCAAGAG 1311

B19_BF2_(AM282696) CTTCGACAAAGGCACGATGACGTTCACTGCGGCAGTTCCAGAGGCAGTTCCCACCAAGAG 1185

B21_BF2_(AB426152) CTTCGACAAAGGCACGATGACGTT**A**ACTGCGGCAGTTCCAGAGGCAGTTCCCACCAAGAG 1207

B21_BF2_(PacBio_N) CTTCGACAAAGGCACGATGACGTT**A**ACTGCGGCAGTTCCAGAGGCAGTTCCCACCAAGAG 1311

B21_BF2_(AM282697) CTTCGACAAAGGCACGATGACGTT**A**ACTGCGGCAGTTCCAGAGGCAGTTCCCACCAAGAG 1185

B21_BF2_(PacBio_0) CTTCGACAAAGGCACGATGACGTT**A**ACTGCGGCAGTTCCAGAGGCAGTTCCCACCAAGAG 1207

B21_BF2_(AM282700) CTTCGACAAAGGCACGATGACGTT**A**ACTGCGGCAGTTCCAGAGGCAGTTCCCACCAAGAG 1185

B2_BF2_(AB426141) GAAATGGGAGGAAGGAGATTATGCTGAGGGGCTGAAGCAGTACCTGGAGGAAACCTGCGT 1267

B2_BF2_(PacBio_6sub1) GAAATGGGAGGAAGGAGATTATGCTGAGGGGCTGAAGCAGTACCTGGAGGAAACCTGCGT 1371

B2_BF2_(AM282692) GAAATGGGAGGAAGGAGATTATGCTGAGGGGCTGAAGCAGTACCTGGAGGAAACCTGCGT 1245

B2_BF2_(PacBio_7sub2) GAAATGGGAGGAAGGAGATTATGCTGAGGGGCTGAAGCAGTACCTGGAGGAAACCTGCGT 1371

B2_BF2_(AM282698) GAAATGGGAGGAAGGAGATTATGCTGAGGGGCTGAAGCAGTACCTGGAGGAAACCTGCGT 1245

B4_BF2_(AM282699) GAAATGGGAGGAAG**AGAG**T**G**A**AC**CTGAG**A**GG**TG**GAAG**A**ATTACCTGGAGGAAACCTGCGT 1245

B4_BF2_(PacBio_C) GAAATGGGAGGAAG**AGAG**T**G**A**AC**CTGAG**A**GG**TG**GAAG**A**ATTACCTGGAGGAAACCTGCGT 1368

B4_BF2_(AM282693) GAAATGGGAGGAAG**AGAG**T**G**A**AC**CTGAG**A**GG**TG**GAAG**A**ATTACCTGGAGGAAACCTGCGT 1245

B12_BF2_(AB426147) GAAATGGGAGGAAG**AGAG**T**G**A**AC**CTGAG**A**GG**TG**GAAG**A**ATTACCTGGAGGAAACCTGCGT 1267

B12_BF2_(PacBio_C) GAAATGGGAGGAAG**AGAG**T**G**A**AC**CTGAG**A**GG**TG**GAAG**A**ATTACCTGGAGGAAACCTGCGT 1371

B12_BF2_(AL023516) GAAATGGGAGGAAG**AGAG**T**G**A**AC**CTGAG**A**GG**TG**GAAG**A**ATTACCTGGAGGAAACCTGCGT 1371

B14_BF2_(PacBio_WL) GAAATGGGAGGAAGGAGATTATGCTGAGGGGCTGAAGCAGTACCTGGAGGAAACCTGCGT 1379

B14_BF2_(AM282694) GAAATGGGAGGAAGGAGATTATGCTGAGGGGCTGAAGCAGTACCTGGAGGAAACCTGCGT 1251

B15_BF2_(AB426149) GAAATGGGAGGAAGGAGATTATGCTGAGGGGCTGAAGCAGTACCTGGAGGAAACCTGCGT 1256

B15_BF2_(PacBio_15I) GAAATGGGAGGAAGGAGATTATGCTGAGGGGCTGAAGCAGTACCTGGAGGAAACCTGCGT 1371

B15_BF2_(AM282695) GAAATGGGAGGAAGGAGATTATGCTGAGGGGCTGAAGCAGTACCTGGAGGAAACCTGCGT 1245

B19_BF2_(AB426151) GAAATGGGAGGAAGGAGATTATGCTGAGGGGCTGAAGCAGTACCTGGAGGAAACCTGCGT 1267

B19_BF2_(PacBio_P2a) GAAATGGGAGGAAGGAGATTATGCTGAGGGGCTGAAGCAGTACCTGGAGGAAACCTGCGT 1371

B19_BF2_(AM282696) GAAATGGGAGGAAGGAGATTATGCTGAGGGGCTGAAGCAGTACCTGGAGGAAACCTGCGT 1245

B21_BF2_(AB426152) GAAATGGGAGGAAGGAGGTTATGCTGAGGGGCTGAAGCAGTACCTGGAGGAAACCTGCGT 1267

B21_BF2_(PacBio_N) GAAATGGGAGGAAGGAGGTTATGCTGAGGGGCTGAAGCAGTACCTGGAGGAAACCTGCGT 1371

B21_BF2_(AM282697) GAAATGGGAGGAAGGAGGTTATGCTGAGGGGCTGAAGCAGTACCTGGAGGAAACCTGCGT 1245

B21_BF2_(PacBio_0) GAAATGGGAGGAAGGAGGTTATGCTGAGGGGCTGAAGCAGTACCTGGAGGAAACCTGCGT 1267

B21_BF2_(AM282700) GAAATGGGAGGAAGGAGGTTATGCTGAGGGGCTGAAGCAGTACCTGGAGGAAACCTGCGT 1245

B2_BF2_(AB426141) GGAGTGGCTGCGGAGATACGTGGAATACGGGAAGGCTGAGCTGGGCAGGAGAGGTGAGCG 1327

B2_BF2_(PacBio_6sub1) GGAGTGGCTGCGGAGATACGTGGAATACGGGAAGGCTGAGCTGGGCAGGAGAGGTGAGCG 1431

B2_BF2_(AM282692) GGAGTGGCTGCGGAGATACGTGGAATACGGGAAGGCTGAGCTGGGCAGGAGAGGTGAGCG 1305

B2_BF2_(PacBio_7sub2) GGAGTGGCTGCGGAGATACGTGGAATACGGGAAGGCTGAGCTGGGCAGGAGAGGTGAGCG 1431

B2_BF2_(AM282698) GGAGTGGCTGCGGAGATACGTGGAATACGGGAAGGCTGAGCTGGGCAGGAGAGGTGAGCG 1305

B4_BF2_(AM282699) GGAGTGGCTGCGGAGATACGTGGAATACGGGAAGGCTGAGCTGGGCAGGAGAGGTGAGCG 1305

B4_BF2_(PacBio_C) GGAGTGGCTGCGGAGATACGTGGAATACGGGAAGGCTGAGCTGGGCAGGAGAGGTGAGCG 1428

B4_BF2_(AM282693) GGAGTGGCTGCGGAGATACGTGGAATACGGGAAGGCTGAGCTGGGCAGGAGAGGTGAGCG 1305

B12_BF2_(AB426147) GGAGTGGCTGCGGAGATACGTGGAATACGGGAAGGCTGAGCTGGGCAGGAGAGGTGAGCG 1327

B12_BF2_(PacBio_C) GGAGTGGCTGCGGAGATACGTGGAATACGGGAAGGCTGAGCTGGGCAGGAGAGGTGAGCG 1431

B12_BF2_(AL023516) GGAGTGGCTGCGGAGATACGTGGAATACGGGAAGGCTGAGCTGGGCAGGAGAGGTGAGCG 1431

B14_BF2_(PacBio_WL) GGAGTGGCTGCGGAGATACGTGGAATACGGGAAGGCTGAGCTGGGCAGGAGAGGTGAGCG 1439

B14_BF2_(AM282694) GGAGTGGCTGCGGAGATACGTGGAATACGGGAAGGCTGAGCTGGGCAGGAGAGGTGAGCG 1311

B15_BF2_(AB426149) GGAGTGGCTGCGGAGATACGTGGAATA**T**GGGAAGGCTGAGCTGGGCAGGAGAGGTGAG**T**G 1316

B15_BF2_(PacBio_15I) GGAGTGGCTGCGGAGATACGTGGAATA**T**GGGAAGGCTGAGCTGGGCAGGAGAGGTGAG**T**G 1431

B15_BF2_(AM282695) GGAGTGGCTGCGGAGATACGTGGAATA**T**GGGAAGGCTGAGCTGGGCAGGAGAGGTGAG**T**G 1305

B19_BF2_(AB426151) GGAGTGGCTGCGGAGATACGTGGAATA**T**GGGAAGGCTGAGCTGGGCAGGAGAGGTGAG**T**G 1327

B19_BF2_(PacBio_P2a) GGAGTGGCTGCGGAGATACGTGGAATA**T**GGGAAGGCTGAGCTGGGCAGGAGAGGTGAG**T**G 1431

B19_BF2_(AM282696) GGAGTGGCTGCGGAGATACGTGGAATA**T**GGGAAGGCTGAGCTGGGCAGGAGAGGTGAG**T**G 1305

B21_BF2_(AB426152) GGAGTGGCTGCGGAGATA**T**GTGGAATACGGGAAGGCTGAGCTGGGCAGGAGAGGTGAGCG 1327

B21_BF2_(PacBio_N) GGAGTGGCTGCGGAGATA**T**GTGGAATACGGGAAGGCTGAGCTGGGCAGGAGAGGTGAGCG 1431

B21_BF2_(AM282697) GGAGTGGCTGCGGAGATA**T**GTGGAATACGGGAAGGCTGAGCTGGGCAGGAGAGGTGAGCG 1305

B21_BF2_(PacBio_0) GGAGTGGCTGCGGAGATA**T**GTGGAATACGGGAAGGCTGAGCTGGGCAGGAGAGGTGAGCG 1327

B21_BF2_(AM282700) GGAGTGGCTGCGGAGATA**T**GTGGAATACGGGAAGGCTGAGCTGGGCAGGAGAGGTGAGCG 1305

Fig. S2

B2_BF2_(AB426141) GGGTGGGGGGGGGAGCGGCTGCAGTGTGGGGCTGGACGTGGGGCGGGGGCTCAGCGTGGG 1387

B2_BF2_(PacBio_6sub1) GGGTGGGGGGGGGAGCGGCTGCAGTGTGGGGCTGGACGTGGGGCGGGGGCTCAGCGTGGG 1491

B2_BF2_(AM282692) GGGTGGGGGGGGGAGCGGCTGCAGTGTGGGGCTGGACGTGGGGCGGGGGCTCAGCGTGGG 1365

B2_BF2_(PacBio_7sub2) GGGTGGGGGGGGGAGCGGCTGCAGTGTGGGGCTGGACGTGGGGCGGGGGCTCAGCGTGGG 1491

B2_BF2_(AM282698) GGGTGGGGGGGGGAGCGGCTGCAGTGTGGGGCTGGACGTGGGGCGGGGGCTCAGCGTGGG 1365

B4_BF2_(AM282699) GGGTGGGGGGGGGAGCGGCTGCAGTGTGGGGCTGGACGTGGGGCGGGGGCTCAGCGTGGG 1365

B4_BF2_(PacBio_C) GGGTGGGGGGGGGAGCGGCTGCAGTGTGGGGCTGGACGTGGGGCGGGGGCTCAGCGTGGG 1488

B4_BF2_(AM282693) GGGTGGGGGGGGGAGCGGCTGCAGTGTGGGGCTGGACGTGGGGCGGGGGCTCAGCGTGGG 1365

B12_BF2_(AB426147) GGGTGGGGGGGGGAGCGGCTGCAGTGTGGGGCTGGACGTGGGGCGGGGGCTCAGCGTGGG 1387

B12_BF2_(PacBio_C) GGGTGGGGGGGGGAGCGGCTGCAGTGTGGGGCTGGACGTGGGGCGGGGGCTCAGCGTGGG 1491

B12_BF2_(AL023516) GGGTGGGGGGGGGAGCGGCTGCAGTGTGGGGCTGGACGTGGGGCGGGGGCTCAGCGTGGG 1491

B14_BF2_(PacBio_WL) GGGTGGGGGGGGGAGCGGCTGCAGTGTGGGGCTGGACGTGGGGCGGGGGCTCAGCGTGGG 1499

B14_BF2_(AM282694) GGGTGGGGGGGGGAGCGGCTGCAGTGTGGGGCTGGACGTGGGGCGGGGGCTCAGCGTGGG 1371

B15_BF2_(AB426149) GGGTGGGGG**--**G**AG**G**G**GGCTGC**G**GTGTGGGGCTG**A**ACGTGGGG**AA**GGGGCTCAGCGTGGG 1374

B15_BF2_(PacBio_15I) GGGTGGGGG**--**G**AG**G**G**GGCTGC**G**GTGTGGGGCTG**A**ACGTGGGG**AA**GGGGCTCAGCGTGGG 1489

B15_BF2_(AM282695) GGGTGGGGG**--**G**AG**G**G**GGCTGC**G**GTGTGGGGCTG**A**ACGTGGGG**AA**GGGGCTCAGCGTGGG 1363

B19_BF2_(AB426151) GGGTGGGGG**--**G**AG**G**G**GGCTGC**G**GTGTGGGGCTG**A**ACGTGGGG**AA**GGGGCTCAGCGTGGG 1385

B19_BF2_(PacBio_P2a) GGGTGGGGG**--**G**AG**G**G**GGCTGC**G**GTGTGGGGCTG**A**ACGTGGGG**AA**GGGGCTCAGCGTGGG 1489

B19_BF2_(AM282696) GGGTGGGGG**--**G**AG**G**G**GGCTGC**G**GTGTGGGGCTG**A**ACGTGGGG**AA**GGGGCTCAGCGTGGG 1363

B21_BF2_(AB426152) G**A**GTGGGGG**-----**G**G**GGC**C**GCAGTGTGGGGCTGGACGTGGGGCGGGGGCT**G**AGCGTGGG 1382

B21_BF2_(PacBio_N) G**A**GTGGGGG**-----**G**G**GGC**C**GCAGTGTGGGGCTGGACGTGGGGCGGGGGCT**G**AGCGTGGG 1486

B21_BF2_(AM282697) G**A**GTGGGGG**------G**GGC**C**GCAGTGTGGGGCTGGACGTGGGGCGGGGGCT**G**AGCGTGGG 1359

B21_BF2_(PacBio_0) G**A**GTGGGGG**-----**G**G**GGC**C**GCAGTGTGGGGCTGGACGTGGGGCGGGGGCT**G**AGCGTGGG 1382

B21_BF2_(AM282700) G**A**GTGGGGG**------G**GGC**C**GCAGTGTGGGGCTGGACGTGGGGCGGGGGCT**G**AGCGTGGG 1359

**BF2 exon 4**

B2_BF2_(AB426141) GAGCTCAGCCCGGCCCTCACTGCCGCCCGCCCGCAGAGCGGCCCGAGGTGCGAGTGTGGG 1447

B2_BF2_(PacBio_6sub1) GAGCTCAGCCCGGCCCTCACTGCCGCCCGCCCGCAGAGCGGCCCGAGGTGCGAGTGTGGG 1551

B2_BF2_(AM282692) GAGCTCAGCCCGGCCCTCACTGCCGCCCGCCCGCAGAGCGGCCCGAGGTGCGAGTGTGGG 1425

B2_BF2_(PacBio_7sub2) GAGCTCAGCCCGGCCCTCACTGCCGCCCGCCCGCAGAGCGGCCCGAGGTGCGAGTGTGGG 1551

B2_BF2_(AM282698) GAGCTCAGCCCGGCCCTCACTGCCGCCCGCCCGCAGAGCGGCCCGAGGTGCGAGTGTGGG 1425

B4_BF2_(AM282699) GAGCTCAGCCCGGCCCTCA**T**TGCC**A**CC**T**GCC**T**GCAGAGCGGCCCGAGGTGCGAGTGTGGG 1425

B4_BF2_(PacBio_C) GAGCTCAGCCCGGCCCTCA**T**TGCC**A**CC**T**GCC**T**GCAGAGCGGCCCGAGGTGCGAGTGTGGG 1548

B4_BF2_(AM282693) GAGCTCAGCCCGGCCCTCA**T**TGCC**A**CC**T**GCC**T**GCAGAGCGGCCCGAGGTGCGAGTGTGGG 1425

B12_BF2_(AB426147) GAGCTCAGCCCGGCCCTCACTGCCGCCCGCCCGCAGAGCGGCCCGAGGTGCGAGTGTGGG 1447

B12_BF2_(PacBio_C) GAGCTCAGCCCGGCCCTCACTGCCGCCCGCCCGCAGAGCGGCCCGAGGTGCGAGTGTGGG 1551

B12_BF2_(AL023516) GAGCTCAGCCCGGCCCTCACTGCCGCCCGCCCGCAGAGCGGCCCGAGGTGCGAGTGTGGG 1551

B14_BF2_(PacBio_WL) GAGCTCAGCCCGGCCCTCACTGCCGCCCGCCCGCAGAGCGGCCCGAGGTGCGAGTGTGGG 1559

B14_BF2_(AM282694) GAGCTCAGCCCGGCCCTCACTGCCGCCCGCCCGCAGAGCGGCCCGAGGTGCGAGTGTGGG 1431

B15_BF2_(AB426149) GAGCTCAGCCCGGCCCTCACTGCCGCCCGCC**T**GCAGAGCGGCCCGAGGTGCGAGTGTGGG 1434

B15_BF2_(PacBio_15I) GAGCTCAGCCCGGCCCTCACTGCCGCCCGCC**T**GCAGAGCGGCCCGAGGTGCGAGTGTGGG 1549

B15_BF2_(AM282695) GAGCTCAGCCCGGCCCTCACTGCCGCCCGCC**T**GCAGAGCGGCCCGAGGTGCGAGTGTGGG 1423

B19_BF2_(AB426151) GAGCTCAGCCCGGCCCTCACTGCCGCCCGCC**T**GCAGAGCGGCCCGAGGTGCGAGTGTGGG 1445

B19_BF2_(PacBio_P2a) GAGCTCAGCCCGGCCCTCACTGCCGCCCGCC**T**GCAGAGCGGCCCGAGGTGCGAGTGTGGG 1549

B19_BF2_(AM282696) GAGCTCAGCCCGGCCCTCACTGCCGCCCGCC**T**GCAGAGCGGCCCGAGGTGCGAGTGTGGG 1423

B21_BF2_(AB426152) GAGCTCAGCCCGGCCCTCACTGCCGCCCGCCCGCAGAGCG**A**CCCGAGGTGCGAGTGTGGG 1442

B21_BF2_(PacBio_N) GAGCTCAGCCCGGCCCTCACTGCCGCCCGCCCGCAGAGCG**A**CCCGAGGTGCGAGTGTGGG 1546

B21_BF2_(AM282697) GAGCTCAGCCCGGCCCTCACTGCCGCCCGCCCGCAGAGCG**A**CCCGAGGTGCGAGTGTGGG 1419

B21_BF2_(PacBio_0) GAGCTCAGCCCGGCCCTCACTGCCGCCCGCCCGCAGAGCG**A**CCCGAGGTGCGAGTGTGGG 1442

B21_BF2_(AM282700) GAGCTCAGCCCGGCCCTCACTGCCGCCCGCCCGCAGAGCG**A**CCCGAGGTGCGAGTGTGGG 1419

B2_BF2_(AB426141) GGAAGGAGGCCGACGGGATCCTGACCTTGTCCTGCCGCGCTCACGGCTTCTACCCGCGGC 1507

B2_BF2_(PacBio_6sub1) GGAAGGAGGCCGACGGGATCCTGACCTTGTCCTGCCGCGCTCACGGCTTCTACCCGCGGC 1611

B2_BF2_(AM282692) GGAAGGAGGCCGACGGGATCCTGACCTTGTCCTGCCGCGCTCACGGCTTCTACCCGCGGC 1485

B2_BF2_(PacBio_7sub2) GGAAGGAGGCCGACGGGATCCTGACCTTGTCCTGCCGCGCTCACGGCTTCTACCCGCGGC 1611

B2_BF2_(AM282698) GGAAGGAGGCCGACGGGATCCTGACCTTGTCCTGCCGCGCTCACGGCTTCTACCCGCGGC 1485

B4_BF2_(AM282699) GGAAGGAGGCCGACGGGATCCTGACCTTGTCCTGCCGCGCTCACGGCTTCTACCCGCGGC 1485

B4_BF2_(PacBio_C) GGAAGGAGGCCGACGGGATCCTGACCTTGTCCTGCCGCGCTCACGGCTTCTACCCGCGGC 1608

B4_BF2_(AM282693) GGAAGGAGGCCGACGGGATCCTGACCTTGTCCTGCCGCGCTCACGGCTTCTACCCGCGGC 1485

B12_BF2_(AB426147) GGAAGGAGGCCGACGGGATCCTGACCTTGTCCTGCCGCGCTCACGGCTTCTACCCGCGGC 1507

B12_BF2_(PacBio_C) GGAAGGAGGCCGACGGGATCCTGACCTTGTCCTGCCGCGCTCACGGCTTCTACCCGCGGC 1611

B12_BF2_(AL023516) GGAAGGAGGCCGACGGGATCCTGACCTTGTCCTGCCGCGCTCACGGCTTCTACCCGCGGC 1611

B14_BF2_(PacBio_WL) GGAAGGAGGCCGACGGGATCCTGACCTTGTCCTGCCGCGCTCACGGCTTCTACCCGCGGC 1619

B14_BF2_(AM282694) GGAAGGAGGCCGACGGGATCCTGACCTTGTCCTGCCGCGCTCACGGCTTCTACCCGCGGC 1491

B15_BF2_(AB426149) GGAAGGAGGCCGACGGGATCCTGACCTTGTCCTGCCGCGCTCACGGCTTCTACCCGCGGC 1494

B15_BF2_(PacBio_15I) GGAAGGAGGCCGACGGGATCCTGACCTTGTCCTGCCGCGCTCACGGCTTCTACCCGCGGC 1609

B15_BF2_(AM282695) GGAAGGAGGCCGACGGGATCCTGACCTTGTCCTGCCGCGCTCACGGCTTCTACCCGCGGC 1483

B19_BF2_(AB426151) GGAAGGAGGCCGACGGGATCCTGACCTTGTCCTGCCGCGCTCACGGCTTCTACCCGCGGC 1505

B19_BF2_(PacBio_P2a) GGAAGGAGGCCGACGGGATCCTGACCTTGTCCTGCCGCGCTCACGGCTTCTACCCGCGGC 1609

B19_BF2_(AM282696) GGAAGGAGGCCGACGGGATCCTGACCTTGTCCTGCCGCGCTCACGGCTTCTACCCGCGGC 1483

B21_BF2_(AB426152) GGAAGGAGGCCGACGGGATCCTGACCTTGTCCTGCCGCGCTCACGGCTTCTACCCGCGGC 1502

B21_BF2_(PacBio_N) GGAAGGAGGCCGACGGGATCCTGACCTTGTCCTGCCGCGCTCACGGCTTCTACCCGCGGC 1606

B21_BF2_(AM282697) GGAAGGAGGCCGACGGGATCCTGACCTTGTCCTGCCGCGCTCACGGCTTCTACCCGCGGC 1479

B21_BF2_(PacBio_0) GGAAGGAGGCCGACGGGATCCTGACCTTGTCCTGCCGCGCTCACGGCTTCTACCCGCGGC 1502

B21_BF2_(AM282700) GGAAGGAGGCCGACGGGATCCTGACCTTGTCCTGCCGCGCTCACGGCTTCTACCCGCGGC 1479

Fig. S2

B2_BF2_(AB426141) CCATCGTTGTCAGCTGGCTGAAGGACGGCGCGGTGCGGGGCCAGGACGCCCA**C**TCGGGGG 1567

B2_BF2_(PacBio_6sub1) CCATCGTTGTCAGCTGGCTGAAGGACGGCGCGGTGCGGGGCCAGGACGCCCA**C**TCGGGGG 1671

B2_BF2_(AM282692) CCATCGTTGTCAGCTGGCTGAAGGACGGCGCGGTGCGGGGCCAGGACGCCCA**C**TCGGGGG 1545

B2_BF2_(PacBio_7sub2) CCATCGTTGTCAGCTGGCTGAAGGACGGCGCGGTGCGGGGCCAGGACGCCCA**C**TCGGGGG 1671

B2_BF2_(AM282698) CCATCGTTGTCAGCTGGCTGAAGGACGGCGCGGTGCGGGGCCAGGACGCCCA**C**TCGGGGG 1545

B4_BF2_(AM282699) CCATCGTTGTCAGCTGGCTGAAGGACGGCGCGGTGCGGGGCCAGGACGCCCA**C**TCGGGGG 1545

B4_BF2_(PacBio_C) CCATCGTTGTCAGCTGGCTGAAGGACGGCGCGGTGCGGGGCCAGGACGCCCA**C**TCGGGGG 1668

B4_BF2_(AM282693) CCATCGTTGTCAGCTGGCTGAAGGACGGCGCGGTGCGGGGCCAGGACGCCCA**C**TCGGGGG 1545

B12_BF2_(AB426147) CCATCGTTGTCAGCTGGCTGAAGGACGGCGCGGTGCGGGGCCAGGACGCCCA**C**TCGGGGG 1567

B12_BF2_(PacBio_C) CCATCGTTGTCAGCTGGCTGAAGGACGGCGCGGTGCGGGGCCAGGACGCCCA**C**TCGGGGG 1671

B12_BF2_(AL023516) CCATCGTTGTCAGCTGGCTGAAGGACGGCGCGGTGCGGGGCCAGGACGCCCA**C**TCGGGGG 1671

B14_BF2_(PacBio_WL) CCATCGTTGTCAGCTGGCTGAAGGACGGCGCGGTGCGGGGCCAGGACGCCCAGTCGGGGG 1679

B14_BF2_(AM282694) CCATCGTTGTCAGCTGGCTGAAGGACGGCGCGGTGCGGGGCCAGGACGCCCAGTCGGGGG 1551

B15_BF2_(AB426149) CCATCG**CC**GTCAGCTGGCTGAAGGACGGCGCGGTGC**A**GGGCCAGGACGCCCAGTCGGGGG 1554

B15_BF2_(PacBio_15I) CCATCG**CC**GTCAGCTGGCTGAAGGACGGCGCGGTGC**A**GGGCCAGGACGCCCAGTCGGGGG 1669

B15_BF2_(AM282695) CCATCG**CC**GTCAGCTGGCTGAAGGACGGCGCGGTGC**A**GGGCCAGGACGCCCAGTCGGGGG 1543

B19_BF2_(AB426151) CCATCG**CC**GTCAGCTGGCTGAAGGACGGCGCGGTGCGGGGCCAGGACGCCCAGTCGGGGG 1565

B19_BF2_(PacBio_P2a) CCATCG**CC**GTCAGCTGGCTGAAGGACGGCGCGGTGCGGGGCCAGGACGCCCAGTCGGGGG 1669

B19_BF2_(AM282696) CCATCG**CC**GTCAGCTGGCTGAAGGACGGCGCGGTGCGGGGCCAGGACGCCCAGTCGGGGG 1543

B21_BF2_(AB426152) CCATCGTTGTCAGCTGGCTGAAGGACGGCGCGGTGCGGGGCCAGGACGCCCAGTCGGGGG 1562

B21_BF2_(PacBio_N) CCATCGTTGTCAGCTGGCTGAAGGACGGCGCGGTGCGGGGCCAGGACGCCCAGTCGGGGG 1666

B21_BF2_(AM282697) CCATCGTTGTCAGCTGGCTGAAGGACGGCGCGGTGCGGGGCCAGGACGCCCAGTCGGGGG 1539

B21_BF2_(PacBio_0) CCATCGTTGTCAGCTGGCTGAAGGACGGCGCGGTGCGGGGCCAGGACGCCCAGTCGGGGG 1562

B21_BF2_(AM282700) CCATCGTTGTCAGCTGGCTGAAGGACGGCGCGGTGCGGGGCCAGGACGCCCAGTCGGGGG 1539

B2_BF2_(AB426141) GCATCGTGCCCAACGGCGACGGCACCTACCACACCTGGGTCACCATCGATGCGCAGCCGG 1627

B2_BF2_(PacBio_6sub1) GCATCGTGCCCAACGGCGACGGCACCTACCACACCTGGGTCACCATCGATGCGCAGCCGG 1731

B2_BF2_(AM282692) GCATCGTGCCCAACGGCGACGGCACCTACCACACCTGGGTCACCATCGATGCGCAGCCGG 1605

B2_BF2_(PacBio_7sub2) GCATCGTGCCCAACGGCGACGGCACCTACCACACCTGGGTCACCATCGATGCGCAGCCGG 1731

B2_BF2_(AM282698) GCATCGTGCCCAACGGCGACGGCACCTACCACACCTGGGTCACCATCGATGCGCAGCCGG 1605

B4_BF2_(AM282699) GCATCGTGCCCAACGGCGACGGCACCTACCACACCTGGGTCACCATCGATGCGCAGCCGG 1605

B4_BF2_(PacBio_C) GCATCGTGCCCAACGGCGACGGCACCTACCACACCTGGGTCACCATCGATGCGCAGCCGG 1728

B4_BF2_(AM282693) GCATCGTGCCCAACGGCGACGGCACCTACCACACCTGGGTCACCATCGATGCGCAGCCGG 1605

B12_BF2_(AB426147) GCATCGTGCCCAACGGCGACGGCACCTACCACACCTGGGTCACCATCGATGCGCAGCCGG 1627

B12_BF2_(PacBio_C) GCATCGTGCCCAACGGCGACGGCACCTACCACACCTGGGTCACCATCGATGCGCAGCCGG 1731

B12_BF2_(AL023516) GCATCGTGCCCAACGGCGACGGCACCTACCACACCTGGGTCACCATCGATGCGCAGCCGG 1731

B14_BF2_(PacBio_WL) GCATCGTGCCCAACGGCGACGGCACCTACCACACCTGGGTCACCATCGATGCGCAGCCGG 1739

B14_BF2_(AM282694) GCATCGTGCCCAACGGCGACGGCACCTACCACACCTGGGTCACCATCGATGCGCAGCCGG 1611

B15_BF2_(AB426149) GCAT**T**GTGCCCAACGGCGACGGCACCTACCACACCTGGGTCACCATCGATGCGCAGCCGG 1614

B15_BF2_(PacBio_15I) GCAT**T**GTGCCCAACGGCGACGGCACCTACCACACCTGGGTCACCATCGATGCGCAGCCGG 1729

B15_BF2_(AM282695) GCAT**T**GTGCCCAACGGCGACGGCACCTACCACACCTGGGTCACCATCGATGCGCAGCCGG 1603

B19_BF2_(AB426151) GCATCGTGCCCAACGGCGACGGCACCTACCACACCTGGGTCACCATCGATGCGCAGCCGG 1625

B19_BF2_(PacBio_P2a) GCATCGTGCCCAACGGCGACGGCACCTACCACACCTGGGTCACCATCGATGCGCAGCCGG 1729

B19_BF2_(AM282696) GCATCGTGCCCAACGGCGACGGCACCTACCACACCTGGGTCACCATCGATGCGCAGCCGG 1603

B21_BF2_(AB426152) GCATCGTGCCCAACGGCGACGGCAC**G**TACCACACCTGGGTCACCATCGATGCGCAGCCGG 1622

B21_BF2_(PacBio_N) GCATCGTGCCCAACGGCGACGGCAC**G**TACCACACCTGGGTCACCATCGATGCGCAGCCGG 1726

B21_BF2_(AM282697) GCATCGTGCCCAACGGCGACGGCAC**G**TACCACACCTGGGTCACCATCGATGCGCAGCCGG 1599

B21_BF2_(PacBio_0) GCATCGTGCCCAACGGCGACGGCAC**G**TACCACACCTGGGTCACCATCGATGCGCAGCCGG 1622

B21_BF2_(AM282700) GCATCGTGCCCAACGGCGACGGCAC**G**TACCACACCTGGGTCACCATCGATGCGCAGCCGG 1599

B2_BF2_(AB426141) GGGACGGGGACAAGTACCAGTGCCGCGTGGAGCACGCCAGCCTGCCCCAGCCCGGCCTCT 1687

B2_BF2_(PacBio_6sub1) GGGACGGGGACAAGTACCAGTGCCGCGTGGAGCACGCCAGCCTGCCCCAGCCCGGCCTCT 1791

B2_BF2_(AM282692) GGGACGGGGACAAGTACCAGTGCCGCGTGGAGCACGCCAGCCTGCCCCAGCCCGGCCTCT 1665

B2_BF2_(PacBio_7sub2) GGGACGGGGACAAGTACCAGTGCCGCGTGGAGCACGCCAGCCTGCCCCAGCCCGGCCTCT 1791

B2_BF2_(AM282698) GGGACGGGGACAAGTACCAGTGCCGCGTGGAGCACGCCAGCCTGCCCCAGCCCGGCCTCT 1665

B4_BF2_(AM282699) GGGACGGGGACAAGTACCAGTGCCGCGTGGAGCACGCCAGCCTGCCCCAGCCCGGCCTCT 1665

B4_BF2_(PacBio_C) GGGACGGGGACAAGTACCAGTGCCGCGTGGAGCACGCCAGCCTGCCCCAGCCCGGCCTCT 1788

B4_BF2_(AM282693) GGGACGGGGACAAGTACCAGTGCCGCGTGGAGCACGCCAGCCTGCCCCAGCCCGGCCTCT 1665

B12_BF2_(AB426147) GGGACGGGGACAAGTACCAGTGCCGCGTGGAGCACGCCAGCCTGCCCCAGCCCGGCCTCT 1687

B12_BF2_(PacBio_C) GGGACGGGGACAAGTACCAGTGCCGCGTGGAGCACGCCAGCCTGCCCCAGCCCGGCCTCT 1791

B12_BF2_(AL023516) GGGACGGGGACAAGTACCAGTGCCGCGTGGAGCACGCCAGCCTGCCCCAGCCCGGCCTCT 1791

B14_BF2_(PacBio_WL) GGGACGGGGACAAGTACCAGTGCCGCGTGGAGCACGCCAGCCTGCCCCAGCCCGGCCTCT 1799

B14_BF2_(AM282694) GGGACGGGGACAAGTACCAGTGCCGCGTGGAGCACGCCAGCCTGCCCCAGCCCGGCCTCT 1671

B15_BF2_(AB426149) GGGACGGGGACAAGTACCAGTGCCGCGTGGAGCACGCCAGCCTGCCCCAGCCCGGCCTCT 1674

B15_BF2_(PacBio_15I) GGGACGGGGACAAGTACCAGTGCCGCGTGGAGCACGCCAGCCTGCCCCAGCCCGGCCTCT 1789

B15_BF2_(AM282695) GGGACGGGGACAAGTACCAGTGCCGCGTGGAGCACGCCAGCCTGCCCCAGCCCGGCCTCT 1663

B19_BF2_(AB426151) GGGACGGGGACAAGTACCAGTGCCGCGTGGAGCACGCCAGCCTGCCCCAGCCCGGCCTCT 1685

B19_BF2_(PacBio_P2a) GGGACGGGGACAAGTACCAGTGCCGCGTGGAGCACGCCAGCCTGCCCCAGCCCGGCCTCT 1789

B19_BF2_(AM282696) GGGACGGGGACAAGTACCAGTGCCGCGTGGAGCACGCCAGCCTGCCCCAGCCCGGCCTCT 1663

B21_BF2_(AB426152) GGGACGGGGACAAGTACCAGTGCCGCGTGGAGCACGCCAGCCTGCCCCAGCCCGGCCTCT 1682

B21_BF2_(PacBio_N) GGGACGGGGACAAGTACCAGTGCCGCGTGGAGCACGCCAGCCTGCCCCAGCCCGGCCTCT 1786

B21_BF2_(AM282697) GGGACGGGGACAAGTACCAGTGCCGCGTGGAGCACGCCAGCCTGCCCCAGCCCGGCCTCT 1659

B21_BF2_(PacBio_0) GGGACGGGGACAAGTACCAGTGCCGCGTGGAGCACGCCAGCCTGCCCCAGCCCGGCCTCT 1682

B21_BF2_(AM282700) GGGACGGGGACAAGTACCAGTGCCGCGTGGAGCACGCCAGCCTGCCCCAGCCCGGCCTCT 1659

Fig. S2

B2_BF2_(AB426141) ACTCGTGGGGTGAGTGAGGGGATGTGGGGCTGGGGGGCTGCGGGCTGCCCCTTCCCCTGC 1747

B2_BF2_(PacBio_6sub1) ACTCGTGGGGTGAGTGAGGGGATGTGGGGCTGGGGGGCTGCGGGCTGCCCCTTCCCCTGC 1851

B2_BF2_(AM282692) ACTCGTGGGGTGAGTGAGGGGATGTGGGGCTGGGGGGCTGCGGGCTGCCCCTTCCCCTGC 1725

B2_BF2_(PacBio_7sub2) ACTCGTGGGGTGAGTGAGGGGATGTGGGGCTGGGGGGCTGCGGGCTGCCCCTTCCCCTGC 1851

B2_BF2_(AM282698) ACTCGTGGGGTGAGTGAGGGGATGTGGGGCTGGGGGGCTGCGGGCTGCCCCTTCCCCTGC 1725

B4_BF2_(AM282699) ACTCGTGGGGTGAGTGAGGGGATGTGGGGCTGGGGGGCTGCGGGCTGCCCCTTCCCCTGC 1725

B4_BF2_(PacBio_C) ACTCGTGGGGTGAGTGAGGGGATGTGGGGCTGGGGGGCTGCGGGCTGCCCCTTCCCCTGC 1848

B4_BF2_(AM282693) ACTCGTGGGGTGAGTGAGGGGATGTGGGGCTGGGGGGCTGCGGGCTGCCCCTTCCCCTGC 1725

B12_BF2_(AB426147) ACTCGTGGGGTGAGTGAGGGGATGTGGGGCTGGGGGGCTGCGGGCTGCCCCTTCCCCTGC 1747

B12_BF2_(PacBio_C) ACTCGTGGGGTGAGTGAGGGGATGTGGGGCTGGGGGGCTGCGGGCTGCCCCTTCCCCTGC 1851

B12_BF2_(AL023516) ACTCGTGGGGTGAGTGAGGGGATGTGGGGCTGGGGGGCTGCGGGCTGCCCCTTCCCCTGC 1851

B14_BF2_(PacBio_WL) ACTCGTGGGGTGAGTGAGGGGATGTGGGGCTGGGGGGCTGCGGGCTGCCCCTTCCCCTGC 1859

B14_BF2_(AM282694) ACTCGTGGGGTGAGTGAGGGGATGTGGGGCTGGGGGGCTGCGGGCTGCCCCTTCCCCTGC 1731

B15_BF2_(AB426149) ACTC**A**TGGGGTGAGTGAGGGGATGTGGGGCTGGGGGGCTGCGGGCTGCCCCTTCCCCTGC 1734

B15_BF2_(PacBio_15I) ACTC**A**TGGGGTGAGTGAGGGGATGTGGGGCTGGGGGGCTGCGGGCTGCCCCTTCCCCTGC 1849

B15_BF2_(AM282695) ACTC**A**TGGGGTGAGTGAGGGGATGTGGGGCTGGGGGGCTGCGGGCTGCCCCTTCCCCTGC 1723

B19_BF2_(AB426151) ACTCGTGGGGTGAGTGAGGGGATGTGGGGCTGGGGGGCTGCGGGCTGCCCCTTCCCCTGC 1745

B19_BF2_(PacBio_P2a) ACTCGTGGGGTGAGTGAGGGGATGTGGGGCTGGGGGGCTGCGGGCTGCCCCTTCCCCTGC 1849

B19_BF2_(AM282696) ACTCGTGGGGTGAGTGAGGGGATGTGGGGCTGGGGGGCTGCGGGCTGCCCCTTCCCCTGC 1723

B21_BF2_(AB426152) ACTCGTGGGGTGAGTGAGGGGATGTGGGGCTGGGGGGCTGCGGGCTGCCCCTTCCCCTGC 1742

B21_BF2_(PacBio_N) ACTCGTGGGGTGAGTGAGGGGATGTGGGGCTGGGGGGCTGCGGGCTGCCCCTTCCCCTGC 1846

B21_BF2_(AM282697) ACTCGTGGGGTGAGTGAGGGGATGTGGGGCTGGGGGGCTGCGGGCTGCCCCTTCCCCTGC 1719

B21_BF2_(PacBio_0) ACTCGTGGGGTGAGTGAGGGGATGTGGGGCTGGGGGGCTGCGGGCTGCCCCTTCCCCTGC 1742

B21_BF2_(AM282700) ACTCGTGGGGTGAGTGAGGGGATGTGGGGCTGGGGGGCTGCGGGCTGCCCCTTCCCCTGC 1719

**BF2 exon 5**

B2_BF2_(AB426141) TGATGGCCCCGCTCTCCCCCAGAGCCGCCACAGCCCAACCTGGTGCCCATCGTGGCGGGG 1807

B2_BF2_(PacBio_6sub1) TGATGGCCCCGCTCTCCCCCAGAGCCGCCACAGCCCAACCTGGTGCCCATCGTGGCGGGG 1911

B2_BF2_(AM282692) TGATGGCCCCGCTCTCCCCCAGAGCCGCCACAGCCCAACCTGGTGCCCATCGTGGCGGGG 1785

B2_BF2_(PacBio_7sub2) TGATGGCCCCGCTCTCCCCCAGAGCCGCCACAGCCCAACCTGGTGCCCATCGTGGCGGGG 1911

B2_BF2_(AM282698) TGATGGCCCCGCTCTCCCCCAGAGCCGCCACAGCCCAACCTGGTGCCCATCGTGGCGGGG 1785

B4_BF2_(AM282699) TGATGGCCCCGCTCTCCCCCAGAGCCGCCACAGCCCAACCTGGTGCCCATCGTGGCGGGG 1785

B4_BF2_(PacBio_C) TGATGGCCCCGCTCTCCCCCAGAGCCGCCACAGCCCAACCTGGTGCCCATCGTGGCGGGG 1908

B4_BF2_(AM282693) TGATGGCCCCGCTCTCCCCCAGAGCCGCCACAGCCCAACCTGGTGCCCATCGTGGCGGGG 1785

B12_BF2_(AB426147) TGATGGCCCCGCTCTCCCCCAGAGCCGCCACAGCCCAACCTGGTGCCCATCGTGGCGGGG 1807

B12_BF2_(PacBio_C) TGATGGCCCCGCTCTCCCCCAGAGCCGCCACAGCCCAACCTGGTGCCCATCGTGGCGGGG 1911

B12_BF2_(AL023516) TGATGGCCCCGCTCTCCCCCAGAGCCGCCACAGCCCAACCTGGTGCCCATCGTGGCGGGG 1911

B14_BF2_(PacBio_WL) TGATGGCCCCGCTCTCCCCCAGAGCCGCCACAGCCCAACCTGGTGCCCATCGTGGCGGGG 1919

B14_BF2_(AM282694) TGATGGCCCCGCTCTCCCCCAGAGCCGCCACAGCCCAACCTGGTGCCCATCGTGGCGGGG 1791

B15_BF2_(AB426149) TGATGGCCCCGCTCTCCCCCAGAGCCGCCACAGCCCAACCTGGTGCCCATCGTGGCGGGG 1794

B15_BF2_(PacBio_15I) TGATGGCCCCGCTCTCCCCCAGAGCCGCCACAGCCCAACCTGGTGCCCATCGTGGCGGGG 1909

B15_BF2_(AM282695) TGATGGCCCCGCTCTCCCCCAGAGCCGCCACAGCCCAACCTGGTGCCCATCGTGGCGGGG 1783

B19_BF2_(AB426151) TGATGGCCCCGCTCTCCCCCAGAGCCGCCACAGCCCAACCTGGTGCCCATCGTGGCGGGG 1805

B19_BF2_(PacBio_P2a) TGATGGCCCCGCTCTCCCCCAGAGCCGCCACAGCCCAACCTGGTGCCCATCGTGGCGGGG 1909

B19_BF2_(AM282696) TGATGGCCCCGCTCTCCCCCAGAGCCGCCACAGCCCAACCTGGTGCCCATCGTGGCGGGG 1783

B21_BF2_(AB426152) TGATGGCCCCGCTCTCCCCCAGAGCCGCCACAGCCCAACCTGGTGCCCATCGTGGCGGGG 1802

B21_BF2_(PacBio_N) TGATGGCCCCGCTCTCCCCCAGAGCCGCCACAGCCCAACCTGGTGCCCATCGTGGCGGGG 1906

B21_BF2_(AM282697) TGATGGCCCCGCTCTCCCCCAGAGCCGCCACAGCCCAACCTGGTGCCCATCGTGGCGGGG 1779

B21_BF2_(PacBio_0) TGATGGCCCCGCTCTCCCCCAGAGCCGCCACAGCCCAACCTGGTGCCCATCGTGGCGGGG 1802

B21_BF2_(AM282700) TGATGGCCCCGCTCTCCCCCAGAGCCGCCACAGCCCAACCTGGTGCCCATCGTGGCGGGG 1779

B2_BF2_(AB426141) GTGGCCGTCGCCATTGTGGCCAT**T**GCCATC**A**TGGTTGGTGTTGGATTCATCATCTACAGA 1867

B2_BF2_(PacBio_6sub1) GTGGCCGTCGCCATTGTGGCCAT**T**GCCATC**A**TGGTTGGTGTTGGATTCATCATCTACAGA 1971

B2_BF2_(AM282692) GTGGCCGTCGCCATTGTGGCCAT**T**GCCATC**A**TGGTTGGTGTTGGATTCATCATCTACAGA 1845

B2_BF2_(PacBio_7sub2) GTGGCCGTCGCCATTGTGGCCAT**T**GCCATC**A**TGGTTGGTGTTGGATTCATCATCTACAGA 1971

B2_BF2_(AM282698) GTGGCCGTCGCCATTGTGGCCAT**T**GCCATC**A**TGGTTGGTGTTGGATTCATCATCTACAGA 1845

B4_BF2_(AM282699) GTGGCCGTCGCCATTGTGGCCATCGCCATCGTGGTTGGTGTTGGATTCATCATCTACAGA 1845

B4_BF2_(PacBio_C) GTGGCCGTCGCCATTGTGGCCATCGCCATCGTGGTTGGTGTTGGATTCATCATCTACAGA 1968

B4_BF2_(AM282693) GTGGCCGTCGCCATTGTGGCCATCGCCATCGTGGTTGGTGTTGGATTCATCATCTACAGA 1845

B12_BF2_(AB426147) GTGGCCGTCGCCATTGTGGCCAT**T**GCCATC**A**TGGTTGGTGTTGGATTCATCATCTACAGA 1867

B12_BF2_(PacBio_C) GTGGCCGTCGCCATTGTGGCCAT**T**GCCATC**A**TGGTTGGTGTTGGATTCATCATCTACAGA 1971

B12_BF2_(AL023516) GTGGCCGTCGCCATTGTGGCCAT**T**GCCATC**A**TGGTTGGTGTTGGATTCATCATCTACAGA 1971

B14_BF2_(PacBio_WL) GTGGCCGTCGCCATTGTGGCCATCGCCATCGTGGTTGGTGTTGGATTCA**C**CATCTACAGA 1979

B14_BF2_(AM282694) GTGGCCGTCGCCATTGTGGCCATCGCCATCGTGGTTGGTGTTGGATTCA**C**CATCTACAGA 1851

B15_BF2_(AB426149) GTGGCCGTCGCCATTGTGGCCATCGCCATCGTGGTTGGTGTTGGATTCATCATCTACAGA 1854

B15_BF2_(PacBio_15I) GTGGCCGTCGCCATTGTGGCCATCGCCATCGTGGTTGGTGTTGGATTCATCATCTACAGA 1969

B15_BF2_(AM282695) GTGGCCGTCGCCATTGTGGCCATCGCCATCGTGGTTGGTGTTGGATTCATCATCTACAGA 1843

B19_BF2_(AB426151) GTGGCCGTCGCCATTGTGGCCATCGCCATCGTGGTTGGTGTTGGATTCATCATCTACAGA 1865

B19_BF2_(PacBio_P2a) GTGGCCGTCGCCATTGTGGCCATCGCCATCGTGGTTGGTGTTGGATTCATCATCTACAGA 1969

B19_BF2_(AM282696) GTGGCCGTCGCCATTGTGGCCATCGCCATCGTGGTTGGTGTTGGATTCATCATCTACAGA 1843

B21_BF2_(AB426152) GTGGC**T**GTCGCCATTGTGGCCATCGCCATCGTGGTTGGTGTTGGATTCATCATCTACAGA 1862

B21_BF2_(PacBio_N) GTGGC**T**GTCGCCATTGTGGCCATCGCCATCGTGGTTGGTGTTGGATTCATCATCTACAGA 1966

B21_BF2_(AM282697) GTGGC**T**GTCGCCATTGTGGCCATCGCCATCGTGGTTGGTGTTGGATTCATCATCTACAGA 1839

B21_BF2_(PacBio_0) GTGGC**T**GTCGCCATTGTGGCCATCGCCATCGTGGTTGGTGTTGGATTCATCATCTACAGA 1862

B21_BF2_(AM282700) GTGGC**T**GTCGCCATTGTGGCCATCGCCATCGTGGTTGGTGTTGGATTCATCATCTACAGA 1839

Fig. S2

B2_BF2_(AB426141) CGCCATGCAGGTAAAAGCAGAGGGGTGCAGGCGGGCAGTGGGGG**---------**GATCTGG 1918

B2_BF2_(PacBio_6sub1) CGCCATGCAGGTAAAAGCAGAGGGGTGCAGGCGGGCAGTGGGGG**---------**GATCTGG 2022

B2_BF2_(AM282692) CGCCATGCAGGTAAAAGCAGAGGGGTGCAGGCGGGCAGTGGGGG**---------**GATCTGG 1896

B2_BF2_(PacBio_7sub2) CGCCATGCAGGTAAAAGCAGAGGGGTGCAGGCGGGCAGTGGGGG**---------**GATCTGG 2022

B2_BF2_(AM282698) CGCCATGCAGGTAAAAGCAGAGGGGTGCAGGCGGGCAGTGGGGG**---------**GATCTGG 1896

B4_BF2_(AM282699) CGCCATGCAGGTAAAAGCAGAGGGGTGCAGGCGGGCAGTGGGGGC**A**GT**G**GGGGGATCTGG 1905

B4_BF2_(PacBio_C) CGCCATGCAGGTAAAAGCAGAGGGGTGCAGGCGGGCAGTGGGGGC**A**GT**G**GGGGGATCTGG 2028

B4_BF2_(AM282693) CGCCATGCAGGTAAAAGCAGAGGGGTGCAGGCGGGCAGTGGGGGC**A**GT**G**GGGGGATCTGG 1905

B12_BF2_(AB426147) CGCCATGCAGGTAAAAGCAGAGGGGTGCAGGCGGGCAGTGGGGG**---------**GATCTGG 1918

B12_BF2_(PacBio_C) CGCCATGCAGGTAAAAGCAGAGGGGTGCAGGCGGGCAGTGGGGG**---------**GATCTGG 2022

B12_BF2_(AL023516) CGCCATGCAGGTAAAAGCAGAGGGGTGCAGGCGGGCAGTGGGGG**---------**GATCTGG 2022

B14_BF2_(PacBio_WL) CGCCA**C**GCAGGTAAAAGCAGAGGGGTGCAGGCGGGCAGTGGGGGCTGTAGGGGGATCTGG 2039

B14_BF2_(AM282694) CGCCA**C**GCAGGTAAAAGCAGAGGGGTGCAGGCGGGCAGTGGGGGCTGTAGGGGGATCTGG 1911

B15_BF2_(AB426149) CGCCA**C**GCAGGTAAAAGCAGAGGGGTGCAGGCGGGCAGTGGGGGCTGTAGGGGGATCT**A**G 1914

B15_BF2_(PacBio_15I) CGCCA**C**GCAGGTAAAAGCAGAGGGGTGCAGGCGGGCAGTGGGGGCTGTAGGGGGATCT**A**G 2029

B15_BF2_(AM282695) CGCCA**C**GCAGGTAAAAGCAGAGGGGTGCAGGCGGGCAGTGGGGGCTGTAGGGGGATCT**A**G 1903

B19_BF2_(AB426151) CGCCA**C**GCAGGTAAAAGCAGAGGGGTGCAGGCGGGCAGTGGGGGCTGTAGGGGGATCT**A**G 1925

B19_BF2_(PacBio_P2a) CGCCA**C**GCAGGTAAAAGCAGAGGGGTGCAGGCGGGCAGTGGGGGCTGTAGGGGGATCT**A**G 2029

B19_BF2_(AM282696) CGCCA**C**GCAGGTAAAAGCAGAGGGGTGCAGGCGGGCAGTGGGGGCTGTAGGGGGATCT**A**G 1903

B21_BF2_(AB426152) CGCCATGCAGGTAAAAGCAGAGGGGTGCAGGCGGGC**C**GTGGGGGC**A**GT**G**GGGGCATCTGG 1922

B21_BF2_(PacBio_N) CGCCATGCAGGTAAAAGCAGAGGGGTGCAGGCGGGC**C**GTGGGGGC**A**GT**G**GGGGCATCTGG 2026

B21_BF2_(AM282697) CGCCATGCAGGTAAAAGCAGAGGGGTGCAGGCGGGC**C**GTGGGGGC**A**GT**G**GGGGCATCTGG 1899

B21_BF2_(PacBio_0) CGCCATGCAGGTAAAAGCAGAGGGGTGCAGGCGGGC**C**GTGGGGGC**A**GT**G**GGGGCATCTGG 1922

B21_BF2_(AM282700) CGCCATGCAGGTAAAAGCAGAGGGGTGCAGGCGGGC**C**GTGGGGGC**A**GT**G**GGGGCATCTGG 1899

B2_BF2_(AB426141) GTCCCCCTTGGGAGCCC**T**CA**G**CCTGGCTGTGATGTGAACCTGTGCTGA**A**GCATCTCTCTG 1978

B2_BF2_(PacBio_6sub1) GTCCCCCTTGGGAGCCC**T**CA**G**CCTGGCTGTGATGTGAACCTGTGCTGA**A**GCATCTCTCTG 2082

B2_BF2_(AM282692) GTCCCCCTTGGGAGCCC**T**CA**G**CCTGGCTGTGATGTG**G**ACCTGTGCTGA**A**GCATCTCTCTG 1956

B2_BF2_(PacBio_7sub2) GTCCCCCTTGGGAGCCC**T**CA**G**CCTGGCTGTGATGTGAACCTGTGCTGA**A**GCATCTCTCTG 2082

B2_BF2_(AM282698) GTCCCCCTTGGGAGCCC**T**CA**G**CCTGGCTGTGATGTG**G**ACCTGTGCTGA**A**GCATCTCTCTG 1956

B4_BF2_(AM282699) GTCCCCCTTGGGAGCCCCCAACCTGGCTGTG**-**TGTGAACCTGTG**T**TGATGCATCTCTCTG 1964

B4_BF2_(PacBio_C) GTCCCCCTTGGGAGCCCCCAACCTGGCTGTG-TGTGAACCTGTG**T**TGATGCATCTCTCTG 2087

B4_BF2_(AM282693) GTCCCCCTTGGGAGCCCCCAACCTGGCTGTG**-**TGTGAACCTGTG**T**TGATGCATCTCTCTG 1964

B12_BF2_(AB426147) GTCCCCCTTGGGAGCCC**T**CA**G**CCTGGCTGTGATGTGAACCTGTGCTGA**A**GCATCTCTCTG 1978

B12_BF2_(PacBio_C) GTCCCCCTTGGGAGCCC**T**CA**G**CCTGGCTGTGATGTGAACCTGTGCTGA**A**GCATCTCTCTG 2082

B12_BF2_(AL023516) GTCCCCCTTGGGAGCCC**T**CA**G**CCTGGCTGTGATGTGAACCTGTGCTGA**A**GCATCTCTCTG 2082

B14_BF2_(PacBio_WL) G**C**CCCCCTTGGGAGCCCCCAACCTGGCTGTGATGTGAACCTGTGCTGATGCATCTCTCTG 2099

B14_BF2_(AM282694) G**C**CCCCCTTGGGAGCCCCCAACCTGGCTGTGATGTGAACCTGTGCTGATGCATCTCTCTG 1971

B15_BF2_(AB426149) G**C**CCCCCTTGGGAGCCCCCAACCTGGCTGTGATGTGAACCTGTGCTGATGCATCTCTCTG 1974

B15_BF2_(PacBio_15I) G**C**CCCCCTTGGGAGCCCCCAACCTGGCTGTGATGTGAACCTGTGCTGATGCATCTCTCTG 2089

B15_BF2_(AM282695) G**C**CCCCCTTGGGAGCCCCCAACCTGGCTGTGATGTGAACCTGTGCTGATGCATCTCTCTG 1963

B19_BF2_(AB426151) G**C**CCCCCTTGGGAGCCCCCAACCTGGCTGTGATGTGAACCTGTGCTGATGCATCTCTCTG 1985

B19_BF2_(PacBio_P2a) G**C**CCCCCTTGGGAGCCCCCAACCTGGCTGTGATGTGAACCTGTGCTGATGCATCTCTCTG 2089

B19_BF2_(AM282696) G**C**CCCCCTTGGGAGCCCCCAACCTGGCTGTGATGTGAACCTGTGCTGATGCATCTCTCTG 1963

B21_BF2_(AB426152) GTCCCCCT**A**GGGAGCCC**T**CAACCTGGCTGTGATGTGAACCTGTG**T**TGAT**T**CATCTCTCTG 1982

B21_BF2_(PacBio_N) GTCCCCCT**A**GGGAGCCC**T**CAACCTGGCTGTGATGTGAACCTGTG**T**TGAT**T**CATCTCTCTG 2086

B21_BF2_(AM282697) GTCCCCCT**A**GGGAGCCC**T**CAACCTGGCTGTGATGTGAACCTGTG**T**TGAT**T**CATCTCTCTG 1959

B21_BF2_(PacBio_0) GTCCCCCT**A**GGGAGCCC**T**CAACCTGGCTGTGATGTGAACCTGTG**T**TGAT**T**CATCTCTCTG 1982

B21_BF2_(AM282700) GTCCCCCT**A**GGGAGCCC**T**CAACCTGGCTGTGATGTGAACCTGTG**T**TGAT**T**CATCTCTCTG 1959

**BF2 exon 6**

B2_BF2_(AB426141) TCTGCAGGGAAGAAGGGGAAGGGCTACAACATCGCGCCCGGTGAGTGATGAGGGCAGCGC 2038

B2_BF2_(PacBio_6sub1) TCTGCAGGGAAGAAGGGGAAGGGCTACAACATCGCGCCCGGTGAGTGATGAGGGCAGCGC 2142

B2_BF2_(AM282692) TCTGCAGGGAAGAAGGGGAAGGGCTACAACATCGCGCCCGGTGAGTGATGAGGGCAGCGC 2016

B2_BF2_(PacBio_7sub2) TCTGCAGGGAAGAAGGGGAAGGGCTACAACATCGCGCCCGGTGAGTGATGAGGGCAGCGC 2142

B2_BF2_(AM282698) TCTGCAGGGAAGAAGGGGAAGGGCTACAACATCGCGCCCGGTGAGTGATGAGGGCAGCGC 2016

B4_BF2_(AM282699) TCTGCAGGGAAGAAGGGGAAGGGCTACAACAT**T**GCGCCCGGTGAGTGATGAGGGCAGCGC 2024

B4_BF2_(PacBio_C) TCTGCAGGGAAGAAGGGGAAGGGCTACAACAT**T**GCGCCCGGTGAGTGATGAGGGCAGCGC 2147

B4_BF2_(AM282693) TCTGCAGGGAAGAAGGGGAAGGGCTACAACAT**T**GCGCCCGGTGAGTGATGAGGGCAGCGC 2024

B12_BF2_(AB426147) TCTGCAGGGAAGAAGGGGAAGGGCTACAACATCGCGCCCGGTGAGTGATGAGGGCAGCGC 2038

B12_BF2_(PacBio_C) TCTGCAGGGAAGAAGGGGAAGGGCTACAACATCGCGCCCGGTGAGTGATGAGGGCAGCGC 2142

B12_BF2_(AL023516) TCTGCAGGGAAGAAGGGGAAGGGCTACAACATCGCGCCCGGTGAGTGATGAGGGCAGCGC 2142

B14_BF2_(PacBio_WL) TCTGCAGGGAAGAAGGGGAAGGGCTACAACATCGCGCCCGGTGAGTGATGAGGGCAGCGC 2159

B14_BF2_(AM282694) TCTGCAGGGAAGAAGGGGAAGGGCTACAACATCGCGCCCGGTGAGTGATGAGGGCAGCGC 2031

B15_BF2_(AB426149) TCTGCAGGGAAGAAGGGGAAGGGCTACAACATCGCGCCCGGTGAGTGATGAGGGCAG**T**GC 2034

B15_BF2_(PacBio_15I) TCTGCAGGGAAGAAGGGGAAGGGCTACAACATCGCGCCCGGTGAGTGATGAGGGCAG**T**GC 2149

B15_BF2_(AM282695) TCTGCAGGGAAGAAGGGGAAGGGCTACAACATCGCGCCCGGTGAGTGATGAGGGCAG**T**GC 2023

B19_BF2_(AB426151) TCTGCAGGGAAGAAGGGGAAGGGCTACAACATCGCGCCCGGTGAGTGATGAGGGCAG**T**GC 2045

B19_BF2_(PacBio_P2a) TCTGCAGGGAAGAAGGGGAAGGGCTACAACATCGCGCCCGGTGAGTGATGAGGGCAG**T**GC 2149

B19_BF2_(AM282696) TCTGCAGGGAAGAAGGGGAAGGGCTACAACATCGCGCCCGGTGAGTGATGAGGGCAG**T**GC 2023

B21_BF2_(AB426152) TCTGCAGGGAAGAAGGGGAAGGGCTACAACATCGCGCCCGGTGAGTGATGAGGGCAGCGC 2042

B21_BF2_(PacBio_N) TCTGCAGGGAAGAAGGGGAAGGGCTACAACATCGCGCCCGGTGAGTGATGAGGGCAGCGC 2146

B21_BF2_(AM282697) TCTGCAGGGAAGAAGGGGAAGGGCTACAACATCGCGCCCGGTGAGTGATGAGGGCAGCGC 2019

B21_BF2_(PacBio_0) TCTGCAGGGAAGAAGGGGAAGGGCTACAACATCGCGCCCGGTGAGTGATGAGGGCAGCGC 2042

B21_BF2_(AM282700) TCTGCAGGGAAGAAGGGGAAGGGCTACAACATCGCGCCCGGTGAGTGATGAGGGCAGCGC 2019

Fig. S2

B2_BF2_(AB426141) TGTCCCCCACCTCTGCCCAGTGCCAGGGCGGTCCTGGGGTC**TG**CACTTTCTCCCAGGGTA 2098

B2_BF2_(PacBio_6sub1) TGTCCCCCACCTCTGCCCAGTGCCAGGGCGGTCCTGGGGTC**TG**CACTTTCTCCCAGGGTA 2202

B2_BF2_(AM282692) TGTCCCCCACCTCTGCCCAGTGCCAGGGCGGTCCTGGGGTC**TG**CACTTTCTCCCAGGGTA 2076

B2_BF2_(PacBio_7sub2) TGTCCCCCACCTCTGCCCAGTGCCAGGGCGGTCCTGGGGTC**TG**CACTTTCTCCCAGGGTA 2202

B2_BF2_(AM282698) TGTCCCCCACCTCTGCCCAGTGCCAGGGCGGTCCTGGGGTC**TG**CACTTTCTCCCAGGGTA 2076

B4_BF2_(AM282699) TGTCCCCCACCTCTGCCCAGTGCCAGGGCGGTCCTGGGGTCCCCACTTTCTCCCAGGGTA 2084

B4_BF2_(PacBio_C) TGTCCCCCACCTCTGCCCAGTGCCAGGGCGGTCCTGGGGTCCCCACTTTCTCCCAGGGTA 2207

B4_BF2_(AM282693) TGTCCCCCACCTCTGCCCAGTGCCAGGGCGGTCCTGGGGTCCCCACTTTCTCCCAGGGTA 2084

B12_BF2_(AB426147) TGTCCCCCACCTCTGCCCAGTGCCAGGGCGGTCCTGGGGTC**TG**CACTTTCTCCCAGGGTA 2098

B12_BF2_(PacBio_C) TGTCCCCCACCTCTGCCCAGTGCCAGGGCGGTCCTGGGGTC**TG**CACTTTCTCCCAGGGTA 2202

B12_BF2_(AL023516) TGTCCCCCACCTCTGCCCAGTGCCAGGGCGGTCCTGGGGTC**TG**CACTTTCTCCCAGGGTA 2202

B14_BF2_(PacBio_WL) TGTCCCCCACCTCTGCCCAGTG**T**CAGGG**T**GGTCCTGGGGTCCC**TG**CTTTCTCCCA**A**GGTA 2219

B14_BF2_(AM282694) TGTCCCCCACCTCTGCCCAGTG**T**CAGGG**T**GGTCCTGGGGTCCC**TG**CTTTCTCCCA**A**GGTA 2091

B15_BF2_(AB426149) TGTCCCCCACCTCTGCCCAGTG**T**CAGGG**T**GGTCCTGGGGTCCC**TG**CTTTCTCCCA**A**GGTA 2094

B15_BF2_(PacBio_15I) TGTCCCCCACCTCTGCCCAGTG**T**CAGGG**T**GGTCCTGGGGTCCC**TG**CTTTCTCCCA**A**GGTA 2209

B15_BF2_(AM282695) TGTCCCCCACCTCTGCCCAGTG**T**CAGGG**T**GGTCCTGGGGTCCC**TG**CTTTCTCCCA**A**GGTA 2083

B19_BF2_(AB426151) TGTCCCCCACCTCTGCCCAGTG**T**CAGGG**T**GGTCCTGGGGTCCC**TG**CTTTCTCCCA**A**GGTA 2105

B19_BF2_(PacBio_P2a) TGTCCCCCACCTCTGCCCAGTG**T**CAGGG**T**GGTCCTGGGGTCCC**TG**CTTTCTCCCA**A**GGTA 2209

B19_BF2_(AM282696) TGTCCCCCACCTCTGCCCAGTGTCAGGG**T**GGTCCTGGGGTCCC**TG**CTTTCTCCCA**A**GGTA 2083

B21_BF2_(AB426152) TGTCCCCCACCTCTGCCCAGTGCCCGGGCGGTCCTGGGGTC**T**CCACTTTCTC**T**CAGGGTA 2102

B21_BF2_(PacBio_N) TGTCCCCCACCTCTGCCCAGTGCCCGGGCGGTCCTGGGGTC**T**CCACTTTCTC**T**CAGGGTA 2206

B21_BF2_(AM282697) TGTCCCCCACCTCTGCCCAGTGCCCGGGCGGTCCTGGGGTC**T**CCACTTTCTC**T**CAGGGTA 2079

B21_BF2_(PacBio_0) TGTCCCCCACCTCTGCCCAGTGCCCGGGCGGTCCTGGGGTC**T**CCACTTTCTC**T**CAGGGTA 2102

B21_BF2_(AM282700) TGTCCCCCACCTCTGCCCAGTGCCCGGGCGGTCCTGGGGTC**T**CCACTTTCTC**T**CAGGGTA 2079

B2_BF2_(AB426141) CCCATTCCTGGTGCTTGGGGCTGCTCCA**C**GCCCCATAGGGAGCACAGGGCTGG**A**TCTCAC 2158

B2_BF2_(PacBio_6sub1) CCCATTCCTGGTGCTTGGGGCTGCTCCA**C**GCCCCATAGGGAGCACAGGGCTGG**A**TCTCAC 2262

B2_BF2_(AM282692) CCCATTCCTGGTGCTTGGGGCTGCTCCA**C**GCCCCATAGGGAGCACAGGGCTGG**A**TCTCAC 2136

B2_BF2_(PacBio_7sub2) CCCATTCCTGGTGCTTGGGGCTGCTCCA**C**GCCCCATAGGGAGCACAGGGCTGG**A**TCTCAC 2262

B2_BF2_(AM282698) CCCATTCCTGGTGCTTGGGGCTGCTCCA**C**GCCCCATAGGGAGCACAGGGCTGG**A**TCTCAC 2136

B4_BF2_(AM282699) CCCATTCCTGGTGCTTGGGGCTGCTCCATGCCCCA**G**AGGGAGCACAGGGCTGGGTCT**T**AC 2144

B4_BF2_(PacBio_C) CCCATTCCTGGTGCTTGGGGCTGCTCCATGCCCCA**G**AGGGAGCACAGGGCTGGGTCT**T**AC 2267

B4_BF2_(AM282693) CCCATTCCTGGTGCTTGGGGCTGCTCCATGCCCCA**G**AGGGAGCACAGGGCTGGGTCT**T**AC 2144

B12_BF2_(AB426147) CCCATTCCTGGTGCTTGGGGCTGCTCCA**C**GCCCCATAGGGAGCACAGGGCTGG**A**TCTCAC 2158

B12_BF2_(PacBio_C) CCCATTCCTGGTGCTTGGGGCTGCTCCA**C**GCCCCATAGGGAGCACAGGGCTGG**A**TCTCAC 2262

B12_BF2_(AL023516) CCCATTCCTGGTGCTTGGGGCTGCTCCA**C**GCCCCATAGGGAGCACAGGGCTGG**A**TCTCAC 2262

B14_BF2_(PacBio_WL) CCCATTCCTGGTGCTTGGGGCTGCTCCATGCCCCATA**A**GGAGCACAGGGCTGGGTCTCAC 2279

B14_BF2_(AM282694) CCCATTCCTGGTGCTTGGGGCTGCTCCATGCCCCATA**A**GGAGCACAGGGCTGGGTCTCAC 2151

B15_BF2_(AB426149) CCCATTCCTGGTGCTTGGGGCTGCTCCATGCCCCATA**A**GGAGCACAGGGCTGGGTCTCAC 2154

B15_BF2_(PacBio_15I) CCCATTCCTGGTGCTTGGGGCTGCTCCATGCCCCATA**A**GGAGCACAGGGCTGGGTCTCAC 2269

B15_BF2_(AM282695) CCCATTCCTGGTGCTTGGGGCTGCTCCATGCCCCATA**A**GGAGCACAGGGCTGGGTCTCAC 2143

B19_BF2_(AB426151) CCCATTCCTGGTGCTTGGGGCTGCTCCATGCCCCATAGGGAGCACAGGGCTGG**A**TCTCAC 2165

B19_BF2_(PacBio_P2a) CCCATTCCTGGTGCTTGGGGCTGCTCCATGCCCCATAGGGAGCACAGGGCTGG**A**TCTCAC 2269

B19_BF2_(AM282696) CCCATTCCTGGTGCTTGGGGCTGCTCCATGCCCCATAGGGAGCACAGGGCTGG**A**TCTCAC 2143

B21_BF2_(AB426152) CCCATTCCTGGTGCTTGGGGCTGCTCCATGCCCCATAGGGAGCACAGG**A**CTGGGTCTCAC 2162

B21_BF2_(PacBio_N) CCCATTCCTGGTGCTTGGGGCTGCTCCATGCCCCATAGGGAGCACAGG**A**CTGGGTCTCAC 2266

B21_BF2_(AM282697) CCCATTCCTGGTGCTTGGGGCTGCTCCATGCCCCATAGGGAGCACAGG**A**CTGGGTCTCAC 2139

B21_BF2_(PacBio_0) CCCATTCCTGGTGCTTGGGGCTGCTCCATGCCCCATAGGGAGCACAGG**A**CTGGGTCTCAC 2162

B21_BF2_(AM282700) CCCATTCCTGGTGCTTGGGGCTGCTCCATGCCCCATAGGGAGCACAGG**A**CTGGGTCTCAC 2139

**BF2 exon 7**

B2_BF2_(AB426141) AGCTGTTCCTCCCTTATAGACAGGGAAGGTGGATCCAGCAGCTCGAGCACAGGTGCGGTG 2218

B2_BF2_(PacBio_6sub1) AGCTGTTCCTCCCTTATAGACAGGGAAGGTGGATCCAGCAGCTCGAGCACAGGTGCGGTG 2322

B2_BF2_(AM282692) AGCTGTTCCTCCCTTATAGACAGGGAAGGTGGATCCAGCAGCTCGAGCACAGGTGCGGTG 2196

B2_BF2_(PacBio_7sub2) AGCTGTTCCTCCCTTATAGACAGGGAAGGTGGATCCAGCAGCTCGAGCACAGGTGCGGTG 2322

B2_BF2_(AM282698) AGCTGTTCCTCCCTTATAGACAGGGAAGGTGGATCCAGCAGCTCGAGCACAGGTGCGGTG 2196

B4_BF2_(AM282699) AGCTGTTC**T**TCCCTTATAGACAGGGAAGGTGGATCCAGCAGCTCGAGCACAGGTGCGGTG 2204

B4_BF2_(PacBio_C) AGCTGTTC**T**TCCCTTATAGACAGGGAAGGTGGATCCAGCAGCTCGAGCACAGGTGCGGTG 2327

B4_BF2_(AM282693) AGCTGTTC**T**TCCCTTATAGACAGGGAAGGTGGATCCAGCAGCTCGAGCACAGGTGCGGTG 2204

B12_BF2_(AB426147) AGCTGTTCCTCCCTTATAGACAGGGAAGGTGGATCCAGCAGCTCGAGCACAGGTGCGGTG 2218

B12_BF2_(PacBio_C) AGCTGTTCCTCCCTTATAGACAGGGAAGGTGGATCCAGCAGCTCGAGCACAGGTGCGGTG 2322

B12_BF2_(AL023516) AGCTGTTCCTCCCTTATAGACAGGGAAGGTGGATCCAGCAGCTCGAGCACAGGTGCGGTG 2322

B14_BF2_(PacBio_WL) AGCTGTTCCTCCCTTATAGACAGGGAAGGTGGATCCAGCAGCTCGAGCACAGGTGCGGTG 2339

B14_BF2_(AM282694) AGCTGTTCCTCCCTTATAGACAGGGAAGGTGGATCCAGCAGCTCGAGCACAGGTGCGGTG 2211

B15_BF2_(AB426149) AGCTGTTCCTCCCTTATAGACAGGGAAGGTGGATCCAGCAGCTCGAGCACAGGTGCGGTG 2214

B15_BF2_(PacBio_15I) AGCTGTTCCTCCCTTATAGACAGGGAAGGTGGATCCAGCAGCTCGAGCACAGGTGCGGTG 2329

B15_BF2_(AM282695) AGCTGTTCCTCCCTTATAGACAGGGAAGGTGGATCCAGCAGCTCGAGCACAGGTGCGGTG 2203

B19_BF2_(AB426151) AGCTGTTCCTCCCTTATAGACAGGGAAGGTGGATCCAGCAGCTCGAGCACAGGTGCGGTG 2225

B19_BF2_(PacBio_P2a) AGCTGTTCCTCCCTTATAGACAGGGAAGGTGGATCCAGCAGCTCGAGCACAGGTGCGGTG 2329

B19_BF2_(AM282696) AGCTGTTCCTCCCTTATAGACAGGGAAGGTGGATCCAGCAGCTCGAGCACAGGTGCGGTG 2203

B21_BF2_(AB426152) AGCTGTTCCTCCCTTATAGACAGGGAAGGTGGATCCAGCAGCTCGAGCACAGGTGCGGTG 2222

B21_BF2_(PacBio_N) AGCTGTTCCTCCCTTATAGACAGGGAAGGTGGATCCAGCAGCTCGAGCACAGGTGCGGTG 2326

B21_BF2_(AM282697) AGCTGTTCCTCCCTTATAGACAGGGAAGGTGGATCCAGCAGCTCGAGCACAGGTGCGGTG 2199

B21_BF2_(PacBio_0) AGCTGTTCCTCCCTTATAGACAGGGAAGGTGGATCCAGCAGCTCGAGCACAGGTGCGGTG 2222

B21_BF2_(AM282700) AGCTGTTCCTCCCTTATAGACAGGGAAGGTGGATCCAGCAGCTCGAGCACAGGTGCGGTG 2199

Fig. S2

B2_BF2_(AB426141) TGGGGCTGTGGGTTGGGAGGGGTCCGTGTGCTCTCTGTGGTACTGCCCAGGGCTGGGCTA 2278

B2_BF2_(PacBio_6sub1) TGGGGCTGTGGGTTGGGAGGGGTCCGTGTGCTCTCTGTGGTACTGCCCAGGGCTGGGCTA 2382

B2_BF2_(AM282692) TGGGGCTGTGGGTTGGGAGGGGTCCGTGTGCTCTCTGTGGTACTGCCCAGGGCTGGGCTA 2256

B2_BF2_(PacBio_7sub2) TGGGGCTGTGGGTTGGGAGGGGTCCGTGTGCTCTCTGTGGTACTGCCCAGGGCTGGGCTA 2382

B2_BF2_(AM282698) TGGGGCTGTGGGTTGGGAGGGGTCCGTGTGCTCTCTGTGGTACTGCCCAGGGCTGGGCTA 2256

B4_BF2_(AM282699) TGGGGCTGTGGGTTGGGAGGGGTCCGTGTGCTCTCTGTGGTACTGCCCAGGGCTGGGCTA 2264

B4_BF2_(PacBio_C) TGGGGCTGTGGGTTGGGAGGGGTCCGTGTGCTCTCTGTGGTACTGCCCAGGGCTGGGCTA 2387

B4_BF2_(AM282693) TGGGGCTGTGGGTTGGGAGGGGTCCGTGTGCTCTCTGTGGTACTGCCCAGGGCTGGGCTA 2264

B12_BF2_(AB426147) TGGGGCTGTGGGTTGGGAGGGGTCCGTGTGCTCTCTGTGGTACTGCCCAGGGCTGGGCTA 2278

B12_BF2_(PacBio_C) TGGGGCTGTGGGTTGGGAGGGGTCCGTGTGCTCTCTGTGGTACTGCCCAGGGCTGGGCTA 2382

B12_BF2_(AL023516) TGGGGCTGTGGGTTGGGAGGGGTCCGTGTGCTCTCTGTGGTACTGCCCAGGGCTGGGCTA 2382

B14_BF2_(PacBio_WL) TGGGGCTGTGGGTTGGGAGGGGTC**T**GTGTGCTCTCTGTGGTACTGCCCAGGGCTGGGCTA 2399

B14_BF2_(AM282694) TGGGGCTGTGGGTTGGGAGGGGTC**T**GTGTGCTCTCTGTGGTACTGCCCAGGGCTGGGCTA 2271

B15_BF2_(AB426149) TGGGGCTGTGGGTTGGGAGGGGTCC**C**TGTGCTCTCTGTGGTACTGCCCAG**T**GCTGGGCTA 2274

B15_BF2_(PacBio_15I) TGGGGCTGTGGGTTGGGAGGGGTCC**C**TGTGCTCTCTGTGGTACTGCCCAG**T**GCTGGGCTA 2389

B15_BF2_(AM282695) TGGGGCTGTGGGTTGGGAGGGGTCC**C**TGTGCTCTCTGTGGTACTGCCCAG**T**GCTGGGCTA 2263

B19_BF2_(AB426151) TGGGGCTGTGGGTTGGGAGGGGTCCGTGTGCTCTCTGTGGTACTGCCCAGGGCTGGGCTA 2285

B19_BF2_(PacBio_P2a) TGGGGCTGTGGGTTGGGAGGGGTCCGTGTGCTCTCTGTGGTACTGCCCAGGGCTGGGCTA 2389

B19_BF2_(AM282696) TGGGGCTGTGGGTTGGGAGGGGTCCGTGTGCTCTCTGTGGTACTGCCCAGGGCTGGGCTA 2263

B21_BF2_(AB426152) TGGGGCTGTGGGTTGGGAGGGGTCC**A**TGTGCTCTC**G**GTGGTACTGCCCAGGGCTGGGCTA 2282

B21_BF2_(PacBio_N) TGGGGCTGTGGGTTGGGAGGGGTCC**A**TGTGCTCTC**G**GTGGTACTGCCCAGGGCTGGGCTA 2386

B21_BF2_(AM282697) TGGGGCTGTGGGTTGGGAGGGGTCC**A**TGTGCTCTC**G**GTGGTACTGCCCAGGGCTGGGCTA 2259

B21_BF2_(PacBio_0) TGGGGCTGTGGGTTGGGAGGGGTCC**A**TGTGCTCTC**G**GTGGTACTGCCCAGGGCTGGGCTA 2282

B21_BF2_(AM282700) TGGGGCTGTGGGTTGGGAGGGGTCC**A**TGTGCTCTC**G**GTGGTACTGCCCAGGGCTGGGCTA 2259

B2_BF2_(AB426141) TGCTGGGGCTCTGCGGGGAGACCCCCGGAGCAGAGGGTTGGGATGTGAACCTGGCCCCGT 2338

B2_BF2_(PacBio_6sub1) TGCTGGGGCTCTGCGGGGAGACCCCCGGAGCAGAGGGTTGGGATGTGAACCTGGCCCCGT 2442

B2_BF2_(AM282692) TGCTGGGGCTCTGCGGGGAGACCCCCGGAGCAGAGGGTTGGGATGTGAACCTGGCCCCGT 2316

B2_BF2_(PacBio_7sub2) TGCTGGGGCTCTGCGGGGAGACCCCCGGAGCAGAGGGTTGGGATGTGAACCTGGCCCCGT 2442

B2_BF2_(AM282698) TGCTGGGGCTCTGCGGGGAGACCCCCGGAGCAGAGGGTTGGGATGTGAACCTGGCCCCGT 2316

B4_BF2_(AM282699) TGCTG**A**GGCTCTGCGGGGAGACCCCCGGAGCAGAGGGTTGGGATGTGAACCTGGCCCCGT 2324

B4_BF2_(PacBio_C) TGCTG**A**GGCTCTGCGGGGAGACCCCCGGAGCAGAGGGTTGGGATGTGAACCTGGCCCCGT 2447

B4_BF2_(AM282693) TGCTG**A**GGCTCTGCGGGGAGACCCCCGGAGCAGAGGGTTGGGATGTGAACCTGGCCCCGT 2324

B12_BF2_(AB426147) TGCTGGGGCTCTGCGGGGAGACCCCCGGAGCAGAGGGTTGGGATGTGAACCTGGCCCCGT 2338

B12_BF2_(PacBio_C) TGCTGGGGCTCTGCGGGGAGACCCCCGGAGCAGAGGGTTGGGATGTGAACCTGGCCCCGT 2442

B12_BF2_(AL023516) TGCTGGGGCTCTGCGGGGAGACCCCCGGAGCAGAGGGTTGGGATGTGAACCTGGCCCCGT 2442

B14_BF2_(PacBio_WL) TGCTGGGGCTCTGC**AC**GGAGACCCCCGGAGCAGAGGGTTGGGATGTGAACCTGGCCCCGT 2459

B14_BF2_(AM282694) TGCTGGGGCTCTGC**AC**GGAGACCCCCGGAGCAGAGGGTTGGGATGTGAACCTGGCCCCGT 2331

B15_BF2_(AB426149) TGCTGGGGCTCTGCGGGGAGACCCCC**A**GAGCAGAGGGTTGGGATGTGAACCTGGCCCCGT 2334

B15_BF2_(PacBio_15I) TGCTGGGGCTCTGCGGGGAGACCCCC**A**GAGCAGAGGGTTGGGATGTGAACCTGGCCCCGT 2449

B15_BF2_(AM282695) TGCTGGGGCTCTGCGGGGAGACCCCC**A**GAGCAGAGGGTTGGGATGTGAACCTGGCCCCGT 2323

B19_BF2_(AB426151) TGCTGGGGCTCTGCGGGGAGACCCCCGGAGCAGAGGGTTGGGATGTGAACCTGG**T**CCCGT 2345

B19_BF2_(PacBio_P2a) TGCTGGGGCTCTGCGGGGAGACCCCCGGAGCAGAGGGTTGGGATGTGAACCTGG**T**CCCGT 2449

B19_BF2_(AM282696) TGCTGGGGCTCTGCGGGGAGACCCCCGGAGCAGAGGGTTGGGATGTGAACCTGG**T**CCCGT 2323

B21_BF2_(AB426152) TGCTGGGGCTCTGCGGGGAGACCCCCGGAGCAGAGGGTTGGGATGTGAACCTGGCCCCGT 2342

B21_BF2_(PacBio_N) TGCTGGGGCTCTGCGGGGAGACCCCCGGAGCAGAGGGTTGGGATGTGAACCTGGCCCCGT 2446

B21_BF2_(AM282697) TGCTGGG**A**CTCTGCGGGGAGACCCCCGGAGCAGAGGGTTGGGATGTGAACCTGGCCCCGT 2319

B21_BF2_(PacBio_0) TGCTGGGGCTCTGCGGGGAGACCCCCGGAGCAGAGGGTTGGGATGTGAACCTGGCCCCGT 2342

B21_BF2_(AM282700) TGCTGGG**A**CTCTGCGGGGAGACCCCCGGAGCAGAGGGTTGGGATGTGAACCTGGCCCCGT 2319

**BF2 exon 8 BF2 stop**

B2_BF2_(AB426141) GGGACATCATCCCTTCTCATCCCCACAGGGAGCAACCCCGCCATCTGAGTGCTGTGCTTC 2398

B2_BF2_(PacBio_6sub1) GGGACATCATCCCTTCTCATCCCCACAGGGAGCAACCCCGCCATCTGAGTGCTGTGCTTC 2502

B2_BF2_(AM282692) GGGACATCATCCCTTCTCATCCCCACAGGGAGCAACCCCGCCATCTGAGTGCTGTGCTTC 2376

B2_BF2_(PacBio_7sub2) GGGACATCATCCCTTCTCATCCCCACAGGGAGCAACCCCGCCATCTGAGTGCTGTGCTTC 2502

B2_BF2_(AM282698) GGGACATCATCCCTTCTCATCCCCACAGGGAGCAACCCCGCCATCTGAGTGCTGTGCTTC 2376

B4_BF2_(AM282699) GGGACA**C**CATC**T**CTTCTCATCCCCACAGGGAGCAACCCC**A**CCATCTGAGTGCTGTGCTTC 2384

B4_BF2_(PacBio_C) GGGACA**C**CATC**T**CTTCTCATCCCCACAGGGAGCAACCCC**A**CCATCTGAGTGCTGTGCTTC 2507

B4_BF2_(AM282693) GGGACA**C**CATC**T**CTTCTCATCCCCACAGGGAGCAACCCC**A**CCATCTGAGTGCTGTGCTTC 2384

B12_BF2_(AB426147) GGGACATCATCCCTTCTCATCCCCACAGGGAGCAACCCCGCCATCTGAGTGCTGTGCTTC 2398

B12_BF2_(PacBio_C) GGGACATCATCCCTTCTCATCCCCACAGGGAGCAACCCCGCCATCTGAGTGCTGTGCTTC 2502

B12_BF2_(AL023516) GGGACATCATCCCTTCTCATCCCCACAGGGAGCAACCCCGCCATCTGAGTGCTGTGCTTC 2502

B14_BF2_(PacBio_WL) GGGACATCATCCCTTCTCATCCCCACAGGGAGCAACCCCGCCATCTGAGTGCTGTGCTTC 2519

B14_BF2_(AM282694) GGGACATCATCCCTTCTCATCCCCACAGGGAGCAACCCCGCCATCTGAGTGCTGTGCTTC 2391

B15_BF2_(AB426149) GGGACATCATCCCTTCTCATCCCCACAGGGAGCAACCCCGCCATCTGAGTGCTGTGCTTC 2394

B15_BF2_(PacBio_15I) GGGACATCATCCCTTCTCATCCCCACAGGGAGCAACCCCGCCATCTGAGTGCTGTGCTTC 2509

B15_BF2_(AM282695) GGGACATCATCCCTTCTCATCCCCACAGGGAGCAACCCCGCCATCTGAGTGCTGTGCTTC 2383

B19_BF2_(AB426151) GGGACATCATCCCTTCTCATCCCCACAGGGAGCAACCCCGCCATCTGAGTGCTGTGCTTC 2405

B19_BF2_(PacBio_P2a) GGGACATCATCCCTTCTCATCCCCACAGGGAGCAACCCCGCCATCTGAGTGCTGTGCTTC 2509

B19_BF2_(AM282696) GGGACATCATCCCTTCTCATCCCCACAGGGAGCAACCCCGCCATCTGAGTGCTGTGCTTC 2383

B21_BF2_(AB426152) GGGACATCATCCCTTCTCATCCCCACAGGGAGCAACCCC**T**CCATCTGAGTGCTGTGCTTC 2402

B21_BF2_(PacBio_N) GGGACATCATCCCTTCTCATCCCCACAGGGAGCAACCCC**T**CCATCTGAGTGCTGTGCTTC 2506

B21_BF2_(AM282697) GGGACATCATCCCTTCTCATCCCCACAGGGAGCAACCCC**T**CCATCTGAGTGCTGTGCTTC 2379

B21_BF2_(PacBio_0) GGGACATCATCCCTTCTCATCCCCACAGGGAGCAACCCC**T**CCATCTGAGTGCTGTGCTTC 2402

B21_BF2_(AM282700) GGGACATCATCCCTTCTCATCCCCACAGGGAGCAACCCC**T**CCATCTGAGTGCTGTGCTTC 2379

Fig. S2

B2_BF2_(AB426141) AGCCTGCAAGGAGCCAACAGTCCACACCAGCATTTGGGGTC**A**GTGATGGGCACAGCCCCA 2458

B2_BF2_(PacBio_6sub1) AGCCTGCAAGGAGCCAACAGTCCACACCAGCATTTGGGGTC**A**GTGATGGGCACAGCCCCA 2562

B2_BF2_(AM282692) AGCCTGCAAGGAGCCAACAGTCCACACCAGCATTTGGGGTC**A**GTGATGGGCACAGCCCCA 2436

B2_BF2_(PacBio_7sub2) AGCCTGCAAGGAGCCAACAGTCCACACCAGCATTTGGGGTC**A**GTGATGGGCACAGCCCCA 2562

B2_BF2_(AM282698) AGCCTGCAAGGAGCCAACAGTCCACACCAGCATTTGGGGTC**A**GTGATGGGCACAGCCCCA 2436

B4_BF2_(AM282699) AGCCTG**T**AAGGAGCCAACAGTCCACACCAGCATTTGGGGTCGGTGATGG**A**CACAGCCCCA 2444

B4_BF2_(PacBio_C) AGCCTG**T**AAGGAGCCAACAGTCCACACCAGCATTTGGGGTCGGTGATGG**A**CACAGCCCCA 2567

B4_BF2_(AM282693) AGCCTG**T**AAGGAGCCAACAGTCCACACCAGCATTTGGGGTCGGTGATGG**A**CACAGCCCCA 2444

B12_BF2_(AB426147) AGCCTGCAAGGAGCCAACAGTCCACACCAGCATTTGGGGTC**A**GTGATGGGCACAGCCCCA 2458

B12_BF2_(PacBio_C) AGCCTGCAAGGAGCCAACAGTCCACACCAGCATTTGGGGTC**A**GTGATGGGCACAGCCCCA 2562

B12_BF2_(AL023516) AGCCTGCAAGGAGCCAACAGTCCACACCAGCATTTGGGGTC**A**GTGATGGGCACAGCCCCA 2562

B14_BF2_(PacBio_WL) AGCCTGCAAGGAGCCAACAGTCCACACCAGCATTTGGGGTCGGTGATGGGCACAGCCCCA 2579

B14_BF2_(AM282694) AGCCTGCAAGGAGCCAACAGTCCACACCAGCATTTGGGGTCGGTGATGGGCACAGCCCCA 2451

B15_BF2_(AB426149) AGCCTGCAAGGAGCCAACAGTCCACACCAGCATTTGGGGTCGGTGATGGGCACAGCCCCA 2454

B15_BF2_(PacBio_15I) AGCCTGCAAGGAGCCAACAGTCCACACCAGCATTTGGGGTCGGTGATGGGCACAGCCCCA 2569

B15_BF2_(AM282695) AGCCTGCAAGGAGCCAACAGTCCACACCAGCATTTGGGGTCGGTGATGGGCACAGCCCCA 2443

B19_BF2_(AB426151) AGCCTGCA**C**G**A**AGCCAACAGTCCACACCAGCATTTGGGGTCGGTGATGG**A**CACAGCCCCA 2465

B19_BF2_(PacBio_P2a) AGCCTGCA**C**G**A**AGCCAACAGTCCACACCAGCATTTGGGGTCGGTGATGG**A**CACAGCCCCA 2569

B19_BF2_(AM282696) AGCCTGCA**C**G**A**AGCCAACAGTCCACACCAGCATTTGGGGTCGGTGATGG**A**CACAGCCCCA 2443

B21_BF2_(AB426152) AGCCTGCAAGGAGCCAACAGTCCACACCAGCATTTGGGGTCGGTGATGG**A**CACAGCCCCA 2462

B21_BF2_(PacBio_N) AGCCTGCAAGGAGCCAACAGTCCACACCAGCATTTGGGGTCGGTGATGG**A**CACAGCCCCA 2566

B21_BF2_(AM282697) AGCCTGCAAGGAGCCAACAGTCCACACCAGCATTTGGGGTCGGTGATGG**A**CACAGCCCCA 2439

B21_BF2_(PacBio_0) AGCCTGCAAGGAGCCAACAGTCCACACCAGCATTTGGGGTCGGTGATGG**A**CACAGCCCCA 2462

B21_BF2_(AM282700) AGCCTGCAAGGAGCCAACAGTCCACACCAGCATTTGGGGTCGGTGATGG**A**CACAGCCCCA 2439

B2_BF2_(AB426141) TCCTCTTGACCTCTCACATCTCATTCTGCTTCCTATGCTGACTGTTATGCTTTGCCTGCA 2518

B2_BF2_(PacBio_6sub1) TCCTCTTGACCTCTCACATCTCATTCTGCTTCCTATGCTGACTGTTATGCTTTGCCTGCA 2622

B2_BF2_(AM282692) TCCTCTTGACCTCTCACATCTCATTCTGCTTCCTATGCTGACTGTTATGCTTTGCCTGCA 2496

B2_BF2_(PacBio_7sub2) TCCTCTTGACCTCTCACATCTCATTCTGCTTCCTATGCTGACTGTTATGCTTTGCCTGCA 2622

B2_BF2_(AM282698) TCCTCTTGACCTCTCACATCTCATTCTGCTTCCTATGCTGACTGTTATGCTTTGCCTGCA 2496

B4_BF2_(AM282699) TCCTCTTGACCTCTCACATCTCATTCTGCTTCCTATGCTGACTGTTATGCTTTGCCTGCA 2504

B4_BF2_(PacBio_C) TCCTCTTGACCTCTCACATCTCATTCTGCTTCCTATGCTGACTGTTATGCTTTGCCTGCA 2627

B4_BF2_(AM282693) TCCTCTTGACCTCTCACATCTCATTCTGCTTCCTATGCTGACTGTTATGCTTTGCCTGCA 2504

B12_BF2_(AB426147) TCCTCTTGACCTCTCACATCTCATTCTGCTTCCTATGCTGACTGTTATGCTTTGCCTGCA 2518

B12_BF2_(PacBio_C) TCCTCTTGACCTCTCACATCTCATTCTGCTTCCTATGCTGACTGTTATGCTTTGCCTGCA 2622

B12_BF2_(AL023516) TCCTCTTGACCTCTCACATCTCATTCTGCTTCCTATGCTGACTGTTATGCTTTGCCTGCA 2622

B14_BF2_(PacBio_WL) TCCTCTTGACCTCTCACATCTCATTCTGCTTCCTATGCTGACTGTTATGCTTTGCCTGCA 2639

B14_BF2_(AM282694) TCCTCTTGACCTCTCACATCTCATTCTGCTTCCTATGCTGACTGTTATGCTTTGCCTGCA 2511

B15_BF2_(AB426149) TCCTCTTGACCTCTCACATCTCATTCTGCTTCCTATGCTGACTGTTATGCTTTGCCTGCA 2514

B15_BF2_(PacBio_15I) TCCTCTTGACCTCTCACATCTCATTCTGCTTCCTATGCTGACTGTTATGCTTTGCCTGCA 2629

B15_BF2_(AM282695) TCCTCTTGACCTCTCACATCTCATTCTGCTTCCTATGCTGACTGTTATGCTTTGCCTGCA 2503

B19_BF2_(AB426151) TCCTCTTGACCTCTCACATCTCATTCTGCTTCCTATGCTGACTGTTATGCTTTGCCTGCA 2525

B19_BF2_(PacBio_P2a) TCCTCTTGACCTCTCACATCTCATTCTGCTTCCTATGCTGACTGTTATGCTTTGCCTGCA 2629

B19_BF2_(AM282696) TCCTCTTGACCTCTCACATCTCATTCTGCTTCCTATGCTGACTGTTATGCTTTGCCTGCA 2503

B21_BF2_(AB426152) TCCTCTTGACCTCTCA**G**AT**G**TC**CC**TCTGCTTCCTATGCTGACTGTTAT**T**CTTTGCCTGCA 2522

B21_BF2_(PacBio_N) TCCTCTTGACCTCTCA**G**AT**G**TC**CC**TCTGCTTCCTATGCTGACTGTTAT**T**CTTTGCCTGCA 2626

B21_BF2_(AM282697) TCCTCTTGACCTCTCA**G**AT**G**TC**CC**TCTGCTTCCTATGCTGACTGTTAT**T**CTTTGCCTGCA 2499

B21_BF2_(PacBio_0) TCCTCTTGACCTCTCA**G**AT**G**TC**CC**TCTGCTTCCTATGCTGACTGTTAT**T**CTTTGCCTGCA 2522

B21_BF2_(AM282700) TCCTCTTGACCTCTCA**G**AT**G**TC**CC**TCTGCTTCCTATGCTGACTGTTAT**T**CTTTGCCTGCA 2499

**BF2 PolyA Site 1**

B2_BF2_(AB426141) CTGCTTCCTGTGAAATAAAATGATGGGCCATTCTGTGCTCAGCTTGCCTGCA**G**TCTGCAC 2578

B2_BF2_(PacBio_6sub1) CTGCTTCCTGTGAAATAAAATGATGGGCCATTCTGTGCTCAGCTTGCCTGCA**G**TCTGCAC 2682

B2_BF2_(AM282692) CTGCTTCCTGTGAAATAAAATGATGGGCCATTCTGTGCTCAGCTTGCCTGCA**G**TCTGCAC 2556

B2_BF2_(PacBio_7sub2) CTGCTTCCTGTGAAATAAAATGATGGGCCATTCTGTGCTCAGCTTGCCTGCA**G**TCTGCAC 2682

B2_BF2_(AM282698) CTGCTTCCTGTGAAATAAAATGATGGGCCATTCTGTGCTCAGCTTGCCTGCA**G**TCTGCAC 2556

B4_BF2_(AM282699) CTGCTTCCTGTGAAATAAAATGATGGGCCATTCTGTGCTCAGCTTGCCTGCATTCTGCAC 2564

B4_BF2_(PacBio_C) CTGCTTCCTGTGAAATAAAATGATGGGCCATTCTGTGCTCAGCTTGCCTGCATTCTGCAC 2687

B4_BF2_(AM282693) CTGCTTCCTGTGAAATAAAATGATGGGCCATTCTGTGCTCAGCTTGCCTGCATTCTGCAC 2564

B12_BF2_(AB426147) CTGCTTCCTGTGAAATAAAATGATGGGCCATTCTGTGCTCAGCTTGCCTGCA**G**TCTGCAC 2578

B12_BF2_(PacBio_C) CTGCTTCCTGTGAAATAAAATGATGGGCCATTCTGTGCTCAGCTTGCCTGCA**G**TCTGCAC 2682

B12_BF2_(AL023516) CTGCTTCCTGTGAAATAAAATGATGGGCCATTCTGTGCTCAGCTTGCCTGCA**G**TCTGCAC 2682

B14_BF2_(PacBio_WL) CTGCTTCCTGTGA**G**ATAAAATGATGGGCCATTCTGT**-**CTCAGCTTGCCT**T**CATTCTGCAC 2698

B14_BF2_(AM282694) CTGCTTCCTGTGA**G**ATAAAATGATGGGCCATTCTGT**-**CTCAGCTTGCCT**T**CATTCTGCAC 2570

B15_BF2_(AB426149) CTGCTTCCTGTGA**G**ATAAAATGATGGGCCATTCTGT**-**CTCAGCTTGCCT**T**CATTCTGCAC 2573

B15_BF2_(PacBio_15I) CTGCTTCCTGTGA**G**ATAAAATGATGGGCCATTCTGT**-**CTCAGCTTGCCT**T**CATTCTGCAC 2688

B15_BF2_(AM282695) CTGCTTCCTGTGA**G**ATAAAATGATGGGCCATTCTGT**-**CTCAGCTTGCCT**T**CATTCTGCAC 2562

B19_BF2_(AB426151) CTGCTTCCTGTGAAATAAAATGATGGGCCATCCTGTGCTCAGCTTGCCTGCATTCTGCAC 2585

B19_BF2_(PacBio_P2a) CTGCTTCCTGTGAAATAAAATGATGGGCCATCCTGTGCTCAGCTTGCCTGCATTCTGCAC 2689

B19_BF2_(AM282696) CTGCTTCCTGTGAAATAAAATGATGGGCCATCCTGTGCTCAGCTTGCCTGCATTCTGCAC 2563

B21_BF2_(AB426152) CTGCTTCCTGTGAAATAAAATGATGGGCCATTCTGTGCTCAGCTTGCCTGCATTCTGCAC 2582

B21_BF2_(PacBio_N) CTGCTTCCTGTGAAATAAAATGATGGGCCATTCTGTGCTCAGCTTGCCTGCATTCTGCAC 2686

B21_BF2_(AM282697) CTGCTTCCTGTGAAATAAAATGATGGGCCATTCTGTGCTCAGCTTGCCTGCATTCTGCAC 2559

B21_BF2_(PacBio_0) CTGCTTCCTGTGAAATAAAATGATGGGCCATTCTGTGCTCAGCTTGCCTGCATTCTGCAC 2582

B21_BF2_(AM282700) CTGCTTCCTGTGAAATAAAATGATGGGCCATTCTGTGCTCAGCTTGCCTGCATTCTGCAC 2559

Fig. S2

B2_BF2_(AB426141) TGTGCTGTGGTTGGGGATGGGGTGG**A**TGAGGGGACCGTGTCCCAGTTTGGCTGCTCAGGG 2638

B2_BF2_(PacBio_6sub1) TGTGCTGTGGTTGGGGATGGGGTGG**A**TGAGGGGACCGTGTCCCAGTTTGGCTGCTCAGGG 2742

B2_BF2_(AM282692) TGTGCTGTGGTTGGGGATGGGGTGG**A**TGAGGGGACCGTGTCCCAGTTTGGCTGCTCAGGG 2616

B2_BF2_(PacBio_7sub2) TGTGCTGTGGTTGGGGATGGGGTGG**A**TGAGGGGACCGTGTCCCAGTTTGGCTGCTCAGGG 2742

B2_BF2_(AM282698) TGTGCTGTGGTTGGGGATGGGGTGG**A**TGAGGGGACCGTGTCCCAGTTTGGCTGCTCAGGG 2616

B4_BF2_(AM282699) TGTGCTGTGGTTGGGGATGGGGTGGGTGAGGGGACCGTGTCCCAGTTTGGCTGCTCAGGG 2624

B4_BF2_(PacBio_C) TGTGCTGTGGTTGGGGATGGGGTGGGTGAGGGGACCGTGTCCCAGTTTGGCTGCTCAGGG 2747

B4_BF2_(AM282693) TGTGCTGTGGTTGGGGATGGGGTGGGTGAGGGGACCGTGTCCCAGTTTGGCTGCTCAGGG 2624

B12_BF2_(AB426147) TGTGCTGTGGTTGGGGATGGGGTGG**A**TGAGGGGACCGTGTCCCAGTTTGGCTGCTCAGGG 2638

B12_BF2_(PacBio_C) TGTGCTGTGGTTGGGGATGGGGTGG**A**TGAGGGGACCGTGTCCCAGTTTGGCTGCTCAGGG 2742

B12_BF2_(AL023516) TGTGCTGTGGTTGGGGATGGGGTGG**A**TGAGGGGACCGTGTCCCAGTTTGGCTGCTCAGGG 2742

B14_BF2_(PacBio_WL) **G**GTGCTGTGGTTGGGGATGGGGTGGGTGAG**A**GGACCGTGTCCCAGTTTGGCTGCTCAGGG 2758

B14_BF2_(AM282694) **G**GTGCTGTGGTTGGGGATGGGGTGGGTGAG**A**GGACCGTGTCCCAGTTTGGCTGCTCAGGG 2630

B15_BF2_(AB426149) **G**GTGCTGTGGTTGGGGATGGGGTGGGTGAGGGGACCGTGTCCCAGTTTGGCTGCTCAGGG 2633

B15_BF2_(PacBio_15I) **G**GTGCTGTGGTTGGGGATGGGGTGGGTGAGGGGACCGTGTCCCAGTTTGGCTGCTCAGGG 2748

B15_BF2_(AM282695) **G**GTGCTGTGGTTGGGGATGGGGTGGGTGAGGGGACCGTGTCCCAGTTTGGCTGCTCAGGG 2622

B19_BF2_(AB426151) TGTGCTGTGGTTGGGGATGGGGTGGGTGAGGGGACCGTGTCCCAGTTTGGCTGCTCAGGG 2645

B19_BF2_(PacBio_P2a) TGTGCTGTGGTTGGGGATGGGGTGGGTGAGGGGACCGTGTCCCAGTTTGGCTGCTCAGGG 2749

B19_BF2_(AM282696) TGTGCTGTGGTTGGGGATGGGGTGGGTGAGGGGACCGTGTCCCAGTTTGGCTGCTCAGGG 2623

B21_BF2_(AB426152) TGTGCTGTGGTTGGGGATGGGGTGGGTGAGGGGACCGTGTCCCAGTTTGGCTGCTCAGGG 2642

B21_BF2_(PacBio_N) TGTGCTGTGGTTGGGGATGGGGTGGGTGAGGGGACCGTGTCCCAGTTTGGCTGCTCAGGG 2746

B21_BF2_(AM282697) TGTGCTGTGGTTGGGGATGGGGTGGGTGAGGGGACCGTGTCCCAGTTTGGCTGCTCAGGG 2619

B21_BF2_(PacBio_0) TGTGCTGTGGTTGGGGATGGGGTGGGTGAGGGGACCGTGTCCCAGTTTGGCTGCTCAGGG 2642

B21_BF2_(AM282700) TGTGCTGTGGTTGGGGATGGGGTGGGTGAGGGGACCGTGTCCCAGTTTGGCTGCTCAGGG 2619

B2_BF2_(AB426141) TGCAGATGTGGCCCTGTGCTGAGTACCCACTGCCCTCCCCTCTTCTATCTGCCTGCTGCT 2698

B2_BF2_(PacBio_6sub1) TGCAGATGTGGCCCTGTGCTGAGTACCCACTGCCCTCCCCTCTTCTATCTGCCTGCTGCT 2802

B2_BF2_(AM282692) TGCAGATGTGGCCCTGTGCTGAGTACCCACTGCCCTCCCCTCTTCTATCTGCCTGCTGCT 2676

B2_BF2_(PacBio_7sub2) TGCAGATGTGGCCCTGTGCTGAGTACCCACTGCCCTCCCCTCTTCTATCTGCCTGCTGCT 2802

B2_BF2_(AM282698) TGCAGATGTGGCCCTGTGCTGAGTACCCACTGCCCTCCCCTCTTCTATCTGCCTGCTGCT 2676

B4_BF2_(AM282699) TGCAGATGTGGCCCTGTGCTGAGTACCCACTGCCCTCCCCTCTTCTATCTGCCTGCTGCT 2684

B4_BF2_(PacBio_C) TGCAGATGTGGCCCTGTGCTGAGTACCCACTGCCCTCCCCTCTTCTATCTGCCTGCTGCT 2807

B4_BF2_(AM282693) TGCAGATGTGGCCCTGTGCTGAGTACCCACTGCCCTCCCCTCTTCTATCTGCCTGCTGCT 2684

B12_BF2_(AB426147) TGCAGATGTGGCCCTGTGCTGAGTACCCACTGCCCTCCCCTCTTCTATCTGCCTGCTGCT 2698

B12_BF2_(PacBio_C) TGCAGATGTGGCCCTGTGCTGAGTACCCACTGCCCTCCCCTCTTCTATCTGCCTGCTGCT 2802

B12_BF2_(AL023516) TGCAGATGTGGCCCTGTGCTGAGTACCCACTGCCCTCCCCTCTTCTATCTGCCTGCTGCT 2802

B14_BF2_(PacBio_WL) TGCAGATGTGGCCCTGTGCTGAGTACCCAC**A**GCCCTCCCC**C**C**--**CTATCTGCCTGCTGCT 2816

B14_BF2_(AM282694) TGCAGATGTGGCCCTGTGCTGAGTACCCAC**A**GCCCTCCCC**C**C**--**CTATCTGCCTGCTGCT 2688

B15_BF2_(AB426149) TGCAGATGTGGCCCTGTGCTGAGTACCCAC**A**GCCCTC**T**CC**C**C**--**CTATCTGCCTGCTGCT 2691

B15_BF2_(PacBio_15I) TGCAGATGTGGCCCTGTGCTGAGTACCCAC**A**GCCCTC**T**CC**C**C**--**CTATCTGCCTGCTGCT 2806

B15_BF2_(AM282695) TGCAGATGTGGCCCTGTGCTGAGTACCCAC**A**GCCCTC**T**CC**C**C**--**CTATCTGCCTGCTGCT 2680

B19_BF2_(AB426151) TGCAGATGTGGCCCTGTGCTGAGTACCCAC**C**GCCCTCCCCTCTTCTATCTGCCTGCTGCT 2705

B19_BF2_(PacBio_P2a) TGCAGATGTGGCCCTGTGCTGAGTACCCAC**C**GCCCTCCCCTCTTCTATCTGCCTGCTGCT 2809

B19_BF2_(AM282696) TGCAGATGTGGCCCTGTGCTGAGTACCCAC**C**GCCCTCCCCTCTTCTATCTGCCTGCTGCT 2683

B21_BF2_(AB426152) TGCAGATGTGGCCCTGTGCTGAGTACCCACTGCCCTCCCC**C---**C**C**ATCTGCCTGCTGCT 2699

B21_BF2_(PacBio_N) TGCAGATGTGGCCCTGTGCTGAGTACCCACTGCCCTCCCC**C---**C**C**ATCTGCCTGCTGCT 2803

B21_BF2_(AM282697) TGCAGATGTGGCCCTGTGCTGAGTACCCACTGCCCTCCCC**C---**C**C**ATCTGCCTGCTGCT 2676

B21_BF2_(PacBio_0) TGCAGATGTGGCCCTGTGCTGAGTACCCACTGCCCTCCCC**C---**C**C**ATCTGCCTGCTGCT 2699

B21_BF2_(AM282700) TGCAGATGTGGCCCTGTGCTGAGTACCCACTGCCCTCCCC**C---**C**C**ATCTGCCTGCTGCT 2676

B2_BF2_(AB426141) CACTCCCCCCTGTGTACCCCCATCCCTTCTCACCTCTCCTCTGTGACCCCATGCTGGTGG 2758

B2_BF2_(PacBio_6sub1) CACTCCCCCCTGTGTACCCCCATCCCTTCTCACCTCTCCTCTGTGACCCCATGCTGGTGG 2862

B2_BF2_(AM282692) CACTCCCCCCTGTGTACCCCCATCCCTTCTCACCTCTCCTCTGTGACCCCATGCTGGTGG 2736

B2_BF2_(PacBio_7sub2) CACTCCCCCCTGTGTACCCCCATCCCTTCTCACCTCTCCTCTGTGACCCCATGCTGGTGG 2862

B2_BF2_(AM282698) CACTCCCCCCTGTGTACCCCCATCCCTTCTCACCTCTCCTCTGTGACCCCATGCTGGTGG 2736

B4_BF2_(AM282699) CACTCCCCCCTGTGTACCCCCATCCCTTCTCACCTCTCCTCTGTGACCCCATGCTGGTGG 2744

B4_BF2_(PacBio_C) CACTCCCCCCTGTGTACCCCCATCCCTTCTCACCTCTCCTCTGTGACCCCATGCTGGTGG 2867

B4_BF2_(AM282693) CACTCCCCCCTGTGTACCCCCATCCCTTCTCACCTCTCCTCTGTGACCCCATGCTGGTGG 2744

B12_BF2_(AB426147) CACTCCCCCCTGTGTACCCCCATCCCTTCTCACCTCTCCTCTGTGACCCCATGCTGGTGG 2758

B12_BF2_(PacBio_C) CACTCCCCCCTGTGTACCCCCATCCCTTCTCACCTCTCCTCTGTGACCCCATGCTGGTGG 2862

B12_BF2_(AL023516) CACTCCCCCCTGTGTACCCCCATCCCTTCTCACCTCTCCTCTGTGACCCCATGCTGGTGG 2862

B14_BF2_(PacBio_WL) CACTC**TTT--**TGT**C**TACCCCC**G**T**T**CCTTCTCACCTCTCCTCTGTGACCCCATGCTGGTGG 2874

B14_BF2_(AM282694) CACTC**TTT--**TGT**C**TACCCCC**G**T**T**CCTTCTCACCTCTCCTCTGTGACCCCATGCTGGTGG 2746

B15_BF2_(AB426149) CACTCCCCCCTGT**C**TACCCCC**G**T**T**CCTTCTCACCTCTCCTCTGTGACCCCATGCTGGTGG 2751

B15_BF2_(PacBio_15I) CACTCCCCCCTGT**C**TACCCCC**G**T**T**CCTTCTCACCTCTCCTCTGTGACCCCATGCTGGTGG 2866

B15_BF2_(AM282695) CACTCCCCCCTGT**C**TACCCCC**G**T**T**CCTTCTCACCTCTCCTCTGTGACCCCATGCTGGTGG 2740

B19_BF2_(AB426151) CACTCCCCCCT**C**TGTACCCCC**G**T**T**CCTTCTCACCTCTCCTCTGTGACCCCATGCTGGTGG 2765

B19_BF2_(PacBio_P2a) CACTCCCCCCT**C**TGTACCCCC**G**T**T**CCTTCTCACCTCTCCTCTGTGACCCCATGCTGGTGG 2869

B19_BF2_(AM282696) CACTCCCCCCT**C**TGTACCCCC**G**T**T**CCTTCTCACCTCTCCTCTGTGACCCCATGCTGGTGG 2743

B21_BF2_(AB426152) CACTCCCCCCTGTGTACCCCCATCCCTTCTCACCTCTCCTCTGTGACCCCATGCTGGTGG 2759

B21_BF2_(PacBio_N) CACTCCCCCCTGTGTACCCCCATCCCTTCTCACCTCTCCTCTGTGACCCCATGCTGGTGG 2863

B21_BF2_(AM282697) CACTCCCCCCTGTGTACCCCCATCCCTTCTCACCTCTCCTCTGTGACCCCATGCTGGTGG 2736

B21_BF2_(PacBio_0) CACTCCCCCCTGTGTACCCCCATCCCTTCTCACCTCTCCTCTGTGACCCCATGCTGGTGG 2759

B21_BF2_(AM282700) CACTCCCCCCTGTGTACCCCCATCCCTTCTCACCTCTCCTCTGTGACCCCATGCTGGTGG 2736

Fig. S2

B2_BF2_(AB426141) TTGCTTGCTCCCTGTCCTGGCAGAACTCT**C**ATTT-TCCCAATGGCATCCCTGTTTGTTGG 2817

B2_BF2_(PacBio_6sub1) TTGCTTGCTCCCTGTCCTGGCAGAACTCT**C**ATTT-TCCCAATGGCATCCCTGTTTGTTGG 2921

B2_BF2_(AM282692) TTGCTTGCTCCCTGTCCTGGCAGAACTCT**C**ATTT-TCCCAATGGCATCCCTGTTTGTTGG 2795

B2_BF2_(PacBio_7sub2) TTGCTTGCTCCCTGTCCTGGCAGAACTCT**C**ATTT-TCCCAATGGCATCCCTGTTTGTTGG 2921

B2_BF2_(AM282698) TTGCTTGCTCCCTGTCCTGGCAGAACTCT**C**ATTT-TCCCAATGGCATCCCTGTTTGTTGG 2795

B4_BF2_(AM282699) TTGCTTGCTCCCTGTCCTGGCAGAACTCT**C**ATTT-TCCCAATGGCATCCCTGTTTGTTGG 2803

B4_BF2_(PacBio_C) TTGCTTGCTCCCTGTCCTGGCAGAACTCT**C**ATTT-TCCCAATGGCATCCCTGTTTGTTGG 2926

B4_BF2_(AM282693) TTGCTTGCTCCCTGTCCTGGCAGAACTCT**C**ATTT-TCCCAATGGCATCCCTGTTTGTTGG 2803

B12_BF2_(AB426147) TTGCTTGCTCCCTGTCCTGGCAGAACTCT**C**ATTT-TCCCAATGGCATCCCTGTTTGTTGG 2817

B12_BF2_(PacBio_C) TTGCTTGCTCCCTGTCCTGGCAGAACTCT**C**ATTT-TCCCAATGGCATCCCTGTTTGTTGG 2921

B12_BF2_(AL023516) TTGCTTGCTCCCTGTCCTGGCAGAACTCT**C**ATTT-TCCCAATGGCATCCCTGTTTGTTGG 2921

B14_BF2_(PacBio_WL) TTGCTTGCTCCCTGTCCTGGCAGAACTCTGATTT**T**TCCCAATGGCATCCCTGTTTGTTGG 2934

B14_BF2_(AM282694) TTGCTTGCTCCCTGTCCTGGCAGAACTCTGATTT**T**TCCCAATGGCATCCCTGTTTGTTGG 2806

B15_BF2_(AB426149) TTGCTTGCTCCCTGTCCTGGCAGAACTCTGATTT**T**TCCCAATGGCATCCCTGTTTGTTGG 2811

B15_BF2_(PacBio_15I) TTGCTTGCTCCCTGTCCTGGCAGAACTCTGATTT**T**TCCCAATGGCATCCCTGTTTGTTGG 2926

B15_BF2_(AM282695) TTGCTTGCTCCCTGTCCTGGCAGAACTCTGATTT**T**TCCCAATGGCATCCCTGTTTGTTGG 2800

B19_BF2_(AB426151) TTGCTTGCTCCCTGTCCTGGCAGAACTCTGATTT-TCCCAATGGCATCCCTG**GG**TGTTGG 2824

B19_BF2_(PacBio_P2a) TTGCTTGCTCCCTGTCCTGGCAGAACTCTGATTT-TCCCAATGGCATCCCTG**GG**TGTTGG 2928

B19_BF2_(AM282696) TTGCTTGCTCCCTGTCCTGGCAGAACTCTGATTT-TCCCAATGGCATCCCTG**GG**TGTTGG 2802

B21_BF2_(AB426152) TTGCTTGCTCCCTGTCCTGGCAGAACTCTGATTT-TCCCAATGGCATCCCTG**GG**TGTTGG 2818

B21_BF2_(PacBio_N) TTGCTTGCTCCCTGTCCTGGCAGAACTCTGATTT-TCCCAATGGCATCCCTG**GG**TGTTGG 2922

B21_BF2_(AM282697) TTGCTTGCTCCCTGTCCTGGCAGAACTCTGATTT-TCCCAATGGCATCCCTG**GG**TGTTGG 2795

B21_BF2_(PacBio_0) TTGCTTGCTCCCTGTCCTGGCAGAACTCTGATTT-TCCCAATGGCATCCCTG**GG**TGTTGG 2818

B21_BF2_(AM282700) TTGCTTGCTCCCTGTCCTGGCAGAACTCTGATTT-TCCCAATGGCATCCCTG**GG**TGTTGG 2795

B2_BF2_(AB426141) GATGTGGTCTCCTTGGTCCTCCCCCCAGCAGTCACTGCACATATCCACCCCACTTCCCCC 2877

B2_BF2_(PacBio_6sub1) GATGTGGTCTCCTTGGTCCTCCCCCCAGCAGTCACTGCACATATCCACCCCACTTCCCCC 2981

B2_BF2_(AM282692) GATGTGGTCTCCTTGGTCCTCCCCCCAGCAGTCACTGCACATATCCACCCCACTTCCCCC 2855

B2_BF2_(PacBio_7sub2) GATGTGGTCTCCTTGGTCCTCCCCCCAGCAGTCACTGCACATATCCACCCCACTTCCCCC 2981

B2_BF2_(AM282698) GATGTGGTCTCCTTGGTCCTCCCCCCAGCAGTCACTGCACATATCCACCCCACTTCCCCC 2855

B4_BF2_(AM282699) GATGTGGTCTCCTTGGTCCTCCCCCCAGCAGTCACTGCACATATCCACCCCACTTCCCCC 2863

B4_BF2_(PacBio_C) GATGTGGTCTCCTTGGTCCTCCCCCCAGCAGTCACTGCACATATCCACCCCACTTCCCCC 2986

B4_BF2_(AM282693) GATGTGGTCTCCTTGGTCCTCCCCCCAGCAGTCACTGCACATATCCACCCCACTTCCCCC 2863

B12_BF2_(AB426147) GATGTGGTCTCCTTGGTCCTCCCCCCAGCAGTCACTGCACATATCCACCCCACTTCCCCC 2877

B12_BF2_(PacBio_C) GATGTGGTCTCCTTGGTCCTCCCCCCAGCAGTCACTGCACATATCCACCCCACTTCCCCC 2981

B12_BF2_(AL023516) GATGTGGTCTCCTTGGTCCTCCCCCCAGCAGTCACTGCACATATCCACCCCACTTCCCCC 2981

B14_BF2_(PacBio_WL) GATGTGGTCTCCTTGGTCCTCCCCCCAGCAGTCACTGCACATATCCACCCCACTTCCCCC 2994

B14_BF2_(AM282694) GATGTGGTCTCCTTGGTCCTCCCCCCAGCAGTCACTGCACATATCCACCCCACTTCCCCC 2866

B15_BF2_(AB426149) GATGTGGTCTCCTTGGTCCTCCCCCCAGCAGTCACTGCACATATCCACCCCACTTCCCCC 2871

B15_BF2_(PacBio_15I) GATGTGGTCTCCTTGGTCCTCCCCCCAGCAGTCACTGCACATATCCACCCCACTTCCCCC 2986

B15_BF2_(AM282695) GATGTGGTCTCCTTGGTCCTCCCCCCAGCAGTCACTGCACATATCCACCCCACTTCCCCC 2860

B19_BF2_(AB426151) GATGTGGTCTCCTTGGTCCTCCCCCCAGCAGTCACTGCACATATCCACCCCACTTCCCCC 2884

B19_BF2_(PacBio_P2a) GATGTGGTCTCCTTGGTCCTCCCCCCAGCAGTCACTGCACATATCCACCCCACTTCCCCC 2988

B19_BF2_(AM282696) GATGTGGTCTCCTTGGTCCTCCCCCCAGCAGTCACTGCACATATCCACCCCACTTCCCCC 2862

B21_BF2_(AB426152) GATGTGGTCTCCTTGGTCCTCCCCCCAGCAGTCACTGCACATATCCACCCCACTTCCCCC 2878

B21_BF2_(PacBio_N) GATGTGGTCTCCTTGGTCCTCCCCCCAGCAGTCACTGCACATATCCACCCCACTTCCCCC 2982

B21_BF2_(AM282697) GATGTGGTCTCCTTGGTCCTCCCCCCAGCAGTCACTGCACATATCCACCCCACTTCCCCC 2855

B21_BF2_(PacBio_0) GATGTGGTCTCCTTGGTCCTCCCCCCAGCAGTCACTGCACATATCCACCCCACTTCCCCC 2878

B21_BF2_(AM282700) GATGTGGTCTCCTTGGTCCTCCCCCCAGCAGTCACTGCACATATCCACCCCACTTCCCCC 2855

B2_BF2_(AB426141) CCAGGTTGCTGTCCCACAGCACTCCTATTTCC**T**TCTC**TG**CC**C**TCC-**---**----------- 2922

B2_BF2_(PacBio_6sub1) CCAGGTTGCTGTCCCACAGCACTCCTATTTCC**T**TCTC**TG**CC**C**TCC-**---**----------- 3026

B2_BF2_(AM282692) CCAGGTTGCTGTCCCACAGCACTCCTATTTCC**T**TCTC**TG**CC**C**TCC-**---**----------- 2900

B2_BF2_(PacBio_7sub2) CCAGGTTGCTGTCCCACAGCACTCCTATTTCC**T**TCTC**TG**CC**C**TCC-**---**----------- 3026

B2_BF2_(AM282698) CCAGGTTGCTGTCCCACAGCACTCCTATTTCC**T**TCTC**TG**CC**C**TCC-**---**----------- 2900

B4_BF2_(AM282699) CCAGGTTGCTGTCCCACAGCACTCCTATTTCCCTCTC**TG**CC**C**TCC-**---**----------- 2908

B4_BF2_(PacBio_C) CCAGGTTGCTGTCCCACAGCACTCCTATTTCCCTCTC**TG**CC**C**TCC-**---**----------- 3031

B4_BF2_(AM282693) CCAGGTTGCTGTCCCACAGCACTCCTATTTCCCTCTC**TG**CC**C**TCC-**---**----------- 2908

B12_BF2_(AB426147) CCAGGTTGCTGTCCCACAGCACTCCTATTTCC**T**TCTC**TG**CC**C**TCC-**---**----------- 2922

B12_BF2_(PacBio_C) CCAGGTTGCTGTCCCACAGCACTCCTATTTCC**T**TCTC**TG**CC**C**TCC-**---**----------- 3026

B12_BF2_(AL023516) CCAGGTTGCTGTCCCACAGCACTCCTATTTCC**T**TCTC**TG**CC**C**TCC-**---**----------- 3026

B14_BF2_(PacBio_WL) CCAGGTTGCTGTCCCACAGCACTCCTATTTCCCTCTCC**C**CCTTCC-CCC----------- 3042

B14_BF2_(AM282694) CCAGGTTGCTGTCCCACAGCACTCCTATTTCCCTCTCC**C**C**-**TTCC-CCC----------- 2913

B15_BF2_(AB426149) CCAGGTTGCTGTCCCACAGCACTCCTATTTCCCTCTCCTCCTTCCACCCCCTTCCCCCCC 2931

B15_BF2_(PacBio_15I) CCAGGTTGCTGTCCCACAGCACTCCTATTTCCCTCTCCTCCTTCCACCCCCTTCCCCCCC 3046

B15_BF2_(AM282695) CCAGGTTGCTGTCCCACAGCACTCCTATTTCCCTCTCCTCCTTCCACCCCCTTCCCCCCC 2920

B19_BF2_(AB426151) CCAGGTTGCTGTCCCACAGCACTCCTATTTCCCTCTCCTCCTTCCACCCCCTTTCCCCCC 2944

B19_BF2_(PacBio_P2a) CCAGGTTGCTGTCCCACAGCACTCCTATTTCCCTCTCCTCCTTCCACCCCCTTTCCCCCC 3048

B19_BF2_(AM282696) CCAGGTTGCTGTCCCACAGCACTCCTATTTCCCTCTCCTCCTTCCACCCCCTTTCCCCCC 2922

B21_BF2_(AB426152) CCAGGTTG**T**TGTCCCACA**C**CACTCCTATTTCCCTCTCCTCCTTCCACCCCCTTCTCCCCC 2938

B21_BF2_(PacBio_N) CCAGGTTG**T**TGTCCCACA**C**CACTCCTATTTCCCTCTCCTCCTTCCACCCCCTTCTCCCCC 3042

B21_BF2_(AM282697) CCAGGTTG**T**TGTCCCACA**C**CACTCCTATTTCCCTCTCCTCCTTCCACCCCCTTCTCCCCC 2915

B21_BF2_(PacBio_0) CCAGGTTG**T**TGTCCCACA**C**CACTCCTATTTCCCTCTCCTCCTTCCACCCCCTTCCCCCCC 2938

B21_BF2_(AM282700) CCAGGTTG**T**TGTCCCACA**C**CACTCCTATTTCCCTCTCCTCCTTCCACCCCCTTCTCCCCC 2915

Fig. S2

B2_BF2_(AB426141) --------CCCCCCGCCCATCCAGCTGCCTCTGCAATCCTCACCCACAACCTTGCCCACT 2974

B2_BF2_(PacBio_6sub1) --------CCCCCCGCCCATCCAGCTGCCTCTGCAATCCTCACCCACAACCTTGCCCACT 3078

B2_BF2_(AM282692) --------CCCCCCGCCCATCCAGCTGCCTCTGCAATCCTCACCCACAACCTTGCCCACT 2952

B2_BF2_(PacBio_7sub2) --------CCCCCCGCCCATCCAGCTGCCTCTGCAATCCTCACCCACAACCTTGCCCACT 3078

B2_BF2_(AM282698) --------CCCCCCGCCCATCCAGCTGCCTCTGCAATCCTCACCCACAACCTTGCCCACT 2952

B4_BF2_(AM282699) --------CCCCCCGCCCATCCAGCTGCCTCTGCAATCCTCACCCACAACCTTGCCCACT 2960

B4_BF2_(PacBio_C) -------CCCCCCCGCCCATCCAGCTGCCTCTGCAATCCTCACCCACAACCTTGCCCACT 3084

B4_BF2_(AM282693) --------CCCCCCGCCCATCCAGCTGCCTCTGCAATCCTCACCCACAACCTTGCCCACT 2960

B12_BF2_(AB426147) --------CCCCCCGCCCATCCAGCTGCCTCTGCAATCCTCACCCACAACCTTGCCCACT 2974

B12_BF2_(PacBio_C) --------CCCCCCGCCCATCCAGCTGCCTCTGCAATCCTCACCCACAACCTTGCCCACT 3078

B12_BF2_(AL023516) --------CCCCCCGCCCATCCAGCTGCCTCTGCAATCCTCACCCACAACCTTGCCCACT 3078

B14_BF2_(PacBio_WL) ------CCCCCCCCGCCCATCCAGCTGCCTCTGCAATCCTCACCCACAACCTTGCCCACT 3096

B14_BF2_(AM282694) ------CCCCCCCCGCCCATCCAGCTGCCTCTGCAATCCTCACCCACAACCTTGCCCACT 2967

B15_BF2_(AB426149) **CCCCCC**CCCCCCCC**C**NNNNNNNNNNNNNNNNNNNNNNNNNNNNNNNNNNNNNNNNNNNNN 2991

B15_BF2_(PacBio_15I) -**CCCCC**CCCCCCCC**C**CCCATCCAGCTGCCTCTGCAATCCTCACCCACAACCTCGCCCACT 3105

B15_BF2_(AM282695) **CCCCCC**CCCTCCCC**C**CCCATCCAGCTGCCTCTGCAATCCTCACCCACAACCTCGCCCACT 2980

B19_BF2_(AB426151) **CCCC**NNNNNNNNNNNNNNNNNNNNNNNNNNNNNNNNNNNNNNNNNNNNNNNNNNNNNNNN 3004

B19_BF2_(PacBio_P2a) ---**CCC**CCCCCCCCGCCC**C**TCCAGCTGCCTCTGCAATCCTCACCCACAACCTTGCCCACT 3105

B19_BF2_(AM282696) -----**C**CCCCCCCCGCCC**C**TCCAGCTGCCTCTGCAATCCTCACCCACAACCTTGCCCACT 2977

B21_BF2_(AB426152) ---**CCC**CCCCCCCC**C**CCCATCCAGCTGCCTCTGCAATCCTCACCCACAACCTTGCCCACT 2995

B21_BF2_(PacBio_N) ---**CCC**CCCCCCCC**C**CCCATCCAGCTGCCTCTGCAATCCTCACCCACAACCTTGCCCACT 3099

B21_BF2_(AM282697) -----**C**CCCCCCCC**C**CCCATCCAGCTGCCTCTGCAATCCTCACCCACAACCTTGCCCACT 2970

B21_BF2_(PacBio_0) -----**C**CCCCCCCC**C**CCCATCCAGCTGCCTCTGCAATCCTCACCCACAACCTTGCCCACT 2993

B21_BF2_(AM282700) -----**C**CCCCCCCC**C**CCCATCCAGCTGCCTCTGCAATCCTCACCCACAACCTTGCCCACT 2970

B2_BF2_(AB426141) CCACCTCCCTCATCCCGCCCTTCCCCCAGCTCTCCTGTCCCTGCTGGGCCCCCTCCCCCC 3034

B2_BF2_(PacBio_6sub1) CCACCTCCCTCATCCCGCCCTTCCCCCAGCTCTCCTGTCCCTGCTGGGCCCCCTCCCCCC 3138

B2_BF2_(AM282692) CCACCTCCCTCATCCCGCCCTTCCCCCAGCTCTCCTGTCCCTGCTGGGCCCCCTCCCCCC 3012

B2_BF2_(PacBio_7sub2) CCACCTCCCTCATCCCGCCCTTCCCCCAGCTCTCCTGTCCCTGCTGGGCCCCCTCCCCCC 3138

B2_BF2_(AM282698) CCACCTCCCTCATCCCGCCCTTCCCCCAGCTCTCCTGTCCCTGCTGGGCCCCCTCCCCCC 3012

B4_BF2_(AM282699) CCACCTCCCTCATCCCGCCCTTCCCCCAGCTCTCCTGTCCCTGCTGGGCCCCCTCCCCCC 3020

B4_BF2_(PacBio_C) CCACCTCCCTCATCCCGCCCTTCCCCCAGCTCTCCTGTCCCTGCTGGGCCCCCTCCCCCC 3144

B4_BF2_(AM282693) CCACCTCCCTCATCCCGCCCTTCCCCCAGCTCTCCTGTCCCTGCTGGGCCCCCTCCCCCC 3020

B12_BF2_(AB426147) CCACCTCCCTCATCCCGCCCTTCCCCCAGCTCTCCTGTCCCTGCTGGGCCCCCTCCCCCC 3034

B12_BF2_(PacBio_C) CCACCTCCCTCATCCCGCCCTTCCCCCAGCTCTCCTGTCCCTGCTGGGCCCCCTCCCCCC 3138

B12_BF2_(AL023516) CCACCTCCCTCATCCCGCCCTTCCCCCAGCTCTCCTGTCCCTGCTGGGCCCCCTCCCCCC 3138

B14_BF2_(PacBio_WL) CCACCTCCCTCATCCCGCCCTTCCCCCAGCTCTCCTGTCCCTGCTGGGCCCCCTCCCCCC 3156

B14_BF2_(AM282694) CCACCTCCCTCATCCCGCCCTTCCCCCAGCTCTCCTGTCCCTGCTGGGCCCCCTCCCCCC 3027

B15_BF2_(AB426149) NNNNNNNNNNNNNNNNNNNNNNNNNNNNNNNNNNNNNNNNNNNNNNNNNNNNNNNNN--N 3049

B15_BF2_(PacBio_15I) CCACCTCCCTCATCCCGCCCTTCCCCCAGCTCTCCTGTCCCTGCTGGGCCCCCTCCCCCC 3165

B15_BF2_(AM282695) CCACCTCCCTCATCCCGCCCTTCCCCCAGCTCTCCTGTCCCTGCTGGGCCCCCTCCCCCC 3040

B19_BF2_(AB426151) NNNNNNNNNNNNNNNNNNNNNNNNNNNNNNNNNNNNN----NNNNNNNN-NNNNNNN--N 3057

B19_BF2_(PacBio_P2a) CCACCGCCCTCATCCCGCCCTTCCCCCAGCTCTCCTGTCCCTGCTGGGCCCCCTCCCCCC 3165

B19_BF2_(AM282696) CCACCGCCCTCATCCCGCCCTTCCCCCAGCTCTCCTGTCCCTGCTGGGCCCCCTCCCCCC 3037

B21_BF2_(AB426152) CCACCTCCCTCATCCCGCCCTTCCCCCAGCTCTCCTGTCCCTGCTGGGCCCCCTCCCC**T**C 3055

B21_BF2_(PacBio_N) CCACCTCCCTCATCCCGCCCTTCCCCCAGCTCTCCTGTCCCTGCTGGGCCCCCTCCCC**T**C 3159

B21_BF2_(AM282697) CCACCTCCCTCATCCCGCCCTTCCCCCAGCTCTCCTGTCCCTGCTGGGCCCCCTCCCC**T**C 3030

B21_BF2_(PacBio_0) CCACCTCCCTCATCCCGCCCTTCCCCCAGCTCTCCTGTCCCTGCTGGGCCCCCTCCCC**T**C 3053

B21_BF2_(AM282700) CCACCTCCCTCATCCCGCCCTTCCCCCAGCTCTCCTGTCCCTGCTGGGCCCCCTCCCC**T**C 3030

**BF2 PolyA Site 2**

B2_BF2_(AB426141) ACATTGTACCCTACACCCAAATAAATACGTTTGTTCTGCTGCCCTCCAGCCGTCTTCTGG 3094

B2_BF2_(PacBio_6sub1) ACATTGTACCCTACACCCAAATAAATACGTTTGTTCTGCTGCCCTCCAGCCGTCTTCTGG 3198

B2_BF2_(AM282692) ACATTGTACCCTACACCCAAATAAATACGTTTGTTCTGCTGCCCTCCAGCCGTCTTCTGG 3072

B2_BF2_(PacBio_7sub2) ACATTGTACCCTACACCCAAATAAATACGTTTGTTCTGCTGCCCTCCAGCCGTCTTCTGG 3198

B2_BF2_(AM282698) ACATTGTACCCTACACCCAAATAAATACGTTTGTTCTGCTGCCCTCCAGCCGTCTTCTGG 3072

B4_BF2_(AM282699) ACATTGTACCCTACACCCAAATAAATA**T**GTTTGTTCTGCTGCCCTCCAGCCGTCT**C**CTGG 3080

B4_BF2_(PacBio_C) ACATTGTACCCTACACCCAAATAAATA**T**GTTTGTTCTGCTGCCCTCCAGCCGTCT**C**CTGG 3204

B4_BF2_(AM282693) ACATTGTACCCTACACCCAAATAAATA**T**GTTTGTTCTGCTGCCCTCCAGCCGTCT**C**CTGG 3080

B12_BF2_(AB426147) ACATTGTACCCTACACCCAAATAAATACGTTTGTTCTGCTGCCCTCCAGCCGTCTTCTGG 3094

B12_BF2_(PacBio_C) ACATTGTACCCTACACCCAAATAAATACGTTTGTTCTGCTGCCCTCCAGCCGTCTTCTGG 3198

B12_BF2_(AL023516) ACATTGTACCCTACACCCAAATAAATACGTTTGTTCTGCTGCCCTCCAGCCGTCTTCTGG 3198

B14_BF2_(PacBio_WL) ACATTGTACCCTACACCCAAATAAATACGTTTGTTCTGCTGCCCTCCAGCCGTCT**C**CTGG 3216

B14_BF2_(AM282694) ACATTGTACCCTACACCCAAATAAATACGTTTGTTCTGCTGCCCTCCAGCCGTCT**C**CTGG 3087

B15_BF2_(AB426149) NNNNNNNNNN-NNNNNNNNNNNNNNNNNNNNNNNNNNNNNNNNNNNNNNNNNNNNNNNNN 3108

B15_BF2_(PacBio_15I) ACATTGTACCCTACACCCAAATAAATA**T**GTTTGTTCTGCTGCCCTC**T**AGCCGTCT**C**CTGG 3225

B15_BF2_(AM282695) ACATTGTACCCTACACCCAAATAAATA**T**GTTTGTTCTGCTGCCCTC**T**AGCCGTCT**C**CTGG 3100

B19_BF2_(AB426151) NNNNNNNNNNNNNNNNNNNNNNNNNNNNNNNNNNNNNNNNNN-NNNNNN-NNNNNNNNNN 3115

B19_BF2_(PacBio_P2a) ACATTGTACCCTACACCCAAATAAATA**T**GTTTGTTCTGCTGCCCTCCAGCTGTCCTCTGG 3225

B19_BF2_(AM282696) ACATTGTACCCTACACCCAAATAAATA**T**GTTTGTTCTGCTGCCCTCCAGCTGTCCTCTGG 3097

B21_BF2_(AB426152) ACATTGTACCCTACACCCAAATAAATACGTTTGTTCTGCTGCCCTCCAGCCGTCTTCT**A**G 3115

B21_BF2_(PacBio_N) ACATTGTACCCTACACCCAAATAAATACGTTTGTTCTGCTGCCCTCCAGCCGTCTTCT**A**G 3219

B21_BF2_(AM282697) ACATTGTACCCTACACCCAAATAAATACGTTTGTTCTGCTGCCCTCCAGCCGTCTTCT**A**G 3090

B21_BF2_(PacBio_0) ACATTGTACCCTACACCCAAATAAATACGTTTGTTCTGCTGCCCTCCAGCCGTCTTCT**A**G 3113

B21_BF2_(AM282700) ACATTGTACCCTACACCCAAATAAATACGTTTGTTCTGCTGCCCTCCAGCCGTCTTCT**A**G 3090

Fig. S2

B2_BF2_(AB426141) TTTATTTCCCCCC**C**GATTTGTTGTTGTTG**T**GGGCTCCGCTCTTC**T**CCCTGGGGGGAAGGG 3154

B2_BF2_(PacBio_6sub1) TTTATTTCCCCCC**C**GATTTGTTGTTGTTG**T**GGGCTCCGCTCTTC**T**CCCTGGGGGGAAGGG 3258

B2_BF2_(AM282692) ------------------------------------------------------------ 3072

B2_BF2_(PacBio_7sub2) TTTATTTCCCCCC**C**GATTTGTTGTTGTTG**T**GGGCTCCGCTCTTC**T**CCCTGGGGGGAAGGG 3258

B2_BF2_(AM282698) ------------------------------------------------------------ 3072

B4_BF2_(AM282699) ------------------------------------------------------------ 3080

B4_BF2_(PacBio_C) TTTATTTCCCCCC-GATTTGTTGTTGTTGGGGGCTCCGCTCTTCACCCTGGGGGGAAGGG 3263

B4_BF2_(AM282693) ------------------------------------------------------------ 3080

B12_BF2_(AB426147) TTTATTTCCCCCC**C**GATTTGTTGTTGTTGTGGGCTCCGCTCTTCTCCCTGGGGGGAAGGG 3154

B12_BF2_(PacBio_C) TTTATTTCCCCCC**C**GATTTGTTGTTGTTGTGGGCTCCGCTCTTCTCCCTGGGGGGAAGGG 3258

B12_BF2_(AL023516) TTTATTTCCCCCC**C**GATTTGTTGTTGTTGTGGGCTCCGCTCTTCTCCCTGGGGGGAAGGG 3258

B14_BF2_(PacBio_WL) TTTATTTCCCCCC-GATTTGTTGTTGTTGGGGGCTCCGCTCTTCACCCTGGGGGGAAGGG 3275

B14_BF2_(AM282694) ------------------------------------------------------------ 3087

B15_BF2_(AB426149) NNNNNNNNNNNNN-NNNNNNNNNNNNNNNNNNNNNNNNNNNNNNNNN-NNNN-NNNNNNN 3165

B15_BF2_(PacBio_15I) TTTATTTCCCCCC-GATTTGTTGTTGTTGGGGGCTCCGCTCTTCACCCTGGGGGGAAGGG 3284

B15_BF2_(AM282695) ------------------------------------------------------------ 3100

B19_BF2_(AB426151) NNNNNNNNNNNNN-NNNNNNNNNNNNNNNNNNNNN-NNNNNNNNNNN-NNNNNNNNNNNN 3172

B19_BF2_(PacBio_P2a) TTTATTTCCCCCC**C**GATTTGTTGTTGTTGGGGGCTCCGCTCTTCACCCTGGGGGGAAGGG 3285

B19_BF2_(AM282696) ------------------------------------------------------------ 3097

B21_BF2_(AB426152) TTTATTTCCCCCC-GATTTGTTGTTGTTGGGGGCTCCGCTCTTCACCCTGGGGGGAAGGG 3174

B21_BF2_(PacBio_N) TTTATTTCCCCCC-GATTTGTTGTTGTTGGGGGCTCCGCTCTTCACCCTGGGGGGAAGGG 3278

B21_BF2_(AM282697) ------------------------------------------------------------ 3090

B21_BF2_(PacBio_0) TTTATTTCCCCCC-GATTTGTTGTTGTTGGGGGCTCCGCTCTTCACCCTGGGGGGAAGGG 3172

B21_BF2_(AM282700) ------------------------------------------------------------ 3090

B2_BF2_(AB426141) GCTCTGGGGGTCCCTCATTCTCCCTGCACTTCTTACAGCACCGGGACTCCCCGCGCTGAG 3214

B2_BF2_(PacBio_6sub1) GCTCTGGGGGTCCCTCATTCTCCCTGCACTTCTTACAGCACCGGGACTCCCCGCGCTGAG 3318

B2_BF2_(AM282692) ------------------------------------------------------------ 3072

B2_BF2_(PacBio_7sub2) GCTCTGGGGGTCCCTCATTCTCCCTGCACTTCTTACAGCACCGGGACTCCCCGCGCTGAG 3318

B2_BF2_(AM282698) ------------------------------------------------------------ 3072

B4_BF2_(AM282699) ------------------------------------------------------------ 3080

B4_BF2_(PacBio_C) GCTCTGGGGGTCCCTCATTCTCCCTGCACTTCTTACAGCACCGGGACTCCCCGCGCTGAG 3323

B4_BF2_(AM282693) ------------------------------------------------------------ 3080

B12_BF2_(AB426147) GCTCTGGGGGTCCCTCATTCTCCCTGCACTTCTTACAGCACCGGGACTCCCCGCGCTGAG 3214

B12_BF2_(PacBio_C) GCTCTGGGGGTCCCTCATTCTCCCTGCACTTCTTACAGCACCGGGACTCCCCGCGCTGAG 3318

B12_BF2_(AL023516) GCTCTGGGGGTCCCTCATTCTCCCTGCACTTCTTACAGCACCGGGACTCCCCGCGCTGAG 3318

B14_BF2_(PacBio_WL) GCTCTGGGGGTCCCTCATTCTCCCTGCACTTCTTACAGCACCGGGACTCCCCGCGCTGAG 3335

B14_BF2_(AM282694) ------------------------------------------------------------ 3087

B15_BF2_(AB426149) NNNNNNNNNNNNNNNNNNNNNNNNNNNNNNNNNNNNNNNNNNNNNNNNNNNNNNNNNNNN 3225

B15_BF2_(PacBio_15I) GCTCTGGGGGTCCCTCATTCTCCCTGCACTTCTTACAGCATCGGGACTCCCCGCGCTGAG 3344

B15_BF2_(AM282695) ------------------------------------------------------------ 3100

B19_BF2_(AB426151) NNNNNNN-NNN---NNNNNNNNNNNNNNNNNNNNNNNNNNNNNNNNNNNNNNNNNNNNNN 3228

B19_BF2_(PacBio_P2a) GCTCTGGGGGTCCCTCATTCTCCCTGCACTTCTTACAGCACCGGGACTCCCCGCGCTGAG 3345

B19_BF2_(AM282696) ------------------------------------------------------------ 3097

B21_BF2_(AB426152) GCTCTGGGGGTCCCTCATTCTCCCTGCACTTCTTACAGCACCGGGACTCCCCGCGCTGAG 3234

B21_BF2_(PacBio_N) GCTCTGGGGGTCCCTCATTCTCCCTGCACTTCTTACAGCACCGGGACTCCCCGCGCTGAG 3338

B21_BF2_(AM282697) ------------------------------------------------------------ 3090

B21_BF2_(PacBio_0) GCTCTGGGGGTCCCTCATTCTCCCTGCACTTCTTACAGCACCGGGACTCCCCGCGCTGAG 3232

B21_BF2_(AM282700) ------------------------------------------------------------ 3090

B2_BF2_(AB426141) ATCCCAACACACCCGGGTACAAACATGCGGCTTTATTCCCAGTTCTGTGTCCCACCCCCG 3274

B2_BF2_(PacBio_6sub1) ATCCCAACACACCCGGGTACAAACATGCGGCTTTATTCCCAGTTCTGTGTCCCACCCCCG 3378

B2_BF2_(AM282692) ------------------------------------------------------------ 3072

B2_BF2_(PacBio_7sub2) ATCCCAACACACCCGGGTACAAACATGCGGCTTTATTCCCAGTTCTGTGTCCCACCCCCG 3378

B2_BF2_(AM282698) ------------------------------------------------------------ 3072

B4_BF2_(AM282699) ------------------------------------------------------------ 3080

B4_BF2_(PacBio_C) ATCCCAACACACCCGGGTACAAACATGCGGCTTTATTCCCAGTTCTGTGTCCCACCCCCG 3383

B4_BF2_(AM282693) ------------------------------------------------------------ 3080

B12_BF2_(AB426147) ATCCCAACACACCCGGGTACAAACATGCGGCTTTATTCCCAGTTCTGTGTCCCACCCCCG 3274

B12_BF2_(PacBio_C) ATCCCAACACACCCGGGTACAAACATGCGGCTTTATTCCCAGTTCTGTGTCCCACCCCCG 3378

B12_BF2_(AL023516) ATCCCAACACACCCGGGTACAAACATGCGGCTTTATTCCCAGTTCTGTGTCCCACCCCCG 3378

B14_BF2_(PacBio_WL) ATCCCAACACACCCGGGTACAAACATGCGGCTTTATTCCCAGTTCTGTGTCCCACCCCCG 3395

B14_BF2_(AM282694) ------------------------------------------------------------ 3087

B15_BF2_(AB426149) NNNNNNNNNNNNNNNNNNNNNNNNNNNNNNNNNNNNNNNNNNNNNNNNNNNNNNNNNNNN 3285

B15_BF2_(PacBio_15I) ATCCCAACACACCCGGGTACAAACATGCGGCTTTATTCCCAGTTCTGTGTCCCACCCCCG 3404

B15_BF2_(AM282695) ------------------------------------------------------------ 3100

B19_BF2_(AB426151) NNNNNNNNNNNNNNNNNNNNNNNNNNNNNNNNNNNNNNNNNNNNNNNNNNNNNNNNNNNN 3288

B19_BF2_(PacBio_P2a) ATCCCAACACACCCGGGTACAAACATGCGGCTTTATTCCCAGTTCTGTGTCCCACCCCCG 3405

B19_BF2_(AM282696) ------------------------------------------------------------ 3097

B21_BF2_(AB426152) ATCCCAACACACCCGGGTACAAACATGCGGCTTTATTCCCAGTTCTGTGTCCCACCCCCG 3294

B21_BF2_(PacBio_N) ATCCCAACACACCCGGGTACAAACATGCGGCTTTATTCCCAGTTCTGTGTCCCACCCCCG 3398

B21_BF2_(AM282697) ------------------------------------------------------------ 3090

B21_BF2_(PacBio_0) ATCCCAACACACCCGGGTACAAACATGCGGCTTTATTCCCAGTTCTGTGTCCCACCCCCG 3292

B21_BF2_(AM282700) ------------------------------------------------------------ 3090

Fig. S2

B2_BF2_(AB426141) GCCCTGGTGGCACTCAGTGGCACTGCAGTCCATGCAGTGGCCGTTGTGTGTCGTACAGCA 3334

B2_BF2_(PacBio_6sub1) GCCCTGGTGGCACTCAGTGGCACTGCAGTCCATGCAGTGGCCGTTGTGTGTCGTACAGCA 3438

B2_BF2_(AM282692) ------------------------------------------------------------ 3072

B2_BF2_(PacBio_7sub2) GCCCTGGTGGCACTCAGTGGCACTGCAGTCCATGCAGTGGCCGTTGTGTGTCGTACAGCA 3438

B2_BF2_(AM282698) ------------------------------------------------------------ 3072

B4_BF2_(AM282699) ------------------------------------------------------------ 3080

B4_BF2_(PacBio_C) GCCCTGGTGGCACTCAGTGGCACCGCAGTCCATGC**T**GTGGCCGTTGTGTGTCGTACAGCA 3443

B4_BF2_(AM282693) ------------------------------------------------------------ 3080

B12_BF2_(AB426147) GCCCTGGTGGCACTCAGTGGCACTGCAGTCCATGCAGTGGCCGTTGTGTGTCGTACAGCA 3334

B12_BF2_(PacBio_C) GCCCTGGTGGCACTCAGTGGCACTGCAGTCCATGCAGTGGCCGTTGTGTGTCGTACAGCA 3438

B12_BF2_(AL023516) GCCCTGGTGGCACTCAGTGGCACTGCAGTCCATGCAGTGGCCGTTGTGTGTCGTACAGCA 3438

B14_BF2_(PacBio_WL) GCCCTGGTGGCACTCAGTGGCAC**C**GCAGTCCATGC**T**GTGGCCGTTGTGTGTCGTACAGCA 3455

B14_BF2_(AM282694) ------------------------------------------------------------ 3087

B15_BF2_(AB426149) NNNNNNNNNNNNNNNNNTGGCACTGCAGTCCATGCAGTGGCCGTTGTGTGTCGTACAGCA 3345

B15_BF2_(PacBio_15I) GCCCTGGTGGCACTCAGTGGCACTGCAGTCCATGCAGTGGCCGTTGTGTGTCGTACAGCA 3464

B15_BF2_(AM282695) ------------------------------------------------------------ 3100

B19_BF2_(AB426151) NNNNNNNTGGCACTCAGTGGCAC**C**GCAGTCCATGCAGTGGCCGTTGTGTGTCGTACAGCA 3348

B19_BF2_(PacBio_P2a) GCCCTGGTGGCACTCAGTGGCAC**C**GCAGTCCATGCAGTGGCCGTTGTGTGTCGTACAGCA 3465

B19_BF2_(AM282696) ------------------------------------------------------------ 3097

B21_BF2_(AB426152) GCCCCGGTGGCACTCAGTGGCACTGCAGTCCATGCAGT**A**GCCGTTGTGTGTCGTACAGCA 3354

B21_BF2_(PacBio_N) GCCCCGGTGGCACTCAGTGGCACTGCAGTCCATGCAGTGGCCGTTGTGTGTCGTACAGCA 3458

B21_BF2_(AM282697) ------------------------------------------------------------ 3090

B21_BF2_(PacBio_0) GCCCCGGTGGCACTCAGTGGCACTGCAGTCCATGCAGT**A**GCCGTTGTGTGTCGTACAGCA 3352

B21_BF2_(AM282700) ------------------------------------------------------------ 3090

B2_BF2_(AB426141) GCGGTACCGCAGCGCGCCCGGCTCGGCATCCATGTGCCCACGGCACAGCTCTTGTGGTCC 3394

B2_BF2_(PacBio_6sub1) GCGGTACCGCAGCGCGCCCGGCTCGGCATCCATGTGCCCACGGCACAGCTCTTGTGGTCC 3498

B2_BF2_(AM282692) ------------------------------------------------------------ 3072

B2_BF2_(PacBio_7sub2) GCGGTACCGCAGCGCGCCCGGCTCGGCATCCATGTGCCCACGGCACAGCTCTTGTGGTCC 3498

B2_BF2_(AM282698) ------------------------------------------------------------ 3072

B4_BF2_(AM282699) ------------------------------------------------------------ 3080

B4_BF2_(PacBio_C) GCGGTACCGCAGCGCGCCCGGCTCGGCATCCATGTGCCCACGGCACAGCTCTTGTGGTCC 3503

B4_BF2_(AM282693) ------------------------------------------------------------ 3080

B12_BF2_(AB426147) GCGGTACCGCAGCGCGCCCGGCTCGGCATCCATGTGCCCACGGCACAGCTCTTGTGGTCC 3394

B12_BF2_(PacBio_C) GCGGTACCGCAGCGCGCCCGGCTCGGCATCCATGTGCCCACGGCACAGCTCTTGTGGTCC 3498

B12_BF2_(AL023516) GCGGTACCGCAGCGCGCCCGGCTCGGCATCCATGTGCCCACGGCACAGCTCTTGTGGTCC 3498

B14_BF2_(PacBio_WL) GCGGTACCGCAGCGCGCCCGGCTCGGCATCCATGTGCCCACGGCACAGCTCTTGTGGTCC 3515

B14_BF2_(AM282694) ------------------------------------------------------------ 3087

B15_BF2_(AB426149) GCGGTACCGCAGCGCGCCCGGCTCGGCATCCACGTGCCCACGGCACAGCTCTTGTGGTCC 3405

B15_BF2_(PacBio_15I) GCGGTACCGCAGCGCGCCCGGCTCGGCATCCACGTGCCCACGGCACAGCTCTTGTGGTCC 3524

B15_BF2_(AM282695) ------------------------------------------------------------ 3100

B19_BF2_(AB426151) GCGGTACCGCAGCGCGCCCGGCTCGGCATCCATGTGCCCACGGCACAGCTCTTGTGGTCC 3408

B19_BF2_(PacBio_P2a) GCGGTACCGCAGCGCGCCCGGCTCGGCATCCATGTGCCCACGGCACAGCTCTTGTGGTCC 3525

B19_BF2_(AM282696) ------------------------------------------------------------ 3097

B21_BF2_(AB426152) GCGGTACCGCAGCGCGCCCGGCTCGGCATCCATGTGCCCACGGCACAGCTCTTGTGGTCC 3414

B21_BF2_(PacBio_N) GCGGTACCGCAGCGCGCCCGGCTCGGCATCCATGTGCCCACGGCACAGCTCTTGTGGTCC 3518

B21_BF2_(AM282697) ------------------------------------------------------------ 3090

B21_BF2_(PacBio_0) GCGGTACCGCAGCGCGCCCGGCTCGGCATCCATGTGCCCACGGCACAGCTCTTGTGGTCC 3412

B21_BF2_(AM282700) ------------------------------------------------------------ 3090

B2_BF2_(AB426141) CTTGTCGCTGCCCCGGTGTCCCCACCTCCACCCTCAGTGTCCCCAACG**T**GCAGTGTCCCC 3454

B2_BF2_(PacBio_6sub1) CTTGTCGCTGCCCCGGTGTCCCCACCTCCACCCTCAGTGTCCCCAACG**T**GCAGTGTCCCC 3558

B2_BF2_(AM282692) ------------------------------------------------------------ 3072

B2_BF2_(PacBio_7sub2) CTTGTCGCTGCCCCGGTGTCCCCACCTCCACCCTCAGTGTCCCCAACG**T**GCAGTGTCCCC 3558

B2_BF2_(AM282698) ------------------------------------------------------------ 3072

B4_BF2_(AM282699) ------------------------------------------------------------ 3080

B4_BF2_(PacBio_C) CTTGTTGCTGCCCCGGTGTCCCCACCTCCACCCTCAGTGTCCCCAACGCGCAGTGTCCCC 3563

B4_BF2_(AM282693) ------------------------------------------------------------ 3080

B12_BF2_(AB426147) CTTGTCGCTGCCCCGGTGTCCCCACCTCCACCCTCAGTGTCCCCAACG**T**GCAGTGTCCCC 3454

B12_BF2_(PacBio_C) CTTGTCGCTGCCCCGGTGTCCCCACCTCCACCCTCAGTGTCCCCAACG**T**GCAGTGTCCCC 3558

B12_BF2_(AL023516) CTTGTCGCTGCCCCGGTGTCCCCACCTCCACCCTCAGTGTCCCCAACG**T**GCAGTGTCCCC 3558

B14_BF2_(PacBio_WL) CTTGTCGCTGCCCCGGTGTCCCCACCTCCACCCTCAGTGTCCCCAACGCCCAGTGTCCCC 3575

B14_BF2_(AM282694) ------------------------------------------------------------ 3087

B15_BF2_(AB426149) CTTGTCGCTGCCCCGGTGTCCCCACCTCCACCCTCAGTGTCCCCAACGCGCAGTGTCCCC 3465

B15_BF2_(PacBio_15I) CTTGTCGCTGCCCCGGTGTCCCCACCTCCACCCTCAGTGTCCCCAACGCGCAGTGTCCCC 3584

B15_BF2_(AM282695) ------------------------------------------------------------ 3100

B19_BF2_(AB426151) CTTGTCGCTGCCCCGGTGTCCCCACCTCCACCCTCAGTGTCCCCAACGCGCAGTGTCCCC 3468

B19_BF2_(PacBio_P2a) CTTGTCGCTGCCCCGGTGTCCCCACCTCCACCCTCAGTGTCCCCAACGCGCAGTGTCCCC 3585

B19_BF2_(AM282696) ------------------------------------------------------------ 3097

B21_BF2_(AB426152) CTTGTCGCTGCCCCGGTGTCCCCACCTCCACCCTCAGTGTCCCCAACGCGCAGTGTCCCC 3474

B21_BF2_(PacBio_N) CTTGTCGCTGCCCCGGTGTCCCCACCTCCACCCTCAGTGTCCCCAACGCGCAGTGTCCCC 3578

B21_BF2_(AM282697) ------------------------------------------------------------ 3090

B21_BF2_(PacBio_0) CTTGTCGCTGCCCCGGTGTCCCCACCTCCACCCTCAGTGTCCCCAACGCGCAGTGTCCCC 3472

B21_BF2_(AM282700) ------------------------------------------------------------ 3090

Fig. S2

B2_BF2_(AB426141) ATCCCACATCCCACCACCCCCTGCCCTCCCCATCCATAACTCCCGCTATCCCTCT----- 3509

B2_BF2_(PacBio_6sub1) ATCCCACATCCCACCACCCCCTGCCCTCCCCATCCATAACTCCCGCTATCCCTCT----- 3613

B2_BF2_(AM282692) ------------------------------------------------------------ 3072

B2_BF2_(PacBio_7sub2) ATCCCACATCCCACCACCCCCTGCCCTCCCCATCCATAACTCCCGCTATCCCTCT----- 3613

B2_BF2_(AM282698) ------------------------------------------------------------ 3072

B4_BF2_(AM282699) ------------------------------------------------------------ 3080

B4_BF2_(PacBio_C) ATCCCACATCCCACCACCCCCTGCCCTCCCCATCCATAACTCCTGCTATCCCTCT----- 3618

B4_BF2_(AM282693) ------------------------------------------------------------ 3080

B12_BF2_(AB426147) ATCCCACATCCCACCACCCCCTGCCCTCCCCATCCATAACTCCCGCTATCCCTCT----- 3509

B12_BF2_(PacBio_C) ATCCCACATCCCACCACCCCCTGCCCTCCCCATCCATAACTCCCGCTATCCCTCT----- 3613

B12_BF2_(AL023516) ATCCCACATCCCACCACCCCCTGCCCTCCCCATCCATAACTCCCGCTATCCCTCT----- 3613

B14_BF2_(PacBio_WL) ATCCCACATCTCACCACCCCCTGCCCTCCCCATCCATAACTCCCGCTATCCCTC**CCCCCC** 3635

B14_BF2_(AM282694) ------------------------------------------------------------ 3087

B15_BF2_(AB426149) ATCCCACATCCCACCACCCCCTGCCCTCCCCATCCATAACTCCCGCTATCCCTC**CCCCCC** 3525

B15_BF2_(PacBio_15I) ATCCCACATCCCACCACCCCCTGCCCTCCCCATCCATAACTCCCGCTATCCCTC**CCCCCC** 3644

B15_BF2_(AM282695) ------------------------------------------------------------ 3100

B19_BF2_(AB426151) ATCCCACATCCCACCACCCCCTGCCCTCCCCATCCATAACTCCCGCTATCCCTCT----- 3523

B19_BF2_(PacBio_P2a) ATCCCACATCCCACCACCCCCTGCCCTCCCCATCCATAACTCCCGCTATCCCTCT----- 3640

B19_BF2_(AM282696) ------------------------------------------------------------ 3097

B21_BF2_(AB426152) ATCCCACATCCCACCACCCCCTGCCCTCCCCATCCATAACTCCCGCTATCCCTCT----- 3529

B21_BF2_(PacBio_N) ATCCCACATCCCACCACCCCCTGCCCTCCCCATCCATAACTCCCGCTATCCCTCT----- 3633

B21_BF2_(AM282697) ------------------------------------------------------------ 3090

B21_BF2_(PacBio_0) ATCCCACATCCCACCACCCCCTGCCCTCCCCATCCATAACTCCCGCTATCCCTCT----- 3527

B21_BF2_(AM282700) ------------------------------------------------------------ 3090

B2_BF2_(AB426141) ---CCCTCTGTCCCCCTCCCCCGGTGCTCCCTGTTGTCCCCAGTCCCGCAGAAGGCTGCC 3566

B2_BF2_(PacBio_6sub1) ---CCCTCTGTCCCCCTCCCCCGGTGCTCCCTGTTGTCCCCAGTCCCGCAGAAGGCTGCC 3670

B2_BF2_(AM282692) ------------------------------------------------------------ 3072

B2_BF2_(PacBio_7sub2) ---CCCTCTGTCCCCCTCCCCCGGTGCTCCCTGTTGTCCCCAGTCCCGCAGAAGGCTGCC 3670

B2_BF2_(AM282698) ------------------------------------------------------------ 3072

B4_BF2_(AM282699) ------------------------------------------------------------ 3080

B4_BF2_(PacBio_C) ---CCCTCTGTCCCCCTCCCCTGGTGCTCCCTGTTGTCCCCAGTCCCGCAGAAGGCTGCT 3675

B4_BF2_(AM282693) ------------------------------------------------------------ 3080

B12_BF2_(AB426147) ---CCCTCTGTCCCCCTCCCCCGGTGCTCCCTGTTGTCCCCAGTCCCGCAGAAGGCTGCC 3566

B12_BF2_(PacBio_C) ---CCCTCTGTCCCCCTCCCCCGGTGCTCCCTGTTGTCCCCAGTCCCGCAGAAGGCTGCC 3670

B12_BF2_(AL023516) ---CCCTCTGTCCCCCTCCCCCGGTGCTCCCTGTTGTCCCCAGTCCCGCAGAAGGCTGCC 3670

B14_BF2_(PacBio_WL) **ACT**CCCTCTGTCCCCCTCCCCCGGTGCTCCCTGTTGTCCCCAGTCCCGCAGAAGGCTGCC 3695

B14_BF2_(AM282694) ------------------------------------------------------------ 3087

B15_BF2_(AB426149) **ACT**CCCTCTGTCCCCCTCCCCCGGTGCTCCCTGTTGTCCCCAGTCCCGCAGAAGGCTGCC 3585

B15_BF2_(PacBio_15I) **ACT**CCCTCTGTCCCCCTCCCCCGGTGCTCCCTGTTGTCCCCAGTCCCGCAGAAGGCTGCC 3704

B15_BF2_(AM282695) ------------------------------------------------------------ 3100

B19_BF2_(AB426151) ---CCCTCTGTCCCCCTCCCCCGGTGCTCCCTGTTGTCCCCAGTCCCGCAGAAGGCTGCC 3580

B19_BF2_(PacBio_P2a) ---CCCTCTGTCCCCCTCCCCCGGTGCTCCCTGTTGTCCCCAGTCCCGCAGAAGGCTGCC 3697

B19_BF2_(AM282696) ------------------------------------------------------------ 3097

B21_BF2_(AB426152) ---CCTTCTGTCCCCCTCCCCCGGTGCTCCCTGTTGTCCCCAGTCCCGCAGAAGGCTGCC 3586

B21_BF2_(PacBio_N) ---CCTTCTGTCCCCCTCCCCCGGTGCTCCCTGTTGTCCCCAGTCCCGCAGAAGGCTGCC 3690

B21_BF2_(AM282697) ------------------------------------------------------------ 3090

B21_BF2_(PacBio_0) ---CCTTCTGTCCCCCTCCCCCGGTGCTCCCTGTTGTCCCCAGTCCCGCAGAAGGCTGCC 3584

B21_BF2_(AM282700) ------------------------------------------------------------ 3090

B2_BF2_(AB426141) GGGGCGCAGCACCTCGTGGGGGGGTCCCT**G**CTGCC**A**CACCTCTCCCCC**G**TCCAACACCAC 3626

B2_BF2_(PacBio_6sub1) GGGGCGCAGCACCTCGTGGGGGGGTCCCT**G**CTGCC**A**CACCTCTCCCCC**G**TCCAACACCAC 3730

B2_BF2_(AM282692) ------------------------------------------------------------ 3072

B2_BF2_(PacBio_7sub2) GGGGCGCAGCACCTCGTGGGGGGGTCCCT**G**CTGCC**A**CACCTCTCCCCC**G**TCCAACACCAC 3730

B2_BF2_(AM282698) ------------------------------------------------------------ 3072

B4_BF2_(AM282699) ------------------------------------------------------------ 3080

B4_BF2_(PacBio_C) GGGGCACAGCACCTC**A**TGGGGGGGTCCCT**G**CTGCCGCACCTCTCCCCCCTCCAACACCAC 3735

B4_BF2_(AM282693) ------------------------------------------------------------ 3080

B12_BF2_(AB426147) GGGGCGCAGCACCTCGTGGGGGGGTCCCT**G**CTGCC**A**CACCTCTCCCCC**G**TCCAACACCAC 3626

B12_BF2_(PacBio_C) GGGGCGCAGCACCTCGTGGGGGGGTCCCT**G**CTGCC**A**CACCTCTCCCCC**G**TCCAACACCAC 3730

B12_BF2_(AL023516) GGGGCGCAGCACCTCGTGGGGGGGTCCCT**G**CTGCC**A**CACCTCTCCCCC**G**TCCAACACCAC 3730

B14_BF2_(PacBio_WL) GGGGCGCAGCACCTCGTGGGGGGGTCCCTCCTGCCGCACCTCTCCCCCCTCCA**G**CACCAC 3755

B14_BF2_(AM282694) ------------------------------------------------------------ 3087

B15_BF2_(AB426149) GGGGCGCAGCACCTCGTGGGGGGGTCCCTCCTGCCGCACCTCTCCCCCCTCCA**G**CACCAC 3645

B15_BF2_(PacBio_15I) GGGGCGCAGCACCTCGTGGGGGGGTCCCTCCTGCCGCACCTCTCCCCCCTCCA**G**CACCAC 3764

B15_BF2_(AM282695) ------------------------------------------------------------ 3100

B19_BF2_(AB426151) GGGGCGCAGCACCTCGTGGGGGGGTCCCTCCTGCCGCACCTCTCCCCCCTCCAACACCAC 3640

B19_BF2_(PacBio_P2a) GGGGCGCAGCACCTCGTGGGGGGGTCCCTCCTGCCGCACCTCTCCCCCCTCCAACACCAC 3757

B19_BF2_(AM282696) ------------------------------------------------------------ 3097

B21_BF2_(AB426152) GGGGCGCAGCACCTCGTGGGGGGGTCCCTCCTGCCGCA**T**CTCTCCCCCCTCCAACACCAC 3646

B21_BF2_(PacBio_N) GGGGCGCAGCACCTCGTGGGGGGGTCCCTCCTGCCGCA**T**CTCTCCCCCCTCCAACACCAC 3750

B21_BF2_(AM282697) ------------------------------------------------------------ 3090

B21_BF2_(PacBio_0) GGGGCGCAGCACCTCGTGGGGGGGTCCCTCCTGCCGCA**T**CTCTCCCCCCTCCAACACCAC 3644

B21_BF2_(AM282700) ------------------------------------------------------------ 3090

Fig. S2

B2_BF2_(AB426141) TACCCATTGT**A**CCAGGGCTGCCCGCCCCGT**C**ACCATCAGCACTGCAC**A**TCCTTACCCTCT 3686

B2_BF2_(PacBio_6sub1) TACCCATTGT**A**CCAGGGCTGCCCGCCCCGT**C**ACCATCAGCACTGCAC**A**TCCTTACCCTCT 3790

B2_BF2_(AM282692) ------------------------------------------------------------ 3072

B2_BF2_(PacBio_7sub2) TACCCATTGT**A**CCAGGGCTGCCCGCCCCGT**C**ACCATCAGCACTGCAC**A**TCCTTACCCTCT 3790

B2_BF2_(AM282698) ------------------------------------------------------------ 3072

B4_BF2_(AM282699) ------------------------------------------------------------ 3080

B4_BF2_(PacBio_C) TACCCAATGTGCCAGGGCTGCCCGCCCCGTTACCATCAGCGCTGCACGTCCTTACCCTCT 3795

B4_BF2_(AM282693) ------------------------------------------------------------ 3080

B12_BF2_(AB426147) TACCCATTGT**A**CCAGGGCTGCCCGCCCCGT**C**ACCATCAGCACTGCAC**A**TCCTTACCCTCT 3686

B12_BF2_(PacBio_C) TACCCATTGT**A**CCAGGGCTGCCCGCCCCGT**C**ACCATCAGCACTGCAC**A**TCCTTACCCTCT 3790

B12_BF2_(AL023516) TACCCATTGT**A**CCAGGGCTGCCCGCCCCGT**C**ACCATCAGCACTGCAC**A**TCCTTACCCTCT 3790

B14_BF2_(PacBio_WL) **C**ACCCATTGTGCCAGGGCTGCCCGCCCCGTTACCATCAGCACTGCACGCCCT**G**ACCCTCT 3815

B14_BF2_(AM282694) ------------------------------------------------------------ 3087

B15_BF2_(AB426149) **C**ACCCATTGTGCCAGGGCTGCCCGCCCCGTTACCATCAGCACTGCACGCCCT**G**ACCCTCT 3705

B15_BF2_(PacBio_15I) **C**ACCCATTGTGCCAGGGCTGCCCGCCCCGTTACCATCAGCACTGCACGCCCT**G**ACCCTCT 3824

B15_BF2_(AM282695) ------------------------------------------------------------ 3100

B19_BF2_(AB426151) TACCCATTGTGCCAGGGCTGCCCG**T**CCCGTTACCATCAGCACTGCACGCCCC**G**ACCCTCT 3700

B19_BF2_(PacBio_P2a) TACCCATTGTGCCAGGGCTGCCCG**T**CCCGTTACCATCAGCACTGCACGCCCC**G**ACCCTCT 3817

B19_BF2_(AM282696) ------------------------------------------------------------ 3097

B21_BF2_(AB426152) TACCCATTGTGCCAGGGCTGCCCGCCCCGT**C**ACCAT**A**AGCACTGCACGTCCTTACCCTCT 3706

B21_BF2_(PacBio_N) TACCCATTGTGCCAGGGCTGCCCGCCCCGT**C**ACCAT**A**AGCACTGCACGTCCTTACCCTCT 3810

B21_BF2_(AM282697) ------------------------------------------------------------ 3090

B21_BF2_(PacBio_0) TACCCATTGTGCCAGGGCTGCCCGCCCCGT**C**ACCAT**A**AGCACTGCACGTCCTTACCCTCT 3704

B21_BF2_(AM282700) ------------------------------------------------------------ 3090

B2_BF2_(AB426141) GGCTACGAGGATCCAACTTGTGGACAGGCTTCTGATGCCCTGTGTATCAGAACCATTCTG 3746

B2_BF2_(PacBio_6sub1) GGCTACGAGGATCCAACTTGTGGACAGGCTTCTGATGCCCTGTGTATCAGAACCATTCTG 3850

B2_BF2_(AM282692) ------------------------------------------------------------ 3072

B2_BF2_(PacBio_7sub2) GGCTACGAGGATCCAACTTGTGGACAGGCTTCTGATGCCCTGTGTATCAGAACCATTCTG 3850

B2_BF2_(AM282698) ------------------------------------------------------------ 3072

B4_BF2_(AM282699) ------------------------------------------------------------ 3080

B4_BF2_(PacBio_C) GGCTACGAGGATCCCACTTGTGGACAGGCTTCTGATGCCCTGTGTATCAGAACCATTCTG 3855

B4_BF2_(AM282693) ------------------------------------------------------------ 3080

B12_BF2_(AB426147) GGCTACGAGGATCCAACTTGTGGACAGGCTTCTGATGCCCTGTGTATCAGAACCATTCTG 3746

B12_BF2_(PacBio_C) GGCTACGAGGATCCAACTTGTGGACAGGCTTCTGATGCCCTGTGTATCAGAACCATTCTG 3850

B12_BF2_(AL023516) GGCTACGAGGATCCAACTTGTGGACAGGCTTCTGATGCCCTGTGTATCAGAACCATTCTG 3850

B14_BF2_(PacBio_WL) GGCTACGAGGATCC**C**ACTTGTGGACAGGCTTCTGATGCCCTGTGTATCAGAACCATTCTG 3875

B14_BF2_(AM282694) ------------------------------------------------------------ 3087

B15_BF2_(AB426149) GGCTACGAGGATCC**C**ACTTGTGGACAGGCTTCTGATGCCCTGTGTATCAGAACCATTCTG 3765

B15_BF2_(PacBio_15I) GGCTACGAGGATCC**C**ACTTGTGGACAGGCTTCTGATGCCCTGTGTATCAGAACCATTCTG 3884

B15_BF2_(AM282695) ------------------------------------------------------------ 3100

B19_BF2_(AB426151) GGCTAC**A**AGGATCCAACTTGTGGACAGGCTTCTGATGCCCTGTGTATCAGAACCATTCTG 3760

B19_BF2_(PacBio_P2a) GGCTAC**A**AGGATCCAACTTGTGGACAGGCTTCTGATGCCCTGTGTATCAGAACCATTCTG 3877

B19_BF2_(AM282696) ------------------------------------------------------------ 3097

B21_BF2_(AB426152) GGCTAC**A**AGGATCCAACTTGTGGACAGGCTTCTGATGCCCTGTGTAT**T**AGAACCATTCTG 3766

B21_BF2_(PacBio_N) GGCTAC**A**AGGATCCAACTTGTGGACAGGCTTCTGATGCCCTGTGTAT**T**AGAACCATTCTG 3870

B21_BF2_(AM282697) ------------------------------------------------------------ 3090

B21_BF2_(PacBio_0) GGCTAC**A**AGGATCCAACTTGTGGACAGGCTTCTGATGCCCTGTGTAT**T**AGAACCATTCTG 3764

B21_BF2_(AM282700) ------------------------------------------------------------ 3090

B2_BF2_(AB426141) ATGGTGGCCCTGCAGATCTGCTCTCAGCCTGCCCGCAGTCTGTTCTGTGCTGTGGTTTGG 3806

B2_BF2_(PacBio_6sub1) ATGGTGGCCCTGCAGATCTGCTCTCAGCCTGCCCGCAGTCTGTTCTGTGCTGTGGTTTGG 3910

B2_BF2_(AM282692) ------------------------------------------------------------ 3072

B2_BF2_(PacBio_7sub2) ATGGTGGCCCTGCAGATCTGCTCTCAGCCTGCCCGCAGTCTGTTCTGTGCTGTGGTTTGG 3910

B2_BF2_(AM282698) ------------------------------------------------------------ 3072

B4_BF2_(AM282699) ------------------------------------------------------------ 3080

B4_BF2_(PacBio_C) ATGGTGGCCCTGCAGATCTGCTCTCAGCCTGCCCACAGTCTGTTCTGTGCTGTGGTTTGG 3915

B4_BF2_(AM282693) ------------------------------------------------------------ 3080

B12_BF2_(AB426147) ATGGTGGCCCTGCAGATCTGCTCTCAGCCTGCCCGCAGTCTGTTCTGTGCTGTGGTTTGG 3806

B12_BF2_(PacBio_C) ATGGTGGCCCTGCAGATCTGCTCTCAGCCTGCCCGCAGTCTGTTCTGTGCTGTGGTTTGG 3910

B12_BF2_(AL023516) ATGGTGGCCCTGCAGATCTGCTCTCAGCCTGCCCGCAGTCTGTTCTGTGCTGTGGTTTGG---3873

B14_BF2_(PacBio_WL) **C**TGGTGGCCCTGCAGATCTGCTCTCAGCCTGCCCGCAGTCTGTTCTGTGCTGTGGTTTGG 3935

B14_BF2_(AM282694) ------------------------------------------------------------ 3087

B15_BF2_(AB426149) **C**TGGTGGCCCTGCAGATCTGCTCTCAGCCTGCCCGCAGTCTGTTCTGTGCTGTGGTTTGG 3825

B15_BF2_(PacBio_15I) **C**TGGTGGCCCTGCAGATCTGCTCTCAGCCTGCCCGCAGTCTGTTCTGTGCTGTGGTTTGG 3944

B15_BF2_(AM282695) ------------------------------------------------------------ 3100

B19_BF2_(AB426151) ATGGTGGCCCTGCAGCTCTGCTCTCAGCCTGCCCGCAGTCTGTTCTGTGCTGTGGTTTGG 3820

B19_BF2_(PacBio_P2a) ATGGTGGCCCTGCAGCTCTGCTCTCAGCCTGCCCGCAGTCTGTTCTGTGCTGTGGTTTGG 3937

B19_BF2_(AM282696) ------------------------------------------------------------ 3097

B21_BF2_(AB426152) ATGGTGGCCCTGCAGATCTGCTCTCAGCCTGCCCGCAGTCTGTTCTGTGCTGTGGTTTGG 3826

B21_BF2_(PacBio_N) ATGGTGGCCCTGCAGATCTGCTCTCAGCCTGCCCGCAGTCTGTTCTGTGCTGTGGTTTGG 3930

B21_BF2_(AM282697) ------------------------------------------------------------ 3090

B21_BF2_(PacBio_0) ATGGTGGCCCTGCAGATCTGCTCTCAGCCTGCCCGCAGTCTGTTCTGTGCTGTGGTTTGG 3824

B21_BF2_(AM282700) ------------------------------------------------------------ 3090

Fig. S2

B2_BF2_(AB426141) GTT-----GGGATGGG**T**GAGGGGGCC**G**TGTCCTGGCTCGGCTTCTCTGG**A**TGCAGGC**A**TG 3861

B2_BF2_(PacBio_6sub1) GTT-----GGGATGGG**T**GAGGGGGCC**G**TGTCCTGGCTCGGCTTCTCTGG**A**TGCAGGC**A**TG 3965

B2_BF2_(AM282692) ------------------------------------------------------------ 3072

B2_BF2_(PacBio_7sub2) GTT-----GGGATGGG**T**GAGGGGGCC**G**TGTCCTGGCTCGGCTTCTCTGG**A**TGCAGGC**A**TG 3965

B2_BF2_(AM282698) ------------------------------------------------------------ 3072

B4_BF2_(AM282699) ------------------------------------------------------------ 3080

B4_BF2_(PacBio_C) -----**GAT**GGGATGGGGGAGGGGGCCATGTCCTGGCTCGGCTTCTCTGGGTGCAGGCGTG 3970

B4_BF2_(AM282693) ------------------------------------------------------------ 3080

B12_BF2_(AB426147) GTT-----GGGATGGG**T**GAGGGGGCC**G**TGTCCTGGCTCGGCTTCTCTGG**A**TGCAGGC**A**TG 3861

B12_BF2_(PacBio_C) GTT-----GGGATGGG**T**GAGGGGGCC**G**TGTCCTGGCTCGGCTTCTCTGG**A**TGCAGGC**A**TG 3965

B12_BF2_(AL023516) GTT-----GGGATGGG**T**GAGGGGGCC**G**TGTCCTGGCTCGGCTTCTCTGG**A**TGCAGGC**A**TG 3965

B14_BF2_(PacBio_WL) GTT-----GGGATGGGGGAGGGGGCCATGTCCTGGCTCGGCTTCTCTGGGTGCAG**A**CGTG 3990

B14_BF2_(AM282694) ------------------------------------------------------------ 3087

B15_BF2_(AB426149) GTT-----GGGATGGGGGAGGGGGCCATGTCCTGGCTCGGCTTCTCTGGGTGCAG**A**CGTG 3880

B15_BF2_(PacBio_15I) GTT-----GGGATGGGGGAGGGGGCCATGTCCTGGCTCGGCTTCTCTGGGTGCAG**A**CGTG 3999

B15_BF2_(AM282695) ------------------------------------------------------------ 3100

B19_BF2_(AB426151) -----**GAT**GGGATGGGTGAGGGGGCCATGTCCTGGCTCGGCTTCTCTGGGTGCAG**A**CGTG 3875

B19_BF2_(PacBio_P2a) -----**GAT**GGGATGGGTGAGGGGGCCATGTCCTGGCTCGGCTTCTCTGGGTGCAG**A**CGTG 3992

B19_BF2_(AM282696) ------------------------------------------------------------ 3097

B21_BF2_(AB426152) GTT**GGGAT**GGGATGGGGGAGGGGGCCATGTCCTGGCTCGGCTTCTCTGGGTGCTGGCGTG 3886

B21_BF2_(PacBio_N) GTT**GGGAT**GGGATGGGGGAGGGGGCCATGTCCTGGCTCGGCTTCTCTGGGTGCTGGCGTG 3990

B21_BF2_(AM282697) ------------------------------------------------------------ 3090

B21_BF2_(PacBio_0) GTT**GGGAT**GGGATGGGGGAGGGGGCCATGTCCTGGCTCGGCTTCTCTGGGTGCTGGCGTG 3884

B21_BF2_(AM282700) ------------------------------------------------------------ 3090

B2_BF2_(AB426141) GACCTGCACTGAGTGCCCACCACCCTGCCCTCCTCCCTCCGGCTGCTGTTTGCCCTCCCC 3921

B2_BF2_(PacBio_6sub1) GACCTGCACTGAGTGCCCACCACCCTGCCCTCCTCCCTCCGGCTGCTGTTTGCCCTCCCC 4025

B2_BF2_(AM282692) ------------------------------------------------------------ 3072

B2_BF2_(PacBio_7sub2) GACCTGCACTGAGTGCCCACCACCCTGCCCTCCTCCCTCCGGCTGCTGTTTGCCCTCCCC 4025

B2_BF2_(AM282698) ------------------------------------------------------------ 3072

B4_BF2_(AM282699) ------------------------------------------------------------ 3080

B4_BF2_(PacBio_C) GACCTGCACTGAGTGCCCACCACCCTGCCCTCCTCCCTCCGGCTGCTGTTTGCCCTCCCC 4030

B4_BF2_(AM282693) ------------------------------------------------------------ 3080

B12_BF2_(AB426147) GACCTGCACTGAGTGCCCACCACCCTGCCCTCCTCCCTCCGGCTGCTGTTTGCCCTCCCC 3921

B12_BF2_(PacBio_C) GACCTGCACTGAGTGCCCACCACCCTGCCCTCCTCCCTCCGGCTGCTGTTTGCCCTCCCC 4025

B12_BF2_(AL023516) GACCTGCACTGAGTGCCCACCACCCTGCCCTCCTCCCTCCGGCTGCTGTTTGCCCTCCCC 4025

B14_BF2_(PacBio_WL) GACCTGCACTGAGTGCCCACCACCCTGCCCTCCTCCCTCC**A**GCTGCTGTTTGCCCTCCCC 4050

B14_BF2_(AM282694) ------------------------------------------------------------ 3087

B15_BF2_(AB426149) GACCTGCACTGAGTGCCCACCACCCTGCCCTCCTCCCTCC**A**GCTGCTGTTTGCCCTCCCC 3940

B15_BF2_(PacBio_15I) GACCTGCACTGAGTGCCCACCACCCTGCCCTCCTCCCTCC**A**GCTGCTGTTTGCCCTCCCC 4059

B15_BF2_(AM282695) ------------------------------------------------------------ 3100

B19_BF2_(AB426151) GACCTGCACTGAGTGCCCACCACCCTGCCCTCCTCCCTCCGGCTGCTGTTTGCCCTCCCC 3935

B19_BF2_(PacBio_P2a) GACCTGCACTGAGTGCCCACCACCCTGCCCTCCTCCCTCCGGCTGCTGTTTGCCCTCCCC 4052

B19_BF2_(AM282696) ------------------------------------------------------------ 3097

B21_BF2_(AB426152) GACCTGCACTGAGTGCCCACCACCCTGCCCTCCTCCCTCCGGCTGCTGTTTGCCCTCCC**T** 3946

B21_BF2_(PacBio_N) GACCTGCACTGAGTGCCCACCACCCTGCCCTCCTCCCTCCGGCTGCTGTTTGCCCTCCC**T** 4050

B21_BF2_(AM282697) ------------------------------------------------------------ 3090

B21_BF2_(PacBio_0) GACCTGCACTGAGTGCCCACCACCCTGCCCTCCTCCCTCCGGCTGCTGTTTGCCCTCCC**T** 3944

B21_BF2_(AM282700) ------------------------------------------------------------ 3090

B2_BF2_(AB426141) ATTCCCCCCATCCCACATCACCT-CCCCCCTGTGCCCCCACGCTGATGGTTGCTAGCTTC 3980

B2_BF2_(PacBio_6sub1) ATTCCCCCCATCCCACATCACCT-CCCCCCTGTGCCCCCACGCTGATGGTTGCTAGCTTC 4084

B2_BF2_(AM282692) ------------------------------------------------------------ 3072

B2_BF2_(PacBio_7sub2) ATTCCCCCCATCCCACATCACCT-CCCCCCTGTGCCCCCACGCTGATGGTTGCTAGCTTC 4084

B2_BF2_(AM282698) ------------------------------------------------------------ 3072

B4_BF2_(AM282699) ------------------------------------------------------------ 3080

B4_BF2_(PacBio_C) ATTCCCCCCATCCCAC**T**TCACCT**T**CCCCCCTGTGCCCCCACGCTGATGGTTGCTAGCTTC 4090

B4_BF2_(AM282693) ------------------------------------------------------------ 3080

B12_BF2_(AB426147) ATTCCCCCCATCCCACATCACCT-CCCCCCTGTGCCCCCACGCTGATGGTTGCTAGCTTC 3980

B12_BF2_(PacBio_C) ATTCCCCCCATCCCACATCACCT-CCCCCCTGTGCCCCCACGCTGATGGTTGCTAGCTTC 4084

B12_BF2_(AL023516) ATTCCCCCCATCCCACATCACCT-CCCCCCTGTGCCCCCACGCTGATGGTTGCTAGCTTC- 4084

B14_BF2_(PacBio_WL) ATTCCCCCCATCCCACATCACC**-**-CCCCCCTGTGCCCCCACGCTGATGGTTGCTAGCTTC 4108

B14_BF2_(AM282694) ------------------------------------------------------------ 3087

B15_BF2_(AB426149) ATTCCCCCCATCCCACATCACC**-**-**-**CCCCC**C**GTGCCCCCACGCTGATGGTTGCTAGCTTC 3997

B15_BF2_(PacBio_15I) ATTCCCCCCATCCCACATCACC**-**-**-**CCCCC**C**GTGCCCCCACGCTGATGGTTGCTAGCTTC 4116

B15_BF2_(AM282695) ------------------------------------------------------------ 3100

B19_BF2_(AB426151) GTTCCCCC-ATCCCACATCACCT-**-**CCCCCTGTGCCCCCACGCTGATGGTTGCTAGCTTC 3992

B19_BF2_(PacBio_P2a) GTTCCCCC-ATCCCACATCACCT-**-**CCCCCTGTGCCCCCACGCTGATGGTTGCTAGCTTC 4109

B19_BF2_(AM282696) ------------------------------------------------------------ 3097

B21_BF2_(AB426152) ATTCCCCCCATCCCACATCACCT**T**CCCCCCTGTGCCCCCAC**A**CTGATGGTTGCTAGCTTC 4006

B21_BF2_(PacBio_N) ATTCCCCCCATCCCACATCACCT**T**CCCCCCTGTGCCCCCAC**A**CTGATGGTTGCTAGCTTC 4110

B21_BF2_(AM282697) ------------------------------------------------------------ 3090

B21_BF2_(PacBio_0) ATTCCCCCCATCCCACATCACCT**T**CCCCCCTGTGCCCCCAC**A**CTGATGGTTGCTAGCTTC 4004

B21_BF2_(AM282700) ------------------------------------------------------------ 3090

Fig. S2

B2_BF2_(AB426141) ATGTCCTGGAAGTGCTGTCCAGGATGAACTCTGGCTTTTGTGATGCCATCCCTGTGTGCT 4040

B2_BF2_(PacBio_6sub1) ATGTCCTGGAAGTGCTGTCCAGGATGAACTCTGGCTTTTGTGATGCCATCCCTGTGTGCT 4144

B2_BF2_(AM282692) ------------------------------------------------------------ 3072

B2_BF2_(PacBio_7sub2) ATGTCCTGGAAGTGCTGTCCAGGATGAACTCTGGCTTTTGTGATGCCATCCCTGTGTGCT 4144

B2_BF2_(AM282698) ------------------------------------------------------------ 3072

B4_BF2_(AM282699) ------------------------------------------------------------ 3080

B4_BF2_(PacBio_C) ATGTCCTGGAAGTGCTGTCCAGGATGAACTCTGGCTTTTG**C**GATGCCATCCCTGTGTGCT 4150

B4_BF2_(AM282693) ------------------------------------------------------------ 3080

B12_BF2_(AB426147) ATGTCCTGGAAGTGCTGTCCAGGATGAACTCTGGCTTTTGTGATGCCATCCCTGTGTGCT 4040

B12_BF2_(PacBio_C) ATGTCCTGGAAGTGCTGTCCAGGATGAACTCTGGCTTTTGTGATGCCATCCCTGTGTGCT 4144

B12_BF2_(AL023516) ATGTCCTGGAAGTGCTGTCCAGGATGAACTCTGGCTTTTGTGATGCCATCCCTGTGTGCT 4144

B14_BF2_(PacBio_WL) ATGCCCTGGAAGTGCTGTCCAGGATGAACTCTGGCTTTTGTGATGCCATCCCTGTGTGCT 4168

B14_BF2_(AM282694) ------------------------------------------------------------ 3087

B15_BF2_(AB426149) ATGTCCTGGAAGTGCTGTCCAGGATGAACTCTGGCTTTTGTGATGCCATCCCTGTGTGCT 4057

B15_BF2_(PacBio_15I) ATGTCCTGGAAGTGCTGTCCAGGATGAACTCTGGCTTTTGTGATGCCATCCCTGTGTGCT 4176

B15_BF2_(AM282695) ------------------------------------------------------------ 3100

B19_BF2_(AB426151) GTGTCCTGCAAGTGCTGTCCAGGATGAACTCCGGGTTTTG**C**GATGCCATCCCTGTGTGCT 4052

B19_BF2_(PacBio_P2a) GTGTCCTGCAAGTGCTGTCCAGGATGAACTCCGGGTTTTG**C**GATGCCATCCCTGTGTGCT 4169

B19_BF2_(AM282696) ------------------------------------------------------------ 3097

B21_BF2_(AB426152) **G**TGTCCTGGAAGTGCTGTCCAGGATGAACTCTGGCTTTTG**C**GATGCCA**C**CCCTGTGTGCT 4066

B21_BF2_(PacBio_N) **G**TGTCCTGGAAGTGCTGTCCAGGATGAACTCTGGCTTTTG**C**GATGCCA**C**CCCTGTGTGCT 4170

B21_BF2_(AM282697) ------------------------------------------------------------ 3090

B21_BF2_(PacBio_0) **G**TGTCCTGGAAGTGCTGTCCAGGATGAACTCTGGCTTTTG**C**GATGCCA**C**CCCTGTGTGCT 4064

B21_BF2_(AM282700) ------------------------------------------------------------ 3090

B2_BF2_(AB426141) GGGATAGCCTCCGTGTTCCTCCCCCTCACCTCTCCCCACAGGTACATCTCTA**G**C**-**AAGAC 4099

B2_BF2_(PacBio_6sub1) GGGATAGCCTCCGTGTTCCTCCCCCTCACCTCTCCCCACAGGTACATCTCTA**G**C**-**AAGAC 4203

B2_BF2_(AM282692) ------------------------------------------------------------ 3072

B2_BF2_(PacBio_7sub2) GGGATAGCCTCCGTGTTCCTCCCCCTCACCTCTCCCCACAGGTACATCTCTA**G**C**-**AAGAC 4203

B2_BF2_(AM282698) ------------------------------------------------------------ 3072

B4_BF2_(AM282699) ------------------------------------------------------------ 3080

B4_BF2_(PacBio_C) GGGATAGCCTCCGTGTTCCTCCCCCTCACCTCTCCCCACAGGTACATCTCTACCCAAGAC 4210

B4_BF2_(AM282693) ------------------------------------------------------------ 3080

B12_BF2_(AB426147) GGGATAGCCTCCGTGTTCCTCCCCCTCACCTCTCCCCACAGGTACATCTCTA**G**C**-**AAGAC 4099

B12_BF2_(PacBio_C) GGGATAGCCTCCGTGTTCCTCCCCCTCACCTCTCCCCACAGGTACATCTCTA**G**C**-**AAGAC 4203

B12_BF2_(AL023516) GGGATAGCCTCCGTGTTCCTCCCCCTCACCTCTCCCCACAGGTACATCTCTA**G**C-AAGAC 4203

B14_BF2_(PacBio_WL) GGGATAGCCTCCGTGTTCCTCCCCCTCACCTCTCCCCACAGGTACATCTCTACCCAAGAC 4228

B14_BF2_(AM282694) ------------------------------------------------------------ 3087

B15_BF2_(AB426149) GGGATAGCCTCCGTGTTCCTCCCCCTCACCTCTCCCCACAGGTACATCTCTACCCAAGAC 4117

B15_BF2_(PacBio_15I) GGGATAGCCTCCGTGTTCCTCCCCCTCACCTCTCCCCACAGGTACATCTCTACCCAAGAC 4236

B15_BF2_(AM282695) ------------------------------------------------------------ 3100

B19_BF2_(AB426151) GGGATAGCCTCCGTGTTCCTCCCCCTCACCTCTCCCCACAGGTACATCTC**CG**CCCGAGA**T** 4112

B19_BF2_(PacBio_P2a) GGGATAGCCTCCGTGTTCCTCCCCCTCACCTCTCCCCACAGGTACATCTC**CG**CCCGAGA**T** 4229

B19_BF2_(AM282696) ------------------------------------------------------------ 3097

B21_BF2_(AB426152) GGGATAGC**A**TCCGTGTTCCTCCCCCTCACCTCTCCCCACAGGTACATCTCTACCCAAGAC 4126

B21_BF2_(PacBio_N) GGGATAGC**A**TCCGTGTTCCTCCCCCTCACCTCTCCCCACAGGTACATCTCTACCCAAGAC 4230

B21_BF2_(AM282697) ------------------------------------------------------------ 3090

B21_BF2_(PacBio_0) GGGATAGC**A**TCCGTGTTCCTCCCCCTCACCTCTCCCCACAGGTACATCTCTACCCAAGAC 4124

B21_BF2_(AM282700) ------------------------------------------------------------ 3090

B2_BF2_(AB426141) CACTTTTTCTTTCC-----TTTCCCTCCCACAGACTGGTCTGGTCTGGCCAGCTTTCCCT 4154

B2_BF2_(PacBio_6sub1) CACTTTTTCTTTCC-----TTTCCCTCCCACAGACTGGTCTGGTCTGGCCAGCTTTCCCT 4258

B2_BF2_(AM282692) ------------------------------------------------------------ 3072

B2_BF2_(PacBio_7sub2) CACTTTTTCTTTCC-----TTTCCCTCCCACAGACTGGTCTGGTCTGGCCAGCTTTCCCT 4258

B2_BF2_(AM282698) ------------------------------------------------------------ 3072

B4_BF2_(AM282699) ------------------------------------------------------------ 3080

B4_BF2_(PacBio_C) CACTTTTTCTTTCC-----TTTCCCTCCCACAGACTGGTCTGGTCTGGCCAGCTTTCCCT 4265

B4_BF2_(AM282693) ------------------------------------------------------------ 3080

B12_BF2_(AB426147) CACTTTTTCTTTCC-----TTTCCCTCCCACAGACTGGTCTGGTCTGGCCAGCTTTCCCT 4154

B12_BF2_(PacBio_C) CACTTTTTCTTTCC-----TTTCCCTCCCACAGACTGGTCTGGTCTGGCCAGCTTTCCCT 4258

B12_BF2_(AL023516) CACTTTTTCTTTCC-----TTTCCCTCCCACAGACTGGTCTGGTCTGGCCAGCTTTCCCT 4258

B14_BF2_(PacBio_WL) CACTTTTTCTTTCC-----TTTCCCTCCCACAGACTGGTCTGGTCTGGCCAGCTTTCCCT 4283

B14_BF2_(AM282694) ------------------------------------------------------------ 3087

B15_BF2_(AB426149) CACTTTTTC**-----**-----TTTCCCTCCCACAGACTGGTCTGGTCTGGCCAGCTTTCCCT 4167

B15_BF2_(PacBio_15I) CACTTTTTC**-----**-----TTTCCCTCCCACAGACTGGTCTGGTCTGGCCAGCTTTCCCT 4286

B15_BF2_(AM282695) ------------------------------------------------------------ 3100

B19_BF2_(AB426151) CACTT**C**TTCTTTCC**TTTCC**TTTCCCTCCCACAGACTGGTCTGGTCTGGCCAGCTTTCCCT 4172

B19_BF2_(PacBio_P2a) CACTT**C**TTCTTTCC**TTTCC**TTTCCCTCCCACAGACTGGTCTGGTCTGGCCAGCTTTCCCT 4289

B19_BF2_(AM282696) ------------------------------------------------------------ 3097

B21_BF2_(AB426152) CACTTTTTCTTTCC-----TTTCCCTCCCACAGACTGGTCTGGTCTGGCCAGCTTTCCCT 4181

B21_BF2_(PacBio_N) CACTTTTTCTTTCC-----TTTCCCTCCCACAGACTGGTCTGGTCTGGCCAGCTTTCCCT 4285

B21_BF2_(AM282697) ------------------------------------------------------------ 3090

B21_BF2_(PacBio_0) CACTTTTTCTTTCC-----TTTCCCTCCCACAGACTGGTCTGGTCTGGCCAGCTTTCCCT 4179

B21_BF2_(AM282700) ------------------------------------------------------------ 3090

Fig. S2

B2_BF2_(AB426141) TGCCC**G**GACA**T**GAGGCTGACAGTGACCTCAGTGAGCCCCATTTTGTCCATTCCTGATCAC 4214

B2_BF2_(PacBio_6sub1) TGCCC**G**GACA**T**GAGGCTGACAGTGACCTCAGTGAGCCCCATTTTGTCCATTCCTGATCAC 4318

B2_BF2_(AM282692) ------------------------------------------------------------ 3072

B2_BF2_(PacBio_7sub2) TGCCC**G**GACA**T**GAGGCTGACAGTGACCTCAGTGAGCCCCATTTTGTCCATTCCTGATCAC 4318

B2_BF2_(AM282698) ------------------------------------------------------------ 3072

B4_BF2_(AM282699) ------------------------------------------------------------ 3080

B4_BF2_(PacBio_C) TGCCCAGACACGAGGCTGACAGTGACCTCAGTGAGCCCCATTTTGTCCATTCCTGATCAC 4325

B4_BF2_(AM282693) ------------------------------------------------------------ 3080

B12_BF2_(AB426147) TGCCC**G**GACA**T**GAGGCTGACAGTGACCTCAGTGAGCCCCATTTTGTCCATTCCTGATCAC 4214

B12_BF2_(PacBio_C) TGCCC**G**GACA**T**GAGGCTGACAGTGACCTCAGTGAGCCCCATTTTGTCCATTCCTGATCAC 4318

B12_BF2_(AL023516) TGCCC**G**GACA**T**GAGGCTGACAGTGACCTCAGTGAGCCCCATTTTGTCCATTCCTGATCAC 4318

B14_BF2_(PacBio_WL) TGCCCAGACACGAGGCTGACAGTGACCTCAGTGAGCCCCATTTTGTCCATTCCTGATCAC 4343

B14_BF2_(AM282694) ------------------------------------------------------------ 3087

B15_BF2_(AB426149) TGCCCAGACACGAGGCTGACAGTGACCTCAGTGAGCCCCATTTTGTCCATTCCTGATCAC 4227

B15_BF2_(PacBio_15I) TGCCCAGACACGAGGCTGACAGTGACCTCAGTGAGCCCCATTTTGTCCATTCCTGATCAC 4346

B15_BF2_(AM282695) ------------------------------------------------------------ 3100

B19_BF2_(AB426151) TGCCC**G**GACA**T**GAGGCTGACAGTGACCTCAGTGAGCCCCATTTTGTCCATTCCTGATCAC 4232

B19_BF2_(PacBio_P2a) TGCCC**G**GACA**T**GAGGCTGACAGTGACCTCAGTGAGCCCCATTTTGTCCATTCCTGATCAC 4349

B19_BF2_(AM282696) ------------------------------------------------------------ 3097

B21_BF2_(AB426152) TGCCCAGACACGAGGCTGACAGTGACCTCAGTGAGCCCCATTTTGTCCATTCCTGATCAC 4241

B21_BF2_(PacBio_N) TGCCCAGACACGAGGCTGACAGTGACCTCAGTGAGCCCCATTTTGTCCATTCCTGATCAC 4345

B21_BF2_(AM282697) ------------------------------------------------------------ 3090

B21_BF2_(PacBio_0) TGCCCAGACACGAGGCTGACAGTGACCTCAGTGAGCCCCATTTTGTCCATTCCTGATCAC 4239

B21_BF2_(AM282700) ------------------------------------------------------------ 3090

B2_BF2_(AB426141) ACAGAACACTGCAACGGCTGACCAGAGGTGGGGGTCTCTTCTGGAGATACTCAAAGCCCA 4274

B2_BF2_(PacBio_6sub1) ACAGAACACTGCAACGGCTGACCAGAGGTGGGGGTCTCTTCTGGAGATACTCAAAGCCCA 4378

B2_BF2_(AM282692) ------------------------------------------------------------ 3072

B2_BF2_(PacBio_7sub2) ACAGAACACTGCAACGGCTGACCAGAGGTGGGGGTCTCTTCTGGAGATACTCAAAGCCCA 4378

B2_BF2_(AM282698) ------------------------------------------------------------ 3072

B4_BF2_(AM282699) ------------------------------------------------------------ 3080

B4_BF2_(PacBio_C) ACAGAACACTGCAACGGCTGACCAGAGGTGGGGGTCTCTTCTGGAGATACTCAAAGCCCA 4385

B4_BF2_(AM282693) ------------------------------------------------------------ 3080

B12_BF2_(AB426147) ACAGAACACTGCAACGGCTGACCAGAGGTGGGGGTCTCTTCTGGAGATACTCAAAGCCCA 4274

B12_BF2_(PacBio_C) ACAGAACACTGCAACGGCTGACCAGAGGTGGGGGTCTCTTCTGGAGATACTCAAAGCCCA 4378

B12_BF2_(AL023516) ACAGAACACTGCAACGGCTGACCAGAGGTGGGGGTCTCTTCTGGAGATACTCAAAGCCCA 4378

B14_BF2_(PacBio_WL) ACAGAACACTGCAACGGCTGACCAGAGGTGGGGGTCTCTTCTGGAGATACTCAAAGCCCA 4403

B14_BF2_(AM282694) ------------------------------------------------------------ 3087

B15_BF2_(AB426149) ACAGAACACTGCAACGGCTGACCAGAGGTGGGGGTCTCTTCTGGAGATACTCAAAGCCCA 4287

B15_BF2_(PacBio_15I) ACAGAACACTGCAACGGCTGACCAGAGGTGGGGGTCTCTTCTGGAGATACTCAAAGCCCA 4406

B15_BF2_(AM282695) ------------------------------------------------------------ 3100

B19_BF2_(AB426151) ACAGAACACTGCAACGGCTGACCAGAGGTGGGGGTCTCTTCTGGAGATACTCAAAGCCCA 4292

B19_BF2_(PacBio_P2a) ACAGAACACTGCAACGGCTGACCAGAGGTGGGGGTCTCTTCTGGAGATACTCAAAGCCCA 4409

B19_BF2_(AM282696) ------------------------------------------------------------ 3097

B21_BF2_(AB426152) ACAGAACACTGCAACGGCTGACCAGAGGTGGGGGTCTCTTCTGGAGATACTCAAAGCCCA 4301

B21_BF2_(PacBio_N) ACAGAACACTGCAACGGCTGACCAGAGGTGGGGGTCTCTTCTGGAGATACTCAAAGCCCA 4405

B21_BF2_(AM282697) ------------------------------------------------------------ 3090

B21_BF2_(PacBio_0) ACAGAACACTGCAACGGCTGACCAGAGGTGGGGGTCTCTTCTGGAGATACTCAAAGCCCA 4299

B21_BF2_(AM282700) ------------------------------------------------------------ 3090

B2_BF2_(AB426141) CCTGGTGTGACTGCTGTAGGGAAATGCTTGGGGAACACAGAAGTCCCTCCCACCCCCTGC 4334

B2_BF2_(PacBio_6sub1) CCTGGTGTGACTGCTGTAGGGAAATGCTTGGGGAACACAGAAGTCCCTCCCACCCCCTGC 4438

B2_BF2_(AM282692) ------------------------------------------------------------ 3072

B2_BF2_(PacBio_7sub2) CCTGGTGTGACTGCTGTAGGGAAATGCTTGGGGAACACAGAAGTCCCTCCCACCCCCTGC 4438

B2_BF2_(AM282698) ------------------------------------------------------------ 3072

B4_BF2_(AM282699) ------------------------------------------------------------ 3080

B4_BF2_(PacBio_C) CC**C**GGTGTGACTGCTGTATGGAAATGCTTGGGGAACACAGAAGTCCCTCCCACCCCC**C**GC 4445

B4_BF2_(AM282693) ------------------------------------------------------------ 3080

B12_BF2_(AB426147) CCTGGTGTGACTGCTGTAGGGAAATGCTTGGGGAACACAGAAGTCCCTCCCACCCCCTGC 4334

B12_BF2_(PacBio_C) CCTGGTGTGACTGCTGTAGGGAAATGCTTGGGGAACACAGAAGTCCCTCCCACCCCCTGC 4438

B12_BF2_(AL023516) CCTGGTGTGACTGCTGTAGGGAAATGCTTGGGGAACACAGAAGTCCCTCCCACCCCCTGC 4438

B14_BF2_(PacBio_WL) CCTGGTGTGACTGCTGTAGGGAAATGCTTGCGGAACACAGAAGTCCCTCCCACCCCCTGC 4463

B14_BF2_(AM282694) ------------------------------------------------------------ 3087

B15_BF2_(AB426149) CCTGGTGTGACTGCTGTAGGGAAATGCTTGCGGAACACAGAAGTCCCTCCCACCCCCTGC 4347

B15_BF2_(PacBio_15I) CCTGGTGTGACTGCTGTAGGGAAATGCTTGCGGAACACAGAAGTCCCTCCCACCCCCTGC 4466

B15_BF2_(AM282695) ------------------------------------------------------------ 3100

B19_BF2_(AB426151) CCTGGTGTGACTGCTGTAGGGAAATGCTTGGGGAACACAGAAGTCCCTCCCACCCCCTGC 4352

B19_BF2_(PacBio_P2a) CCTGGTGTGACTGCTGTAGGGAAATGCTTGGGGAACACAGAAGTCCCTCCCACCCCCTGC 4469

B19_BF2_(AM282696) ------------------------------------------------------------ 3097

B21_BF2_(AB426152) CCTGGTGTGACTGCTGTAGGGAAATGCTTGGGGAACACAGAAGTCCCTCCCACCCCCTGC 4361

B21_BF2_(PacBio_N) CCTGGTGTGACTGCTGTAGGGAAATGCTTGGGGAACACAGAAGTCCCTCCCACCCCCTGC 4465

B21_BF2_(AM282697) ------------------------------------------------------------ 3090

B21_BF2_(PacBio_0) CCTGGTGTGACTGCTGTAGGGAAATGCTTGGGGAACACAGAAGTCCCTCCCACCCCCTGC 4359

B21_BF2_(AM282700) ------------------------------------------------------------ 3090

Fig. S2

B2_BF2_(AB426141) AGTCCTGTGCCCACCCCAC**A**CTCCTTGCTTGTCACTCCATCACACACTGCTGCTCTCCCT 4394

B2_BF2_(PacBio_6sub1) AGTCCTGTGCCCACCCCAC**A**CTCCTTGCTTGTCACTCCATCACACACTGCTGCTCTCCCT 4498

B2_BF2_(AM282692) ------------------------------------------------------------ 3072

B2_BF2_(PacBio_7sub2) AGTCCTGTGCCCACCCCAC**A**CTCCTTGCTTGTCACTCCATCACACACTGCTGCTCTCCCT 4498

B2_BF2_(AM282698) ------------------------------------------------------------ 3072

B4_BF2_(AM282699) ------------------------------------------------------------ 3080

B4_BF2_(PacBio_C) AGTCCTGTGCCCACCCCACCCTCCTTGCTTGTCACTCCATCACACACTGCTGCTCTCCCT 4505

B4_BF2_(AM282693) ------------------------------------------------------------ 3080

B12_BF2_(AB426147) AGTCCTGTGCCCACCCCAC**A**CTCCTTGCTTGTCACTCCATCACACACTGCTGCTCTCCCT 4394

B12_BF2_(PacBio_C) AGTCCTGTGCCCACCCCAC**A**CTCCTTGCTTGTCACTCCATCACACACTGCTGCTCTCCCT 4498

B12_BF2_(AL023516) AGTCCTGTGCCCACCCCAC**A**CTCCTTGCTTGTCACTCCATCACACACTGCTGCTCTCCCT --4498

B14_BF2_(PacBio_WL) AGTCCTGTGCCCACCCCACCCTCCTTGCTTGTCACTCCATCACACACTGCTGCTCTCCCT 4523

B14_BF2_(AM282694) ------------------------------------------------------------ 3087

B15_BF2_(AB426149) AGTCCTGTGCCCACCCCACCCTCCTTGCTTGTCACTCCATCACACACTGCTGCTCTCCCT 4407

B15_BF2_(PacBio_15I) AGTCCTGTGCCCACCCCACCCTCCTTGCTTGTCACTCCATCACACACTGCTGCTCTCCCT 4526

B15_BF2_(AM282695) ------------------------------------------------------------ 3100

B19_BF2_(AB426151) AGTCCTGTGCCCACCCCACCCTCCTTGCTTGTCACTCCATCACACACTGCTGCTCTCCCT 4412

B19_BF2_(PacBio_P2a) AGTCCTGTGCCCACCCCACCCTCCTTGCTTGTCACTCCATCACACACTGCTGCTCTCCCT 4529

B19_BF2_(AM282696) ------------------------------------------------------------ 3097

B21_BF2_(AB426152) AGTCCTGTGCCCACCCCACCCTCCTTGCTTGTCACTCCATCACACACTGCTGCTCTCCCT 4421

B21_BF2_(PacBio_N) AGTCCTGTGCCCACCCCACCCTCCTTGCTTGTCACTCCATCACACACTGCTGCTCTCCCT 4525

B21_BF2_(AM282697) ------------------------------------------------------------ 3090

B21_BF2_(PacBio_0) AGTCCTGTGCCCACCCCACCCTCCTTGCTTGTCACTCCATCACACACTGCTGCTCTCCCT 4419

B21_BF2_(AM282700) ------------------------------------------------------------ 3090

B2_BF2_(AB426141) CTTCTCCC-AGCATTTGCCCTTGCACTCTCCTTGGTATCTGCATGGCTTTCATATGGAGA 4453

B2_BF2_(PacBio_6sub1) CTTCTCCC-AGCATTTGCCCTTGCACTCTCCTTGGTATCTGCATGGCTTTCATATGGAGA 4557

B2_BF2_(AM282692) ------------------------------------------------------------ 3072

B2_BF2_(PacBio_7sub2) CTTCTCCC-AGCATTTGCCCTTGCACTCTCCTTGGTATCTGCATGGCTTTCATATGGAGA 4557

B2_BF2_(AM282698) ------------------------------------------------------------ 3072

B4_BF2_(AM282699) ------------------------------------------------------------ 3080

B4_BF2_(PacBio_C) CTTCTCCC-AGCATTTGCCCTTGCACTCTCCTTGGTATCTGCATGGCTTTCATATGGAGA 4564

B4_BF2_(AM282693) ------------------------------------------------------------ 3080

B12_BF2_(AB426147) CTTCTCCC-AGCATTTGCCCTTGCACTCTCCTTGGTATCTGCATGGCTTTCATATGGAGA 4453

B12_BF2_(PacBio_C) CTTCTCCC-AGCATTTGCCCTTGCACTCTCCTTGGTATCTGCATGGCTTTCATATGGAGA 4557

B12_BF2_(AL023516) CTTCTCCC-AGCATTTGCCCTTGCACTCTCCTTGGTATCTGCATGGCTTTCATATGGAGA --4557

B14_BF2_(PacBio_WL) CTTCTCCC-AGCATTTGCCCTTGCACTCTCCTTGGTATCTGCATGGCTTTCATATGGAGA 4582

B14_BF2_(AM282694) ------------------------------------------------------------ 3087

B15_BF2_(AB426149) CTTCTCCC-AGCATTTGCCCTTGCACTCTCCTTGGTATCTGCATGGCTTTCATATGGAGA 4466

B15_BF2_(PacBio_15I) CTTCTCCC-AGCATTTGCCCTTGCACTCTCCTTGGTATCTGCATGGCTTTCATATGGAGA 4585

B15_BF2_(AM282695) ------------------------------------------------------------ 3100

B19_BF2_(AB426151) CTTCTCCC-AGCATTTGCCCTTGCACTCTCCCTGGTATCTGCATGGCTTTCATATGGAGA 4471

B19_BF2_(PacBio_P2a) CTTCTCCC-AGCATTTGCCCTTGCACTCTCCCTGGTATCTGCATGGCTTTCATATGGAGA 4588

B19_BF2_(AM282696) ------------------------------------------------------------ 3097

B21_BF2_(AB426152) CTTCTCCC**C**AGCATTTGCCCTTGCACTCTCCCTGGTATCTGCATGGCTTTCATATGGAGA 4481

B21_BF2_(PacBio_N) CTTCTCCC**C**AGCATTTGCCCTTGCACTCTCCCTGGTATCTGCATGGCTTTCATATGGAGA 4585

B21_BF2_(AM282697) ------------------------------------------------------------ 3090

B21_BF2_(PacBio_0) CTTCTCCC**C**AGCATTTGCCCTTGCACTCTCCCTGGTATCTGCATGGCTTTCATATGGAGA 4479

B21_BF2_(AM282700) ------------------------------------------------------------ 3090

B2_BF2_(AB426141) CATGGCTGGAAACTTCGTCCCACTT**C**GGGCTGAGCTGTGCCTC**T**CCTCCATAACCTACAG 4513

B2_BF2_(PacBio_6sub1) CATGGCTGGAAACTTCGTCCCACTT**C**GGGCTGAGCTGTGCCTC**T**CCTCCATAACCTACAG 4617

B2_BF2_(AM282692) ------------------------------------------------------------ 3072

B2_BF2_(PacBio_7sub2) CATGGCTGGAAACTTCGTCCCACTT**C**GGGCTGAGCTGTGCCTC**T**CCTCCATAACCTACAG 4617

B2_BF2_(AM282698) ------------------------------------------------------------ 3072

B4_BF2_(AM282699) ------------------------------------------------------------ 3080

B4_BF2_(PacBio_C) CATGGCTGGAAACTTCGTCCCACTT**C**GGGCTGAGCTGTGCCTCCCCTCCATAACCTACAG 4624

B4_BF2_(AM282693) ------------------------------------------------------------ 3080

B12_BF2_(AB426147) CATGGCTGGAAACTTCGTCCCACTT**C**GGGCTGAGCTGTGCCTCTCCTCCATAACCTACAG 4513

B12_BF2_(PacBio_C) CATGGCTGGAAACTTCGTCCCACTT**C**GGGCTGAGCTGTGCCTCTCCTCCATAACCTACAG 4617

B12_BF2_(AL023516) CATGGCTGGAAACTTCGTCCCACTT**C**GGGCTGAGCTGTGCCTCTCCTCCATAACCTACAG --4617

B14_BF2_(PacBio_WL) CATGGCTGGAAACTT**TT**TCCCACTTTGGGCTGAGCTGTGCCTCCCCTCCATAACCTACAG 4642

B14_BF2_(AM282694) ------------------------------------------------------------ 3087

B15_BF2_(AB426149) CATGGCTGGAAACTT**TT**TCCCACTTTGGGCTGAGCTGTGCCTCCCCTCCATAACCTACAG 4526

B15_BF2_(PacBio_15I) CATGGCTGGAAACTT**TT**TCCCACTTTGGGCTGAGCTGTGCCTCCCCTCCATAACCTACAG 4645

B15_BF2_(AM282695) ------------------------------------------------------------ 3100

B19_BF2_(AB426151) CATGGCTGGAAACTTCGTCCCA**T**TTTGGGCTGAGCTGTGCCTCCCCTCCAT**G**ACCTACAG 4531

B19_BF2_(PacBio_P2a) CATGGCTGGAAACTTCGTCCCA**T**TTTGGGCTGAGCTGTGCCTCCCCTCCAT**G**ACCTACAG 4648

B19_BF2_(AM282696) ------------------------------------------------------------ 3097

B21_BF2_(AB426152) CATGGCTGGAAACTTCGTCCCACTTTGGGCTGAGCTGTGCCTCCCCTCCATAACCTACAG 4541

B21_BF2_(PacBio_N) CATGGCTGGAAACTTCGTCCCACTTTGGGCTGAGCTGTGCCTCCCCTCCATAACCTACAG 4645

B21_BF2_(AM282697) ------------------------------------------------------------ 3090

B21_BF2_(PacBio_0) CATGGCTGGAAACTTCGTCCCACTTTGGGCTGAGCTGTGCCTCCCCTCCATAACCTACAG 4539

B21_BF2_(AM282700) ------------------------------------------------------------ 3090

Fig. S2

B2_BF2_(AB426141) TGCTGCTGCAACTGAGGGCAAACACTCAGCTTCCATAACCAATCATGCACTGAGGATGAG 4573

B2_BF2_(PacBio_6sub1) TGCTGCTGCAACTGAGGGCAAACACTCAGCTTCCATAACCAATCATGCACTGAGGATGAG 4677

B2_BF2_(AM282692) ------------------------------------------------------------ 3072

B2_BF2_(PacBio_7sub2) TGCTGCTGCAACTGAGGGCAAACACTCAGCTTCCATAACCAATCATGCACTGAGGATGAG 4677

B2_BF2_(AM282698) ------------------------------------------------------------ 3072

B4_BF2_(AM282699) ------------------------------------------------------------ 3080

B4_BF2_(PacBio_C) TGCTGCTGCAACTGAGGGCAAACACTCAGCTTCCATAACCAATCATGCACTGAGGATGAG 4684

B4_BF2_(AM282693) ------------------------------------------------------------ 3080

B12_BF2_(AB426147) TGCTGCTGCAACTGAGGGCAAACACTCAGCTTCCATAACCAATCATGCACTGAGGATGAG 4573

B12_BF2_(PacBio_C) TGCTGCTGCAACTGAGGGCAAACACTCAGCTTCCATAACCAATCATGCACTGAGGATGAG 4677

B12_BF2_(AL023516) TGCTGCTGCAACTGAGGGCAAACACTCAGCTTCCATAACCAATCATGCACTGAGGATGAG --4677

B14_BF2_(PacBio_WL) TGCTGCTGCAACTGAGGG**G**AA**G**CACTCAGCTTCC**----**CC**--------------------** 4678

B14_BF2_(AM282694) ------------------------------------------------------------ 3087

B15_BF2_(AB426149) TGCTGCTGCAACTGAGGG**G**AA**G**CACTCAGCTTCC**----**CC**--------------------** 4562

B15_BF2_(PacBio_15I) TGCTGCTGCAACTGAGGG**G**AA**G**CACTCAGCTTCC**----**CC**--------------------** 4681

B15_BF2_(AM282695) ------------------------------------------------------------ 3100

B19_BF2_(AB426151) TGCTGCTGCAACTGAGGGCAAACACTCAGCTTCCATAACCAATCATGCACTGAGGATGAG 4591

B19_BF2_(PacBio_P2a) TGCTGCTGCAACTGAGGGCAAACACTCAGCTTCCATAACCAATCATGCACTGAGGATGAG 4708

B19_BF2_(AM282696) ------------------------------------------------------------ 3097

B21_BF2_(AB426152) TGCTGCTGCAACTGAGGGCAAACACTCAGCTTCCATAACCAATCATGCACTGAGGATGAG 4601

B21_BF2_(PacBio_N) TGCTGCTGCAACTGAGGGCAAACACTCAGCTTCCATAACCAATCATGCACTGAGGATGAG 4705

B21_BF2_(AM282697) ------------------------------------------------------------ 3090

B21_BF2_(PacBio_0) TGCTGCTGCAACTGAGGGCAAACACTCAGCTTCCATAACCAATCATGCACTGAGGATGAG 4599

B21_BF2_(AM282700) ------------------------------------------------------------ 3090

**TAP2 PolyA Site**

B2_BF2_(AB426141) CTGCCCACCCCCCACATCCCAACCCACTCCTCTACAAAGCATCTCCACTTTATTCCACAC 4633

B2_BF2_(PacBio_6sub1) CTGCCCACCCCCCACATCCCAACCCACTCCTCTACAAAGCATCTCCACTTTATTCCACAC 4737

B2_BF2_(AM282692) ------------------------------------------------------------ 3072

B2_BF2_(PacBio_7sub2) CTGCCCACCCCCCACATCCCAACCCACTCCTCTACAAAGCATCTCCACTTTATTCCACAC 4737

B2_BF2_(AM282698) ------------------------------------------------------------ 3072

B4_BF2_(AM282699) ------------------------------------------------------------ 3080

B4_BF2_(PacBio_C) CTGCCCACCCCCCACATCCCAACCCACTCCTCTACAAAGCATCTCCACTTTATTCCACAC 4744

B4_BF2_(AM282693) ------------------------------------------------------------ 3080

B12_BF2_(AB426147) CTGCCCACCCCCCACATCCCAACCCACTCCTCTACAAAGCATCTCCACTTTATTCCACAC 4633

B12_BF2_(PacBio_C) CTGCCCACCCCCCACATCCCAACCCACTCCTCTACAAAGCATCTCCACTTTATTCCACAC 4737

B12_BF2_(AL023516) CTGCCCACCCCCCACATCCCAACCCACTCCTCTACAAAGCATCTCCACTTTATTCCACAC --4737

B14_BF2_(PacBio_WL) **-**T**---**CACCCCCCACATCCCAACCCACTC**T**TCTACAAAGCATCTCCACTTTATTCCACAC 4734

B14_BF2_(AM282694) ------------------------------------------------------------ 3087

B15_BF2_(AB426149) **-**T**---**CACCCCCCACATCCCAACCCACTC**T**TCTACAAAGCATCTCCACTTTATTCCACAC 4618

B15_BF2_(PacBio_15I) **-**T**---**CACCCCCCACATCCCAACCCACTC**T**TCTACAAAGCATCTCCACTTTATTCCACAC 4737

B15_BF2_(AM282695) -------1----------------------------------------------------- 3100

B19_BF2_(AB426151) CTGCCCACCCCCCACATCCCAACCCACTCCTCTACAAAGCATCTCCACTTTATTCCACAC 4651

B19_BF2_(PacBio_P2a) CTGCCCACCCCCCACATCCCAACCCACTCCTCTACAAAGCATCTCCACTTTATTCCACAC 4768

B19_BF2_(AM282696) ------------------------------------------------------------ 3097

B21_BF2_(AB426152) CTGCCCACCCCCCACATCCCAACCCACTCCTCTACAAAGCATCTCCACTTTATTCCACAC 4661

B21_BF2_(PacBio_N) CTGCCCACCCCCCACATCCCAACCCACTCCTCTACAAAGCATCTCCACTTTATTCCACAC 4765

B21_BF2_(AM282697) ------------------------------------------------------------ 3090

B21_BF2_(PacBio_0) CTGCCCACCCCCCACATCCCAACCCACTCCTCTACAAAGCATCTCCACTTTATTCCACAC 4659

B21_BF2_(AM282700) ------------------------------------------------------------ 3090

**TAP2 exon 9**

B2_BF2_(AB426141) AAATCCCAG**A**GCATCCCATCCTGCAGCTGGAAGCAGAGGCAG**T**CACTGCTCCCCATATCC 4693

B2_BF2_(PacBio_6sub1) AAATCCCAG**A**GCATCCCATCCTGCAGCTGGAAGCAGAGGCAG**T**CACTGCTCCCCATATCC 4797

B2_BF2_(AM282692) ------------------------------------------------------------ 3072

B2_BF2_(PacBio_7sub2) AAATCCCAG**A**GCATCCCATCCTGCAGCTGGAAGCAGAGGCAG**T**CACTGCTCCCCATATCC 4797

B2_BF2_(AM282698) ------------------------------------------------------------ 3072

B4_BF2_(AM282699) ---------------------exon -------------------------------------3080

B4_BF2_(PacBio_C) AAATCCCAGGGCATCCCATCCTGCAGCTGGAAGCAGAGGCAG**T**CACTGCTCCCCATATCC 4804

B4_BF2_(AM282693) ------------------------------------------------------------ 3080

B12_BF2_(AB426147) AAATCCCAG**A**GCATCCCATCCTGCAGCTGGAAGCAGAGGCAG**T**CACTGCTCCCCATATCC 4693

B12_BF2_(PacBio_C) AAATCCCAG**A**GCATCCCATCCTGCAGCTGGAAGCAGAGGCAG**T**CACTGCTCCCCATATCC 4797

B12_BF2_(AL023516) AAATCCCAG**A**GCATCCCATCCTGCAGCTGGAAGCAGAGGCAG**T**CACTGCTCCCCATATCC 4797

B14_BF2_(PacBio_WL) AAATCCCAGGGCATCCCATCCTGCAGCTGGAAGCAGAGGCAGCCACTGCTCCCCATATCC 4794

B14_BF2_(AM282694) ------------------------------------------------------------ 3087

B15_BF2_(AB426149) AAATCCCAGGGCATCCCATCCTGCAGCTGGAAGCAGAGGCAGCCACTGCTCCCCATATCC 4678

B15_BF2_(PacBio_15I) AAATCCCAGGGCATCCCATCCTGCAGCTGGAAGCAGAGGCAGCCACTGCTCCCCATATCC 4797

B15_BF2_(AM282695) ------------------------------------------------------------ 3100

B19_BF2_(AB426151) AAATCCCAGGGCATCCCATCCTGCAGCTGGAAGCAGAGGCAGCCACTGCTCCCCATATCC 4711

B19_BF2_(PacBio_P2a) AAATCCCAGGGCATCCCATCCTGCAGCTGGAAGCAGAGGCAGCCACTGCTCCCCATATCC 4828

B19_BF2_(AM282696) ------------------------------------------------------------ 3097

B21_BF2_(AB426152) AAATCCCAGGGCATCCCATCCTGCAGCTGGAAGCAGAGGCAGCCACTGCTCCCCATATCC 4721

B21_BF2_(PacBio_N) AAATCCCAGGGCATCCCATCCTGCAGCTGGAAGCAGAGGCAGCCACTGCTCCCCATATCC 4825

B21_BF2_(AM282697) ------------------------------------------------------------ 3090

B21_BF2_(PacBio_0) AAATCCCAGGGCATCCCATCCTGCAGCTGGAAGCAGAGGCAGCCACTGCTCCCCATATCC 4719

Fig. S2

B21_BF2_(AM282700) ------------------------------------------------------------ 3090

**TAP2 Stop**

B2_BF2_(AB426141) CATCGCATGCCACTCCAGCTGCTCCATGGTTCTCAGTGCTGTAGCAGCCGGCTGTAGGGT 4753

B2_BF2_(PacBio_6sub1) CATCGCATGCCACTCCAGCTGCTCCATGGTTCTCAGTGCTGTAGCAGCCGGCTGTAGGGT 4857

B2_BF2_(AM282692) ------------------------------------------------------------ 3072

B2_BF2_(PacBio_7sub2) CATCGCATGCCACTCCAGCTGCTCCATGGTTCTCAGTGCTGTAGCAGCCGGCTGTAGGGT 4857

B2_BF2_(AM282698) ------------------------------------------------------------ 3072

B4_BF2_(AM282699) ------------------------------------------------------------ 3080

B4_BF2_(PacBio_C) CATCCCATGCCACTCCAGCTGCTCCATGGTTCTCAGTGCTGTAGCAGCCGGCTGTAGGGT 4864

B4_BF2_(AM282693) ------------------------------------------------------------ 3080

B12_BF2_(AB426147) CATCGCATGCCACTCCAGCTGCTCCATGGTTCTCAGTGCTGTAGCAGCCGGCTGTAGGGT 4753

B12_BF2_(PacBio_C) CATCGCATGCCACTCCAGCTGCTCCATGGTTCTCAGTGCTGTAGCAGCCGGCTGTAGGGT 4857

B12_BF2_(AL023516) CATCGCATGCCACTCCAGCTGCTCCATGGTTCTCAGTGCTGTAGCAGCCGGCTGTAGGGT --4857

B14_BF2_(PacBio_WL) CATCGCATGCCACTCCAGCTGCTCCATGGTTCTCAGTGCTGTAGCAGCCGGCTGTAGGGT 4854

B14_BF2_(AM282694) ------------------------------------------------------------ 3087

B15_BF2_(AB426149) CATCGCATGCCACTCCAGCTGCTCCATGGTTCTCAGTGCTGTAGCAGCCGGCTGTAGGGT 4738

B15_BF2_(PacBio_15I) CATCGCATGCCACTCCAGCTGCTCCATGGTTCTCAGTGCTGTAGCAGCCGGCTGTAGGGT 4857

B15_BF2_(AM282695) ------------------------------------------------------------ 3100

B19_BF2_(AB426151) CATTGCATGCCACTCCAGCTGCTCCATGGTTCTCAGTGCTGTAGCAGCCGGCTGTAGGGT 4771

B19_BF2_(PacBio_P2a) CATTGCATGCCACTCCAGCTGCTCCATGGTTCTCAGTGCTGTAGCAGCCGGCTGTAGGGT 4888

B19_BF2_(AM282696) ------------------------------------------------------------ 3097

B21_BF2_(AB426152) CATTGCATGCCACTCCAGCTGCTCCATGGTTCTCAGTGCTGTAGCAGCCGGCTGTAGGGT 4781

B21_BF2_(PacBio_N) CATTGCATGCCACTCCAGCTGCTCCATGGTTCTCAGTGCTGTAGCAGCCGGCTGTAGGGT 4885

B21_BF2_(AM282697) ------------------------------------------------------------ 3090

B21_BF2_(PacBio_0) CATTGCATGCCACTCCAGCTGCTCCATGGTTCTCAGTGCTGTAGCAGCCGGCTGTAGGGT 4779

B21_BF2_(AM282700) ------------------------------------------------------------ 3090

B2_BF2_(AB426141) CCGCCGCGGGTCCTCAGCTCGGCGGGTGTCCCCATCTCAGCCACCGTGCCATGCTCCAGC 4813

B2_BF2_(PacBio_6sub1) CCGCCGCGGGTCCTCAGCTCGGCGGGTGTCCCCATCTCAGCCACCGTGCCATGCTCCAGC 4917

B2_BF2_(AM282692) ------------------------------------------------------------ 3072

B2_BF2_(PacBio_7sub2) CCGCCGCGGGTCCTCAGCTCGGCGGGTGTCCCCATCTCAGCCACCGTGCCATGCTCCAGC 4917

B2_BF2_(AM282698) ------------------------------------------------------------ 3072

B4_BF2_(AM282699) ------------------------------------------------------------ 3080

B4_BF2_(PacBio_C) CCGCCGCGGGTCCTCAGCTCGGCGGGTGTCCCCATCTCAGCCACCGTGCCATGCTCCAGC 4924

B4_BF2_(AM282693) ------------------------------------------------------------ 3080

B12_BF2_(AB426147) CCGCCGCGGGTCCTCAGCTCGGCGGGTGTCCCCATCTCAGCCACCGTGCCATGCTCCAGC 4813

B12_BF2_(PacBio_C) CCGCCGCGGGTCCTCAGCTCGGCGGGTGTCCCCATCTCAGCCACCGTGCCATGCTCCAGC 4917

B12_BF2_(AL023516) CCGCCGCGGGTCCTCAGCTCGGCGGGTGTCCCCATCTCAGCCACCGTGCCATGCTCCAGC --4917

B14_BF2_(PacBio_WL) CCGCCGCGGGTCCTCAGCTCGGCGGGTGTCCCCATCTCAGCCACCGTGCCATGCTCCAGC 4914

B14_BF2_(AM282694) ------------------------------------------------------------ 3087

B15_BF2_(AB426149) CCGCCGCGGGTCCTCAGCTCGGCGGGTGTCCCCATCTCAGCCACCGTGCCATGCTCCAGC 4798

B15_BF2_(PacBio_15I) CCGCCGCGGGTCCTCAGCTCGGCGGGTGTCCCCATCTCAGCCACCGTGCCATGCTCCAGC 4917

B15_BF2_(AM282695) ------------------------------------------------------------ 3100

B19_BF2_(AB426151) CCGCCGCGGGTCCTCAGCTCGGCGGGTGTCCCCATCTCAGCCACCGTGCCATGCTCCAGC 4831

B19_BF2_(PacBio_P2a) CCGCCGCGGGTCCTCAGCTCGGCGGGTGTCCCCATCTCAGCCACCGTGCCATGCTCCAGC 4948

B19_BF2_(AM282696) ------------------------------------------------------------ 3097

B21_BF2_(AB426152) CCGCCGCGGGTCCTCAGCTCGGCGGGTGTCCCCATCTCAGCCACCGTGCCATGCTCCAGC 4841

B21_BF2_(PacBio_N) CCGCCGCGGGTCCTCAGCTCGGCGGGTGTCCCCATCTCAGCCACCGTGCCATGCTCCAGC 4945

B21_BF2_(AM282697) ------------------------------------------------------------ 3090

B21_BF2_(PacBio_0) CCGCCGCGGGTCCTCAGCTCGGCGGGTGTCCCCATCTCAGCCACCGTGCCATGCTCCAGC 4839

B21_BF2_(AM282700) ------------------------------------------------------------ 3090

B2_BF2_(AB426141) ACCACAATGCGGTCTGCCTTCTCCAGCATCCGTGGTTGGTGGGTGATGAGCAGCACCGTC 4873

B2_BF2_(PacBio_6sub1) ACCACAATGCGGTCTGCCTTCTCCAGCATCCGTGGTTGGTGGGTGATGAGCAGCACCGTC 4977

B2_BF2_(AM282692) ------------------------------------------------------------ 3072

B2_BF2_(PacBio_7sub2) ACCACAATGCGGTCTGCCTTCTCCAGCATCCGTGGTTGGTGGGTGATGAGCAGCACCGTC 4977

B2_BF2_(AM282698) ------------------------------------------------------------ 3072

B4_BF2_(AM282699) ------------------------------------------------------------ 3080

B4_BF2_(PacBio_C) ACCACAACGCGGTCTGCCTTCTCCAGCATCCGTGGTTGGTGGGTGATGAGCAGCACCGTC 4984

B4_BF2_(AM282693) ------------------------------------------------------------ 3080

B12_BF2_(AB426147) ACCACAATGCGGTCTGCCTTCTCCAGCATCCGTGGTTGGTGGGTGATGAGCAGCACCGTC 4873

B12_BF2_(PacBio_C) ACCACAATGCGGTCTGCCTTCTCCAGCATCCGTGGTTGGTGGGTGATGAGCAGCACCGTC 4977

B12_BF2_(AL023516) ACCACAATGCGGTCTGCCTTCTCCAGCATCCGTGGTTGGTGGGTGATGAGCAGCACCGTC---4977

B14_BF2_(PacBio_WL) ACCACAATGCGGTCTGCCTTCTCCAGCATCCGTGGTTGGTGGGTGATGAGCAGCACCGTC 4974

B14_BF2_(AM282694) ------------------------------------------------------------ 3087

B15_BF2_(AB426149) ACCACAATGCGGTCTGCCTTCTCCAGCATCCGTGGTTGGTGGGTGATGAGCAGCACCGTC 4858

B15_BF2_(PacBio_15I) ACCACAATGCGGTCTGCCTTCTCCAGCATCCGTGGTTGGTGGGTGATGAGCAGCACCGTC 4977

B15_BF2_(AM282695) ------------------------------------------------------------ 3100

B19_BF2_(AB426151) ACCACAACGCGGTCTGCCTTCTCCAGCATCCGTGGTTGGTGGGTGATGAGCAGCACCGTC 4891

B19_BF2_(PacBio_P2a) ACCACAACGCGGTCTGCCTTCTCCAGCATCCGTGGTTGGTGGGTGATGAGCAGCACCGTC 5008

B19_BF2_(AM282696) ------------------------------------------------------------ 3097

B21_BF2_(AB426152) ACCACAACGCGGTCTGCCTTCTCCAGCATCCGTGGTTGGTGGGTGATGAGCAGCACCGTC 4901

B21_BF2_(PacBio_N) ACCACAACGCGGTCTGCCTTCTCCAGCATCCGTGGTTGGTGGGTGATGAGCAGCACCGTC 5005

B21_BF2_(AM282697) ------------------------------------------------------------ 3090

B21_BF2_(PacBio_0) ACCACAACGCGGTCTGCCTTCTCCAGCATCCGTGGTTGGTGGGTGATGAGCAGCACCGTC 4899

B21_BF2_(AM282700) ------------------------------------------------------------ 3090

Fig. S2

B2_BF2_(AB426141) CGGTCCCCTCC**G**TTCCTCACCCACTGCTG**T**AGCTGTAGGACACACAGCTGCCCATCAGCA 4933

B2_BF2_(PacBio_6sub1) CGGTCCCCTCC**G**TTCCTCACCCACTGCTG**T**AGCTGTAGGACACACAGCTGCCCATCAGCA 5037

B2_BF2_(AM282692) ------------------------------------------------------------ 3072

B2_BF2_(PacBio_7sub2) CGGTCCCCTCC**G**TTCCTCACCCACTGCTG**T**AGCTGTAGGACACACAGCTGCCCATCAGCA 5037

B2_BF2_(AM282698) ------------------------------------------------------------ 3072

B4_BF2_(AM282699) ------------------------------------------------------------ 3080

B4_BF2_(PacBio_C) CGGTCCCCTCC**G**TTCCTCACCCACTGCTG**T**AGCTGTAGGACACACAGCTGCCCATCAGCA 5044

B4_BF2_(AM282693) ------------------------------------------------------------ 3080

B12_BF2_(AB426147) CGGTCCCCTCC**G**TTCCTCACCCACTGCTG**T**AGCTGTAGGACACACAGCTGCCCATCAGCA 4933

B12_BF2_(PacBio_C) CGGTCCCCTCC**G**TTCCTCACCCACTGCTG**T**AGCTGTAGGACACACAGCTGCCCATCAGCA 5037

B12_BF2_(AL023516) CGGTCCCCTCC**G**TTCCTCACCCACTGCTG**T**AGCTGTAGGACACACAGCTGCCCATCAGCA --5037

B14_BF2_(PacBio_WL) CGGTCCCCTCCATTCCTCACCCACTGCTGCAGCTGTAGGACACACAGCTGCCCATCAGCA 5034

B14_BF2_(AM282694) ------------------------------------------------------------ 3087

B15_BF2_(AB426149) CGGTCCCCTCCATTCCTCACCCACTGCTGCAGCTGTAGGACACACAGCTGCCCATCAGCA 4918

B15_BF2_(PacBio_15I) CGGTCCCCTCCATTCCTCACCCACTGCTGCAGCTGTAGGACACACAGCTGCCCATCAGCA 5037

B15_BF2_(AM282695) ------------------------------------------------------------ 3100

B19_BF2_(AB426151) CGGTCCCCTCCATTCCTCACCCACTGCTGCAGCTGTAGGACACACAGCTGCCCATCAGCA 4951

B19_BF2_(PacBio_P2a) CGGTCCCCTCCATTCCTCACCCACTGCTGCAGCTGTAGGACACACAGCTGCCCATCAGCA 5068

B19_BF2_(AM282696) ------------------------------------------------------------ 3097

B21_BF2_(AB426152) CGGTCCCCTCCATTCCTCACCCACTGCTGCAGCTGTAGGACACACAGCTGCCCATCAGCA 4961

B21_BF2_(PacBio_N) CGGTCCCCTCCATTCCTCACCCACTGCTGCAGCTGTAGGACACACAGCTGCCCATCAGCA 5065

B21_BF2_(AM282697) ------------------------------------------------------------ 3090

B21_BF2_(PacBio_0) CGGTCCCCTCCATTCCTCACCCACTGCTGCAGCTGTAGGACACACAGCTGCCCATCAGCA 4959

B21_BF2_(AM282700) ------------------------------------------------------------ 3090

**TAP2 exon 8**

B2_BF2_(AB426141) TGCACTGCAGGGCCAGACCCTCCCCCC-ACCCACTGCTCAGTGCTCACCATTGCATC**A**CT 4992

B2_BF2_(PacBio_6sub1) TGCACTGCAGGGCCAGACCCTCCCCCC-ACCCACTGCTCAGTGCTCACCATTGCATC**A**CT 5096

B2_BF2_(AM282692) ------------------------------------------------------------ 3072

B2_BF2_(PacBio_7sub2) TGCACTGCAGGGCCAGACCCTCCCCCC-ACCCACTGCTCAGTGCTCACCATTGCATC**A**CT 5096

B2_BF2_(AM282698) ------------------------------------------------------------ 3072

B4_BF2_(AM282699) ------------------------------------------------------------ 3080

B4_BF2_(PacBio_C) TGCGCTGCAGGGCCAGACCTTCCCCCC-CCACACTGCTCAGTGCTCACCATTGCATC**A**CT 5103

B4_BF2_(AM282693) ------------------------------------------------------------ 3080

B12_BF2_(AB426147) TGCACTGCAGGGCCAGACCCTCCCCCC-ACCCACTGCTCAGTGCTCACCATTGCATC**A**CT 4992

B12_BF2_(PacBio_C) TGCACTGCAGGGCCAGACCCTCCCCCC-ACCCACTGCTCAGTGCTCACCATTGCATC**A**CT 5096

B12_BF2_(AL023516) TGCACTGCAGGGCCAGACCCTCCCCCC-ACCCACTGCTCAGTGCTCACCATTGCATC**A**CT 5096

B14_BF2_(PacBio_WL) TGCGCTGCAGGGCCAGACCCTCCCCCC**CC**CCCACTGCTCAGTGCTCACCATTGCATCGCT 5094

B14_BF2_(AM282694) ------------------------------------------------------------ 3087

B15_BF2_(AB426149) TGCGCTGCAGGGCCAGACCCTCCCCCC-**C**CCCACTGCTCAGTGCTCACCATTGCATCGCT 4977

B15_BF2_(PacBio_15I) TGCGCTGCAGGGCCAGACCCTCCCCCC-**C**CCCACTGCTCAGTGCTCACCATTGCATCGCT 5096

B15_BF2_(AM282695) ------------------------------------------------------------ 3100

B19_BF2_(AB426151) TGCACTGCAGGGCCAGACCCTCCCCCC-ACCCACTGCTCAGTGCTCACCATTGCATCGCT 5010

B19_BF2_(PacBio_P2a) TGCACTGCAGGGCCAGACCCTCCCCCC-ACCCACTGCTCAGTGCTCACCATTGCATCGCT 5127

B19_BF2_(AM282696) ------------------------------------------------------------ 3097

B21_BF2_(AB426152) TGCACTGCAGGGCCAGACCCTCCCCCC-ACCCACTGCTCAGTGCTCACCATTGCATCGCT 5020

B21_BF2_(PacBio_N) TGCACTGCAGGGCCAGACCCTCCCCCC-ACCCACTGCTCAGTGCTCACCATTGCATCGCT 5124

B21_BF2_(AM282697) ------------------------------------------------------------ 3090

B21_BF2_(PacBio_0) TGCACTGCAGGGCCAGACCCTCCCCCC-ACCCACTGCTCAGTGCTCACCATTGCATCGCT 5018

B21_BF2_(AM282700) ------------------------------------------------------------ 3090

B2_BF2_(AB426141) GTCCCCATCCAGAGC**A**CTGGTGGCTTCGTCGAGGATAAGGACGGTGGGA**T**GCCGCACCAA 5052

B2_BF2_(PacBio_6sub1) GTCCCCATCCAGAGC**A**CTGGTGGCTTCGTCGAGGATAAGGACGGTGGGA**T**GCCGCACCAA 5156

B2_BF2_(AM282692) ------------------------------------------------------------ 3072

B2_BF2_(PacBio_7sub2) GTCCCCATCCAGAGC**A**CTGGTGGCTTCGTCGAGGATAAGGACGGTGGGA**T**GCCGCACCAA 5156

B2_BF2_(AM282698) ------------------------------------------------------------ 3072

B4_BF2_(AM282699) ------------------------------------------------------------ 3080

B4_BF2_(PacBio_C) GTCCCCATCCAGAGCGCTGGTGGCTTCGTCGAGGATAAGGACGGTGGGACGCCGCACCAA 5163

B4_BF2_(AM282693) ------------------------------------------------------------ 3080

B12_BF2_(AB426147) GTCCCCATCCAGAGC**A**CTGGTGGCTTCGTCGAGGATAAGGACGGTGGGA**T**GCCGCACCAA 5052

B12_BF2_(PacBio_C) GTCCCCATCCAGAGC**A**CTGGTGGCTTCGTCGAGGATAAGGACGGTGGGA**T**GCCGCACCAA 5156

B12_BF2_(AL023516) GTCCCCATCCAGAGC**A**CTGGTGGCTTCGTCGAGGATAAGGACGGTGGGA**T**GCCGCACCAA 5156

B14_BF2_(PacBio_WL) GTCCCCATCCAGAGCGCTGGTGGCTTCGTCGAGGATAAGGACGGTGGGA**T**GCCGCACCAA 5154

B14_BF2_(AM282694) ------------------------------------------------------------ 3087

B15_BF2_(AB426149) GTCCCCATCCAGAGCGCTGGTGGCTTCGTCGAGGATAAGGACGGTGGGACGCCGCACCAA 5037

B15_BF2_(PacBio_15I) GTCCCCATCCAGAGCGCTGGTGGCTTCGTCGAGGATAAGGACGGTGGGACGCCGCACCAA 5156

B15_BF2_(AM282695) ------------------------------------------------------------ 3100

B19_BF2_(AB426151) GTCCCCATCCAGAGCGCTGGTGGCTTCGTCGAGGATAAGGACGGTGGGAGGCCGCACCAA 5070

B19_BF2_(PacBio_P2a) GTCCCCATCCAGAGCGCTGGTGGCTTCGTCGAGGATAAGGACGGTGGGACGCCGCACCAA 5187

B19_BF2_(AM282696) ------------------------------------------------------------ 3097

B21_BF2_(AB426152) GTCCCCATCCAGAGC**A**CTGGTGGCTTCGTCGAGGATAAGGACGGTGGGACGCCGCACCAA 5080

B21_BF2_(PacBio_N) GTCCCCATCCAGAGC**A**CTGGTGGCTTCGTCGAGGATAAGGACGGTGGGACGCCGCACCAA 5184

B21_BF2_(AM282697) ------------------------------------------------------------ 3090

B21_BF2_(PacBio_0) GTCCCCATCCAGAGC**A**CTGGTGGCTTCGTCGAGGATAAGGACGGTGGGACGCCGCACCAA 5078

B21_BF2_(AM282700) ------------------------------------------------------------ 3090

Fig. S2

B2_BF2_(AB426141) AGCGCGGGCGATGGCGATGCGCTGCTTCTGCCCCGCTGACAGCTGCCCCCCTCTCTCCCC 5112

B2_BF2_(PacBio_6sub1) AGCGCGGGCGATGGCGATGCGCTGCTTCTGCCCCGCTGACAGCTGCCCCCCTCTCTCCCC 5216

B2_BF2_(AM282692) ------------------------------------------------------------ 3072

B2_BF2_(PacBio_7sub2) AGCGCGGGCGATGGCGATGCGCTGCTTCTGCCCCGCTGACAGCTGCCCCCCTCTCTCCCC 5216

B2_BF2_(AM282698) ------------------------------------------------------------ 3072

B4_BF2_(AM282699) ------------------------------------------------------------ 3080

B4_BF2_(PacBio_C) AGCGCGGGCGATGGCGATGCGCTGCTTCTGCCCCGCTGACAGCTGCCCCCCTCTCTCCCC 5223

B4_BF2_(AM282693) ------------------------------------------------------------ 3080

B12_BF2_(AB426147) AGCGCGGGCGATGGCGATGCGCTGCTTCTGCCCCGCTGACAGCTGCCCCCCTCTCTCCCC 5112

B12_BF2_(PacBio_C) AGCGCGGGCGATGGCGATGCGCTGCTTCTGCCCCGCTGACAGCTGCCCCCCTCTCTCCCC 5216

B12_BF2_(AL023516) AGCGCGGGCGATGGCGATGCGCTGCTTCTGCCCCGCTGACAGCTGCCCCCCTCTCTCCCC --5216

B14_BF2_(PacBio_WL) AGCGCGGGCGATGGCGATGCGCTGCTTCTGCCCCGCTGACAGCTGCCCCCCTCTCTCCCC 5214

B14_BF2_(AM282694) ------------------------------------------------------------ 3087

B15_BF2_(AB426149) AGCGCGGGCGATGGCGATGCGCTGCTTCTGCCCCGCTGACAGCTGCCCCCCTCTCTCCCC 5097

B15_BF2_(PacBio_15I) AGCGCGGGCGATGGCGATGCGCTGCTTCTGCCCCGCTGACAGCTGCCCCCCTCTCTCCCC 5216

B15_BF2_(AM282695) ------------------------------------------------------------ 3100

B19_BF2_(AB426151) AGCGCGGGC**A**ATGGCGATGCGCTGCTTCTGCCCCGCTGACAGCTGCCCCCCTCTCTCCCC 5130

B19_BF2_(PacBio_P2a) AGCGCGGGC**A**ATGGCGATGCGCTGCTTCTGCCCCGCTGACAGCTGCCCCCCTCTCTCCCC 5247

B19_BF2_(AM282696) ------------------------------------------------------------ 3097

B21_BF2_(AB426152) AGCGCGGGC**A**ATGGCGATGCGCTGCTTCTGCCCCGCTGACAGCTGCCCCCCTCTCTCCCC 5140

B21_BF2_(PacBio_N) AGCGCGGGC**A**ATGGCGATGCGCTGCTTCTGCCCCGCTGACAGCTGCCCCCCTCTCTCCCC 5244

B21_BF2_(AM282697) ------------------------------------------------------------ 3090

B21_BF2_(PacBio_0) AGCGCGGGC**A**ATGGCGATGCGCTGCTTCTGCCCCGCTGACAGCTGCCCCCCTCTCTCCCC 5138

B21_BF2_(AM282700) ------------------------------------------------------------ 3090

B2_BF2_(AB426141) TACGTCTGCAGGATGAGGGTGGGGATGAGTGGGGTCAGACACCCGGGTCCCCCCCTGCTC 5172

B2_BF2_(PacBio_6sub1) TACGTCTGCAGGATGAGGGTGGGGATGAGTGGGGTCAGACACCCGGGTCCCCCCCTGCTC 5276

B2_BF2_(AM282692) ------------------------------------------------------------ 3072

B2_BF2_(PacBio_7sub2) TACGTCTGCAGGATGAGGGTGGGGATGAGTGGGGTCAGACACCCGGGTCCCCCCCTGCTC 5276

B2_BF2_(AM282698) ------------------------------------------------------------ 3072

B4_BF2_(AM282699) ------------------------------------------------------------ 3080

B4_BF2_(PacBio_C) TACGTCTGCAGGATGAGGGTGGGGATGAGTGGGGTCAGACACCCGGGTCCCCCCCTGCTC 5283

B4_BF2_(AM282693) ------------------------------------------------------------ 3080

B12_BF2_(AB426147) TACGTCTGCAGGATGAGGGTGGGGATGAGTGGGGTCAGACACCCGGGTCCCCCCCTGCTC 5172

B12_BF2_(PacBio_C) TACGTCTGCAGGATGAGGGTGGGGATGAGTGGGGTCAGACACCCGGGTCCCCCCCTGCTC 5276

B12_BF2_(AL023516) TACGTCTGCAGGATGAGGGTGGGGATGAGTGGGGTCAGACACCCGGGTCCCCCCCTGCTC --5276

B14_BF2_(PacBio_WL) TACGTCTGCAGGATGAGGGTGGGGATGAGTGGGGTCAGACACCCGGGTCCCCCCCTGCTC 5274

B14_BF2_(AM282694) ------------------------------------------------------------ 3087

B15_BF2_(AB426149) TACGTCTGCAGGATGAGGGTGGGGATGAGTGGGGTCAGACACCCGGGTCCCCCCCTGCTC 5157

B15_BF2_(PacBio_15I) TACGTCTGCAGGATGAGGGTGGGGATGAGTGGGGTCAGACACCCGGGTCCCCCCCTGCTC 5276

B15_BF2_(AM282695) ------------------------------------------------------------ 3100

B19_BF2_(AB426151) TACGTCTGCAGGATGAGGGTGGGGATGAGTGGGGTCAGACACCCGGGTCCCCCCCTGCTC 5190

B19_BF2_(PacBio_P2a) TACGTCTGCAGGATGAGGGTGGGGATGAGTGGGGTCAGACACCCGGGTCCCCCCCTGCTC 5307

B19_BF2_(AM282696) ------------------------------------------------------------ 3097

B21_BF2_(AB426152) TACGTCTGCAGGATGAGGGTGGGGATGAGTGGGGTCAGACACCCGGGTCCCCCCCTGCTC 5200

B21_BF2_(PacBio_N) TACGTCTGCAGGATGAGGGTGGGGATGAGTGGGGTCAGACACCCGGGTCCCCCCCTGCTC 5304

B21_BF2_(AM282697) ------------------------------------------------------------ 3090

B21_BF2_(PacBio_0) TACGTCTGCAGGATGAGGGTGGGGATGAGTGGGGTCAGACACCCGGGTCCCCCCCTGCTC 5198

B21_BF2_(AM282700) ------------------------------------------------------------ 3090

**TAP2 exon 7**

B2_BF2_(AB426141) CCCAGCACTCACCAGTGCCAAAGCCTTGCTCCAGTGCAGAGATGAAGCCCAAAGCACCCG 5232

B2_BF2_(PacBio_6sub1) CCCAGCACTCACCAGTGCCAAAGCCTTGCTCCAGTGCAGAGATGAAGCCCAAAGCACCCG 5336

B2_BF2_(AM282692) ------------------------------------------------------------ 3072

B2_BF2_(PacBio_7sub2) CCCAGCACTCACCAGTGCCAAAGCCTTGCTCCAGTGCAGAGATGAAGCCCAAAGCACCCG 5336

B2_BF2_(AM282698) ------------------------------------------------------------ 3072

B4_BF2_(AM282699) ------------------------------------------------------------ 3080

B4_BF2_(PacBio_C) CCCAGCACTCACCAGTGCCAAAGCCTTGCTCCAGTGCAGAGATGAAGCCCAAAGCACCCG 5343

B4_BF2_(AM282693) ------------------------------------------------------------ 3080

B12_BF2_(AB426147) CCCAGCACTCACCAGTGCCAAAGCCTTGCTCCAGTGCAGAGATGAAGCCCAAAGCACCCG 5232

B12_BF2_(PacBio_C) CCCAGCACTCACCAGTGCCAAAGCCTTGCTCCAGTGCAGAGATGAAGCCCAAAGCACCCG 5336

B12_BF2_(AL023516) CCCAGCACTCACCAGTGCCAAAGCCTTGCTCCAGTGCAGAGATGAAGCCCAAAGCACCCG 5336

B14_BF2_(PacBio_WL) CCCAGCACTCACCAGTGCCAAAGCCTTGCTCCAGTGCAGAGATGAAGCCCAAAGCACCCG 5334

B14_BF2_(AM282694) ------------------------------------------------------------ 3087

B15_BF2_(AB426149) CCCAGCACTCACCAGTGCCAAAGCCTTGCTCCAGTGCAGAGATGAAGCCCAAAGCACCCG 5217

B15_BF2_(PacBio_15I) CCCAGCACTCACCAGTGCCAAAGCCTTGCTCCAGTGCAGAGATGAAGCCCAAAGCACCCG 5336

B15_BF2_(AM282695) ------------------------------------------------------------ 3100

B19_BF2_(AB426151) CCCAGCACTCACCAGTGCCAAAGCCTTGCTCCAGTGCAGAGATGAAGCCCAAAGCACCCG 5250

B19_BF2_(PacBio_P2a) CCCAGCACTCACCAGTGCCAAAGCCTTGCTCCAGTGCAGAGATGAAGCCCAAAGCACCCG 5367

B19_BF2_(AM282696) ------------------------------------------------------------ 3097

B21_BF2_(AB426152) CCCAGCACTCACCAGTGCCAAAGCCTTGCTCCAGTGCAGAGATGAAGCCCAAAGCACC**T**G 5260

B21_BF2_(PacBio_N) CCCAGCACTCACCAGTGCCAAAGCCTTGCTCCAGTGCAGAGATGAAGCCCAAAGCACC**T**G 5364

B21_BF2_(AM282697) ------------------------------------------------------------ 3090

B21_BF2_(PacBio_0) CCCAGCACTCACCAGTGCCAAAGCCTTGCTCCAGTGCAGAGATGAAGCCCAAAGCACC**T**G 5258

B21_BF2_(AM282700) ------------------------------------------------------------ 3090

Fig. S2

B2_BF2_(AB426141) CAGCCCTTGCAGCTGCTATGATCTCCTCCTCTTCGCAGTCCTCCATCCCGTAGGCAATGT 5292

B2_BF2_(PacBio_6sub1) CAGCCCTTGCAGCTGCTATGATCTCCTCCTCTTCGCAGTCCTCCATCCCGTAGGCAATGT 5396

B2_BF2_(AM282692) ------------------------------------------------------------ 3072

B2_BF2_(PacBio_7sub2) CAGCCCTTGCAGCTGCTATGATCTCCTCCTCTTCGCAGTCCTCCATCCCGTAGGCAATGT 5396

B2_BF2_(AM282698) ------------------------------------------------------------ 3072

B4_BF2_(AM282699) ------------------------------------------------------------ 3080

B4_BF2_(PacBio_C) CAGCCCTTGCAGCTGCTATGATCTCCTCCTCTTCGCAGTCCTCCATCCCGTAGGCAATGT 5403

B4_BF2_(AM282693) ------------------------------------------------------------ 3080

B12_BF2_(AB426147) CAGCCCTTGCAGCTGCTATGATCTCCTCCTCTTCGCAGTCCTCCATCCCGTAGGCAATGT 5292

B12_BF2_(PacBio_C) CAGCCCTTGCAGCTGCTATGATCTCCTCCTCTTCGCAGTCCTCCATCCCGTAGGCAATGT 5396

B12_BF2_(AL023516) CAGCCCTTGCAGCTGCTATGATCTCCTCCTCTTCGCAGTCCTCCATCCCGTAGGCAATGT --5396

B14_BF2_(PacBio_WL) CAGCCCTTGCAGCTGCTAT**T**ATCTCCTCCTCTT**T**GCAGTCCTCCATCCCGTAGGCAATGT 5394

B14_BF2_(AM282694) ------------------------------------------------------------ 3087

B15_BF2_(AB426149) CAGCCCTTGCAGCTGCTATGATCTCCTCCTCTTCGCAGTCCTCCATCCCGTAGGCAATGT 5277

B15_BF2_(PacBio_15I) CAGCCCTTGCAGCTGCTATGATCTCCTCCTCTTCGCAGTCCTCCATCCCGTAGGCAATGT 5396

B15_BF2_(AM282695) ------------------------------------------------------------ 3100

B19_BF2_(AB426151) CAGCCCTTGCAGCTGCTAT**T**ATCTCCTCCTCTT**T**GCAGTCCTCCATCCCGTAGGCAATGT 5310

B19_BF2_(PacBio_P2a) CAGCCCTTGCAGCTGCTAT**T**ATCTCCTCCTCTT**T**GCAGTCCTCCATCCCGTAGGCAATGT 5427

B19_BF2_(AM282696) ------------------------------------------------------------ 3097

B21_BF2_(AB426152) CAGCCTTTGCAGCTGCTATGATCTCCTCCTCTTCGCAGTCCTCCATCCCGTAGGCAATGT 5320

B21_BF2_(PacBio_N) CAGCCTTTGCAGCTGCTATGATCTCCTCCTCTTCGCAGTCCTCCATCCCGTAGGCAATGT 5424

B21_BF2_(AM282697) ------------------------------------------------------------ 3090

B21_BF2_(PacBio_0) CAGCCTTTGCAGCTGCTATGATCTCCTCCTCTTCGCAGTCCTCCATCCCGTAGGCAATGT 5318

B21_BF2_(AM282700) ------------------------------------------------------------ 3090

B2_BF2_(AB426141) TATCCCGAATGGAGCCAGAGAAGAGCACGGGTTCCTGCCCCACCAGTGCCACCTGCCCCA 5352

B2_BF2_(PacBio_6sub1) TATCCCGAATGGAGCCAGAGAAGAGCACGGGTTCCTGCCCCACCAGTGCCACCTGCCCCA 5456

B2_BF2_(AM282692) ------------------------------------------------------------ 3072

B2_BF2_(PacBio_7sub2) TATCCCGAATGGAGCCAGAGAAGAGCACGGGTTCCTGCCCCACCAGTGCCACCTGCCCCA 5456

B2_BF2_(AM282698) ------------------------------------------------------------ 3072

B4_BF2_(AM282699) ------------------------------------------------------------ 3080

B4_BF2_(PacBio_C) TATCCCGAATGGAGCCAGAGAAGAGCACGGGTTCCTGCCCCACCAGTGCCACCTGCCCCA 5463

B4_BF2_(AM282693) ------------------------------------------------------------ 3080

B12_BF2_(AB426147) TATCCCGAATGGAGCCAGAGAAGAGCACGGGTTCCTGCCCCACCAGTGCCACCTGCCCCA 5352

B12_BF2_(PacBio_C) TATCCCGAATGGAGCCAGAGAAGAGCACGGGTTCCTGCCCCACCAGTGCCACCTGCCCCA 5456

B12_BF2_(AL023516) TATCCCGAATGGAGCCAGAGAAGAGCACGGGTTCCTGCCCCACCAGTGCCACCTGCCCCA 5456

B14_BF2_(PacBio_WL) TATCCCGAATGGAGCCAGAGAAGAGCACGGGTTCCTGCCCCACCAGTGCCACCTGCCCCA 5454

B14_BF2_(AM282694) ------------------------------------------------------------ 3087

B15_BF2_(AB426149) TATCCCGAATGGAGCCAGAGAAGAGCACGGGTTCCTGCCCCACCAGTGCCACCTGCCCCA 5337

B15_BF2_(PacBio_15I) TATCCCGAATGGAGCCAGAGAAGAGCACGGGTTCCTGCCCCACCAGTGCCACCTGCCCCA 5456

B15_BF2_(AM282695) ------------------------------------------------------------ 3100

B19_BF2_(AB426151) TATCCCGAATGGAGCCAGAGAAGAGCACGGGTTCCTGCCCCACCAGTGCCACCTGCCCCA 5370

B19_BF2_(PacBio_P2a) TATCCCGAATGGAGCCAGAGAAGAGCACGGGTTCCTGCCCCACCAGTGCCACCTGCCCCA 5487

B19_BF2_(AM282696) ------------------------------------------------------------ 3097

B21_BF2_(AB426152) TATCCCGAATGGAGCCAGAGAAGAGCACGGGTTCCTGCCCCACCAGTGCCACCTGCCCCA 5380

B21_BF2_(PacBio_N) TATCCCGAATGGAGCCAGAGAAGAGCACGGGTTCCTGCCCCACCAGTGCCACCTGCCCCA 5484

B21_BF2_(AM282697) ------------------------------------------------------------ 3090

B21_BF2_(PacBio_0) TATCCCGAATGGAGCCAGAGAAGAGCACGGGTTCCTGCCCCACCAGTGCCACCTGCCCCA 5378

B21_BF2_(AM282700) ------------------------------------------------------------ 3090

B2_BF2_(AB426141) GGGCAGAGGCTCAGCCCCAGCACTGCTCAGTGCAGCCACATCTCCCCCCACCCCCTCACC 5412

B2_BF2_(PacBio_6sub1) GGGCAGAGGCTCAGCCCCAGCACTGCTCAGTGCAGCCACATCTCCCCCCACCCCCTCACC 5516

B2_BF2_(AM282692) ------------------------------------------------------------ 3072

B2_BF2_(PacBio_7sub2) GGGCAGAGGCTCAGCCCCAGCACTGCTCAGTGCAGCCACATCTCCCCCCACCCCCTCACC 5516

B2_BF2_(AM282698) ------------------------------------------------------------ 3072

B4_BF2_(AM282699) ------------------------------------------------------------ 3080

B4_BF2_(PacBio_C) GGGCAGAGGCTCAGCCCCAGCACTGCTCAGTGCAGCCACATCTCCCCCCACCCCCTCACC 5523

B4_BF2_(AM282693) ------------------------------------------------------------ 3080

B12_BF2_(AB426147) GGGCAGAGGCTCAGCCCCAGCACTGCTCAGTGCAGCCACATCTCCCCCCACCCCCTCACC 5412

B12_BF2_(PacBio_C) GGGCAGAGGCTCAGCCCCAGCACTGCTCAGTGCAGCCACATCTCCCCCCACCCCCTCACC 5516

B12_BF2_(AL023516) GGGCAGAGGCTCAGCCCCAGCACTGCTCAGTGCAGCCACATCTCCCCCCACCCCCTCACC 5516

B14_BF2_(PacBio_WL) GGGCAGAGGCTCAGCCCCAGCACTGCTCAGTGCAGCCACATCTCCCCCCACCCCCTCACC 5514

B14_BF2_(AM282694) ------------------------------------------------------------ 3087

B15_BF2_(AB426149) GGGCAGAGGCTCAGCCCCAGCACTGCTCAGTGCAGCCACATCTCCCCCCACCCCCTCACC 5397

B15_BF2_(PacBio_15I) GGGCAGAGGCTCAGCCCCAGCACTGCTCAGTGCAGCCACATCTCCCCCCACCCCCTCACC 5516

B15_BF2_(AM282695) ------------------------------------------------------------ 3100

B19_BF2_(AB426151) GGGCAGAGGCTCAGCCCCAGCACTGCTCAGTGCAGCCACATCTCCCCCCACCCCCTCACC 5430

B19_BF2_(PacBio_P2a) GGGCAGAGGCTCAGCCCCAGCACTGCTCAGTGCAGCCACATCTCCCCCCACCCCCTCACC 5547

B19_BF2_(AM282696) ------------------------------------------------------------ 3097

B21_BF2_(AB426152) GGGCAGAGGCTCAGCCCCAGCACTGCTCAGTGCAGCCACATCTCCCCCCACCCCCTCACC 5440

B21_BF2_(PacBio_N) GGGCAGAGGCTCAGCCCCAGCACTGCTCAGTGCAGCCACATCTCCCCCCACCCCCTCACC 5544

B21_BF2_(AM282697) ------------------------------------------------------------ 3090

B21_BF2_(PacBio_0) GGGCAGAGGCTCAGCCCCAGCACTGCTCAGTGCAGCCACATCTCCCCCCACCCCCTCACC 5438

B21_BF2_(AM282700) ------------------------------------------------------------ 3090

Fig. S2

**TAP2 exon 6**

B2_BF2_(AB426141) TGGCGGTGCAGGTAGCGGTGCTCGTAGTCCCGCAGCGGCACCCCGTCCAGCAGCACTTCC 5472

B2_BF2_(PacBio_6sub1) TGGCGGTGCAGGTAGCGGTGCTCGTAGTCCCGCAGCGGCACCCCGTCCAGCAGCACTTCC 5576

B2_BF2_(AM282692) ------------------------------------------------------------ 3072

B2_BF2_(PacBio_7sub2) TGGCGGTGCAGGTAGCGGTGCTCGTAGTCCCGCAGCGGCACCCCGTCCAGCAGCACTTCC 5576

B2_BF2_(AM282698) ------------------------------------------------------------ 3072

B4_BF2_(AM282699) ------------------------------------------------------------ 3080

B4_BF2_(PacBio_C) TGGCGGTGCAGGTAGCGGTGCTCGTAGTCCC**T**CAGCGGCACCCCGTCCAGCAGCACTTCC 5583

B4_BF2_(AM282693) ------------------------------------------------------------ 3080

B12_BF2_(AB426147) TGGCGGTGCAGGTAGCGGTGCTCGTAGTCCCGCAGCGGCACCCCGTCCAGCAGCACTTCC 5472

B12_BF2_(PacBio_C) TGGCGGTGCAGGTAGCGGTGCTCGTAGTCCCGCAGCGGCACCCCGTCCAGCAGCACTTCC 5576

B12_BF2_(AL023516) TGGCGGTGCAGGTAGCGGTGCTCGTAGTCCCGCAGCGGCACCCCGTCCAGCAGCACTTCC --5576

B14_BF2_(PacBio_WL) TGGCGGTGCAGGTAGCGGTGCTCGTAGTCCCGCAGCGGCACCCCGTCCAGCAGCACTTCC 5574

B14_BF2_(AM282694) ------------------------------------------------------------ 3087

B15_BF2_(AB426149) TGGCGGTGCAGGTAGCGGTGCTCGTAGTCCCGCAGCGGCACCCCGTCCAGCAGCACTTCC 5457

B15_BF2_(PacBio_15I) TGGCGGTGCAGGTAGCGGTGCTCGTAGTCCCGCAGCGGCACCCCGTCCAGCAGCACTTCC 5576

B15_BF2_(AM282695) ------------------------------------------------------------ 3100

B19_BF2_(AB426151) TGGCGGTGCAGGTAGCGGTGCTCGTAGTCCCGCAGCGGCACCCCGTCCAGCAGCACTTCC 5490

B19_BF2_(PacBio_P2a) TGGCGGTGCAGGTAGCGGTGCTCGTAGTCCCGCAGCGGCACCCCGTCCAGCAGCACTTCC 5607

B19_BF2_(AM282696) ------------------------------------------------------------ 3097

B21_BF2_(AB426152) TGGCGGTGCAGGTAGCGGTGCTCGTAGTCCCGCAGCGGCACCCCGTCCAGCAGCACTTCC 5500

B21_BF2_(PacBio_N) TGGCGGTGCAGGTAGCGGTGCTCGTAGTCCCGCAGCGGCACCCCGTCCAGCAGCACTTCC 5604

B21_BF2_(AM282697) ------------------------------------------------------------ 3090

B21_BF2_(PacBio_0) TGGCGGTGCAGGTAGCGGTGCTCGTAGTCCCGCAGCGGCACCCCGTCCAGCAGCACTTCC 5498

B21_BF2_(AM282700) ------------------------------------------------------------ 3090

B2_BF2_(AB426141) CCGGCCCCAGGTTCATAGAATCTCTCCAGCAGTGCCACGCAGGTGCTCTTCCCGCTGCCA 5532

B2_BF2_(PacBio_6sub1) CCGGCCCCAGGTTCATAGAATCTCTCCAGCAGTGCCACGCAGGTGCTCTTCCCGCTGCCA 5636

B2_BF2_(AM282692) ------------------------------------------------------------ 3072

B2_BF2_(PacBio_7sub2) CCGGCCCCAGGTTCATAGAATCTCTCCAGCAGTGCCACGCAGGTGCTCTTCCCGCTGCCA 5636

B2_BF2_(AM282698) ------------------------------------------------------------ 3072

B4_BF2_(AM282699) ------------------------------------------------------------ 3080

B4_BF2_(PacBio_C) CCGGCCCCAGGTTCATAGAATCTCTCCAGCAGTGCCACGCAGGTGCTCTTCCCGCTGCCA 5643

B4_BF2_(AM282693) ------------------------------------------------------------ 3080

B12_BF2_(AB426147) CCGGCCCCAGGTTCATAGAATCTCTCCAGCAGTGCCACGCAGGTGCTCTTCCCGCTGCCA 5532

B12_BF2_(PacBio_C) CCGGCCCCAGGTTCATAGAATCTCTCCAGCAGTGCCACGCAGGTGCTCTTCCCGCTGCCA 5636

B12_BF2_(AL023516) CCGGCCCCAGGTTCATAGAATCTCTCCAGCAGTGCCACGCAGGTGCTCTTCCCGCTGCCA 5636

B14_BF2_(PacBio_WL) CCGGCCCCAGGTTCATAGAATCTCTCCAGCAGTGCCACGCAGGTGCTCTTCCCGCTGCCA 5634

B14_BF2_(AM282694) ------------------------------------------------------------ 3087

B15_BF2_(AB426149) CC**A**GCCCCAGGTTCATAGAATCTCTCCAGCAGTGCCACGCAGGTGCTCTTCCCGCTGCCA 5517

B15_BF2_(PacBio_15I) CC**A**GCCCCAGGTTCATAGAATCTCTCCAGCAGTGCCACGCAGGTGCTCTTCCCGCTGCCA 5636

B15_BF2_(AM282695) ------------------------------------------------------------ 3100

B19_BF2_(AB426151) CCGGCCCCAGGTTCATAGAATCTCTCCAGCAGTGCCACGCAGGTGCTCTTCCCGCTGCCA 5550

B19_BF2_(PacBio_P2a) CCGGCCCCAGGTTCATAGAATCTCTCCAGCAGTGCCACGCAGGTGCTCTTCCCGCTGCCA 5667

B19_BF2_(AM282696) ------------------------------------------------------------ 3097

B21_BF2_(AB426152) CC**A**GCCCCAGGTTCATAGAATCTCTCCAGCAGTGCCACGCAGGTGCTCTTCCCGCTGCCA 5560

B21_BF2_(PacBio_N) CC**A**GCCCCAGGTTCATAGAATCTCTCCAGCAGTGCCACGCAGGTGCTCTTCCCGCTGCCA 5664

B21_BF2_(AM282697) ------------------------------------------------------------ 3090

B21_BF2_(PacBio_0) CC**A**GCCCCAGGTTCATAGAATCTCTCCAGCAGTGCCACGCAGGTGCTCTTCCCGCTGCCA 5558

B21_BF2_(AM282700) ------------------------------------------------------------ 3090

B2_BF2_(AB426141) TTCAGCCCCGCCAACGCCGTCACCTCACCGGGGCGCAGCTCGAAGGTGACATCTTGCAGG 5592

B2_BF2_(PacBio_6sub1) TTCAGCCCCGCCAACGCCGTCACCTCACCGGGGCGCAGCTCGAAGGTGACATCTTGCAGG 5696

B2_BF2_(AM282692) ------------------------------------------------------------ 3072

B2_BF2_(PacBio_7sub2) TTCAGCCCCGCCAACGCCGTCACCTCACCGGGGCGCAGCTCGAAGGTGACATCTTGCAGG 5696

B2_BF2_(AM282698) ------------------------------------------------------------ 3072

B4_BF2_(AM282699) ------------------------------------------------------------ 3080

B4_BF2_(PacBio_C) TTCAGCCCCGCCAACGCCGTCACCTCAC**T**GGGGCGCAGCTCGAAGGTGACATCTTGCAGG 5703

B4_BF2_(AM282693) ------------------------------------------------------------ 3080

B12_BF2_(AB426147) TTCAGCCCCGCCAACGCCGTCACCTCACCGGGGCGCAGCTCGAAGGTGACATCTTGCAGG 5592

B12_BF2_(PacBio_C) TTCAGCCCCGCCAACGCCGTCACCTCACCGGGGCGCAGCTCGAAGGTGACATCTTGCAGG 5696

B12_BF2_(AL023516) TTCAGCCCCGCCAACGCCGTCACCTCACCGGGGCGCAGCTCGAAGGTGACATCTTGCAGG --5696

B14_BF2_(PacBio_WL) TTCAGCCCCGCCAA**T**GCCGTCACCTCACCGGGGCGCAGCTCGAAGGTGACATCTTGCAGG 5694

B14_BF2_(AM282694) ------------------------------------------------------------ 3087

B15_BF2_(AB426149) TTCAGCCCCGCCAA**T**GCCGTCACCTCACCGGGGCGCAGCTCGAAGGTGACATCTTGCAGG 5577

B15_BF2_(PacBio_15I) TTCAGCCCCGCCAA**T**GCCGTCACCTCACCGGGGCGCAGCTCGAAGGTGACATCTTGCAGG 5696

B15_BF2_(AM282695) ------------------------------------------------------------ 3100

B19_BF2_(AB426151) TTCAGCCCCGCCAACGCCGTCACCTCACCGGGGCGCAGCTCGAAGGTGACATCTTGCAGG 5610

B19_BF2_(PacBio_P2a) TTCAGCCCCGCCAACGCCGTCACCTCACCGGGGCGCAGCTCGAAGGTGACATCTTGCAGG 5727

B19_BF2_(AM282696) ------------------------------------------------------------ 3097

B21_BF2_(AB426152) TTCAGCCCCGCCAACGCCGTCACCTCACCGGGGCGCAGCTCGAAGGTGACATCTTGCAGG 5620

B21_BF2_(PacBio_N) TTCAGCCCCGCCAACGCCGTCACCTCACCGGGGCGCAGCTCGAAGGTGACATCTTGCAGG 5724

B21_BF2_(AM282697) ------------------------------------------------------------ 3090

B21_BF2_(PacBio_0) TTCAGCCCCGCCAACGCCGTCACCTCACCGGGGCGCAGCTCGAAGGTGACATCTTGCAGG 5618

B21_BF2_(AM282700) ------------------------------------------------------------ 3090

Fig. S2

**-----BF2 F Primer----**

B2_BF2_(AB426141) ACGAGGCGCTCAGGGCGAGTGGGATAGGCGAAGGACACCCGATGGAA 5652

B2_BF2_(PacBio_6sub1) ACGAGGCGCTCAGGGCGAGTGGGATAGGCGAAGGACACCCGATGGAA------------- 5756

B2_BF2_(AM282692) ------------------------------------------------------------ 3072

B2_BF2_(PacBio_7sub2) ACGAGGCGCTCAGGGCGAGTGGGATAGGCGAAGGACACCCGATGGAA 5756

B2_BF2_(AM282698) ------------------------------------------------------------ 3072

B4_BF2_(AM282699) ------------------------------------------------------------ 3080

B4_BF2_(PacBio_C) ACGAGGCGCTCAGGGCGAGTGGGATAGGCGAAGGACACCCGATGGAA------------- 5763

B4_BF2_(AM282693) ------------------------------------------------------------ 3080

B12_BF2_(AB426147) ACGAGGCGCTCAGGGCGAGTGGGATAGGCGAAGGACACCCGATGGAA 5652

B12_BF2_(PacBio_C) ACGAGGCGCTCAGGGCGAGTGGGATAGGCGAAGGACACCCGATGGAA------------- 5756

B12_BF2_(AL023516) ACGAGGCGCTCAGGGCGAGTGGGATAGGCGAAGGACACCCGATGGAA 5767

B14_BF2_(PacBio_WL) ACGAGGCGCTCAGGGCGAGTGGGATAGGCGAAGGACACCCGATGGAA------------- 5754

B14_BF2_(AM282694) ------------------------------------------------------------ 3087

B15_BF2_(AB426149) ACAAGGCGCTCAGGGCGAGTGGGATAGGCGAAGGACACCCGATGGAA 5637

B15_BF2_(PacBio_15I) ACAAGGCGCTCAGGGCGAGTGGGATAGGCGAAGGACACCCGATGGAA------------- 5756

B15_BF2_(AM282695) ------------------------------------------------------------ 3100

B19_BF2_(AB426151) ACGAGGCGCTCAGGGCAAGTGGGATAGGCGAAGGACACCCGATGGAA 5670

B19_BF2_(PacBio_P2a) ACGAGGCGCTCAGGGCAAGTGGGATAGGCGAAGGACACCCGATGGAA------------- 5787

B19_BF2_(AM282696) ------------------------------------------------------------ 3097

B21_BF2_(AB426152) ACGAGGCGCTCAGGGCGAGTGGGATAGGCGAAGGACACCCGATGGAA ------------ 5668

B21_BF2_(PacBio_N) ACGAGGCGCTCAGGGCGAGTGGGATAGGCGAAGGACACCCGATGGAA------------- 5784

B21_BF2_(AM282697) ------------------------------------------------------------ 3090

B21_BF2_(PacBio_0) ACGAGGCGCTCAGGGCGAGTGGGATAGGCGAAGGACACCCGATGGAA------------- 5666

B21_BF2_(AM282700) ------------------------------------------------------------ 3090

Fig. S2
